# Adaptive Planning Approaches for Coastal Climate Adaptation: Process and Key-elements

**DOI:** 10.1007/s00267-025-02117-1

**Published:** 2025-01-21

**Authors:** Sofia Valente, Paulo Pinho

**Affiliations:** CITTA - Research Centre for Territory, Transports and Environment, Department of Civil Engineering, Faculty of Engineering, University of Porto, Porto, Portugal

**Keywords:** Adaptive planning and management approaches, Adaptation pathways approach, Coastal climate adaptation, Changing coastal risks, Flood risk management

## Abstract

The paradigm of Adaptive Planning and Management provides several methodological approaches for designing robust adaptive plans to cope with uncertain future changes, namely the Adaptation Pathways’ method (APs). These approaches, particularly those containing APs, have captured increasing interest in the field of coastal climate adaptation as useful for guiding its planning and management. While these approaches have been tested in several research cases, there are still few real cases of application into coastal spatial planning instruments. Furthermore, the lack of implementation of coastal adaptation actions in urbanized coasts worldwide, so-called adaptation gaps, points to the need of investigating to what extent these Adaptive Planning approaches containing APs are being applied in coastal plans. A deeper analysis of cases of application of these approaches in coastal plans is required to understand how adaptive plans are being crafted. This article focusses on the two major cases of application of APs-based Adaptive Planning and Management approaches into planning and management instruments – the Thames Estuary 2100 Plan and the Delta Programme – to identify what elements were essential to design an adaptive plan and operationalize an Adaptive Planning and Management approach, including ingredients that the plan had to meet to be robust and adaptive. Our results suggest that at least five elements are required to craft and deliver a robust adaptive plan and accomplish a real Adaptive Planning and Management.

## Introduction and Objectives

Coastal climate adaptation is often surrounded by deep uncertainties about future effects of climate change (CC) and sea-level rise (SLR), their magnitude and rate; lack of agreement on what coastal adaptation exactly is, how to tackle SLR, what adaptation measures apply, when, where; multi-scalar complexity; ambiguity associated with multiple actors, making it a “wicked problem” par excellence (Moser et al., 2012; Brown et al., 2014).

Over the last 25 years, the deep uncertainties about future conditions and changes have led to the emergence and consolidation of a new paradigm of methodological approaches known as *Decision-Making under Deep Uncertainty* (DMDU), also called *Adaptive Planning and Management* (AP&M)^1^. AP&M approaches aim to develop a robust adaptive plan, i.e. a plan that is both robust, i.e. performs well across a wide range of plausible future scenarios, and adaptive, i.e. designed to be adapted/changed over time as new information arises or conditions change, thus, it can “survive” change (Walker et al., 2013; Haasnoot et al., 2013; Jeuken et al., 2014; Kwakkel et al., 2015; Ranger et al., 2013; Maier et al., 2016; Marchau et al., 2019). In AP&M, a plan holds properties of dynamic robustness, flexibility, and adaptability, which may also apply to plan’s measures or to the targeted system.

Dynamic robustness refers to a plan’s ability to perform acceptably well across a wide range of plausible futures and unforeseen conditions, rather than optimization for a single scenario, but also designing plans that can be adapted as changes and new knowledge emerge. Unlike a static robust plan, a dynamic robust plan explicitly accounts for its own adaptation from the outset, making it better suited to manage unpredicted and evolving conditions (Jeuken et al., 2014; Sayers et al., 2012; Walker et al., 2013; Marchau et al., 2019). This shifts the focus from finding a single optimal solution to creating a plan capable of evolving in response to changing circumstances (Kwakkel et al., 2015; Ranger et al., 2013).

Flexibility relates to the ability to adjust a plan if the future unfolds differently than expected or conditions change (Sayers et al., 2012; Jeuken et al., 2014). It involves the capacity of rapid or easy modification or adjustment of measures or tools. A flexible plan or measure can be easily modified in real time e.g., employed differently, continuing to meet objectives as changes occur (Marchau et al., 2019; Haasnoot et al., 2013). Flexibility often implies avoiding measures that foreclose future options, keeping options open, a phased implementation of various measures rather than a single action, linking short-term decisions to long-term options, using low-regrets measures, reversibility or correctability.

Adaptability refers to the ability to change/adapt a plan, its goals, measures, or methods, over the mid- to long-term, based on evolving knowledge and ongoing revaluation of decisions and priorities. Adaptability does not involve a one-time procedure, immediate on-the-spot flexibility or short-term responsiveness, instead, its emphasis is on longer timeframes, implying ongoing learning (Marchau et al., 2019; Haasnoot et al., 2013; Walker et al., 2013).

AP&M paradigm provides several methodological approaches for designing robust adaptive plans, among them the Adaptation Pathways (APs) – a method for exploring and sequencing sets of possible measures based on external changes over time (Haasnoot et al., 2013, 2012). Central to APs approach is the concept of Adaptation Tipping-points (ATPs)—conditions under which a measure no longer meets predefined objectives, and a new measure is required (Kwadijk et al., 2010). A pathway is a sequence of possible actions over time: when the ATP of a given action is approaching (i.e. once the predecessor action is about to reach its “sell-by date”), a new action is needed, and, hence, a pathway arises. The APs usually produces a set of pathways represented in a map, like a metro-map or decision-tree.

The APs approach explicitly uses a multiplicity of plausible future scenarios, namely “transient scenarios” outlining unfolding developments over time, to assess the performance of measures and the moment of their ATP. Moreover, APs-based approaches require a monitoring and reevaluation system, including indicators and triggers that signal when to activate new measures (Haasnoot et al., 2013). Most AP&M approaches entail an iterative cycle, so-called “iterative risk management,” involving several inter-related steps, which enable ongoing learning and adaptations over time, based on evolving knowledge and changes (Ranger et al., 2010).

Within the family of AP&M, the APs method, and approaches containing it, have captured increasing interest in climate adaptation literature as useful for streamlining and guiding adaptation planning and implementation in contexts marked by uncertainty, complexity, and change (Wise et al., 2014). Nevertheless, these approaches require further on-the-ground exploration and application in distinct contexts and policy domains (Marchau et al., 2019), particularly in coastal spatial planning and management (Ranger et al., 2013; Van der Voorn et al., 2017).

Although APs-based AP&M approaches have been increasingly tested in diverse coastal settings (Oppenheimer et al., 2019), real-world applications in coastal risk management plans/policies are still limited. There is a lack of comparative assessments of the use of differentiated AP&M methods containing APs by policymakers and decision-makers involved in coastal management (Lin et al., 2017, Bloemen et al., 2018).^2^ Despite the growing number of applications of APs method reported in literature, it is still necessary to systematically review, and compare cases of APs design and use (Werners et al., 2021), particularly in coastal adaptation realm.

Within the literature on APs-based AP&M approaches for coastal adaptation purposes, three main types of research studies/strands of analysis can be distinguished: (i) research studies that are theoretically-driven and focus on developing, improving or advancing the methodological approaches themselves and/or their tools, to support the design of long-term robust adaptive plans/policies (e.g. Walker et al. 2013, 2001, Kwadjik et al. 2010, Haasnoot et al. 2013,2012, Kwakkel et al. 2015, Stephens et al. 2017, Haasnoot et al. 2020a, b, Schlumberger et al. 2022), (ii) research studies focused on experimenting and testing these novel approaches in either hypothetical cases, in cases in real world contexts, or coastal territorial archetypes, which, in different ways, bring new insights to the theorization on the approaches themselves, or contribute to enhance their applicability and usefulness (e.g. on hypothetical cases: Haasnoot et al. 2012b; on research/study cases in real contexts: Barnett et al. 2013, Campos et al. 2016, Gray et al. 2016, Veelen and Meijer 2016, Aerts et al. 2018a, Ramm et al. 2018, Zandvoort et al. 2019, Bosomworth and Gaillard 2019, Kool et al. 2020, Haasnoot et al. 2020c, Jenewein and Hummel 2022, Colloff et al. 2024; and on coastal archetypes: Rocle et al. 2020, Magnan and Duvat 2020, Haasnoot et al. 2019, 2021a, b, Toimil et al. 2021, Muccione et al. 2024); (iii) evidence-based analyses and studies documenting, reporting, or evaluating real-world practical examples of application of APs-based AP&M approaches into coastal planning and their instruments (e.g. Reeder and Ranger 2011, Ranger et al. 2013, Penning-Rowsell et al. 2013, Ramsbottom et al. 2018, Rosenzweig et al. 2011, Haegen and Wieriks 2015, Gersonius et al. 2016, Werners et al. 2016, Alphen 2016, Brugge and Bruggeman 2019, Alphen et al. 2022, Lawrence et al. 2019a, b, c, Ryan et al. 2021, and some developing a more critical analysis of such cases, e.g. Klijn et al. 2015, 2016, Buuren et al. 2016, Restemeyer et al. 2017, Bloemen et al. 2019). Within this latter typology, some studies have sought to compare and contrast (two or more) real-world examples of application of AP&M approaches containing APs in plans/policies for coastal adaptation purposes, identifying similarities and differences, or distilling lessons or issues for further research (Jeuken et al., 2014; Jeuken and Reeder, 2011; Van der Voorn et al., 2012; Zevenbergen et al., 2018; Zandvoort et al., 2018; Lin et al., 2017; Bloemen et al., 2018).^3^ This article falls within this latter typology.

To date, the two major applications of AP&M approaches containing APs into planning instruments that have been implemented, for coastal climate adaptation or flood risk management (FRM) purposes, are the Thames Estuary 2100 Project (TE2100) and the Delta Programme 2014 (DP2014).^4^ Focusing on these two cases, this article aims to investigate what elements of the AP&M approach are essential to develop and operationalize a robust adaptive plan/strategy and carry out an Adaptive Planning and Management approach (research question).

## Methodology

To answer this research question, two tasks were undertaken:

1. Analysis of the planning instrument (plan/policy-program) in which the AP&M approach was developed and applied to plan for coastal adaptation/risk management under uncertain future changes. This task analyzed how such approach has been devised and implemented in each case. It implied a content analysis of the plans (TE2100 Plan, DP annual reports), and a review of associated documents, including scientific and grey literature. Bibliographical references on “Thames Estuary 2100 Plan and Project” and “Delta Programme” were compiled from searches on Web of Science and Scopus databases and Google.
2. Identification of key-elements of AP&M necessary and essential for developing and operationalizing a *robust adaptive plan*. Drawing on insights from the first task, the second task involved searching for references to key-concepts like *robust*, *dynamic robust*, *adaptive*/*adaptable*, *adapt*, *scenario(s)*, *flexible*, *uncertain*, *change*, *threshold, tipping-point*, and *pathway*, across the reviewed bibliographical sources, extracting and deriving elements fundamental to craft and concretize a robust adaptive plan/strategy. Besides, a literature review was conducted on this topic, which helped to uncover the key-elements. Hence, a mixed method was employed, involving primarily an induction exercise (drawing insights from the studied cases, their practical experiences, and literature on them), followed by a deduction exercise (confronting prior results with theoretical perspectives, scientific knowledge and debates on component/requisite elements of AP-based AP&M), which, together, led to the final systematization of the key-elements.

The mixed method used (induction-followed-by-deduction exercise), which is mainly evidence-based but informed by theoretical perspectives to confirm findings, contrasts with traditional methodologies that first propose theoretically driven assumptions about how a robust adaptive plan should be, or what are its required components/building-blocks, and then assess whether different cases meet them (e.g. Jeuken et al. 2014, Restemeyer et al. 2017). The method used not only enriches the debate on key-elements of AP&M, but also helps to bridge gaps between theory and practice, highlighting difficulties and challenges in real-world applications, and fostering mutual learning between practitioners and AP&M scholars.

The case-studies section is structured as follows:

- *Section “The Adaptive Planning and Management approach devised”* presents the AP&M approach devised and applied.
- *Section “How the Adaptive Planning and Management approach was applied: process of steps”* outlines the plan development process.
- *Section “Key-elements essential to develop and operationalize an adaptive plan / strategy”* identifies essential elements of the applied approach for developing and implementing an adaptive plan.

Each section addresses the two cases, first the TE2100, then the DP2014, followed by a joint sub-section of comparative analysis.

## Case-studies: Thames Estuary 2100 Plan, and Delta Programme 2014 for 2015

### The Adaptive Planning and Management approach devised

#### TE2100’s dynamic adaptive planning approach

The UK’s Environment Agency (EA) initiated the TE2100 Project in 2002 with the aim of developing a long-term FRM plan for the Thames Estuary (TE) up to 2100, and address climate adaptation as a central issue (Environment Agency UK, 2009a; Environment Agency UK, 2009b; Environment Agency UK, 2012). The final Plan, issued in 2012, lays down the possible *Options* (pathways) to manage flood risk throughout the century.

Given the uncertain future conditions (CC-related flood risk) and the high stakes involved, the TE2100 Team developed a new approach to plan for FRM based on the concept of *Dynamic Robustness*^5^. Dynamic Robustness involves building a plan that is *robust* (i.e. performs well against several decision criteria under a wide range of possible future states of the world) and *flexible* (i.e. designed to be changed/adjusted over time as new knowledge or changes arise, therefore, *adaptable*) (Environment Agency UK, 2009a; Environment Agency UK, 2012; Ranger et al., 2013).

Drawing on Dynamic Robustness, the TE2100 developed its *Dynamic Adaptive Planning approach*, also denominated *Managed Adaptive* or *Iterative Risk Management approach*, where the plan (its measures) is implemented iteratively over time to keep risk below acceptable levels, and at the same time keeping options open to manage future risk (Ranger et al., 2013; Reeder and Ranger, 2011; Ramsbottom et al., 2018).

#### TE2100’s adaptation pathways approach

**Fig. 1:**
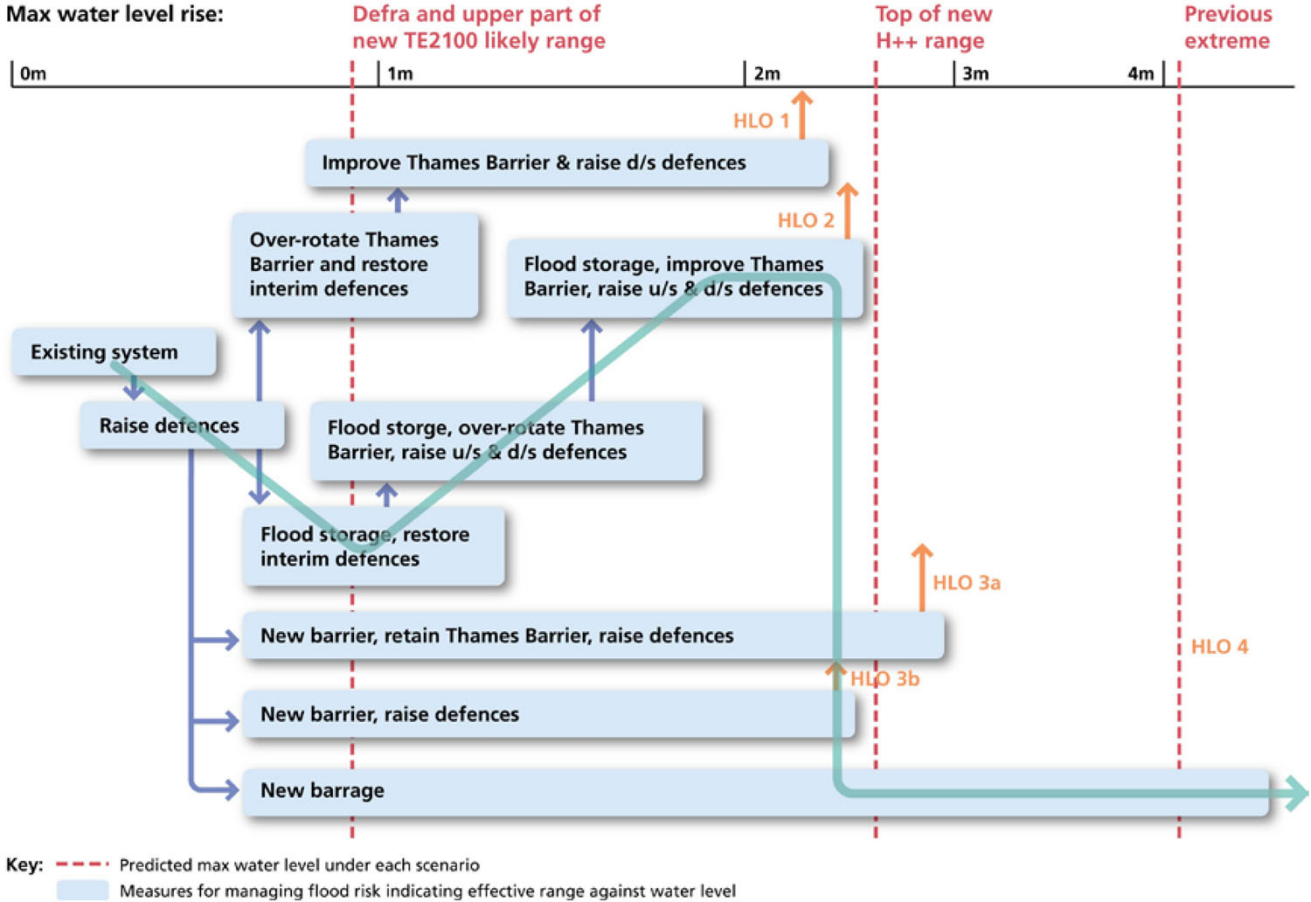
The original (first) route-map developed for the TE2100 Plan, showing the High-Level Options (HLOs) – i.e. pathways on the *Y*-axis, in relation to threshold levels in terms of rising water levels in the TE (on the *X*-axis). The blue line shows a possible pathway/route made up of several pathways (initially, it would follow HLO2, and subsequently, if SLR occurred faster than expected, it would switch to HLO4). Plausible future SLR scenarios are illustrated (vertical dashed red lines), e.g.: *DEFRA* (0,9 m), initial *High*+*+* (4,2 m), and *High*+ (2,7 m. i.e. top of new High++). Source: Reeder and Ranger 2011. © Environment Agency copyright and/or database right 2024. All rights reserved. NB this diagram includes *Adaptation Options* that were ruled out before publication of the Thames Estuary 2100 Plan in 2012. In this route-map, an HLO/Option refers to a set of portfolios that, when implemented in sequence, offer a comprehensive FRM solution over the next 100 years; a “portfolio” consists of a number of responses combined that offer a solution for a specific increase in the sea level/water level; a “response” is an individual FRM measure, e.g. a barrier, a raised defense (Environment Agency UK, 2012; Bloemen et al., 2018). Each HLO can be adapted according to the rate of change (water level rise) observed, e.g.: in HLO1, a 0,20 m SLR implies raising small defenses; a 0,6 m SLR implies over-rotating the TB and restoring interim defenses; a 0,9 m SLR implies improving the TB and raising downstream defenses (Environment Agency UK, 2009b)

As part of its Dynamic Adaptive Planning approach, the TE2100 developed a novel method for designing a dynamic robust adaptive plan – the *Route-map* or *Decision pathways approach*, later called *Adaptation Pathways approach* (APs) (Environment Agency UK, 2009b; Ranger et al., 2013; Reeder and Ranger, 2011).^6^ The APs allowed developing several possible pathways, so-called *High-Level Options* (*HLOs*, later *Options*), and representing them in a route-map. Each HLO (pathway) is a package of FRM measures sequenced that can be implemented over time to manage risk stepwise (Environment Agency UK, 2009a; Environment Agency UK, 2009b; Environment Agency UK, 2012; Ranger et al., 2013; Reeder and Ranger, 2011; Ramsbottom et al., 2018). Each pathway is made up of a sequence of various measures—individual measures, or “portfolios” of measures – that act together to reduce risk to an agreed level over the century (Environment Agency UK, 2009a; Environment Agency UK, 2012; HM Treasury, 2009). The route-map (Fig. 1) shows five possible alternative HLOs, in relation to critical threshold-levels indicated in the *X*-axis (which expresses water level rise/SLR). It also illustrates the main SLR scenarios considered. Each measure (individual or portfolio) is represented in a box that finishes at the SLR level at which it ceases to be effective; the arrows link to other measures available that may be implemented before the prior measure reaches a threshold.

Thresholds consisted of critical levels, i.e. key threshold-values for the FRM system, its sensitivity and vulnerability, e.g. the level of SLR at which preexisting sea defenses fail, levels that pose limits to adapting existing defenses (walls and embankments), the level of SLR at which the Thames Barrier as designed no longer meets the target protection level, the engineering limit of the Thames Barrier with retrofitting works, the limit at which retreat will be required. The Team had to examine key thresholds in terms of water levels at which other measure or Option will be required (Reeder and Ranger, 2011; Ranger et al., 2013).

The Dynamic Adaptive Planning approach implied identifying several FRM measures, exploring their possible sequencing and determining the critical conditions under which a measure no longer meets predefined criteria, and a new measure is needed. In particular, the APs method required identifying the threshold of, and sequencing, possible FRM measures over time, and thereby developing several pathways (HLOs) (Ranger et al., 2013), in ways that allow the system to be adapted to changes over time, and in which alternatives are left open to cope with multiple plausible future conditions (Reeder and Ranger, 2011). The idea was to design packages/sets of adaptation measures that may be implemented over time, ensuring that such pathways cost-effectively reduce risk while being flexible and adaptable to future changes. The *Options* were developed in an iterative process: different FRM measures were identified and tested to detect thresholds, then used to assemble various pathways, which were assessed on several criteria under different future scenarios, and progressively iterated and refined (Environment Agency UK, 2009a; Environment Agency UK, 2009b).

The series of pathways designed can span the range of plausible water level rise estimated for the TE until 2100, up to 4,2 m. The HLOs are different possible routes to manage flood risk over the next 100 years under the plausible SLR scenarios. Each HLO/Option is included in the final Plan, with a description and a georeferenced map.

**Fig. 2:**
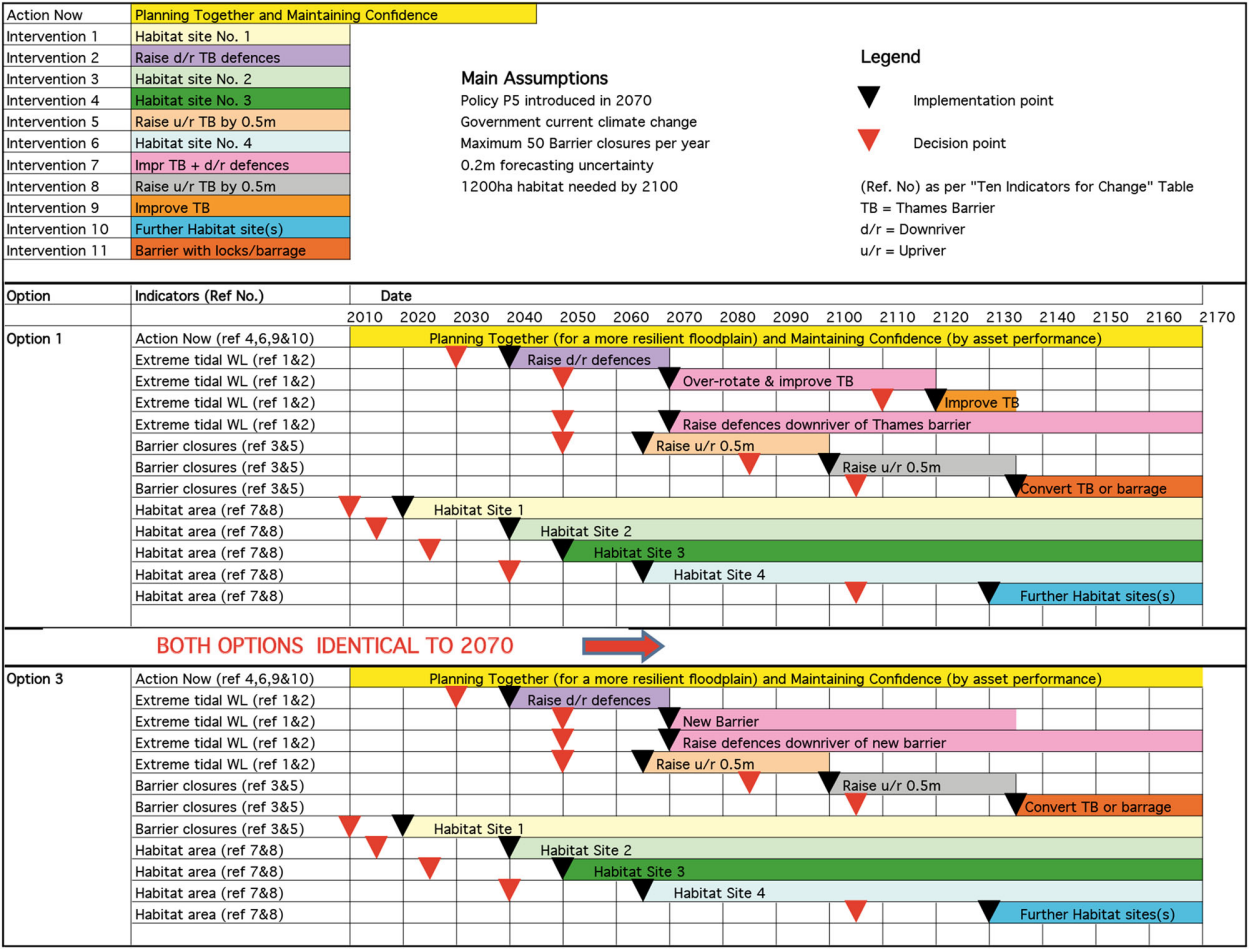
Two Options (pathways) designed in the TE2100 Plan (Environment Agency UK, 2012), including the decision-points and implementation-points. A decision-point is a point at which a decision on a given measure must be made, which triggers its planning and construction; its estimation is conditional on the monitoring of actual changes in indicators (e.g. recorded values of SLR), thus, the timing of this decision-point (and of the implementation-point) may change depending on the monitoring results; its estimation accounts for the lead-time required to construct or implement such measure. The illustrated Options, their design and timing, namely their decision-points, were superseded by updates made during the 10-Year Plan’s Review, which demonstrates the adaptive nature of the Plan. Source: Environment Agency UK (2012), p.38. © Environment Agency copyright and/or database right 2024. All rights reserved

**Fig. 3:**
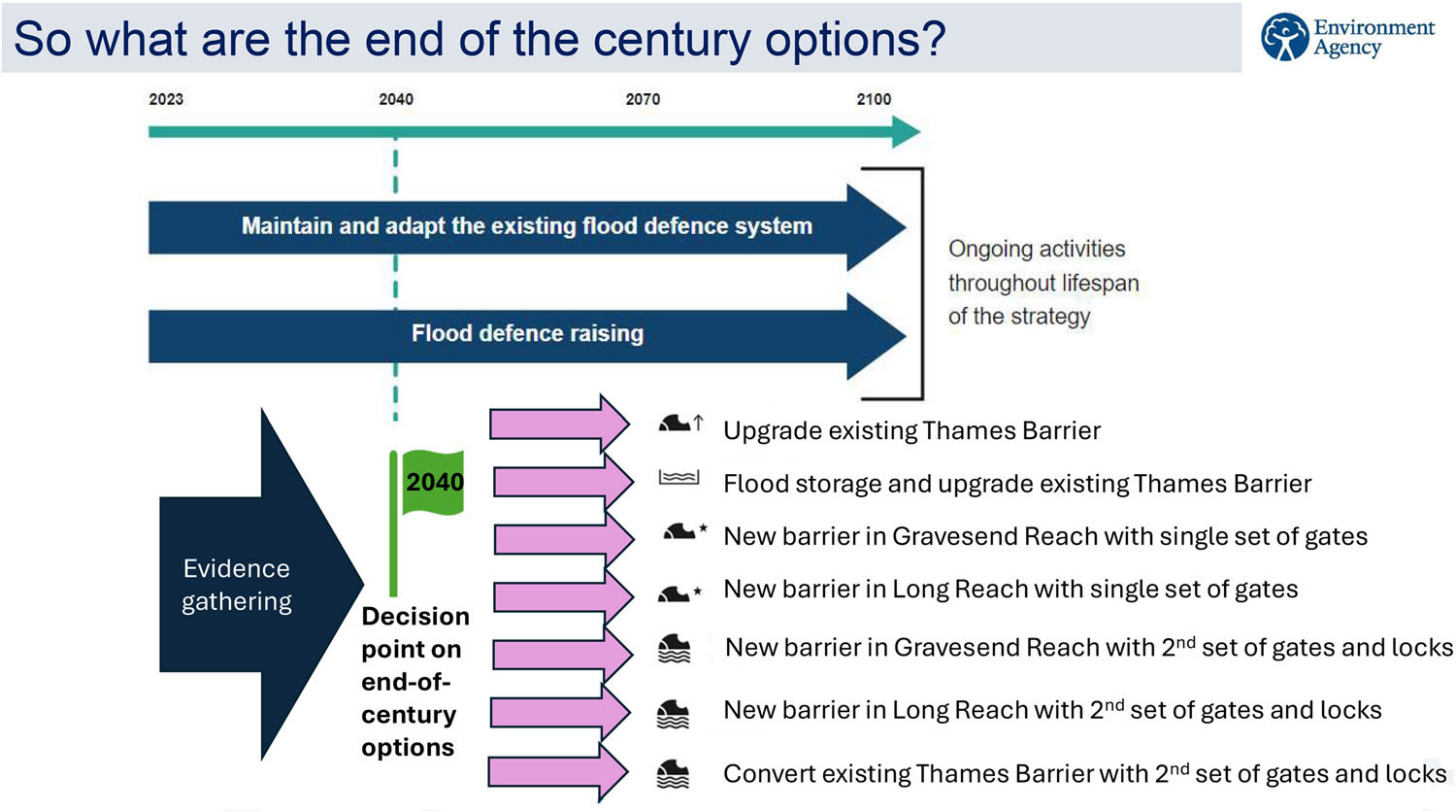
Recent map of adaptation pathways for the TE2100, showing the updated “End-of-the-Century Options”, i.e. the pathways devised for the long-term period (from 2023 up to 2100), and on which a decision will be made around 2040 during a major 10-year review of the Plan. In this map, the End-of-the-Century Options are displayed (*Y*-axis) against time. Source: Environment Agency, personal communication, October 8, 2024

A key feature of the HLOs/Options is that they can be adapted to respond to change. The HLOs/pathways are flexible since it is possible to move from a measure to another within a pathway, but also move from a pathway to another one, depending on the actual rate of change. In addition, the timing of new measures, and the measures themselves, can be adjusted over time, building in flexibility into the broader pathway. Overall, the APs method allows for switching of measure or pathway, or adjusting measures and their timing, thereby safeguarding the Plan’s robustness and flexibility (Environment Agency UK, 2009a; Environment Agency UK, 2009b; Environment Agency UK, 2012; Ramsbottom et al., 2018; Lowe et al., 2009; Ranger et al., 2013; Reeder and Ranger, 2011) (Figs. 2 and 3).

#### DP’s adaptive delta management (ADM)

The DP, a Dutch national policy-program initiated in 2010, aims to ensure sustainable and robust FRM, freshwater supply (FS), and spatial adaptation, now and in the future (DP Delta Programme, 2013; DP Delta Programme, 2014). It was devised to develop “Decisions” and “Strategies” for FRM and FS, with 2100 as time-horizon, addressing uncertainties about future socioeconomic and climatic developments (Brugge and Roosjen, 2015; Gersonius et al., 2016; Alphen, 2016). It is elaborated yearly and subdivided into 9 Subprogrammes.^7^

Since its inception, the DP has developed and applied its own planning approach, called *Adaptive Delta Management* (ADM) (DP Delta Programme, 2010; DP Delta Programme, 2012; DP Delta Programme, 2013; DP Delta Programme, 2014), based on the Dynamic Adaptive Policy Pathways approach (DAPP) created by Deltares Institute and TU Delft and presented by Haasnoot et al. (2013), and inspired by the TE2100’s approach (DP Delta Programme, 2011).^8^ ADM was devised by Deltares and the DP National Staff: the first led its elaboration and offered guidance for its application in the DP (Deltares, 2018). The latter provided technical support to regional Subprogrammes and an “ADM Implementation Guide” (Rhee, 2012).^9^ ADM is founded on four core-principles (DP Delta Programme, 2012; DP Delta Programme, 2013):

1. Linking short-term decisions with long-term tasks (needs, goals) around FRM and FS.
2. Incorporating flexibility into possible solution-strategies.
3. Working with *multiple strategies that can be alternated between* i.e., *adaptation paths* (pathways) between which it is possible to switch, depending on developments.
4. Linking FRM and FS measures with other investment agendas.

Principle 2 could be achieved by e.g.: designing each strategy as a path; foreseeing a stepwise implementation of several measures throughout time; keeping options open; envisioning various paths (alternative pathways); allowing switching to other measures or paths, modifying measures, or altering their timing; using measures inherently flexible (e.g. sand nourishments); searching for short-term measures adequate in the long-term or measures that enhance system’s robustness (DP Delta Programme, 2011; DP Delta Programme, 2012; DP Delta Programme, 2013; DP Delta Programme, 2014).

Principle 3 implied developing *paths* with successive actions over time, instead of a single solution suited for a certain future point (Rhee, 2012; Klijn et al., 2016, 2015; Werners et al., 2016).

Principles 2 and 3 are related to robustness and flexibility. The ADM Implementation Guide required strategies to be both robust and flexible: a robust strategy is one that “works in all plausible futures”, whereas flexibility allows for cutting off a strategy and switching to another one if needed, depending on the contextual conditions (DP Staff member, *in* Restemeyer et al., 2017, p.930). According to the Guide, to develop robust flexible strategies, it is necessary to identify tipping-points and design adaptation pathways (Zandvoort et al., 2018; Restemeyer et al., 2017). Consequently, the DP assumed that its *Strategies* should be robust and flexible, and used ADM to devise them: a robust strategy is future-proof and provides a solution for tasks arising in all the scenarios considered (with it, objectives are met in the four *Delta Scenarios*), while a flexible strategy is one that can be easily speeded up/slowed down and that allows shifting to another measure or path, if necessary (DP Delta Programme, 2013; DP Delta Programme, 2014).^10^

The DP issued the final *Delta Decisions* (DD) and *Preferential Strategies* (PS) in the DP Delta Programme (2014). The first are structuring decisions on FRM and FS. The latter specify concrete measures for each region. The four ADM principles substantiated the development of DD and PS. Though the ADM principles seem obvious, their practical application in the DP was not simple nor without intricacies. To support their application, two methods were used: ATPs (Kwadijk et al., 2010) and Adaptation Pathways (APs) (Haasnoot et al., 2013; Haasnoot, 2013).

#### ATPs and APs within DP’s ADM

The ADM Guide explicitly recommended working with Scenarios, Tipping-points, and Adaptation Pathways, to make strategies robust and flexible (Restemeyer et al., 2017; Zandvoort et al., 2018).

In ADM, ATPs are understood as “points where the magnitude of change due to socioeconomic developments, CC, or SLR, is such that the current strategy will no longer be able to meet the objectives” (Haegen and Wieriks, 2015, p.51), or conditions under which a given measure ceases to be effective or acceptable and a new measure is required (Rhee 2012, *in* Zandvoort et al. 2018, Zevenbergen et al. 2018). The design of pathways implies identifying ATPs i.e., analyzing at which level of a climate-related variable, or other parameter, a given measure is no longer suitable or acceptable and a transition of measure is needed, and then placing such ATP against different future scenarios to determine its date (Klijn et al., 2015). By crossing ATPs with scenarios, it is possible to obtain information on the timing for new measures.^11^

The DP defined tipping-points as points at which the existing system ceases to meet the requirements; an ATP occurs when, due to climatic or socioeconomic changes, a measure, policy or infrastructure becomes insufficient to comply with defined criteria or agreed standards (due to physical, technical, financial constraints or socially unacceptable effects); hence, ATPs’ analysis helps determining when new measures and decisions must be taken (DP Delta Programme, 2011).

Within ADM, the APs is an adaptive approach for designing strategies as pathways, which allows for switching between measures or strategies, if necessary, in view of uncertain future changes. A key step in ADM process is developing pathways. In the DP, the APs method involved: mapping available measures, assessing them, specifying their tipping-point whenever possible, determining when this might occur at earliest and latest, and analyzing whether it is necessary to adjust such measure or shift to another measure (Klijn et al., 2015). Each pathway is a sequence of actions over time to achieve predefined objectives (Zevenbergen et al., 2018; Gersonius et al., 2016). The pathways offer different possible trajectories the system may follow (Zandvoort et al., 2018).

**Fig. 4:**
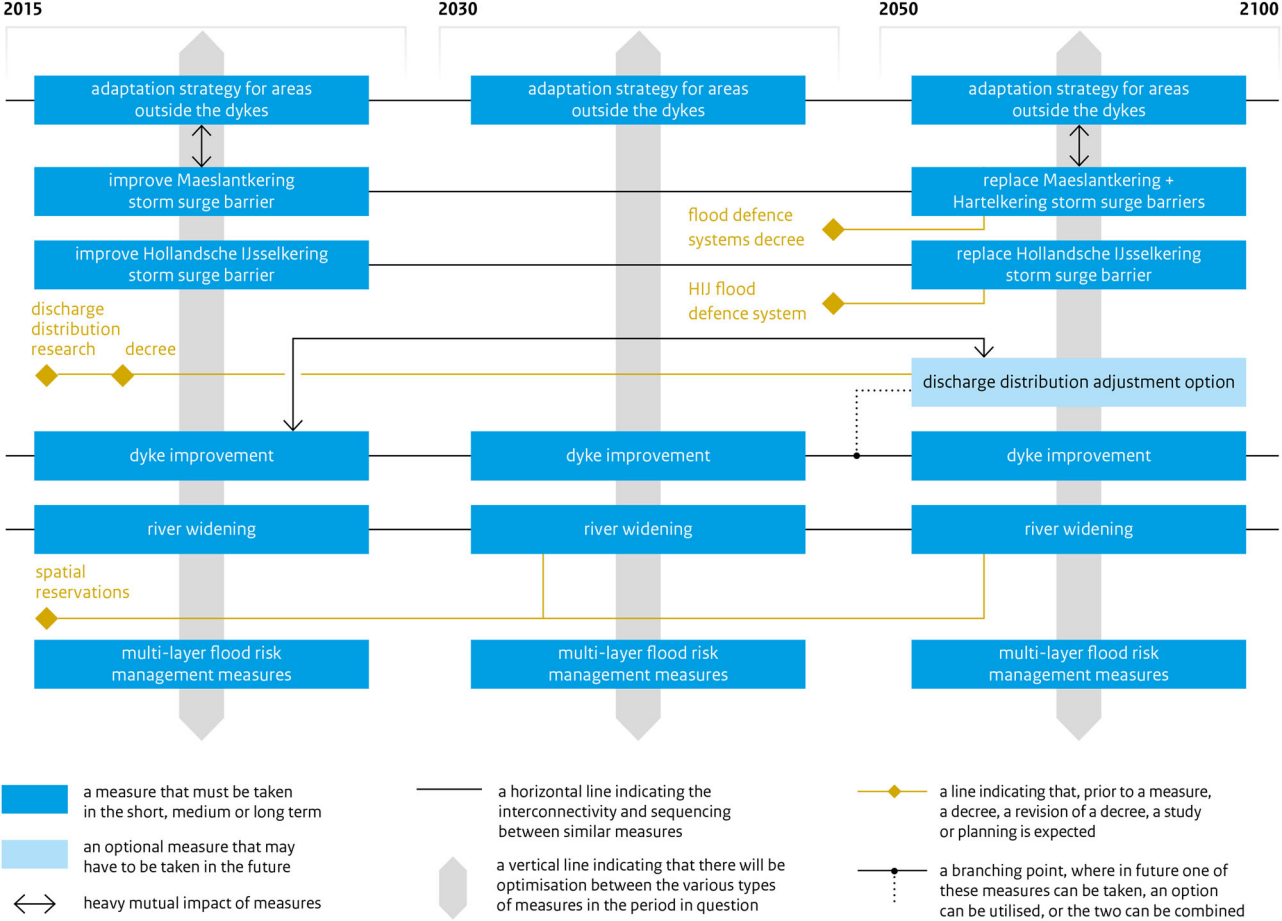
Adaptation path of the Preferential Strategy for FRM for the Rhine Estuary-Drechtsteden (developed by the RE-D Subprogramme). Source: DP Delta Programme (2014) (for year 2015), p.66. The map illustrates the measures that must be taken (blue boxes) at three time periods. The light blue boxes show possible options (alternative measures) for the future. In this case, the only alternative option considered is modifying the discharge distribution along the Rhine River branches. The yellow diamonds represent ‘preparatory actions’ (e.g. the revision of the decree, a study, the definition of ‘spatial reservations’ required for river widening; or research on the costs and technical feasibility of a possible measure). The arrows show a strong influence between measures’ effectiveness (e.g. if research into the possible modification of the discharge distribution results in a decree for its implementation, then the design of ‘dyke improvement projects’ will need to take into account the effects of such measure) (Bloemen et al., 2018, p.11). The branching point shows a point where it will be necessary to select one option (opt for a measure or use both measures); it is like a bifurcation point where either one option or a combination of options can be chosen. The yellow diamond indicates the need for a decision before the measure can be implemented. Each horizontal line represents a measure (or measures) that will be implemented in a sequenced way; while the grey vertical stripes show measures that will be implemented in the same time-period (Restemeyer et al., 2017). The vertical grey stripes demonstrate that there is optimization between the various types of measures in the same period (Bloemen et al., 2018, p.11)

Principle 3 translated in the design of *adaptation paths* within each *Preferential Strategy* (PS) for FRM of each regional Subprogramme. In the DP Delta Programme (2014), each PS contains a map of *adaptation paths*, with one or more preferred *paths* (Fig. 4); specifically, each map indicates what measures can be taken and when these are expected to be necessary, across three time-periods (DP Delta Programme, 2014). A *path* is a pathway i.e., a logical set of measures including measures for the short-term and options for the mid- and long-term, and measures required now to keep options open in the future. Each map may display several alternative paths or a single preferred path. In each map, depending on climatic and socioeconomic developments, measures may be taken sooner or later, and there are possibilities of switching of measure or *path* (DP Delta Programme, 2014). APs method granted flexibility by allowing shifting between different measures or *paths*, changing the timing for measures, and keeping options open (Bloemen et al., 2018; Haegen and Wieriks, 2015). The main outcome of ADM was a single path, or set of paths, that schedule measures over time, i.e. ADM led to “a composite strategy, or a set of alternative strategies with intermediate possibilities for revisions” (Rhee 2012, *in* Zandvoort et al. 2018, p.192).

**Fig. 5:**
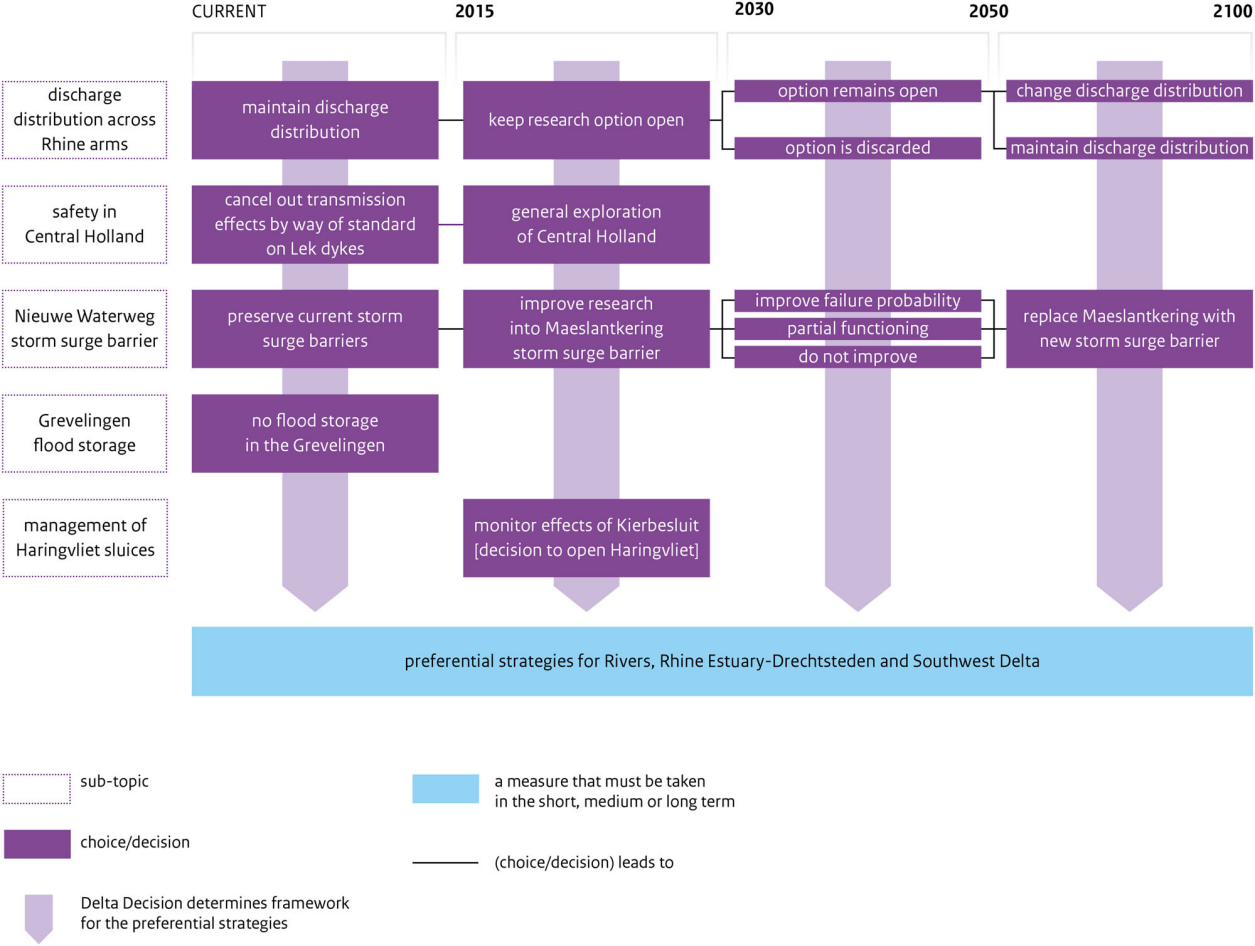
Adaptation path of the Delta Decision on the Rhine-Meuse Delta (developed by DP national staff). Source: DP Delta Programme (2014) (for year 2015), p.39

**Fig. 6:**
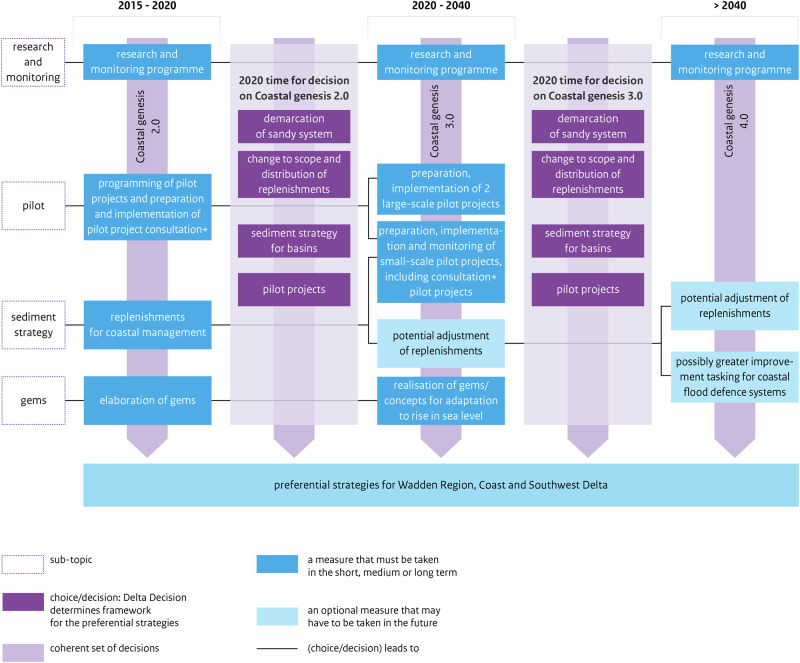
Adaptation path of the Decision on Sand (developed by DP national staff). Source: DP^2014^ (for year 2015), p.42

The DP National Staff developed the DD (Figs. 5 and 6). In three DD and all PS, there is a map of adaptation paths. Both DD and PS reflect ADM principles.

In the DP, the design of *adaptation paths* involved exploring possible measures and combinations of measures, devising diverse sequences to cover the range of *Delta Scenarios* and visualizing possibilities to alternate. The map of *paths* served to identify various measures, sequence them, and explore possible shifts between them (Werners et al., 2016; Brugge and Bruggeman, 2019; Zandvoort et al., 2018). Designing adap*tation paths* also required studying the conditions under which changing or switching of measure or strategy is reasonable (DP Delta Programme, 2011; DP Delta Programme, 2012; DP Delta Programme, 2013). The calculation of the moment for switching to a new measure accounted for the lead-time needed for deciding and constructing it, and for triggers that warn of upcoming ATP. The analysis of ATPs, though not easy, was important to design *paths*.

The DP, initially, examined how long the current policy will suffice and when the tipping-point will be reached in different scenarios (DP, 2010). In 2010, DP assessed how much longer the current policy and management measures will be satisfactory, and when adjustments will be required, i.e. when the first ATPs for the existing system will be reached, using diverse scenarios (four climatic scenarios and four socioeconomic scenarios, later combined into four *Delta Scenarios*) (DP Delta Programme, 2010). In 2010/2011 and 2011/2012, to develop their *adaptation paths*, Subprogrammes investigated conditions under which it is logical to move from a measure to another, and at what times new measures will be required, explored and assessed other possible measures under the *Delta Scenarios* (DP Delta Programme, 2012). In practice, the DP and Subprogrammes sought to identify ATPs as points at which the objectives of FRM policy along the coast and rivers are no longer met, or under which existing management strategies are no longer able to meet objectives and alternative strategies are needed, and then analyze when these ATPs might be reached (Jeuken and Reeder, 2011). In the DP, ATPs describe conditions under which the current or alternative management measures or policies fail and are often related with acceptable flood return periods which were translated into design criteria for defenses (updated standards expressed as a flood probability) (DP Delta Programme, 2012; Jeuken et al., 2014).

#### Comparative overview

Both cases deliberately developed and applied an AP&M methodology to create robust adaptive plan/strategies under uncertain future changes. The TE2100 introduced an innovative approach based on Dynamic Robustness, denominated Dynamic Adaptive Planning, which encompassed APs method (initially called *Route-map* or *Decision Pathways*) and threshold analyses (also called “scenario-neutral threshold approach”). The DP2014 devised Adaptive Delta Management (ADM) based on Dynamic Adaptive Policy Pathways (DAPP) approach (Haasnoot et al., 2013) and the TE2100 case. Notably, the Netherlands pioneered AP&M adoption to an entire country and all its regions and integrating the multiple objectives of FRM, FS and spatial adaptation. ADM, especially the ADM Implementation Guide endorsed using Scenarios, ATP and APs methods, to make robust flexible strategies.

The TE2100 produced a route-map with five main alternative pathways, which together can cover a range of SLR scenarios up to 4,2 m by 2100; the *X*-axis represents water level rise and displays critical threshold-values. In DP (2014), each PS and three DD present a map with one or more preferred paths, with horizon-years in the *X*-axis. Overall, ADM led to a composite path or a set of alternative paths, scheduling measures over three time-periods, and allowing for adjustments over time.

These cases illustrate two main ways of representing pathways in maps: (1) a single map with threshold-values on the *X*-axis (less dependent on probability distributions over scenarios), as the TE2100’s route-map; or (2) a map with time-horizons on the *X*-axis (a map produced per each scenario considered, as in DP, where Subprogrammes initially considered the four *Delta Scenarios*, but then evaluated their PS under the most and less extreme *Delta Scenarios*, i.e. *Steam* and *Rest*). These two ways of developing APs maps are explained in Deltares’ “Pathways Generator” (https://publicwiki.deltares.nl/display/AP/Pathways+Generator).

### How the Adaptive Planning and Management approach was applied: process of steps

**Fig. 7:**
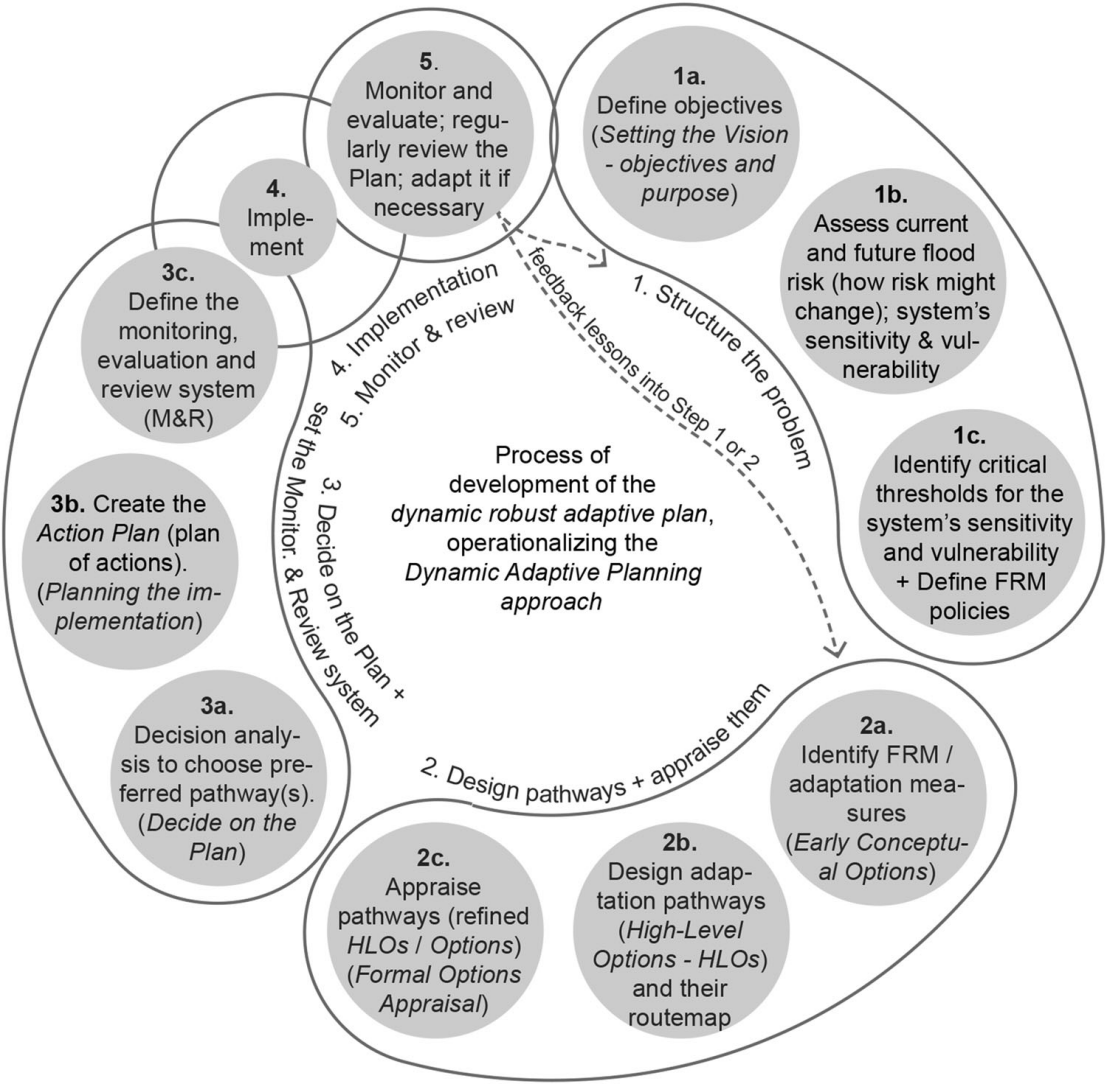
Planning process of the TE2100, showing the various steps of development of the Plan, in line with a “decision-centric” process. Source: own elaboration, based on Environment Agency UK (2012), Environment Agency UK (2009a), Environment Agency UK (2009b), Ranger et al. (2013, 2010), Reeder and Ranger (2011), Ramsbottom et al. (2018); Lowe et al. (2009), HM Treasury (2009), Penning-Rowsell et al. (2013), London Councils’ TEC Executive Sub Committee (2007), London Councils Member briefing (2018), etc. See more in Supplementary Material (Note 1)

The TE2100’s Dynamic Adaptive Planning approach entailed an iterative process of planning, decision-making and risk management, also deemed a decision-centred (policy-first, context-first) planning process (Ranger et al., 2013, 2010; Reeder and Ranger, 2011). Figure 7 outlines how the TE2100’s Dynamic Adaptive Planning approach was applied to develop and implement an adaptive plan, and how the pathways were designed.

**Fig. 8:**
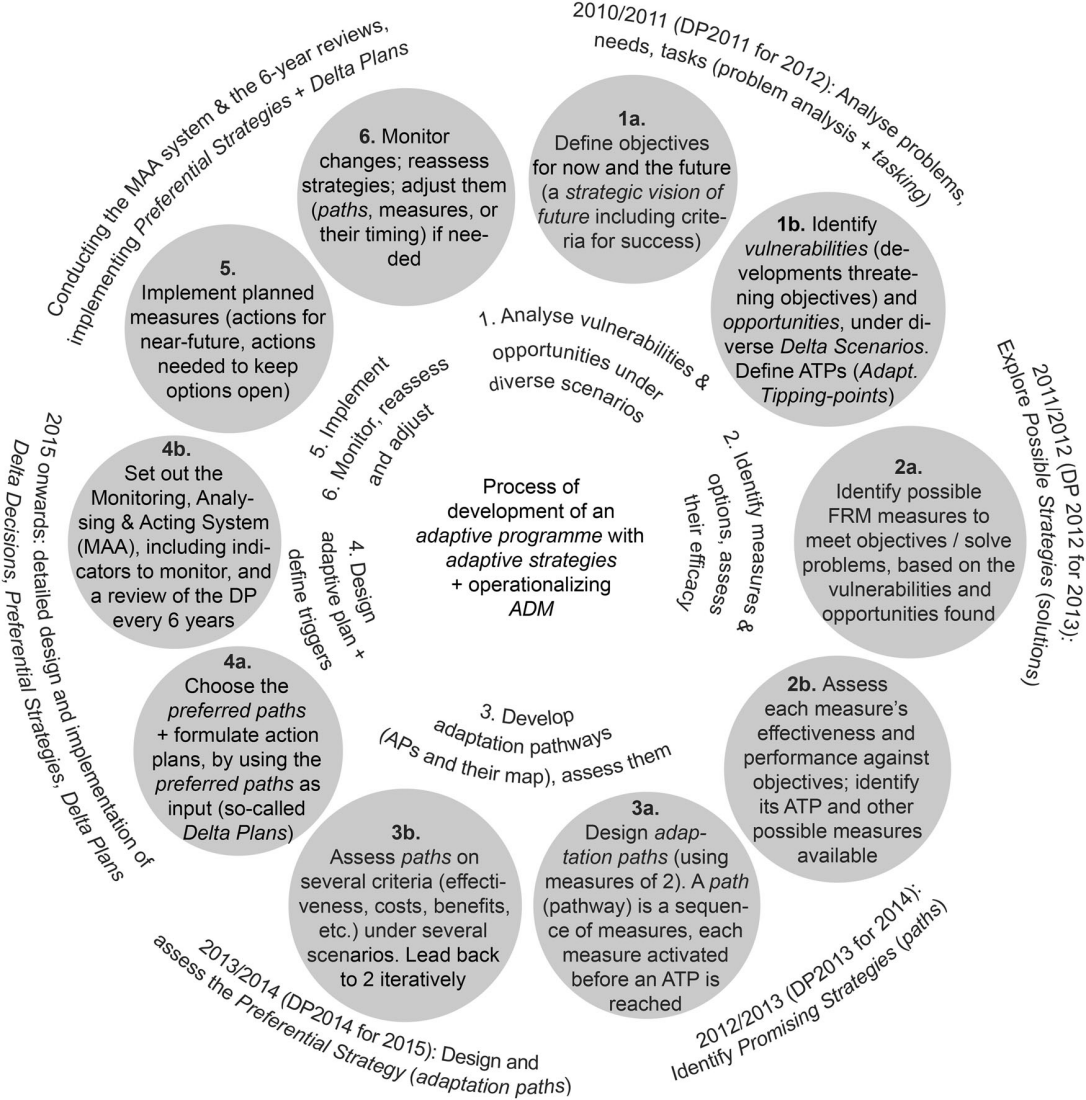
ADM process in the DP, including the development of *Strategies*. Source: own elaboration, based on DP Delta Programme (2011), DP Delta Programme (2012), DP Delta Programme (2013), DP Delta Programme (2014), DP Delta Programme (2016), etc. See more in Supplementary Material (Note 2)

In its turn, ADM defines its own process to develop and implement an adaptive strategy (Deltares 2018‐online, adapted from Haasnoot et al. 2013, Brugge and Bruggeman 2019‐online, Jeuken et al. 2014). It is based on the process of DAPP approach (Haasnoot et al. 2013). It is a phased cyclical process with 6 main steps inspired in DAPP. Figure 8 illustrates the ADM process as undertaken in the DP: it identifies its main steps and how these have been carried out, and how a robust flexible strategy has been developed. It builds on the analysis of DP Reports (DP Delta Programme, 2012; DP Delta Programme, 2013; DP Delta Programme, 2014; DP Delta Programme, 2016; DP Delta Programme, 2017; DP Delta Programme, 2018) and prior studies (Restemeyer et al., 2017; Gersonius et al., 2016; etc.).

The DP and Subprogrammes applied ADM to draw up Delta Decisions (DD) and Preferential Strategies (PS), respectively.

#### Comparative overview

The TE2100 and Delta Programme (2014) cases show, in two different ways, how a dynamic robust adaptive plan/strategy has been created and implemented, and two slightly distinct AP&M processes. In both cases, the devised AP&M approach entails an ongoing iterative process of interrelated steps to develop and operationalize a robust adaptive plan (deemed the 5th key-element in Section “Key-elements essential to develop and operationalize an adaptive plan / strategy”). In the TE2100, this process was implicit and stemmed from the cycle of steps organically followed and inspired by the process provided by Willows and Connell (2003). In contrast, in the DP, this process was explicit in the ADM diagram provided to DP and Subprogrammes and adapted from the diagram of DAPP process (Haasnoot et al., 2013). This tailored diagram of ADM process served as a practical tool for communicating with decision-makers and practitioners and explaining what AP&M is about, and the ongoing, iterative nature of long-term coastal climate adaptation. Notwithstanding, the availability of the diagram of ADM process since the outset of DP (Deltares 2018‐online; Brugge and Bruggeman 2019‐online) did not mean that its prescribed steps were exactly the same conducted by DP and Subprogrammes.

### Key-elements essential to develop and operationalize an *adaptive plan/strategy*

This section identifies and systematizes the elements that were essential to design and implement a robust adaptive plan/strategies, and operationalize the Adaptive Planning and Management approach, in each studied case. Moreover, it synthesizes the main ingredients that confer robustness, flexibility, and adaptability to the general plan/strategy.

Drawing on the in-depth analysis of the TE2100 Plan and of the Delta Programme (2014), and on prior studies on these cases, through an induction process, it was possible to identify what elements of the TE2100’s Dynamic Adaptive Planning approach and of the DP’s ADM have been essential to develop and operationalize a robust adaptive plan/strategies and fulfil an AP&M approach (Table 1).

**Table 1:** Key-elements essential to develop a robust adaptive plan/strategy, and operationalize the Adaptive Planning and Management approach

Key-element	Rationale for deriving key-elements from the TE2100’s Dynamic Adaptive Planning	Rationale for deriving key-elements from the DP’s ADM
**K1: To consider and use a wide range of plausible future scenarios** (of CC, SLR, socioeconomic change) instead of a single scenario, to assess measures’ and pathways’ effectiveness	Given the deep uncertainties about the future effects of CC, SLR and storm surges on flood risk, the Team commissioned a study to produce a range of plausible future SLR scenarios for the TE until 2100. Meanwhile, it worked with an extreme scenario (*High*+*+*, 4,2 m), which set an upper boundary for the identification of possible measures and design of *HLOs*/pathways, and four socioeconomic scenarios (A to D). Later, other three SLR scenarios – *Defra 06 central* (0,9 m, *most likely*), *Medium-High* (1,5 m), *High*+(2,7 m) – and socioeconomic scenarios A and D, were used for refining the *HLOs* into *Detailed Options for appraisal* and appraising them. **1b 2b 2c**	Due to uncertain future changes, the DP and Subprogrammes used a range of four plausible futures called *Delta Scenarios* (*Busy*, *Steam*, *Rest, Warm*), to assess measures and strategies. The *Delta Scenarios* show how the climatic and socioeconomic conditions might change until 2050 and 2100, varying in terms of rapid/moderate CC and socioeconomic growth/squeeze, and present their figures for SLR, river discharges, and land subsidence. The Rest and Busy consider a 0,35 m SLR up to 2100, while the Warm and Steam consider a 0,85 m SLR until 2100. **1b1**
**K2: To identify critical thresholds**/**Adaptation Tipping-points (ATPs)**, i.e. conditions under which the current or a proposed measure no longer meets the objectives (i.e. ceases to be effective), or the current system performs unacceptably, and a new measure is needed	The Team identified critical thresholds in the sensitivity and vulnerability of the FRM system to flood risk that may occur between the present and the upper-bound figure of SLR initially used (*High*+*+*) and which would require new measures, e.g. critical levels, major change points, disruptive limits for the existing system that imply modifying defenses, SLR levels at which existing defenses fail, engineering limits (sea levels) to adapting defenses, level at which retreat will be needed. Thresholds were the starting point for planning measures and pathways (*HLOs*). Besides, the Team assigned a FRM policy to each area of the TE (specifying whether the risk of flooding would increase, be maintained or be decreased), which implied assessing if pre-existing Standards of Protection (translating the maximum acceptable flood probability/flood return period) should be increased, maintained, or reduced (e.g., 1 in 10000-year return period set for London area). To design *HLOs*, it was necessary to identify conditions under which a measure no longer meets prespecified criteria and it is necessary to either take a new measure or switch to another *HLO*. This required a threshold analysis (scenario-neutral threshold approach). Measures were assessed on their effectiveness under various plausible levels of SLR: when a measure ceased to be effective, a new measure was needed: each *HLO* is made up of sequenced measures to prevent that a certain probability of flooding or water level (threshold) is exceeded. **1c 2a 2c**	The DP defined *tipping-points* as points at which the existing system ceases to meet requirements; a *tipping-point* occurs when, due to changes in climate or socioeconomic conditions, an existing measure, policy, or infrastructure, becomes insufficient to comply with the defined criteria (due to physical, technical, or financial constraints or socially unacceptable effects) (DP Delta Programme, 2011). The analysis of ATPs sets out which and when decisions and new measures must be taken (DP Delta Programme, 2011). Subprogrammes sought to assess how much longer the current (policy and management) measures will suffice and when adjustments will be required – i.e. when the first tipping-point of the existing system/measures will be reached (DP Delta Programme, 2010; Werners et al., 2016). The main issue was analyzing for how long the current policy and management measures will suffice (be satisfactory) under a changing climate (more than determining the levels of SLR), and when the first ATP (at which such measures are no longer tenable) will be reached, and, to answer this, the *Delta Scenarios* were used (DP Delta Programme, 2010). In the DP, ATPs refer to conditions under which the current or alternative measures might fail and are associated with acceptable return periods for flood events, which were translated into design criteria for flood defenses (Jeuken et al., 2014). To develop their *paths*, some Subprogrammes analyzed at what times new measures will be required, which implied examining the moment/timing of ATPs (DP Delta Programme, 2012). **1b2 2b**
**K3: To develop a robust and flexible set of measures** (or sets of measures, each set as an Option/pathway), to deal with uncertain future changes, using APs approach. Each Option/strategy is a robust flexible set of measures, i.e. a pathway. Implies designing robust flexible strategies with APs; devising each strategy as a pathway (a set of sequenced measures)	APs (initially called Route-map/Decision Pathways approach) was devised and used as a method to build a robust adaptive plan. It involved developing several possible pathways (*HLOs/Options*) and representing them in a route-map. Each *HLO* is a package (set) of measures (individual or in *portfolios*) sequenced to manage flood risk over time. Five *HLOs* were designed. Each pathway is flexible as it is possible to move from a measure to another, and it is also possible to move from a pathway to another or change the timing of measures or measures themselves. Using APs, the Team designed a set of Options – a range of different FRM Options, where each Option is itself a set of sequenced measures, a pathway. This resulted in a set of Options that are robust and adaptable to uncertain future changes i.e., *flexible adaptable decision pathways*. Using APs, it was possible to design a robust and flexible set of measures, or, more precisely, sets of measures. APs allowed identifying and sequencing suites of measures to manage risk over time, while maintaining flexibility to deal with plausible future SLR. The Plan (its actions) is implemented iteratively over time; it keeps options open. The Plan and its Options can be adapted over time as changes or new insights arise e.g., the timing for measures, or measures, can be adjusted. **2**	DP assumed that each of its *Strategies* should be robust and flexible. The second and third principles of ADM are directly related to dynamic robustness and flexibility. The third principle explicitly implied designing adaptation paths (pathways), which should allow switching to another measure or path depending on developments. Working with *adaptation paths* was not only a principle of ADM, but also an element essential to develop each Preferential Strategy (PS) as an overall robust adaptive strategy, containing a robust flexible set (or sets) of measures. ADM recommended using the APs (as a method for exploring and sequencing a set of possible measures based on external changes over time). With APs, Subprogrammes envisioned short-term measures chained with possible long-term options and possibilities for switching between them. By using APs, each Subprogramme developed a map of *adaptation path(s)* in its PS. Such map contains one or more *paths*; each *path* (pathway) is deemed a robust and flexible set of possible measures sequenced over time. The design of robust flexible strategies (each strategy containing a robust flexible set, or sets, of measures), using the APs method, was essential to craft each PS as an adaptive strategy. **3**
**KE4: To continuously monitor changes and new information, regularly reevaluate the Plan, and review/adapt it accordingly**	TE2100’s Monitoring and Review system sets indicators to be tracked over the Plan’s lifespan, and decision-points (which trigger a decision on the measures to implement based on observation of indicators). It defines a 10-year review of the Plan and a mid-term 5-year monitoring review. Monitoring local and global changes is required to see if Options, measures, or decisions, must be taken earlier/postponed, revised, or altered. It allows adapting the Plan e.g., its Action Plan **3c 5**	DP’s *Monitoring, Analyzing and Acting system* (MAA) uses indicators to monitor changes, implemented actions’ effects, and new insights, and regularly reassesses DD, PS, Delta Plans, and measures implemented and planned - and, if necessary, adjusts them in terms of timing, scope, or design, i.e. by changing their pace or timing (anticipating/slowing down measures), or switching of measure or *path*, if changes prompt so (if a trigger warns so). MAA operates since 2017. **4b 6**
**KE5: Ongoing process of** ***Adaptive Planning and Management***, involving iterative risk management. In TE2100: *Dynamic Adaptive Planning*. In DP Delta Programme: *Adaptive Delta Planning*/*Management*	TE2100 follows an ongoing iterative process of several steps that allow adapting the Plan (its contents) over time: (1) assessing current and potential future risks and impacts, specifying thresholds; (2) identifying possible measures and developing Options (pathways) considering various plausible future scenarios, appraising Options; (3) choosing preferred Option(s); (4) implementing; (5) monitoring changes, applied measures and new information, regularly reviewing Plan, and, if needed, change its pathways, measures, or their timing (feeding back information into prior steps). Implies ongoing learning and ensures adaptability. Whole process	ADM set out its own process for developing an adaptive strategy, based on DAPP process. DP and Subprogrammes follow the ADM process. It is a continual, circular, iterative process with 6 main steps that allow adjusting elements of the general Programme and its PS. This ensures adaptiveness in the DP, in each PS, and in the planning and management process, to cope with change and uncertainty. The ongoing process of ADM – an iterative cycle of planning, managing risks, monitoring and adapting – has been essential to develop robust adaptive strategies and operationalize a real Adaptive Management. Entire process

Table 1. Key-elements of the Adaptive Planning and Management approach essential to develop and implement a robust adaptive plan/strategy in each case (derived through an induction process, namely from the analysis of the process for developing and operationalizing such plan). The steps from Figs. 7 and 8 at which these elements emerged are shadowed in grey. Source: Own elaboration based on several authors (e.g. Ranger et al. 2013, Reeder and Ranger 2011, Ramsbottom et al. 2018, Environment Agency UK 2009a, Environment Agency UK 2009b, Environment Agency UK 2012, Jeuken and Reeder 2011, Sayers et al. 2012, Bloemen et al. 2018, DP Delta Programme 2011, DP Delta Programme 2012, DP Delta Programme 2013, DP Delta Programme 2014, DP Delta Programme 2016, DP Delta Programme 2017, Jeuken and Reeder 2011, Jeuken et al. 2014, Haasnoot et al. 2013, Marchand and Ludwig 2014, Deltares 2018, Brugge and Bruggeman 2019, Gersonius et al. 2016, Haegen and Wieriks 2015, Alphen 2016, Restemeyer et al. 2017, Zandvoort et al. 2018, Zevenbergen et al. 2018). See more on each case in the Supplementary Material (Notes 3 and 4). Prior to the DP, an ‘high-end’ scenario of 1,3m SLR was considered, however, it raised public debate, hence, when the DP was initiated, it was abandoned (Jeuken and Reeder, 2011; Jeuken et al., 2014). Instead, the DP decided to use the formal Dutch climatic scenarios, which cover a large range of plausible futures but not high-end projections (these scenarios cover extreme flood events up to 1/10,000 years) (Jeuken et al., 2014; Vink et al., 2013, p.97). Although worse/more extreme scenarios were available, the DP Staff decided to work with the *Delta Scenarios* to develop the *Strategies*, and then the selected strategies were tested under more extreme scenarios (Restemeyer et al., 2017, p.930). The main climatic parameters considered in the *Delta Scenarios* were SLR, storm surges, and river discharges (Jeuken et al., 2014; Alphen, 2016; Haegen and Wieriks, 2015)

**Fig. 9:**
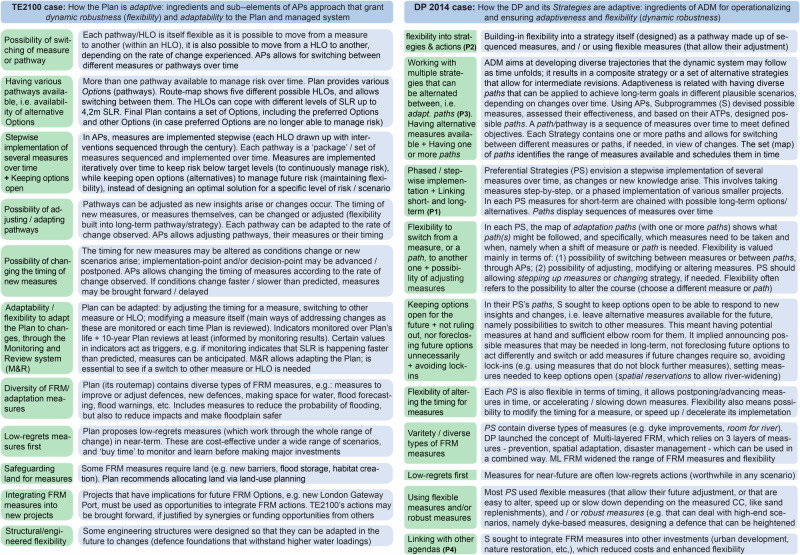
Main ways through which the TE2100 Plan (left) and the DP’s Strategies (right) are adaptive, including ingredients that make them robust and flexible, and that grant dynamic robustness and adaptiveness, namely sub-elements of Key-element 3. Some of these are patent in the ADM principles (P1, P2, P3, P4). Source: own elaboration, based on several references (e.g. Environment Agency UK 2009a, Environment Agency UK 2012, Lowe et al. 2009, Reeder and Ranger 2011, Ranger et al. 2013, Ramsbottom et al. 2018, Zandvoort et al. 2018, Jeuken et al. 2014, Jeuken and Reeder 2011, Alphen 2016, Zevenbergen et al. 2018, DP DP Delta Programme 2011, DP Delta Programme 2012, DP Delta Programme 2013, DP Delta Programme 2014, DP Delta Programme 2017, Restemeyer et al. 2017, Haegen and Wieriks 2015, Werners et al. 2016, etc.)

Figure 9 synthesizes how the TE2100 Plan (left side) and the DP’s Strategies (right side) are *adaptive*: it identifies the main ways through which the Plan/Strategies can be adapted to changes and uncertain future conditions over time, and how adaptiveness is operationalized.

#### Comparative outlook

*Regarding Key-element 1*, the TE2100 used four SLR scenarios alongside four socioeconomic scenarios, while the DP employed a matrix of four plausible future scenarios that combined rapid/moderate CC and socioeconomic growth/squeeze. The TE2100 accounted for a wider range of SLR scenarios (0,9–4,2 m SLR), including a high-end (high-impact, low-probability) scenario (4.2 m by 2100), to assess the robustness of measures, while the DP *Delta Scenarios* covered a narrower range (0.35–0.85 m SLR). The RE-D Subprogramme Team found it difficult to work with multiple scenarios: for the Team, it was too difficult to think about four possible futures, thus, they tended to focus on the worst and best *Delta Scenarios*, i.e. *Steam* and *Rest*, especially the *Steam* (Restemeyer et al., 2017).

*Regarding Key-element 2*, both TE2100 and DP2014 sought to identify thresholds or ATPs. In the TE2100, these thresholds were integral to sequencing measures and creating pathways. DP Subprogrammes faced more difficulties in specifying ATPs, particularly for variables subjected to high natural variability (e.g. river discharges), where clear policy goals do not exist, and for inherently flexible measures (Gersonius et al., 2016; Bloemen et al., 2018; Restemeyer et al., 2017). Determining ATPs for variables with large natural variability is challenging. ATPs method works better for parameters affected by gradual changes (e.g. SLR) than sudden/abrupt changes. In the DP, CC signals were hard to discern from high natural variability in river discharges, leading to the adoption of fixed values.

In the RE-D Subprogramme, experience showed that working with multiple scenarios was easier in theory than in practice. The Team found that whatever *Delta Scenario* was used, the existing defense system (without radical modifications) could cope with it, which hampered the identification of tipping-points and visualization of alternative pathways (Restemeyer et al., 2017). Thus, the RE-D Subprogramme struggled with determining ATPs and exploring alternative pathways.

Despite these difficulties, both the TE2100 and DP Delta Programme (2014) determined thresholds/ATPs, for example as acceptable flood return periods, which were translated into design criteria for FRM measures, particularly for flood defenses. In TE2100, these thresholds are patent in the flood protection standard set for each policy-unit across the TE, while in DP2014, new flood safety standards were defined based on the worst *Delta Scenario* (*Steam*) (DP Delta Programme, 2014, p.158; Bloemen et al., 2018).

*Regarding Key-element 3*, the TE2100’s route-map clearly shows five alternative pathways to manage flood risk until 2100, while the DP Delta Programme (2014) maps of adaptation paths may depict several preferred pathways (as the DD) or a single preferred path (as the PS for RE-D, which offers less options). This single path, however, aligns with the step of ADM process that involves selecting one or more preferred pathways as input for an adaptive plan (Jeuken et al., 2014).

The TE2100 route-map includes alternative pathways, allowing for adaptations if new information arises e.g., by switching of measure or pathway, adjusting the timing of measures, or reviewing measures themselves. The DP Delta Programme (2014) maps typically show fewer options, though flexibility is still safeguarded by allowing shifting between measures, linking short-term measures to long-term options, and through a staged implementation of various strategies. The PS for RE-D uses parallel, simultaneous measures (lines of action), planned for diverse geographical areas or sectors, potentially raising implementation challenges due to complex interrelated outcomes and involvement of various actors (Bloemen et al., 2018).

The comparison of the two cases highlights that it may be useful to develop two types of APs maps: a threshold-dependent map displaying various alternative pathways, and a time-dependent map illustrating preferred pathways per each scenario.

While the APs method has a strong emphasis on the temporal dimension of adaptation, the spatial scale for which the APs map is developed is also a critical aspect. The DP showed that it may be too complex to develop APs maps at regional and national scales (presenting several pathways per each area can make the map unreadable), while the local scale allows exploring more alternative pathways for a smaller area.

In the RE-D Subprogramme, the development of strategies followed a linear process from “Possible” to “Promising” strategies, and then to a “Preferred” strategy”, excluding controversial measures initially considered. The final PS focuses on improving the existing flood defense system: it is mainly based on preventive measures to reduce flood probability (gradual adjustments to defenses, e.g. dike reinforcements), and less on the integration of measures to reduce flood consequences which offer greater flexibility. Spatial adaptation measures were included but only for a few un-embanked areas. Though some flexibility was built into the system, this PS primarily involves incremental adjustments to existing measures, potentially limiting adaptability and resilience to future changes. For Restemeyer et al. (2017), the PS for RE-D reflects a key dilemma faced by the Subprogramme: balancing the aspiration for adaptability with a long-standing “urge-to-control” flood risk and nature. Adaptability was embraced but only partially: while ADM recommended identifying tipping-points and designing alternative pathways to make robust flexible strategies, this PS seems more “deterministic”, offering few possibilities to switch to alternative measures or paths.

Both the TE2100 and DP Delta Programme (2014) foresee a stepwise implementation of various measures over time, linking short-term measures to long-term options, allowing switching between different measures and/or pathways, and keeping options open, which safeguarded and enhanced the flexibility of the general plan/strategy. The TE2100 clearly identified thresholds of measures in terms of rising water levels, but also decision-points and implementation-points.

Both cases leaned heavily on improving existing hard defenses, reflecting a certain path dependency that constrains the future solution-space and flexibility. A greater variety of measures, including soft protection, nature-based solutions, and managed realignment/room-for-river measures, may be needed to expand the solution-space in the future. Yet, in both cases, the deferral of large-scale irreversible structural measures has preserved future flexibility.

Importantly, the final TE2100 Plan represents each of its pathways/options in a georeferenced map, which significantly helps to understand the spatial implications of different pathways over time.

*Regarding Key-element 4*: In AP&M, the monitoring and re-evaluation (M&R) system, including indicators and trigger-values, is a condition *sine-qua-non* to ensure learning and adaptability over the longer-term, making adaptations over time explicit since the plan’s outset (Marchau et al., 2019; Walker et al., 2001).

In the TE2100 Plan, the M&R system defines 10 indicators to be monitored and sets 10-year plan reviews and mid-term monitoring reviews every 5 years. Its first monitoring review (2016) underscored the need to distinguish CC signals from natural variability, informing adjustments to indicators.

The DP introduced its “Monitor, Analyze, Act” (MAA) system in 2017, defining indicators to be tracked, establishing systematic reviews every 6 years and a major review every 12 years, and feedback mechanisms tailored to ADM. The first 6-year review (2021/2022) revealed that: MAA outputs need to be better aligned with the needs of regional Subprogrammes, who are key to implementing PS; the initial design of MAA was too optimistic as some of its components took several years to prepare; outcome criteria needed refinement to allow evaluating PS effectively and aligning measurable and modelled criteria. The DP also highlights the challenges of distinguishing CC signals for highly variable parameters.

*Regarding Key-element 5*, implementing adaptive plans requires a planning and decision-making process that links M&E outputs to the other processual steps. An adaptive plan will ideally evolve iteratively based on predefined triggers, emerging knowledge, evolving conditions, and changing stakeholder preferences. Moreover, pathways are not set in stone, and may need periodic reassessment e.g. to align with updated acceptable risk levels (Bloemen et al., 2018). The TE2100 route-map has already evolved, with updated versions issued over the last decade (Fig. 3). In the DP, no updated maps of adaptation paths were published since DP2014 (until DP2023), although the DP’s annual reporting cycle offers potential for this. Studies involving DP Teams (after the DP Delta Programme 2014 release) found that ADM and using APs are key achievements of the DP, and that a top-three quality to be maintained in the future is adaptivity. The need for preserving DP’s adaptability during the implementation and next phases was emphasized (Bloemen et al., 2018). Inclusion of ADM process diagram into future DP annual reports could remind this.

In sum, the comparison of the two cases allowed identifying five elements essential for designing and operationalizing a robust adaptive plan. Likewise, important ingredients granting plan’s dynamic robustness and flexibility were systematized.

## Discussion

To answer the research question, it was necessary to analyze: the definition and development of the Adaptive Planning approach created in each case, and the Plan/Strategy’s development process.

**Fig. 10:**
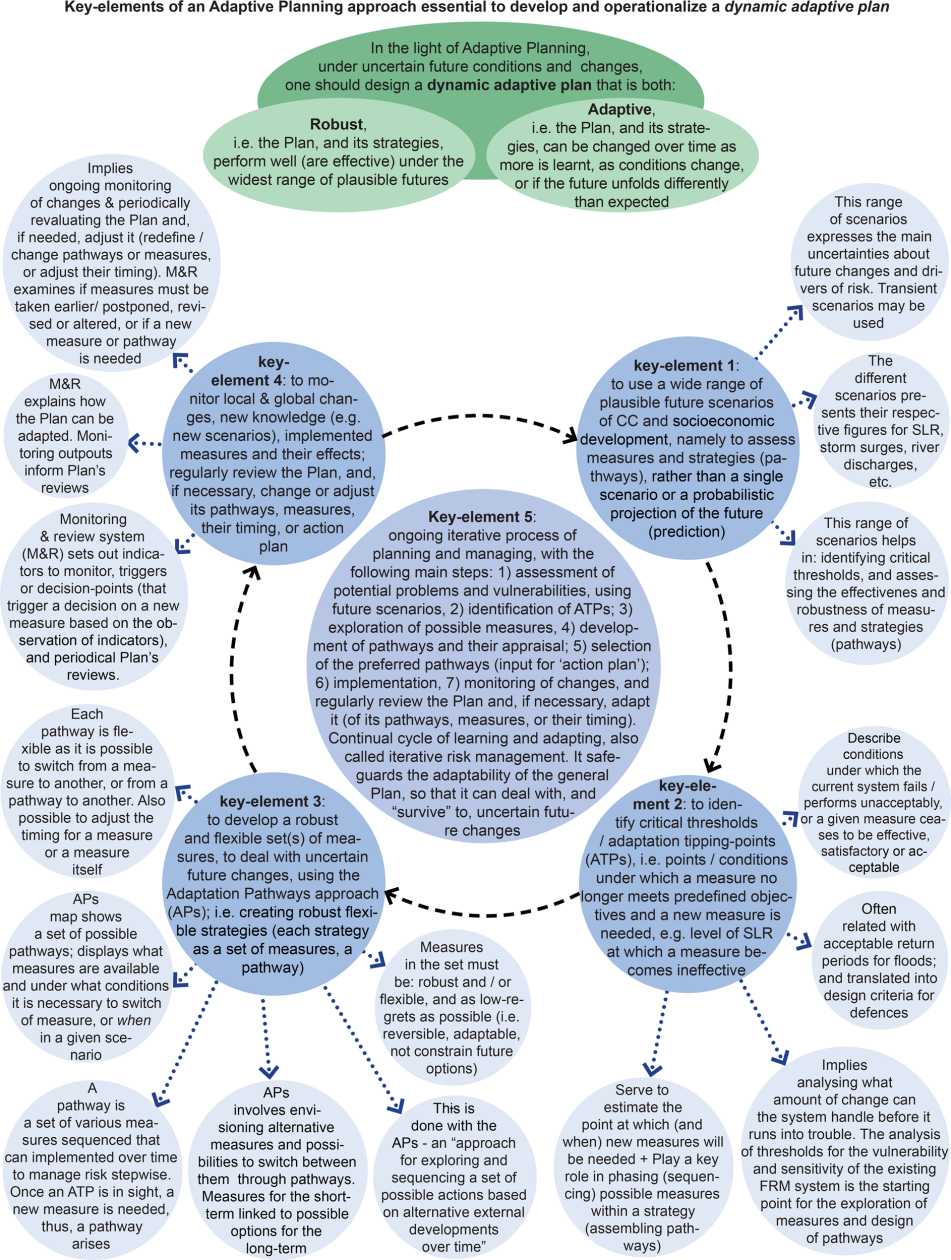
Key-elements of an AP&M approach essential for designing and implementing a dynamic adaptive plan. This figure provides a framework that can be used for analyzing and guiding the development of future adaptive plans, but also for evaluating existing cases of AP&M. Sources: own elaboration based on several references

The research then focused on identifying what elements of each case were essential to develop a robust adaptive plan and fulfil an approach of Adaptive Planning and Management. In these cases, five key-elements were identified (Fig. 10): (1) the use of a wide range of plausible future scenarios, including SLR scenarios, to assess measures and pathways on their effectiveness and robustness; (2) the identification of thresholds or tipping-points; (3) the development of robust and flexible sets of measures to deal with uncertain changes, using APs approach (each pathway is itself a robust flexible set of various measures sequenced over time), i.e. designing robust adaptable Options as pathways in the TE2100, and designing a robust flexible strategy with one or more adaptation paths in each Preferential Strategy and Delta Decision of the DP Delta Programme (2014); (4) the establishment of a monitoring and review system setting indicators and decision-points or triggers, and regular Plan’s reviews, and explaining when and how the Plan/Strategy may be adapted; (5) the ongoing iterative process to adapt the system and the Plan to, and manage, changing risk over time.

**Fig. 11:**
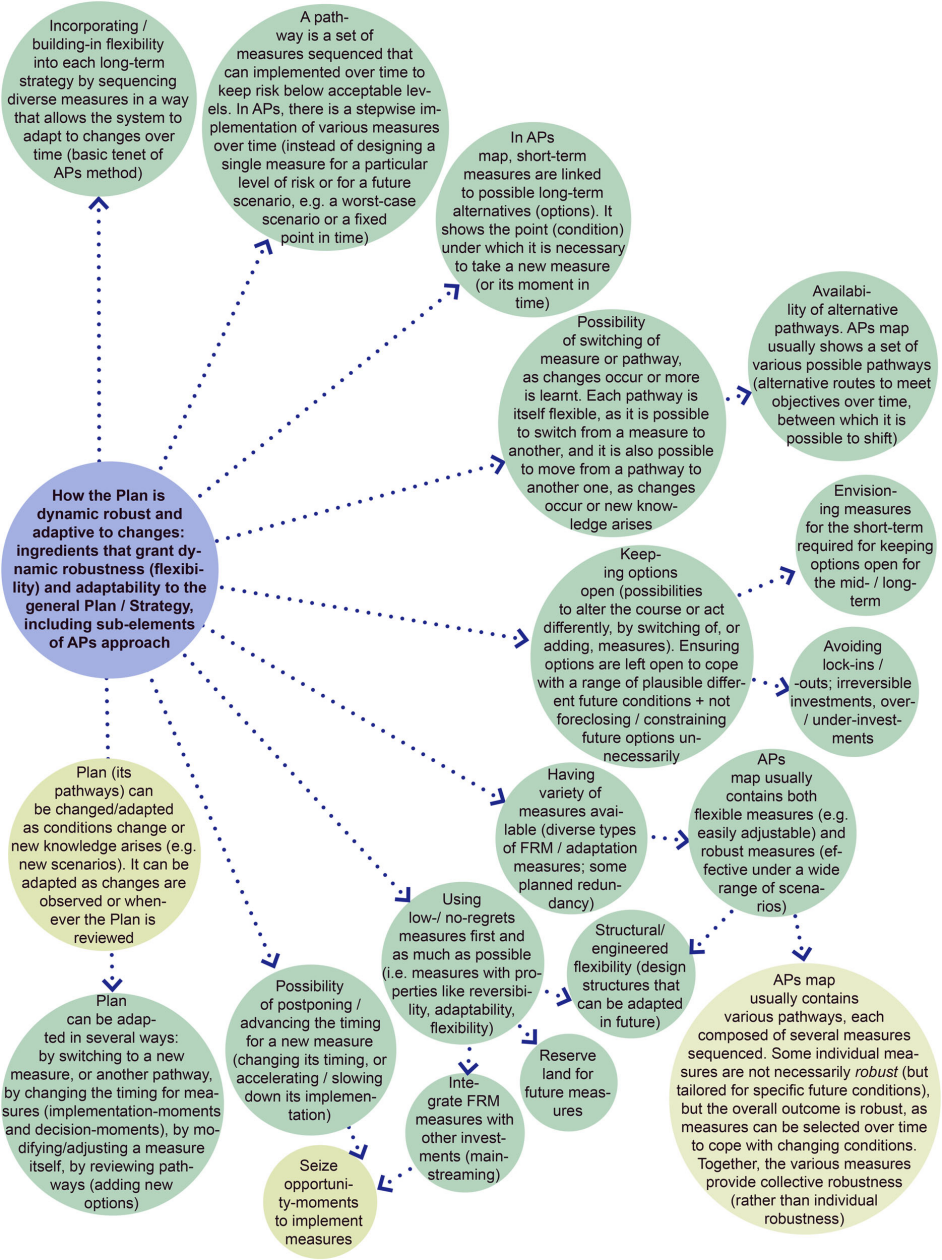
Sub-elements of the APs approach that deliver and ensure dynamic robustness (flexibility) and enhance the adaptability of the general Plan/Strategy. Source: own elaboration based on several references

**Fig. 12:**
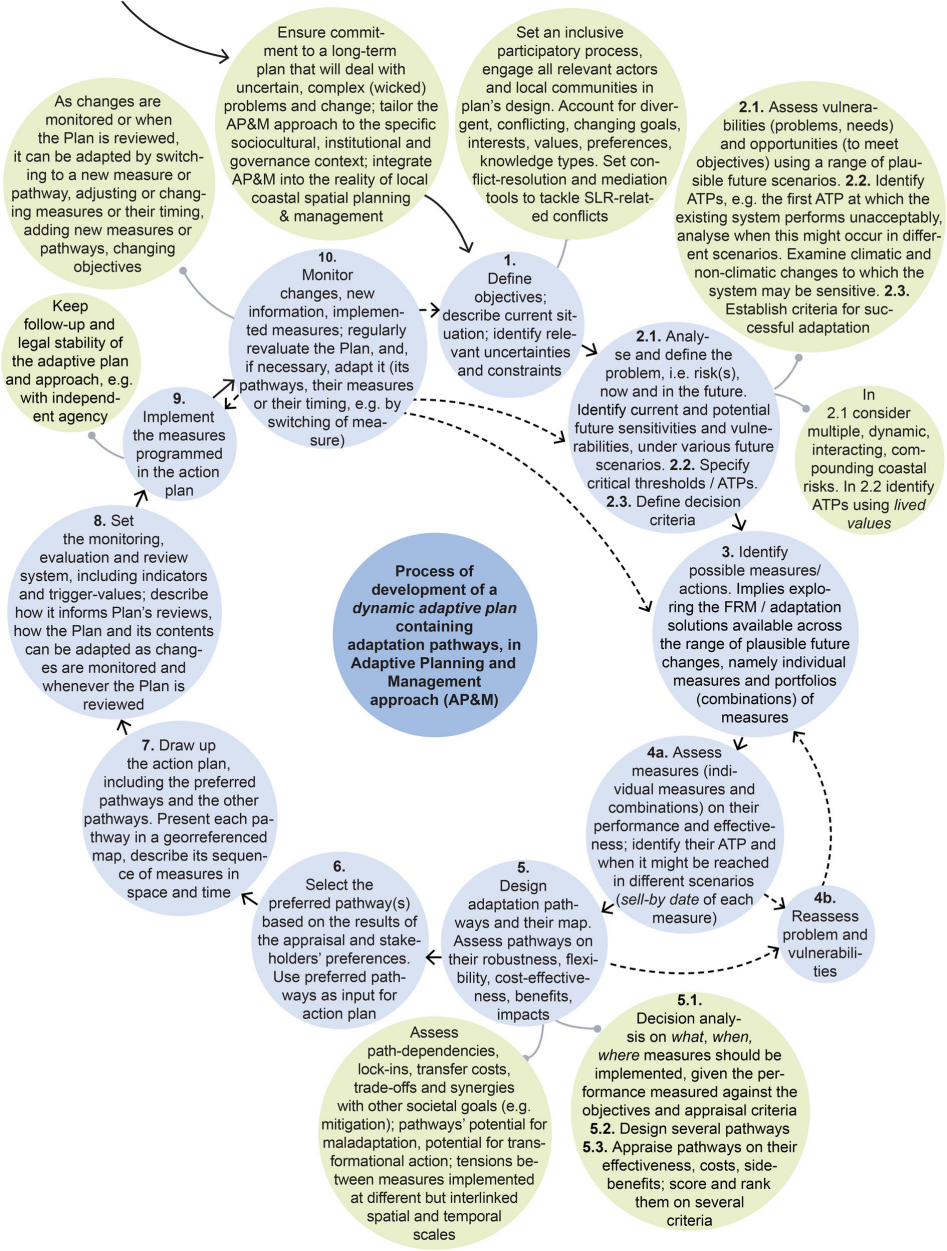
Possible process to develop an adaptive plan containing APs, using an AP&M approach for coastal adaptation. The diagram shows the main steps of a type-process (derived drawing on the two studied cases, and on the literature review on AP&M/DMDU) which might be followed to develop and operationalize a robust adaptive plan. Blue circles indicate the main steps, while the green circles relate to issues that have been raised more recently, as key challenges for developing APs-based coastal adaptive plans. Source: own elaboration based on several references

The analysis also allowed systematizing ingredients that make the Plan/Strategies adaptive, including sub-elements of Key-element 3 that grant robustness, flexibility, and adaptability (Fig. 11), and deriving a type-process of AP&M (Fig. 12).

Despite the proliferation of scientific papers exploring the APs method and its potential (Lawrence et al., 2019a), there are still few examples of development and implementation of APs-based plans beyond the three prominent well-documented examples of the TE2100, DP, and the New York City case. There is a need to continue developing, experimenting, critiquing and examining how pathways approaches can be utilized in informing and facilitating adaptation planning (Kingsborough et al., 2017).

Many studies on the use of DMDU/AP&M approaches for managing climate risks are theoretical, and it is difficult to find analyses of the value of such methods for substantiating final decisions and plans for climate risk management/adaptation (New et al., 2022). There is a need to demonstrate the value of APs-based approaches to practitioners and decision-makers (Haasnoot et al., 2019b), particularly in coastal planning and management. Much of the work that has been undertaken on APs concerns well-resourced, large-scale plans/projects, but science has demonstrated that this approach can also be useful in smaller, resource-constrained contexts, and that it can be tailored to local contexts (Haasnoot et al., 2019b; Barnett et al., 2013; Butler et al., 2016). More applications of APs-based approaches into local planning practices are required (Haasnoot et al., 2019b). Guidance on applying APs in more complex contexts marked by ambiguous or conflicting goals, values, interests, distributed responsibilities, and diverse knowledge types, is still quite scarce (Wise et al., 2014; Bosomworth and Gaillard, 2019; Haasnoot et al., 2019b).

The resources required in APs-based DMDU approaches, namely for applying modelling tools, often make them too complex and expensive, which calls for the development of “lite” versions of these approaches (Marchau et al., 2019).

To be effectively adopted, APs-based DMDU/AP&M approaches will need to be embedded into local coastal governance processes (Oppenheimer et al., 2019; Portner et al., 2022). Addressing this need requires enhanced cooperation between SLR science, DMDU/AP&M science, and planning/governance science.

Further work is needed for applying APs-based approaches into coastal spatial planning practices and instruments. These have traditionally adopted a static view of current and future risk, operating within legal systems that value certainty and stability. It is necessary to overcome the paradox certainty/uncertainty and stability/adaptability and tackle the inappropriateness of current planning practices when uncertain future changes and dynamic risks are involved (Lawrence et al., 2019a). This requires mobilizing planners’ and decision-makers’ interest in these approaches with a convincing narrative, especially at the beginning of long-term plans/projects. APs maps can be powerful illustrations of an adaptive approach (Marchau et al., 2019).

Figures 10–12 build on the analysis of the empirical cases, but also on the review of the existing literature on AP&M/DMDU approaches for coastal adaptation purposes, to provide a framework of the essential elements to design and implement a robust adaptive plan. This framework offers both an analytical and evaluation tool. It is now recognized the need for guidance on the methodological means, basic elements and tools of DMDU/AP&M to support real-world planning and decision-making around complex, deeply uncertain problems and change, especially changing coastal risks. The diagrams of the key-elements that make a plan robust and adaptive, of the ingredients that ensure dynamic robustness and adaptability, and of a type-process of AP&M, might be used as input for a future guidebook to support the implementation of AP&M approaches in coastal planning and management. Such guide could contribute to bridge the gap between science and practice on DMDU/AP&M. The authors have themselves used the diagrams of Figs. 10–12 to evaluate recent cases of introduction of an AP&M approach into coastal management plans in Portugal, proving the usefulness of these diagrams. We recommend applying this framework in future ex-ante analyses of plans that intend to follow an AP&M approach, but also in ex-post analyses of plans that have attempted to carry out an AP&M model. Practitioners may use this framework to devise their own context-specific AP&M approach, while scholars and researchers may use it to carry a more in-depth evaluation of AP&M applications, allowing detecting differences, similarities, and nuances among distinct cases.

## Conclusions

This work focused on the two major examples of application of Adaptive Planning approaches into planning instruments with coastal adaptation purposes – TE2100 Project and DP Delta Programme (2014) – to analyze, in each case, what key-elements were essential to develop and operationalize an adaptive plan.

These cases created their own methodological approaches of AP&M to build up a robust adaptive plan/strategies. In both, the devised approach contained APs method, which served to explore available measures and different possible pathways.

From these cases, it was possible to systematize five key-elements of the AP&M approach essential to design and implement an adaptive plan. The analysis also allowed reconstructing how a type-process of Adaptive Planning and Management might unfold, addressing the issue of how adaptive plans are being developed.

Important issues for further research on AP&M approaches containing the APs method could also be raised. There are still few applications of these approaches in real coastal plans. Coastal climate adaptation is a long-term ongoing process, and these approaches can be valuable to grasp how such process should unfold. The APs seem promising to guide coastal adaptation planning through manageable sequences of actions over time. However, further efforts are needed to apply existing knowledge on AP&M into the practice of coastal adaptation/risk management. As these approaches are increasingly advocated in scientific literature for supporting coastal adaptation, it is important to examine whether coastal managers and planners are using them and how these have been used for developing adaptive plans.

Given the uncertain future climatic and physical changes in inherently dynamic coastal environments, and changing coastal risks, it is necessary to design adaptive plans with route-maps of coastal adaptation pathways that address the short-term and long-term, and near and far territorial implications. These temporal and spatial scales, often conflictive, require careful consideration. Moreover, coastal APs should address multiple, interacting and compounding coastal risks.

It is also necessary to investigate to what extent different cases worldwide claiming to use an adaptive coastal planning and management are pursuing a real AP&M approach containing APs method, how such process is unfolding, and whether and how these cases meet the key-elements essential to develop and implement an *adaptive plan*. The authors have investigated these issues regarding two recent Portuguese coastal management plans which seek to adopt a new model of Adaptive Coastal Planning and Management.

The identified key-elements are flexible enough to receive additional inputs that might contribute to increase the dynamic robustness and adaptability of the plan or planning approach, and which might stem from its adjustment to diverse contexts. Notwithstanding, these elements seem to be keystones to ensure an adaptive plan, and vital “organs” of Adaptive Planning and Management approaches. A wider exploration of other cases of application of these approaches into coastal planning instruments is required to deepen this debate, grounded on empirical evidence.

## Supplementary information

Supplementary Material

## Data Availability

No datasets were generated or analysed during the current study.

## Footnotes

1 Abbreviations: (DMDU) Decision-making under Deep Uncertainty approaches; (AP&M) Adaptive Planning and Management approaches that use the Adaptation Pathways method; (APs) Adaptation Pathways approach, also called Route-map or Decision Pathways approach; (CC) climate change; (SLR) sea-level rise; (TE) Thames Estuary; (TE2100) Thames Estuary 2100; (TB) Thames Barrier; (HLO) *High-Level Option*; (DP) Delta Programme; (ADM) Adaptive Delta Management; (ATP) Adaptation Tipping-Points; (PS) Preferential Strategies; (DD) Delta Decisions; (RE-D) Rhine Estuary-Drechtsteden Subprogramme; (FRM) Flood Risk Management; (FS) freshwater supply. Adaptive Planning and Management (AP&M) or Decision-Making under Deep Uncertainty (DMDU) approaches are often given different names, like *Planning and Decision-making under Uncertainty* (Haasnoot et al., 2013), *Decision-oriented approaches* (Ranger et al., 2013; Wise et al., 2014), *Planning approaches for Adaptation under Deep Uncertainty* or *Approaches for designing a sustainable adaptive plan* (Walker et al., 2013), *Dynamic Robustness-based approaches*, and *Adaptive Planning approaches* (Haasnoot et al., 2013, p.485, 496; Walker et al., 2013; Kwakkel et al., 2015, p.374; Ranger et al., 2013).

2 Moreover, scarce work has been done to evaluate the current use of APs and its utility to planners, policymakers and decision-makers (Lin et al., 2017; Bloemen et al., 2018). Further analysis is needed to understand how adaptive plans have been developed and operationalized (Walker et al., 2013; Restemeyer et al., 2017).

3 In addition to these three main typologies of studies specifically targeted at adaptation/risk management in coastal contexts (including estuarine and deltaic regions), there is another type of studies which focuses on further developing, and discussing (critically reflecting on) DMDU/AP&M approaches within the broader fields of climate adaptation, environmental management, or sustainable development (Wise et al., 2014; Butler et al., 2016; Maier et al., 2016; Bosomworth et al., 2017; Burnham et al., 2018; Marchau et al., 2019; Magnan et al., 2020; Werners et al., 2021; Sparkes et al., 2023; Dittrich et al., 2016; Schlumberger et al., 2022).

4 Other applications for coastal risk management purposes in ‘real’ plans/policy-programs include, for example, the New York City case (Rosenzweig et al., 2011; Rosenzweig and Solecki, 2014; Yohe and Leichenko, 2010), the case of Eyre Peninsula in Australia (Siebentritt et al., 2014); and, more recently, the case of the *National Adaptation Plan of Bangladesh 2023-2050* (MEFCC, 2022) and *Bangladesh Delta Plan* (Alphen et al., 2021; GPRB, 2017; https://cdkn.org/story/feature-bangladeshs-delta-plan-offers-major-opportunity-for-climate-compatible-development?loclang=en_gb), the case of Hawkes Bay, New Zealand (Lawrence et al., 2019a, b, c; Ryan et al., 2021; https://www.hbcoast.co.nz/strategy-development/), the case of Los Angeles (Aerts et al., 2018a); the case of Miami (Roscoe et al. 2019); the case of Queensland QCoast2100 program in Australia and its related *Coastal Hazard Adaptation Strategies* (LGAQ, 2016; https://www.qcoast2100.com.au/), the Coastal Adaptation Pathways (CAPs) program in Australia (Lin et al., 2017); the case of the State of California, USA (SOC - State of California, 2018; SOC - State of California, 2024; CCC, 2018), the case of the City of Santa Cruz, California (CSC, 2019, 2020; SCCC, 2020), and the case of North Shore, Hawaii (NSCRWG, 2022).

5 *Dynamic Robustness* was recommended by UK Government for domains marked by *unquantifiable uncertainty*, which demanded a *flexible adaptive planning approach*. The TE2100 placed *Dynamic Robustness* at the heart of its planning approach, recognizing the need for robust methods to elaborate a long-term plan robust to uncertainty about CC and other changes (Ranger et al., 2013, p.248; Environment Agency, 2009b, p.199).

6 This Dynamic Adaptive Planning approach contained the APs (Decision Pathways / Route-map) approach as a sub-method (Reeder and Ranger, 2011, p.2-3), and it was largely based on the “Risk, Uncertainty and Decision-Making Technical Report” produced for UKCIP (Willows et al., 2003) and other tools and emerging guidance, and prior work of the EA’s collaboration in the ‘European Spatial Planning Adapting to Climate Events Project (ESPACE) where partners from UK, Holland, Germany worked on transnational methods (Environment Agency, 2009b, p.3, 200; 2009a, p.71; Ranger et al., 2013; Reeder and Ranger, 2011; Lowe et al., 2009). From the outset, through the ESPACE partnership and advancements in the TE2100, the Team developed techniques for “scenario-neutral analysis” which involved identifying critical thresholds in the FRM system.

7 Three nation-wide (FRM, FS, urban development and restructuring) and six regional (Rhine Estuary-Drechtsteden; Southwest Delta; Ijsselmeer Region; Rivers; Coast; Wadden Region). The DP is institutionally substantiated in five Ds: *Delta Act*, *Delta Fund, Delta Commissioner*, *DP Report*, and *Delta Decisions* (five decisions, prepared between 2010 and 2014, issued in the DP Delta Programme, 2014) (Restemeyer et al., 2017; Werners et al., 2016).

8 In 2010, the DP Commissioner underlined that “dealing with uncertainties in the future climate, but also in population, economy and society (…) requires a new way of planning, which we call adaptive delta planning”, which “seeks to maximize flexibility; keeping options open and avoiding ‘lock-in’” (DP Commissioner Kuijken’s speech at ‘Deltas in Times of Climate Change conference’, Rotterdam 2010, *in* Haasnoot et al. (2013) p.490). This *Adaptive Delta Planning* resembles the DAPP; the DP offered a study-case to test its applicability (Haasnoot et al., 2013).

9 To implement ADM, the national DP Staff provided to regional Subprogrammes an ADM Implementation Guide (Rhee, 2012) and personnel support. Subprogrammes could also hire two external consultants from private companies to assist in applying ADM, one of whom helped write the implementation guide. While the national staff encouraged and supported ADM in the Subprogrammes, it did not impose (dictate) anything (Restemeyer et al., 2017, p.929; Werners et al., 2016; Zandvoort et al., 2018).

10 To ensure a “sustainable and robust FRM and FS” and cope with climatic extremes, the DP’s strategies should be “prepared for various scenarios” and provide flexibility to respond to new insights and changing conditions either by “stepping up measures” or “changing of strategy” if necessary (DP Delta Programme, 2014, p.6; DP Delta Programme (2013), p.6). The ultimate goal is that the FRM system meets the requirements “at all times”, which requires solution “able to adapt to new insights and circumstances” and “sufficient options to remain open in the future to take the required measures” (DP Delta Programme, 2013, p.102). The Subprogrammes searched for short-term measures that are adequate in the long-term and enhance system’s robustness, making it suited for diverse scenarios (DP Delta Programme, 2010). The DP aims to make the country ‘robust’ to withstand greater climatic extremes, thus “prepared for various scenarios”; and strategies should provide flexibility to respond to change, by “stepping up measures” or “changing strategy” if necessary (DP Delta Programme, 2014, p.6). A *robust system* is “*one that can withstand extreme events and accommodates different future developments*”, and a *flexible system* is “*one that can easily adapt or be adapted to changing circumstances*”, e.g. ‘room for river’ increases robustness, while sand replenishment is a measure that maintains flexibility (DP Delta Programme, 2010, p.69). Strategies should ensure ‘*flexibility’* (also called *‘adaptability’*) and ‘*robustness*’ (also called *resistance to extreme events*) (DP Delta Programme, 2010, p.4).

11 The ADM Guide explains that, to make strategies flexible, it is necessary to know “when to take action or change course” (ahead of when an ATP is reached); in addition, “thinking about the first decision, and potential follow-up decisions in the long-run” is required to prevent lock-in/-outs (Rhee 2012, *in* Restemeyer et al. 2017, p.931, 933). Overall, ADM starts by questioning how long current strategies will be effective under diverse scenarios, which leads to the exploration of alternative measures and design of multiple pathways (rather than a single solution for a ‘business-as-usual’ or worst scenario). The basic idea underlying the APs in ADM is “to generate a wide array of pathways (each pathway consisting of a series of measures) through which the policy-objectives are achieved under changing climate and socioeconomic conditions” (Deltares, 2018, p.4). The set of pathways in a map provides a range of options that may be used in future; thus, the APs approach helps to enhance the plan’s and the system’s flexibility and adaptability (capacity to adapt to multiple plausible futures and changes) (Deltares, 2018, p.2-4). In the DP, strategy-making built on an important element to develop strategies – the construction of pathways – which implied assessing the flexibility of measures and paths and scanning options to keep open – both aspects patent in DP Strategies; the outcome of ADM was a single pathway or a set of pathways, with which it was possible to schedule measures despite uncertain future change (Rhee 2012, *in* Zandvoort et al. 2018).
