# Supplementary material for: Adaptive Planning Approaches for Coastal Climate Adaptation: Process and Key-elements
Source: Environ Manage. 2025 Jan 21;75(4):1013–38. doi: 10.1007/s00267-025-02117-1 (PMC11965211; doi:10.1007/s00267-025-02117-1)
Supplement: Supplementary file 1 — Supplementary Material [file 267_2025_2117_MOESM1_ESM.docx]

# Supplementary Material


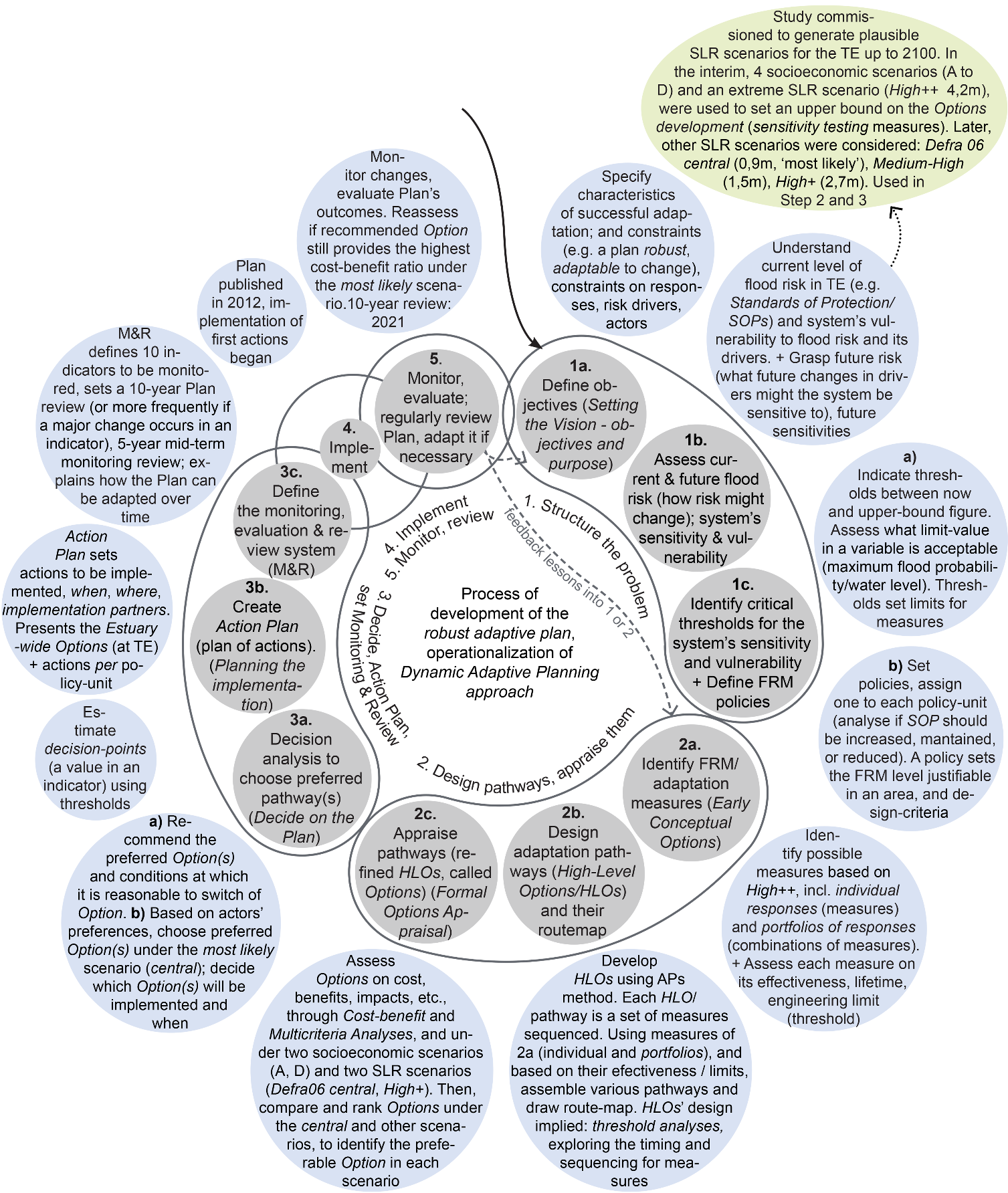


Figure: TE2100 process with a medium level of detail


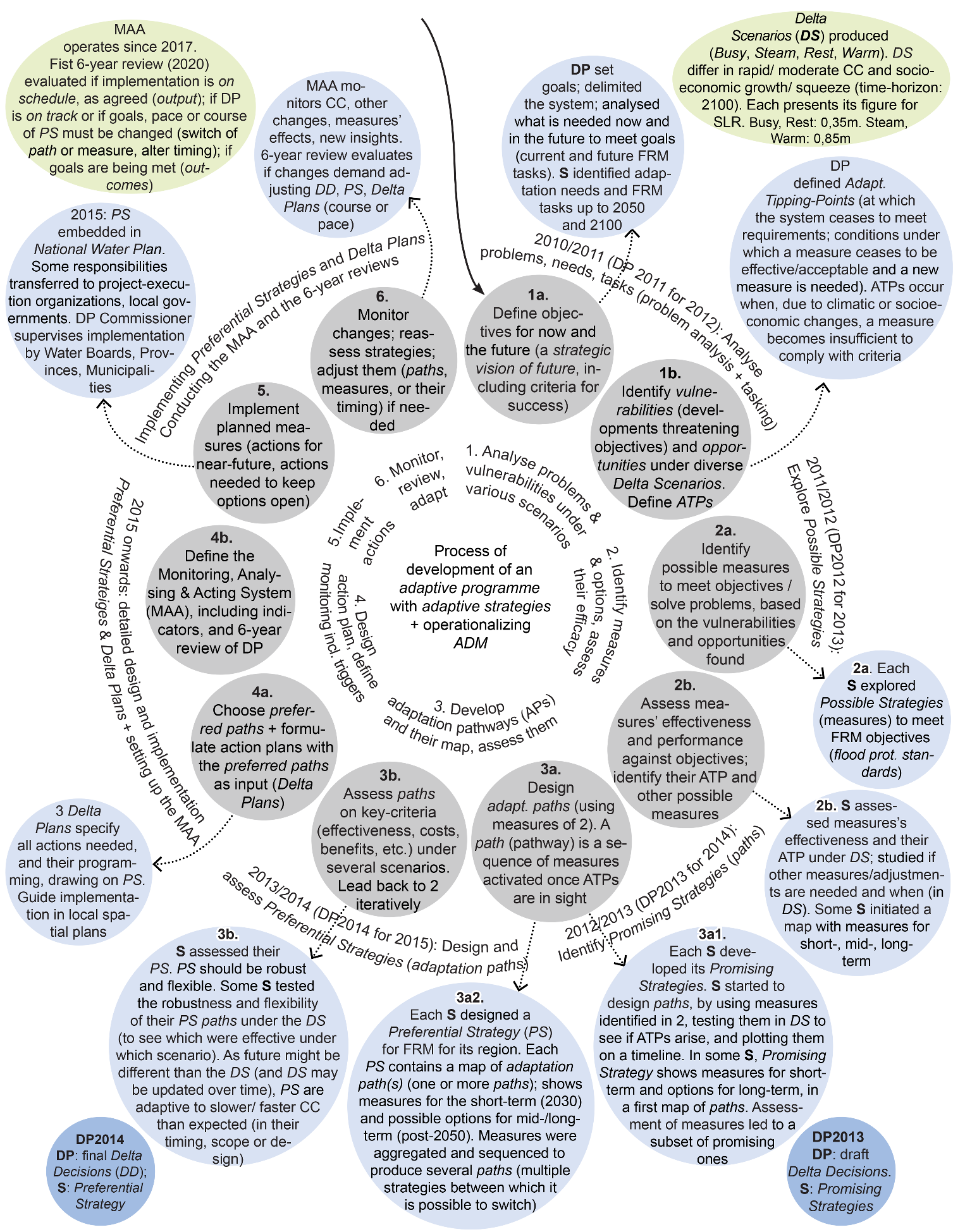


Figure: DP process with a medium level of detail

# NOTE 1. How the TE2100 has developed and applied its *Dynamic Adaptive Planning approach*: process of steps

This section describes how the *Dynamic Adaptive Planning approach* was applied in the TE2100 Project to develop an *adaptive FRM plan*, focusing particularly on how the APs method was used to design the pathways (how the pathways and route-map were designed). The TE2100 followed a *decision-centred* *planning process* (also called *policy-first*, *context-first* or *bottom-up process*) (Ranger et al. 2013; Reeder and Ranger 2011; Ranger et al. 2010).[[1]](#footnote-1) The TE2100 Plan itself (EA 2012; EA 2009b), and several authors and documents have provided a detailed description of the creation and application of such Adaptive Planning approach (including the APs’ method within it) and of the development of a robust adaptive plan using it (see e.g. Reeder and Ranger 2011, Ranger et al. 2013; Penning-Rowsell et al. 2013; Lowe et al. 2009; HM Treasury 2009; London Councils’ TEC 2007; London Councils 2018; EA 2016; Ramsbottom and Sheppard 2017[[2]](#footnote-2); Bloemen et al. 2018). **Figure 1** provides a detailed depiction of such process, and it builds on the reconstruction of the process described in **Table 1**.


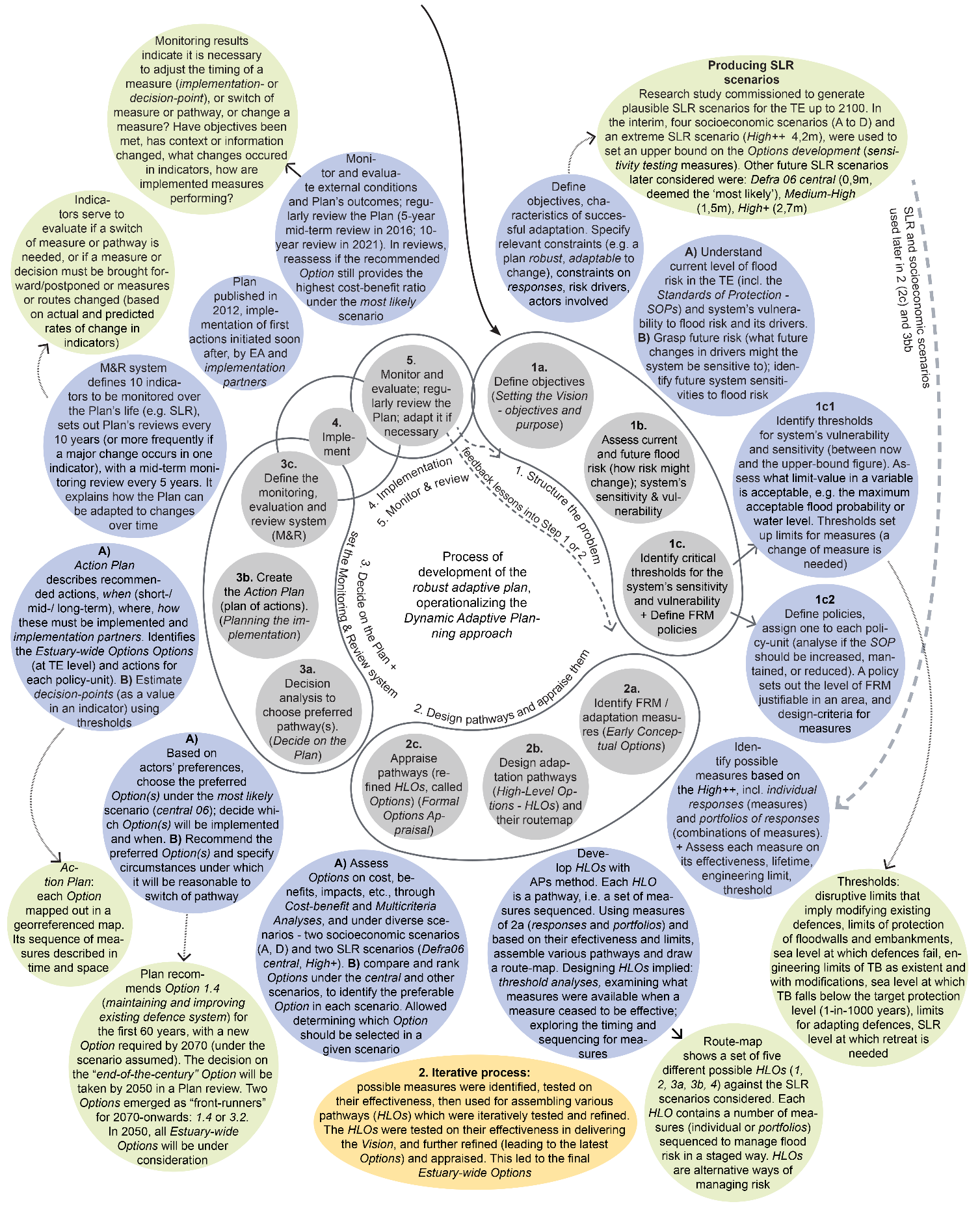


**Fig. 1** Planning process of the TE2100, showing the various steps of development of the Plan. Source: own elaboration, based on EA 2012, 2009a, 2009b; Ranger et al. 2013, 2010; Reeder and Ranger 2011; Ramsbottom and Sheppard 2017; Lowe et al. 2009; HM Treasury 2009; Penning-Rowsell et al. 2013; London Councils 2007, 2018; etc.

| **Table 1. Planning process of the TE2100 and its main steps and sub-steps** | | |
| --- | --- | --- |
| Structure the Problem | 1a. Defining objectives and relevant constraints  *Understanding main characteristics of the decision problem i.e. objectives, stakeholders’ values, constraint, decision criteria* | - Defining the objectives of the TE2100 (*Setting the Vision – objectives & purpose*, *in* EA 2012, p.32; EA 2009A, p.vii, 7, 8, 65-66, 266)   The main objective of the Project was to develop a FRM plan to manage tidal flood risk in the TE. Specific objectives were, e.g. ensuring sustainable urban development in the floodplain, analysing when the existing FRM system might need modifications and produce a *forward plan* up to 2100 (EA 2012, p.32; EA 2009A, p.vii, 7; Ranger et al. 2013, p.239; Ramsbottom and Sheppard 2017, p.). |
|  |  | - Specifying constraints and decision criteria   This implied identifying constraints on the responses. The Plan must be justifiable in terms of cost-benefit (*value for money*), and against policy, coordinate diverse stakeholders’ interests and trade-offs, meet social, economic and environmental objectives, and account for multiple uncertainties, namely about the rate of plausible future increases in the water level, climate projections, and valuation of non-monetary impacts. It was decided that the Plan must be ‘*as robust as possible*’, ‘*adaptable to change and remain fit for purpose throughout its 100-year lifetime*’ (EA 2012, p.36, 38, 27, 29, 35; Ranger et al. 2013, p.239, 240-242; Bloemen et al. 2018, p.8).  This step also implied identifying the main drivers of changing flood risk in the TE (CC, urban development in the floodplain, increasing population, growing economic value at risk, ageing flood defences) (Ranger et al. 2013, p.242; Marchand and Ludwig 2014, p.13; LC TEC 2007; EA 2009A, p.74-84; Bloemen et al. 2018, p.8). |
|  | 1b. Understanding and assessing flood risk, and analysing the vulnerability of the system  (flood risk assessment + vulnerability analysis)  *Understanding flood risk and the TE* (studying flood risk and *how it might change in the future*) (EA 2012, p.32) | *Understanding flood risk and the TE*  The Team studied current flood risk and *how it might change in the future* (EA 2012, p.32), i.e understand and assess current flood risk and future flood risk (EA 2009A, p.8, 23, 70). A comprehensive flood risk assessment was conducted, it involved an in-depth analysis of the existing flood defence system (type, dimension, crest level of defences), areas at risk of flooding, TE’s natural processes and habitats, and inspections to defences from the TB to the sea, and collection of data on sediments (EA 2012, p.32; London Councils’ TEC 2007; Ramsbottom and Sheppard 2017, p.5,16; EA 2009A, p.vii). |
|  |  | - 1b1) Assessing current flood risk and vulnerabilities of the system   A detailed assessment of the current level of flood risk was carried, which involved analysing the existent *Standards of Protection* (*SOPs*) in the TE and current vulnerability of the defence system (Ranger et al. 2013, p.242; Reeder and Ranger 2011, p.5; Bloemen et al. 2018, p.7; EA 2009A, p.23, 33-54). Firstly, flood risk was assessed without existing defences (to examine its magnitude and the importance of defences), and then with defences. To calculate current risk, the Team has estimated the *current flood probability* and analysed existent ‘*design* SoPs’ (expressed as an *annual flood probability* and available in previous studies or projects) (Ramsbottom and Sheppard 2017, p.8, 10). |
|  |  | - 1b2) Assessing future vulnerabilities and identifying sensitivities of the system; understanding how flood risk might change in the future   The Team sought to assess and map potential future vulnerabilities and identify sensitivities of the defence system to plausible futures increases in the water level, and to CC-related risks (Ranger et al. 2013, p.242; Reeder and Ranger 2011, p.6; Bloemen et al. 2018, p.7). This implied analysing how flood risk might change in the future due to CC effects, socioeconomic development, and ageing defences (EA 2012, p.32, 25; Ranger et al. 2013, p.239). Thus, at this stage, the Project Team commissioned research work to better understand the effects of CC on relative SLR, storm surges and river flows, and quantify the plausible future increases in water levels in the TE, and generate a range of plausible scenarios of water level rise up to 2100 (Ranger et al. 2013, p.246; Reeder and Ranger 2011, p.7; Ramsbottom and Sheppard 2017, p.7; EA 2012, p.28; EA 2009b, p.2, 5; Lowe et al. 2009, p.88; EA 2009A, p.71). This research work proceeded in parallel with the development of *Options* in the TE2100 (as a ‘second track’ to the planning process) (Ranger et al. 2013, p.246; Reeder and Ranger 2011, p.7; Ramsbottom and Sheppard 2017, p.7; EA 2012, p.28; EA 2009b, p.2, 5; Lowe et al. 2009, p.88).[[3]](#footnote-3) In the meantime, to address future flood risk and its plausible increase, the Team developed and used an extreme / high SLR scenario – the *High++* (*first guess*) (4,2m by 2100) (based on expert judgement, it captured plausible ‘worst-case’ estimates of the influence of physical processes) – which set an *upper bound* on the *Options development* (Ranger et al. 2013, p.246, 242; Reeder and Ranger 2011, p.7, 6). This *High++* (first guess) was then used for *sensitivity testing* the robustness of adaptation / FRM measures to uncertainties (this value was used in the initial identification of measures and design of Options) (Ranger et al. 2013, p.246, 247; Reeder and Ranger 2011, p.7).[[4]](#footnote-4) In addition, four socioeconomic scenarios were developed (based on expert elicitation), which also set an upper-bound for the *Options* development (Ranger et al. 2013, p.242; Penning-Rowsell et al. 2013, p.1401; Jeuken et al. 2014, p.13; EA 2009A, p.11, 83-84).  The commissioned research work was scheduled to finish by the end of the TE2100 Project timeline, therefore, in the interim, the Project devised / developed a set of scenarios of water level rise up to 2100 *to use in the interim to develop the Options* (EA 2009b, p.2, 7; Lowe et al. 2009, p.88; HM Treasury 2009, p.22; EA 2009A, p.229; Ramsbottom and Sheppard 2017, p.7-8): *High++* (4,2m, *first guess*); *Medium-High* (1,5m rise ); *High+* (2,7m rise); and *Defra06 central* (0,9m rise) (EA 2009b, p.2, 7; Lowe et al. 2009, p.88-89; EA 2012, p.28; HM Treasury 2009, p.22; Ranger et al. 2013, p.247, 246, 242; Reeder and Ranger 2011, p.7, 6; Ramsbottom and Sheppard 2017, p.8; EA 2009A, p.229, 72-73).  These were used (this set of scenarios was used) in the Early Conceptual *Option* (ECO) and subsequent *High-Level Option* development (*Phase 2*) and in the first part of *Phase 3 - Option Development* (e.g. four scenarios were used for testing the *Early Conceptual Options* –*Defra 2003* (around 0,60m by 2100), *Medium-High*, *High+* and *High++*) (EA 2009b, p.2, 188,195).  Later, the results of the commissioned research work showed that the Project had been planning for the right potential range of water levels over this century, and that the previous *worst-case scenario* could be revised down from 4,2m to 2,7 m (Lowe et al. 2009, p.89; EA 2012, p.28); these results also served to identify which scenario is the most probable to allow the final Options to be refined (redeveloped under such scenario) (EA 2009b, p.2). Further TE2100 modelling work (done in collaboration with UKCIP) later confirmed the levels suggested by the *central* *scenario*, and showed that the “worst-case situation’ is more likely to be nearer the *High+* than the *High++* (HM Treasury 2009, p.22).  Overall, the TE2100 studies involved the estimation of future extreme water levels in the TE, which range from a 0,94m rise (which is based on *2009 Government guidance*) up to a 2,7m rise until 2100 (EA 2009A, p.72, 229). |
|  | 1c. Identifying thresholds in the system’s sensitivity and vulnerability; & define FRM policies and assign one to different areas | - 1c1) Identification of key thresholds in terms of system’s sensitivity and vulnerability   The Team identified *critical thresholds* for the sensitivity and vulnerability of the system to flood risk, i.e. limits that would be disruptive for the FRM system, and which may occur between the present and the upper-bound figure of SLR considered (*High++, first guess*), and which might imply modifying existing defences, namely:   - limits (of protection) of the existing flood defence system (floodwalls and embankments), e.g. the level of SLR at which the existing defences would fail. - engineering limits of the TB as existent and with modifications. - the SLR level at which the TB (as designed) falls below the target protection level (1 in 1000 years). - the ‘limit of adaptation’ of the defence system, e.g. the level of SRL at which it will be quite difficult to continue to protect London in its current form and some retreat will be needed. This level represented a limit to continuing with upgrades and additions to the existing defences. It was estimated to be a 5m SLR (Ranger et al. 2013, p. 241, 242, 255, Reeder and Ranger 2011, p.6; Bloemen et al. 2018, p.7, 8; Ramsbottom and Sheppard 2017, p.4; EA 2009A, p.149-151).   The Team focused on examining ‘*what change can the system handle before it runs into trouble’*, particularly what increase in SLR and peak river discharge could cause a technical failure or overtopping of the defence system, including its embankments, floodwalls and the TB (Jeuken and Reeder 2011, p.4). Thus, in the TE2100, the analysis of thresholds in the vulnerability of the existing system was the starting point of the planning process (Jeuken and Reeder 2011, p.4). Such thresholds helped to identify limits for current measures and signalize conditions under which new measures are needed (Reeder and Ranger 2011, p.5).  Key-thresholds for tidal FRM (at which one *portfolio* must change to another *portfolio*) included, for example (EA 2009A, p.139, 149-151, 116, 95, xi-xii):  • The point when tide reaches the maximum design water level at the TB.  • The point when the tide reaches the maximum design water level at other parts of the fixed defence system.  • The point at which the number of closures per year of the TB or other new barriers reaches 50. This represents the operational limit of the TB (an average maximum of 50 closures per year; when this threshold is reached, it will be necessary to raise upriver defences or convert the TB to a barrier with locks).  • the maximum increase in defence levels upriver of the TB (assumed as 1m, due to aesthetic reasons).  • the limit of options involving barriers (assumed to be a 1,5m SLR, as at this point the TB would have to close about 50 times per year, and the upriver defences would be raised by 1m).  • the area required for habitat replacement to be provided by managed realignments.  • the design life of engineering interventions (assumed as 100 years). |
|  |  | - 1c2) Defining FRM policies and assigning one to different areas in the TE (*policy-units*)   Five policies were defined (P1 to P5). Each policy sets the level / standard of FRM justifiable in an area, given the number of people, assets, and value at-risk in such area. The policies served as the basis for the exploration of actions (each policy sets different design criteria for actions). Based on an assessment of how much FRM activity could be justified in different parts of the TE, a policy was assigned to each part, called *policy-unit* (EA 2012, p.32, 34, 44-45; EA 2009A, p.viii-ix, 8, 95, 111, 113, 123-126, 150-151, 266; London Councils’ TEC 2007, p.4; Penning-Rowsell et al. 2013, p.1388). The Plan’s area is divided into 23 *policy-units*. The allocation of policies required assessing the existing flood risk per zone, including the existing SoPs, and examining whether such SoPs should be increased, maintained, or reduced (Ramsbottom & Sheppard 2017, p.9). |
| Identifying measures, designing pathways and appraising them  Development of pathways (HLOs / *Options*) in an iterative way, through the sub-steps 2a, 2b, 2c  The pathways (*Options*) were developed in an iterative way, in three main steps – 2a, 2b 2c (Jeuken et al. 2014, p.6). To develop the pathways – so-called ‘*High-Level Options*’ – the Team devised and applied the ‘Route-map approach’ (EA 2009b, p.3-4). | 2a. Identifying FRM / adaptation measures | Exploring and identifying possible FRM measures (i.e. actions, *responses*)  The Team identified possible / feasible FRM measures, so-called *responses* (EA 2012, p.32; Reeder and Ranger 2011, p.6; Ranger et al. 2013, p.242-243; Ramsbottom and Sheppard 2017, p.4-6, 10-11; Bloemen et al. 2018, p.7). A measure (*response*) is an action to keep *risk below target levels* (as defined in the policy) (HM Treasury 2009, p.23). Various measures to deliver the policies were explored (London Councils’ TEC 2007, p.4).  Firstly, various measures were identified (by experts) through a conceptual analysis based on the High++ scenario (first guess) (Ranger et al. 2013, p.242, 243); these measures were named *Early Conceptual Options* (and presented for public consultation in 2005) [[5]](#footnote-5) (EA 2012, p.34, 32; EA 2009A, p.xi, 141, 266, 296; Ramsbottom and Sheppard 2017, p.6). At this stage, the Team sought to identify all available measures to manage flood risk, either ‘*individual responses*’ or ‘*portfolios of responses’* (combined measures) (EA 2012, p.31-33; Ramsbottom and Sheppard 2017, p.5; HM Treasury 2009, p.23). A measure (*response*) can be either an *individual measure* or a *portfolio of measures* (a combination of measures that work together), and it can be a generic ‘estuary-wide’ measure[[6]](#footnote-6) or a measure for a specific area of the TE (EA 2012, p.34, 32-33; Ramsbottom and Sheppard 2017, p.5-6; HM Treasury 2009, p.23).  Then, each measure (*response*) was examined on its lifetime, engineering limits, effectiveness, environmental implications, and potential for flexibility (for making adjustments over time) (Ranger et al. 2013, p.242; Reeder and Ranger 2011, p.6; EA 2012, p.35; EA 2009b, p.3; Ramsbottom and Sheppard 2017, p.10,11; Bloemen et al. 2018, p.7).  In sum, in Step 2a, the Team explored a wide range of possible measures, and through successive investigations, identified those promising that should be further studied – the *Early Conceptual Options* – which were subsequently used for the development of *High-Level Options* (EA 2012, p.32-33; 2009A, p.141). |
|  | 2b. Designing *pathways* / *routes* (the HLOs, i.e. packages of measures implemented in sequence to manage risk over time) | Developing the *High-Level Options* (pathways)  The Team developed several adaptation pathways / routes, so-called *High-Level Options* (HLOs) (Reeder and Ranger 2011, p.5, 6, 8; Ranger et al. 2013, p.242, 233, 239, 258; Ramsbottom and Sheppard 2017, p.6, 10; Bloemen et al. 2018, p.7; Jeuken et al. 2014, p.6). By using the measures previously identified, and based on the engineering limits and effectiveness of each measure (investigated in Step 2a), diverse pathways were designed (assembled) and represented in a *route-map* (Haigh and Fisher 2010; Ranger et al. 2013, p.242; Reeder and Ranger 2011, p.6). Each HLO is a pathway, i.e. a *package* of FRM measures sequenced and implemented over time (EA 2009b, p.3-4, 1, 10; Reeder and Ranger 2011, p.8; Ranger et al. 2013, p.249; EA 2012, p.34, 38; Jeuken and Reeder 2011, p.4). More specifically, each HLO consists of a sequence of measures (*individual* or *portfolios*) (Ramsbottom and Sheppard 2017, p.4, 2; EA 2009A, p.138-139).   - To develop the HLOs (pathways), the Team applied the Route-map / APs’ approach. The APs approach served to identify the timing for and sequencing of measures and, in this way, develop (assemble) several possible ‘adaptation pathways’ (Ranger et al. 2013, p.233, 239, 258). In other words, the Team has used the measures previously identified, explored their timing and sequencing over time under different amounts/ figures of water level rise, and in this way, designed various possible pathways (ibid). Based on the lifetime, engineering limits and cost-effectiveness of each measure (investigated in Step 2a), several HLOs were assembled and represented in a preliminary route-map (Ranger et al. 2013, p.242). The High++ scenario set the upper bound for the route-map (Ranger et al. 2013, p.243). - The development of the HLOs implied the detection of critical thresholds – i.e. a *threshold analysis* (testing the diverse measures on their suitability under rising water levels) (EA 2009b, p.3, 10), including thresholds for existing defences (e.g. their useful life) and conditions under which a measure no longer meets pre-specified criteria and it is necessary to take a new measure (Ranger et al. 2013, p.250). In this way, it was possible to design various pathways (Ranger et al. 2013, p.233, 239, 258; Reeder and Ranger 2011, p.6). Hence, each HLO / pathway is made up of measures to cope with thresholds over time (Reeder and Ranger 2011, p.6,).   In step 2a, the Team had identified and assessed diverse measures and ‘*portfolios* of measures’ on their effectiveness (e.g. using hydraulic modelling). Using this method, it was possible to identify thresholds (conditions under which a measure ceases to be effective) and explore alternative measures to tackle such thresholds. In this way, the pathways emerged. Several pathways were designed, then tested and refined (leading to the HLOs shown in the route-map) (Ramsbottom and Sheppard 2017, p.10, 5; EA 2009A, p.285-286).   - To design the pathways (HLOs), it was necessary to assemble sequences of measures (*individual measures* or *portfolios of measures*) to manage flood risk over time; which implied analysing when each measure (or *portfolio*) will reach a threshold (the maximum acceptable flood probability) and another measure is needed (Ramsbottom and Sheppard 2017, p.4-5). The *measures* and *portfolios* preidentified (in Step 2a) were used to create (assemble) several pathways (HLOs); by sequencing them in diverse ways, it was possible to produce different pathways (i.e. packages of sequenced measures or sequenced portfolios) (Ramsbottom and Sheppard 2017, p.5; HM Treasury 2009, p.23; EA 2009A, p.149-150; 285-286, 296). Thus, the development of the *HLOs* implied sequencing measures to assemble several possible pathways: when a measure reached a threshold level, another measure was needed, and a pathway emerged. The pathways created (HLOs) are able to deal with differing levels of water level rise (expressed in metres, without considering time at this stage)[[7]](#footnote-7) (Ramsbottom and Sheppard 2017, p.10; HM Treasury 2009, p.23). - The HLOs designed were represented in a *route-map*. // Five possible HLOs (pathways) were developed and represented in a *route-map*. On their whole, the HLOs can cope with the estimated plausible range of increases in water levels in the TE until 2100 (up to 4,2m) (Reeder and Ranger 2011, p.8; Ranger et al. 2013, p.250; Jeuken and Reeder 2011, p.4; Bloemen et al. 2018, p.7; EA 2009A, p.140).   Step 2b resulted in a series (a range) of adaptation pathways that are *‘appropriate to cope with the plausible range of climatic changes that could be seen by 2100*’ (Reeder and Ranger 2011, p.5), and that can be adapted to changes and uncertain future conditions (EA 2009b, p.4, 1). The various pathways keep risk below acceptable levels while maintaining *flexibility* by keeping open options (alternatives) to manage future risk (Ranger et al. 2013, p.233, 239, 258, 249).  The HLOs are a set of five possible pathways (HLO 1, 2, 3a, 3b, 4). Each HLO consists of ‘*a pathway or route through the century that can be adapted to the rate of change that we experience*’ (EA 2009b, p.3). The route-map shows the HLOs under the range of updated SLR scenarios later considered (EA 2009b, p.3, 10). The *HLOs* were produced in 2007 and subjected of extensive online stakeholder engagement (Reeder and Ranger 2011; EA 2009b, p.3; Lowe et al. 2009, p.86).  Overall, the design of the *HLOs* / *Options* (pathways) involved an iterative process, in which measures and ‘portfolios of measures’ were identified and tested on their effectiveness (to detect thresholds) – through *threshold analyses* – and then used to design (assemble) diverse possible pathways, which were subsequently tested against diverse criteria and progressively iterated and refined, and appraised under different SLR and socioeconomic scenarios (substep 3c) (EA 2009b, p.3, 1; Lowe et al. 2009, p.86; EA 2009A, p.x-xii).[[8]](#footnote-8)  Then, each HLO (pathway) was tested under different scenarios, to assess its suitability and robustness (Reeder and Ranger 2011, p.9; Jeuken and Reeder 2011, p.4), i.e. in the next step, the suitability of the HLOs was assessed under diverse futures (associated to different socioeconomic and climate scenarios) (EA 2009b, p.3; Lowe et al. 2009, p.86). The HLOs were refined into *Options* that were then appraised (*Detailed Options* for appraisal), and the most effective (cost-beneficial) option was identified (EA 2009b, p.3; Lowe et al. 2009, p.86).  A *HLO* (later called *Option*) is made up of different sequenced *interventions* (or sequenced *portfolios*) which act together to achieve the recommended policy over time (as illustrated by the TE2100’s *Managed Adaptive approach*) (EA 2012, p.34, 30; EA 2009A, p.xi; 138-139).[[9]](#footnote-9) The Team has developed a series of generic *Estuary-wide Options* to manage flood risk throughout the century; then, these *Estuary-wide Options* were further developed (variations studied) and tested on their effectiveness in meeting the *Vision* (objectives) (EA 2012, p.34, 35, 30).  *Detailed Options for appraisal*  Then, the Team further developed and refined the HLOs into ‘*detailed Options for appraisal*’ under the *Defra06-central scenario* (the *most likely* scenario based on 2010 Government guidance). Assuming this scenario, and drawing on the HLOs, the Team set out four refined generic *Options* (EA 2009b, p.4; Ramsbottom and Sheppard 2017, p.8, 10; Jeuken et al. 2014, p.6) – also called *Estuary-wide Options.*[[10]](#footnote-10) The Team devised four *Estuary-wide Options* (pathways) to manage flood risk throughout this century and deliver the *Strategic Vision* (objectives), though they vary in the way of delivering it (EA 2012, p. 34-35, 30, 6, 56). Subsequently, these *Options* (pathways) were appraised (in Step 2c). |
|  | 2c. Appraise the pathways (Detailed Options), against various criteria, and under different scenarios; compare and rank the pathways | Appraising the ‘*Detailed* *Options*’ (pathways) (*2009 Options Appraisal*)  In this step, the *Detailed Options* (refined pathways) were appraised through a *Formal Options Appraisal* and a *Strategic Environmental Assessment* (EA 2012, p.40; EA 2009A, p.xx, 186, 189, 190-234, 235-241, 266-267; Ranger et al. 2013, p. 237, 239; Ramsbottom and Sheppard 2017, p.11; Bloemen et al. 2018, p.8). Two main methods were used to assess the Options: a *Formal Options Appraisal* including a Cost-Benefit Analysis and a Multi-Criteria Analysis, and a *Strategic Environmental Assessment* (SEA). These methods were used to assess costs, benefits, cost-benefit ratio, and impacts of each Option, compare the Options on these aspects, and identify *the best course of action* (the best Option(s)) (EA 2012, p.46-48; EA 2009A, p.xx, 189, 190-234).[[11]](#footnote-11) The *Options* (pathways) that were appraised are similar to the four HLOs shown in the *route-map* but with some refinements (Ranger et al. 2013, p.251; Penning-Roswell et al. 2013, p.1389; EA 2009A, p.189, 201).  In the *Formal Options Appraisal*, the *Options* (pathways) were appraised / assessed under several scenarios, in a cost-benefit analysis (CBA) and a multi-criteria analysis (MCA) (Penning-Rowsell et al. 2013, p.1386-1387; Ranger et al. 2013, p.243, 251; Reeder and Ranger 2011, p.6; HM Treasury 2009, p.25; Bloemen et al. 2018, p.7; Ramsbottom and Sheppard 2017, p.11, 6; EA 2009A, p.11, 188-234).[[12]](#footnote-12) The *Options* were assessed on their costs, benefits, and impacts, under various scenarios, and also through societal valuation (ibid). The *Formal Options Appraisal* followed on from the adaptation pathways’ analysis. The appraisal employed traditional CBA and MCA to assess a set of four possible Options (with sub-options), which aligned with the four HLOs (with some refinements) (Ranger et al. 2013, p.251). The *Formal Options Appraisal* is reported by Penning-Rowsell et al. (2013) and the technical report of TE2100 (EA 2009A).[[13]](#footnote-13)  In this Appraisal, the *Options* were assessed under different future scenarios: first, the *Options* were appraised on their costs and benefits (CBA) and on their impacts (MCA) under the *Defra06 central* *scenario* (*most likely*), and then, the results of the CBA and MCA were tested under other climate and socioeconomic scenarios (i.e. the prior steps were repeated under different scenarios). Subsequently, the *Options* were ranked under two climate scenarios (the *Defra06 central* and the *High+*) and two socioeconomic scenarios (A and D) (Ranger et al. 2013, p.242, 243, 251; Reeder and Ranger 2011, p.6, 11; Bloemen et al. 2018, p.7-8; HM Treasury 2009, p.25; Penning-Rowsell et al. 2013, p.1392, 1400; EA 2009A, p.232).[[14]](#footnote-14)  The Appraisal allowed the identification of the *Option* with the highest cost-benefit ratio under the *central* *scenario* (*most likely*) and the other scenarios (HM Treasury 2009, p.26), i.e. it showed what was the preferable route under these scenarios (Ranger et al. 2013, p.243; Bloemen et al. 2018, p.7).  The *Options Appraisal* led to the identification of the ‘*front-runner’* *Options*, i.e. the top preferable pathways under diverse scenarios (Penning-Rowsell et al. 2013, p.1383). In the *Defra06 central scenario*, *Option 3.2* is the preferred option followed by *Option 1.4*; in the *High+ scenario*, there is a preference for *Options 3.1* and *3.2*. In socioeconomic scenario A and D, the top options are *Option 1.4* and *3.2*, showing that these options are robust in appraisal terms. Based on the whole appraisal, Penning-Rowsell et al. recommended *Option 1.4* until 2050, however, this Option was incorporated in the proposed post-2050 actions (Penning-Rowsell et al. 2013, p.1395).  Conclusions of the Economic Appraisal: *The “top two” Options suggested by the Appraisal are Option 3.2 (improve existing system, with new standard barrier at Long Reach from 2070), and Option 1.4 (improve existing system, including enhancing the TB from 2070). Before 2050, all Options involve improving the existing system, and the approach taken in Option 1.4 has been shown to be the most cost-effective way of doing this* (EA 2009A, p.233; Penning-Rowsell et al. 2013, p.1401-1402, 1383). The Appraisal results were used to identify the Options that are ‘front-runners’ in terms of benefits and costs, but the choice of main “end of century” intervention will be made later (EA 2009A, p.233). Based on the appraisal results, the preferred “end of century” Options are Option 3.2 and Option 1.4. These preferred Options are included in the Plan (EA 2009A, p.234).  In sum, in Step 2c, the Team appraised the *Options* (on their cost, benefits, and impacts) and identified the *most promising* *Options* (EA 2012, p.32-33, 30, 6), (i.e. the front-runners) (EA 2009A, p.296).  The detailed Options (that were appraised and refined) are mapped in georeferenced maps within the Plan (EA 2012, p.59-65). In the *2009* Technical Report (EA 2009A), each Option was described, including the interventions required in it (that make up such Option) and their dates, under the Defra06 scenario. For each Option, it is provided a table describing the interventions required, and their dates, under the Defra06 scenario (a written description of the Option / pathway), as well as a georeferenced map of each Option under the Defra06scenario (spatial representation) (EA 2009A, p.151-161). |
| Decide on the Plan | 3a. Choose the preferred pathway(s) | 3a: *Decision analysis*: choosing the ‘preferred Option (pathway)’  This step involved choosing the ‘preferred pathway’ under the *most likely rate of change*, and recommending it (Reeder and Ranger 2011, p.6). [[15]](#footnote-15) The final Plan recommends the following: in the period 2010-2069, maintaining and improving the existing flood defence system (HLO1), and, by 2050, make a decision on the *Option* needed to manage flood risk up to the end of the century, which should be in place by 2070, and, thus, by 2050, choose between HLO1 or 3 (Reeder and Ranger 2011, p.6). The *Option* recommended is based on the *Option* that, in the *Options Appraisal*, showed the highest cost-benefit ratio under the *most likely scenario* (HM Treasury 2009, p.26).  More specifically, drawing on the results of the *Options Appraisal*, the Team selected the ‘preferred Option’ (pathway). The Plan recommended the following:   - For the first 60 years of the Plan (2010-2069), ‘*maintaining and* *improving the existing flood defence system*’ (*Option 1.4*) is the recommended option (EA 2012, p.35, 56). The Appraisal showed that *Option 1.4* was the optimal approach for the first 60 years under the expected scenario (*Defra06 central*, deemed the *most likely* in Government guidance) (EA 2012, p.58, 35). - From 2070 onwards, it is expected that a new approach / *Option* will be required to manage flood risk up to 2100 and into the 22^nd^ century (under the expected scenario, the *Defra06 central*) – also called ‘*end-of-the-century Option*’ (EA 2012, p.35, 41, 56). The design and construction of such *end-of-the-century option* will take a lead-time; given this and the expected scenario, it is estimated that a decision on the preferred *Option* will need to be made by 2050 (so that it can be ready in 2070), and, at that moment, all the four *Estuary-wide Options* (pathways) will be under consideration (EA 2012, p.35, 56). In the *Options Appraisal*, the Team identified two front-runner options for the post-2070 period: *Option 1.4* (which involves *continuing the defence improvements* and a *major improvement to the TB*), or *Option 3.2* (which involves *continuing the defence improvements and a new barrier at Long Reach*) (EA 2012, p.56) (the TE2100 *Appraisal* showed two ‘front runner’ options for managing flood risk from 2070 until the end of the century and into the 22^nd^ century – *Option 1.4* and *Option 3.2*) (EA 2012, p.58; EA 2009A, p.xxi). Given the uncertainties regarding the long-term (post-2070) assessment, all the four generic *Estuary-wide Options* (pathways) will remain candidates for appraisal in future reviews of the Plan (namely in the 2050 review) (EA 2012, p.56, 58). [[16]](#footnote-16)   The *Options Appraisal* (led to) allowed the identification of two “front runners” for the period from 2070 onwards:   - - *Option 1.4 – Optimised maintenance and enhancement of the existing system with modifications made to the TB by 2070, and further adapting the structure to become a barrier with locks after 2135.*   - *Option 3.2 – Optimised maintenance and enhancement of the existing system to 2070 and building a new barrier at Long Reach by 2070; (converting to a barrier with locks or “open” barrage after 2135).*   For the period until 2070, *‘maintaining and enhancing the current system’* (in Option 1.4) was preferred regardless of the ‘end-of century’ option chosen thereafter, thus, this is the main recommendation of the Plan (EA 2012, p.47).[[17]](#footnote-17)  As stated in the Plan, to plan effectively for the end of this century (*planning for the long-term*), the Plan identified the *Options 1* and *3* as possible FRM strategies up to the end of this century and beyond, yet, the choice of the ‘*end of the century Option*’ will be made around 2050 in a future review of the Plan based on then-existing conditions (EA 2012, p.38-39).[[18]](#footnote-18) |
|  | 3b. Elaborate ‘implementation plan(s)’: the *Action Plan* | 3b. Elaborating ‘implementation plan(s)’ (*planning the implementation)*  The Team developed the *Action Plan* – i.e. a programme that lays down the actions necessary to manage flood risk over the next 100 years at the estuary-wide level and at local level (EA 2012, p.41, 30). The Action Plan is included within the TE2100 Plan (EA 2012, p.50-220). The *Action Plan* presents the recommended actions for a ‘estuary-wide zone’ (*Action Zone 0*), and for 8 local *Action Zones* (each zone contains various ‘policy units’ with similar characteristics and types of actions) (EA 2012, p.50; EA 2009A, 95, 117-118, 296). In specific, the Action Plan defines: *what* actions must be implemented per Action Zone (according to the FRM policy allocated to each policy-unit), *when* to implement them, *who* will deliver them, and *how* this will be done (EA 2012, p.6, 35, 41, 50-55).[[19]](#footnote-19)There are three phases of implementation in the *Action Plan* (with different objectives associated), and the recommended actions are distributed per time-period: short-term (2010- 2034); medium-term (2035-2049); long-term (2050-2100 / onwards) (EA 2012, p.6, 30, 40-41; EA 2009A, p.xiii, 263).  The *Action Plan* provides, for the first 40 years (until 2049), a detailed investment programme with works for upgrading the existing flood defence system (maintenance and improvement works for existing defences)[[20]](#footnote-20), and, for the period 2050-2100, a high-level programme (based on the *Options* that performed best in the Appraisal under the *most likely* scenario) (EA 2012, p.41, 56).[[21]](#footnote-21)  In the *Action Plan*, the programme for the long-term is based on the *Options* that performed *best* in the *Options Appraisal* (under the then-current conditions), however, CC and other changes over the next 50 years might suggest a different recommended Option for the end-of-the-century; the decision on (choice of) the ‘*end-of-the-century’ Option* will be made in 2050 in a review of the Plan based on then-existent conditions (EA 2012, p.41, 39).  Although the decision on the ‘end-of-the-century’ generic *Estuary-wide Option* will not be made until 2050, two ‘front runners’ emerged in the Appraisal – *Option 1.4* and *Option 3.2* (EA 2012, p.72-73). The Plan’s recommendations are based on the conditions in 2009 (namely the results of the *Options Appraisal*), however, the final decision on the *end-of-the-century Option* will be taken in the 2050 Plan’s review (based on Government guidance); meanwhile, reviews will be carried at 10 yearly intervals at minimum – or more frequently if there are significant changes in one or more indicators; and there will be further consultation each time the Plan is reviewed (e.g. to assess whether the chosen ‘end-of-the-century option’ must be changed) (EA 2012, p.73).  As stated in the Plan, all four *Estuary-wide Options* (pathways) remain candidates for appraisal in future reviews of the Plan (namely in the 2050 review) (EA 2012, p.56, 58). These four *Options* were mapped out in georeferenced maps in the Action Plan under the *Defra06 central scenario* (each *Estuary-wide Option* is represented in a geo-referenced map under the *most likely scenario* –*Defra06 Central*) (EA 2012, p.59-65), each map shows an *Option* translated in space, at the TE scale, including the various measures that together will compose such pathway (located in space and time).  In specific, the Action Plan specifies (EA 2012, p.6, 29, 35, 30, 52-53, 57-65, 44-45; 50-220; EA 2009A, p.xv, 8):   - the policy allocated to each *policy unit* (from P1 to P5, a policy to each of 23 *policy units* that are aggregated into 8 *Action Zones*). - The *Options* (pathways) available to manage flood risk over this century at the estuary-wide level (*Action Zone 0*), i.e. *Estuary-wide Options*. The Plan defined a set of five possible *Options* (pathways). - The actions needed at each *policy unit* (local level) to achieve the policy, over the 3 time-periods (short-, mid-, long-term).   The Action Plan is *adaptable* to changes in a range of indicators (indicators of change), in order to ensure that ‘*the actions that are taken are the right ones, taken at the right time and will not waste money on over-engineered solutions* (EA 2012, p.35).[[22]](#footnote-22) It is also a multi-agency plan (designed to facilitate a multi-partner approach) (EA 2012, p.35). |
|  | 3c Define the monitoring, evaluation, and review system | **3c) Estimating / deriving relevant ‘*decision-points*’**  The Team sought to identify relevant ‘*decision-points*’ and ‘*implementation-points*’, namely those associated to circumstances under which it may be necessary to switch from the selected route to another route (Ranger et al. 2013, p.243, 250).  Using the route-map, the Team identified relevant *decision-points*, a decision-point is a point at which a decision must be made on a given measure, which triggers its planning and construction (Ranger et al. 2013, p.250, 252; Reeder and Ranger 2011, p.9; Ramsbottom and Sheppard 2017, p.12). A decision on measures must be made ahead of when such measures are needed (as most measures require a lead-time of several years lor decades) (EA 2012, p.34).  Decision-points were derived in the following way: for each measure, the Team assessed the threshold at which such measure will be needed (the threshold-value in an indicator, e.g. a water level) and when it will occur in a given scenario; then, assessed the lead-time required to design and implement such measure, and based on these, estimated the *decision-point* (in terms of a value in a monitored indicator, e.g. a water level) including an ‘uncertainty band’ (e.g. about the SLR rate) (Ranger et al. 2013, p.252-253; Reeder and Ranger 2011, p.9; HM Treasury 2009, p.26; Lowe et al. 2009, p.90; Ramsbottom and Sheppard 2017, p.12; Bloemen et al. 2018; EA 2012, p.34, 38; EA 2009A, p.xiv, 278-282, 287-289).[[23]](#footnote-23) This estimation considered the observed values in the indicator[[24]](#footnote-24) (monitored changes), as well as the predicted future changes in the indicator (e.g. when the threshold will be reached under the scenario expected). Thus, the *decision-point* (expressed as a value in an indicator) depends on the monitored /observed changes in such indicator (ibid).  The detection of *decision-points* is conditional on the monitoring of indicators (the observed values in 10 key-indicators, namely observed SLR, may trigger a *decision-point*) (Ranger et al. 2013, p.243, 233, 239, 252; Reeder and Ranger 2011, p.9). The timing for deciding on, and the timing for implementing, a measure may change depending on monitoring results (Bloemen et al. 2018). The results of the monitoring of the indictor (observed change) must be used to update the date estimated for a *decision-point* and an *implementation-point* (Ramsbottom and Sheppard 2017, p.13). If monitoring reveals that an indicator (e.g. the water level) is increasing faster or slower than predicted under the *central scenario*, then, *decision-points* may be *brought forward* or *put back*, *to ensure that decisions are made at the right time and allow an effective and cost-beneficial response* (i.e. to ensure that the real ‘cost-benefit ratio’ of a measure is similar to that envisioned in the *Options Appraisal*) (Ranger et al. 2013, p.254; Reeder and Ranger 2011, p.10; HM Treasury 2009, p.26). Hence, the moment for implementing a new action, and the moment for deciding on it, may be brought forward if the rate of SLR is faster than the projection assumed in the Plan (EA 2016).  Moreover, the timing of a *decision-point* can be estimated under different scenarios; thus, it is important to consider new scenarios that may arise, which may require updating the moment of the *implementation-point*, lead-time, and the *decision-point* (Reeder and Ranger 2011, p.10; Ranger et al. 2013, p.254; Ramsbottom and Sheppard 2017, p.12). The prediction of how the indicator will change must inform the estimation of the *decision-point*, in this way, the moment of this *point* may be updated if new scenarios arise (if new scenarios emerge, and the date estimated for reaching a threshold-value changes, then it is necessary to update the date of the ‘decision-point’). The rate of change predicted in a new scenario may be faster or slower than the assumed by the Plan, which will require bringing forward or postpone the decision-point, respectively.  The Team estimated the date of some important *decision-points*. For example, under the scenario assumed (*Defra06 central scenario*), the first *decision-point* at which the *HLOs* (pathways) start to diverge was estimated to occur around 2050 - the date when a decision should be made on the Option for the end of the century (by then, a choice between *more irreversible* options, e.g. a new barrage, will have to be made) (Ranger et al. 2013, p.253-254; Reeder and Ranger 2011, p.10; HM Treasury 2009, p.26).  This way of estimating *decision-points* was devised to respond to any possible changes in the indicators and potential future updates in projections of water level rise (Ranger et al. 2013, p.254). Decision-points help to ensure that measures are timely taken and cost-effective (Ranger et al. 2013, p.258). |
|  |  | **3c2) Defining the monitoring and review programme**  The Team defined a monitoring programme, including 10 ‘indicators of changes’ (that affect FRM) which must be monitored throughout the Plan’s life, namely mean sea level (relative SLR), peak surge tide levels, erosion, etc. (EA 2012, p.30, 36-37; EA 2009A, p.xvi, 262-263, 283, 291; EA 2016; Ranger et al. 2013, p.233, 256; Ramsbottom and Sheppard 2017, p.12; Reeder and Ranger 2011, Lowe et al.2009; HM Treasury 2009, p.26). The monitoring programme keeps track of the 10 indicators. These indicators serve to monitor changes in the TE and flood risk. The monitoring system must monitor how climate and flood risk are changing locally, which implies measuring local changes in key climate-related variables, but also monitor of global changes and CC progress (the rate of CC effects), which implies keeping track of *updated future projections* and ongoing investment in predictive science and climate services (EA 2009b; Lowe et al. 2009, p.85, 90; Met Office 2012). The indicators help to assess whether the TE is changing in differently than what was envisaged by the Plan, whether the actions / interventions recommended in the Plan need revision, namely whether the actions / interventions recommended in the Plan must be implemented at an earlier or later date (if the timing of FRM interventions must be altered), whether the type of interventions outlined in the Plan needs reviewing, and whether the implemented actions / interventions are adequately managing flood risk (EA 2016, p.15). Moreover, the indicators correspond to key-variables that must monitored to check if a switch of measure or pathway is necessary (Reeder and Ranger 2011, p.6).  Beside this, the Plan must be reviewed and updated (against the ten indicators) every 10 years at minimum, or more frequently if there is a substantial change in one or more indicators; and, in addition, a mid-term monitoring review must be carried out every 5 years (EA 2012, p.30, 39, 36; EA 2009A, p.xv, 262-263; Ranger et al. 2013, p.243, 254; EA 2009b, p.3, 4; HM Treasury 2009, p.26; Ramsbottom and Sheppard 2017, p.13; Bloemen et al. 2018, p.9, 6; EA 2016, p.5). The outputs from the monitoring programme must inform the regular reviews and re-appraisal of the Plan, and may trigger a move on a *decision-point* if a rapid significant change occurs in one or more indicators (EA 2012, p.36-37, 39; HM Treasury 2009, p.26-28), thus, the 10 indicators (monitored through the formal programme) must be reviewed against the Plan (EA 2009A, p.xv, 262-263).[[25]](#footnote-25) Monitoring results must be used to periodically update of the Plan, namely: the dates when actions are needed, or if necessary, its pathways (*Options*), and / or their actions (Ramsbottom and Sheppard 2017, p.13). The Plan’s reviews and re-appraisals must be informed by the monitoring programme, in this way, the need for periodic updates of the pathways can be addressed (Bloemen et al. 2018, p.9, 20, 22). Decisions will need to be taken on accelerating or decelerating elements of the Plan based on actual and predicted rates of change in the indicators (Ranger et al. 2013, p.256).  At each 10-yearly review and update of the TE2100 Plan, the *front-runner(s) Options* for the period post-2070 will be reviewed – and around 2050 a firm decision must be made on the *end-of-the-century Option* (EA 2009A, p.xxi). As part of the regular monitoring and of the periodic planned revision of the TE2100 Plan, it will be necessary to keep the ranking of *Options* under review (EA 2009A, p.232); thus, at each 10-year review, the *preferred Options* must be reviewed using the results of the monitoring of indicators (EA 2009A, p.262-263). Moreover, the design standards of protection for flood defences, the FRM Policy, or floodplain management activities, might need to be changed, as time unfolds – these issues will need to be monitored over time and revisited as part of regular reviews of the TE2100 Plan (EA 2009A, p.232).  The monitoring of indicators is vital component of the Plan; monitoring results must be used to update the Plan’s contents (e.g. the exact date when major interventions will be required will depend on the rate of CC and other changes as these are monitored) (the dates estimated for interventions are likely to change, as the actual rates of change are unlikely to be the same as the assumed rates of change, the indicators must be monitored as part of the Plan, and the intervention-dates must be modified as necessary and the Plan updated) (EA 2009A, p.267).  As indicators are monitored, *monitoring results must be used to update the estimated dates when ‘portfolios’ must be implemented as well as the dates when decisions must be made* (EA 2009A, p.280). The *preferred Options* and the *alternative Options* must be re-appraised, using the updated best estimates of future change, which may lead to a change in the selected *Option*. Moreover, while the Plan contains alternative Options, it will be necessary to consider whether there are any other alternative options whenever the *Options* are reviewed (EA 2009A, p.280), i.e. it is necessary *to consider whether the preferred options* identified in 2009 *are still the best; appraisal is (…) needed to decide the best way forward whenever the Plan is updated* (EA 2009A, p.280).  The Plan’s recommendations were based on 2009 Government guidance on CC; however, if CC predictions (or other pressures) worsen, then the date of an intervention might be changed – this was allowed for in the Plan (EA 2009A, p.xiii). Moreover, if the10-yearly review of the Plan identifies that a different *Option* is then preferred, this is possible as long as the final decision is made with sufficient lead-in time to implement it (e.g. an act of parliament may be required); whatever the current end-of-the-century Options is recommended, it was important to have a preferred Plan (*preferred Option(s)*) as a basis from which stakeholders could plan for the future (EA 2009A, p.xiii).  The mid-term monitoring-review process (every 5 years), in turn, serves to analyse the changes (physical and socioeconomic changes) that are occurring in the Estuary over time, based on the 10 indicators, and assess whether the FRM policies and recommendations of the Plan require any adjustment, whether it is necessary to review the timing of FRM actions and whether the recommendations remain the right choices for the TE (EA 2016, p.5). In specific, the 5-year monitoring review serves to assess how the TE is changing (its physical environment and socioeconomic environment) and how this affects flood risk, whether the Plan’s objectives are being met, and whether the flood defences are being managed as recommended by the Plan (EA 2016, p.5), and evaluate whether the actions in the Plan remain appropriate and ensure they are implemented at the right time (EA 2016, p.15). Although this mid-term monitoring review was set every 5 years, if monitoring in the interim period reveals that a given indicator is changing faster (or slower) than expected, the *implementation-point* and *decision-point* of a measure may be anticipated (or postponed) (HM Treasury 2009, p.26-27; Ranger et al. 2013, p.254; Reeder and Ranger 2011, p.10). In practice, the *decision-points* and *implementation-points* of measures (and measures themselves, as well as the pathways – i.e. the *Option* and the individual elements of the *Option*) may be modified in light of new information on indicators (HM Treasury 2009, p.27).  The effectiveness and success of the TE2100 Plan will strongly depend on a continuing process of ongoing monitoring (of local changes and conditions) and periodical / regular review of the Plan (its re-appraisal and update) (Reeder and Ranger 2011, p.10; Ranger et al. 2013, p.254; EA 2009b, p.10; Lowe et al. 2009, p.90, 85), namely through the review of decisions in light of observations of SLR, new knowledge, and updated projections (Reeder and Ranger 2011, p.10).[[26]](#footnote-26) It is, therefore, essential to monitor how the TE and flood risk are changing (which requires measuring local changes in key-variables / indicators), but also ensure that *‘the Plan is regularly reviewed*’ (which implies keeping track of global changes, namely CC, its effects, their rate and progress, and updated future projections) (EA 2009b, p.3, 4, 10; Lowe et al. 2009, p.90). Overall, the TE2100’s success depends on ‘*monitoring and adjusting as the century progresses*’ (Lowe et al. 2009, p.85), or, more precisely, ‘*monitoring and adapting to changes*’ (Met Office 2012, p.2), i.e. adapting the Plan and the physical system.  The Plan was designed to be *adaptable to change and remain fit for purpose throughout its 100 year life* (EA 2012, p.36; EA 2009A, p.xv)[[27]](#footnote-27); in particular, it should be adjustable (and respond) to possible changes in any indicator or updated projections (Ranger et al. 2013, 254-255) (see *decision-points*). The key-indicators must be monitored throughout the Plan’s life, and the regular reviews (re-appraisal of the Plan) must be carried out (based on monitoring outputs), to ensure that the Plan remains *adaptable* and *fit for purpose* (thus, that it responds adequately to changes) (EA 2012, p.36, 38, 39; EA 2016).  There are various ways to respond to changes, as these are monitored, or each time the TE2100 Plan is reviewed or updated (EA 2012, p.39), e.g. by adjusting the date of *implementation-points* and *decision-points* of measures, or by shifting of measure (or pathway), or altering (changing) the measures themselves.  The monitoring, and the periodic review of the Plan, allow for assessing if an eventual change (switch) of, measure or route (pathway), or anticipation / postponing of actions, will be needed (Reeder and Ranger 2011, p.6; HM Treasury 2009, p.27-28).[[28]](#footnote-28) Based on the observed and projected changes in the key-indicators, decisions will need to be taken on accelerating / decelerating the elements of the Plan (namely the actions, Options, decision-points) (Ranger et al. 2013, p.243), e.g. a higher rate of SLR may require accelerating measures, or high rates of erosion in the existing defences could accelerate the need to upgrade defences (Reeder and Ranger 2011, p.6). In the Plan’s reviews, it is also necessary to evaluate if the recommended *Option* (pathway), and its measures, still provide the ‘highest cost-benefit ratio’ under the then-predicted as the ‘most likely’ scenario (HM Treasury 2009, p.28).  The Options ranking *will need to be kept under review as part of regular monitoring and periodic planned revision of the TE2100 Plan* (EA 2009A, p.232). The design standards of protection for flood defences, Policy level, or floodplain management activities, will need to be monitored over time and revisited as part of regular reviews of the TE2100 Plan in the future (EA 2009A, p.232). |
| Implement | 4. Implement the plan – its actions / interventons (i.e. the measures envisioned) | The final TE2100 Plan was published in November 2012 (Ranger et al. 2013; EA 2016; Ramsbottom and Sheppard 2017, p.14). After the publication, the *implementation partners* to whom the Plan sets recommendations should deliver their actions, namely local authorities (who have a key role in delivering the recommendations for spatial planning and emergency procedures), Natural England, and English Heritage (EA 2012).  Since the Plan’s publication and approval by the Government, the implementation of recommended actions, and the first scheduled investments and works, began (EA 2012, p.41). To streamline the implementation of actions, an organisation called *TEAM2100* (composed by EA staff and consultants) signed a 10-year contract to refurbish, improve and replace flood defences in the TE, namely the TB. Furthermore, new works on port facilities and other urban developments have been built in line with the Plan (Ramsbottom and Sheppard 2017, p.12,14-16). |
| Monitor, evaluate, and review | 5. Ongoing Monitor and evaluate, and review the Plan (feed-back results to steps 1+2)  Carrying the monitoring and review programme | The ongoing monitoring, as well as the regular reviews of the Plan, have been undertaken. The Plan has been reviewed at ten-year intervals (at least) against the ten indicators.  The regular monitoring of indicators was initiated in 2012 and has proceeded as required to allow the regular review of the Plan (Ramsbottom and Sheppard 2017, p13). The EA, together with the Met Office and others, has conducted the monitoring and review programme (EA 2009b, p.6; Reeder and Ranger 2011, p.10; Lowe et al. 2009, p.90).  The first 5-year Monitoring-review Report was published in 2016 (EA 2016), and, in general, it confirmed that the TE has been changing as expected by the Plan: the changes observed in the indicators, and the future projections, were generally consistent with those expected in the Plan, thus, it was not necessary to modify the content nor the timing of actions; however, some indicators have been refined (EA 2016; London Councils 2018; Ramsbottom and Sheppard 2017, p.13-15). The *implementation partners* should continue to deliver their actions as planned (EA 2016; London Councils 2018).  Importantly, this step may lead back to previous steps (Step 1 or Step 2). |

**Table 1.** Description of the process of development of a robust adaptive plan in the TE2100 case. Source: own elaboration based on several references.

# NOTE 2. How the DP developed and followed the ‘ADM approach’: process of steps

The ADM approach defines its own process of steps necessary for developing an *adaptive plan/strategy* and operationalizing an *Adaptive Delta Management*. This section describes the process of ADM: first it identifies its main steps, then it explains how these were carried out and applied in the DP, namely how the APs method was used to develop an *adaptive programme* with *flexible adaptive strategies*. It builds on the analysis of the DP Reports (DP 2012, 2013, 2014, 2015) and prior studies (e.g. Bloemen et al. 2018; Restemeyer et al. 2017; Gersonius et al. 2016; etc.).

## 2.1. ADM process and its steps

The ADM approach involves a phased decision-making process (DP 2012, p.88) that includes the design of adaptation pathways. The process of ADM is based on the process of the DAPP approach (presented by Haasnoot et al. 2013 and by Jeuken et al. 2014), and it involves a circular (on ongoing / continual) cycle with 6 main steps (inspired in the DAPP steps) (Deltares 2018, adapted from Haasnoot et al. 2013; Brugge and Bruggeman 2019). These steps are:

- *Step 1. Analyse vulnerabilities and opportunities under different scenarios*. This step, also called ‘*problem analysis*’, serves to examine the possible occurrence of problems (risks, hazards, adverse impacts), their nature, extent and timing, under diverse future scenarios (Deltares 2018, p.2-3; Brugge and Bruggeman 2019). It involves: a) the definition of the main objectives for now and for the future, and b) the identification of current and future ‘vulnerabilities’ (external developments to which the objectives are most vulnerable or that may threaten the objectives, i.e. problems, needs, challenges) and ‘opportunities’ to achieve the objectives, under different future scenarios (Jeuken et al. 2014, p.4-5; Haasnoot and Jeuken). Sub-step 1a involves the definition of objectives, i.e. a ‘*strategic vision of the future*’, goals and criteria for success (also called ‘agenda setting’) (Marchand and Ludwig 2014; Zevenbergen et al. 2018; Haasnoot and Jeuken; Gersonius et al. 2016)[[29]](#footnote-29); ADM assumes that clear goals can be defined to then find measures and design ‘development trajectories’ (Zandvoort et al. 2018, p.190). Sub-step 1b involves the identification of current and future problems and needs based on relevant future scenarios (also called ‘*problem analysis*’, this requires using scenarios) (Marchand and Ludwig 2014; Zevenbergen et al. 2018; Haasnoot and Jeuken; Gersonius et al. 2016). In particular, Sub-step 1b requires analysing the amount of change that the system can handle (critical levels) and specifying (what are) the *Adaptation Tipping-points* (ATPs, i.e. conditions / points at which the objectives cease to be met), and assessing when the first ATP might occur using various scenarios (Jeuken et al. 2014, p.4-5), i.e. ‘*how long the current management strategies continue to be effective under different climate change scenarios*’ (Deltares 2018, p.2). Moreover, Sub-step 1b implies the identification of constraints and uncertainties relevant for decision-making, and such uncertainties must be considered in the generation of an ensemble of plausible future scenarios (Haasnoot and Jeuken; Gersonius et al. 2016)[[30]](#footnote-30); such scenarios are then compared to the defined objectives to see if problems (vulnerabilities), or opportunities, arise; and they are used to determine when the first ATP may occur and a new measure will be needed (Haasnoot and Jeuken). Thus, within Step 1b, it is necessary to specify (define) what are ‘adaptation tipping-points’, i.e. the ‘boundary conditions’ / points under which the objectives are no longer met (e.g. an unacceptable level of SLR) (Kwadjik et al. 2010), and then, confront such ATPs with a range plausible futures to estimate the moment when such ATPs might occur; it is important to use different futures with changing conditions to gain insight into the possible occurrence of ATPs, and estimate when the first ATP might happen (the point at which the current measure will no longer meet the objectives and new measures will be required) (Gersonius et al.2016, p.204-206). Thus, Step 1 implies analysing what, where, and when problems will occur under the different scenarios – a problem occurs if the policy objectives (e.g. flood safety, flood protection standards, required water levels) are no longer met, which is also denominated a ‘tipping-point’ (Step 1 requires analysing the nature, extent and timing of the problems in diverse scenarios) (Brugge and Bruggeman 2019, p.4-5).
- *Step 2. Identify measures and options and assess their efficacy.* It involves identifying possible measures to solve the problem (risk management / adaptation actions) and assessing their efficacy (Deltares 2018, p.2-3; Marchand and Ludwig 2014, p.3; Brugge and Bruggeman 2019, p.4-5).[[31]](#footnote-31) In this step, it is necessary to: (a) search for and identify possible measures / actions available based on the vulnerabilities and opportunities found in Step 1, and (b) assess the performance and efficacy of each measure in light of the predefined objectives to determine its tipping-point (by exploring for how long a given measure will be effective, and the timing of this is largely influenced by the scenario considered); and, then, exploring which other measures are available) (Haasnoot and Jeuken; Brugge and Bruggeman 2019, p.4-5; Gersonius et al. 2016, p.204-206, 202).[[32]](#footnote-32) In the DAPP approach (which inspired ADM), an *adaptive plan* should contain measures for the short-term and options for the long-term, including measures that should be taken now to keep options open to adapt the plan (its strategies or measures) if necessary in the future (e.g. by changing or switching of measure) (Haasnoot and Jeuken).[[33]](#footnote-33) Through an iterative assessment of measures’ efficacy and tipping-point, it is possible to select the most successful measures; and, once a set of measures seems adequate, it is possible to build various possible pathways (Jeuken et al. 2014, p.4).
- *Step 3. Develop adaptation pathways and the APs map*. It consists of the design of adaptation pathways (APs) (Haasnoot and Jeuken; Gersonius et al. 2016, p.204; Brugge and Bruggeman 2019, p.4-5). A pathway is a sequence of measures that may be activated before an ATP occurs (Gersonius et al. 2016, p.204). A pathway is a sequence of possible measures (assembled with measures identified in Step 2). This step requires constructing / assembling several possible pathways (sequences of measures) by using the promising measures identified in Step 2 as building blocks, and then, representing such pathways in an APs’ map (and the costs and benefits of each pathway may be presented in a scorecard) (Haasnoot and Jeuken; Jeuken et al. 2014, p.4-5).[[34]](#footnote-34) Crucial to design the APs are the adaptation tipping-points (i.e. points/ conditions under which a measure no longer meets the defined objectives); when an ATP is reached, other / additional measures are required to achieve the objectives. The APs map shows which measures are available and when these will be needed (Deltares 2018, p.2-4). Therefore, in this Step, it is necessary to design various adaptation pathways in a map (based on the assessment of the efficiency of the individual measures identified in step 2); the various pathways provide flexibility to adapt to a wide range of future changes; the APs’ map shows the points in time for implementing measures (Gersonius et al. 2016). To design the adaptation pathways in a map, the method of Haasnoot et al. (2012, 2013) is used; adaptation pathways are designed as series of sequenced measures that provide a solution to problems (*in* Brugge and Bruggeman 2019). This step also implies the assessment of the proposed strategies (pathways) on their *robustness* and *flexibility*, under different scenarios; in addition, other criteria, e.g. effectiveness, costs, side-benefits, *impacts*, *uncertainty involved*, *desire to keep options*, may be used; then, the results of this assessment can then help to select a subset of promising pathways (it is important to include inputs from different actors and seize opportunities to link different investment agendas) (Gersonius et al. 2016, p.204-206; Zandvoort et al. 2018, p.189; Brugge and Bruggeman 2019, p.4-5).[[35]](#footnote-35) The pathways are generated (assembled) and then assessed, which may lead back to Step 2 in an iterative way (Brugge and Bruggeman 2019, p.4-5). Several methods can be used to assess measures and pathways, their ATPs and effects, e.g.: cost-benefit, cost-effectiveness and multi-criteria analyses; robustness analysis (focused on measures’ performance under diverse scenarios); feasibility analysis (focused on barriers and facilitators) (Deltares 2018).[[36]](#footnote-36)
- *Step 4. Design an adaptive plan, and define triggers*. This consists of formulating an *adaptive plan*. It implies: (a) the appraisal of the various pathways on their social and economic feasibility, robustness and flexibility[[37]](#footnote-37), and the selection of one or more (preferred) pathways as input for a *dynamic adaptive (action) plan*, and (b) the specification of indicators (signposts) and triggers (critical values at which it is necessary to activate actions, or make adjustments or change of measure; triggers act as warning signals for the implementation of actions or reassessment of the plan, and must be monitored) (Deltares 2018, p.2-3; Jeuken et al. 2014, p.4-5; Haasnoot and Jeuken; Marchand and Ludwig 2014; Brugge and Bruggeman 2019, p.4-5; Gersonius et al. 2016, p.204-206).[[38]](#footnote-38) Step 4b requires the definition of a monitoring system that collects information on indicators and triggers (Haasnoot and Jeuken) and mechanisms to adjust the plan (e.g. its strategies) if necessary (Deltares 2018, p.2-3). Step 4b implies the definition of a monitoring and evaluation system that specifies ‘triggers’ (critical points beyond which it is necessary to make adjustments to the strategy or shift to another measure) and ‘preparatory actions’ (required to enable long-term options, e.g. adjustments in rules / legislation, research, spatial reservations, etc. (Gersonius et al. 2016, p.204-206). A trigger may indicate that it is necessary to activate *contingency actions* (to keep the pathways open as long as possible) (Haasnoot and Jeuken). To keep open the preferred pathway as long as possible, it may be necessary to specify contingency actions (Jeuken et al. 2014, p. 4-5). It is also important to integrate measures with investments of other actors (Zandvoort et al. 2018, p.189).
- *Step 5. Implement the plan*. This means implementing the plan’s measures, namely measures necessary to keep open options that may be needed in the future (Deltares 2018, p.2-3; Jeuken et al. 2014). The selected short-term measures are implemented, as well as measures necessary to keep options open in the long-term (and, thus, maintain flexibility).
- *Step 6. Monitor, reassess the plan (its strategies or measures), and adjust it, if necessary*. This step implies the monitoring and evaluation of external changes, implemented measures, and progresses in knowledge, and the reassessment of the plan, and, if necessary, its review (Haasnoot and Jeuken). It requires a monitoring and evaluation system to keep track of climatic and socioeconomic developments, and assess if and when it is necessary to review or adjust the plan (e.g. change its strategies or measures) (Deltares 2018, p.2-3). The monitoring and evaluation system must keep track of climatic, physical, and socioeconomic trends (monitoring climatic and socioeconomic developments is necessary to identify when it is necessary to adjust a strategy, e.g. to shift from a measure to another) (Deltares 2018, p.5). Step 6 is essential to allow a real adaptive planning and management. Variables that may induce to triggers must be monitored (Jeuken et al. 2014, p. 4). In this step, the monitoring and evaluation system is conducted: it tracks climatic and socioeconomic changes relevant for adapting the plan’s strategies or measures, e.g. for anticipating / delaying the implementation of measures; e.g. if SLR unfolds at a faster rate than expected and a tipping-point is expected to be reached earlier, it may be necessary to implement a measure earlier than projected (if SLR evolves slower, then, the measure can be delayed). The monitoring system indicates the rate and direction of risks and their implications for adjusting the plan, it allows the adjustment of the plan - its measures or their timing (Brugge and Bruggeman 2019, p.4-5).

In sum, ADM involves a cyclical and iterative process of 6 main steps. Each step offers the possibility to adjust elements of the plan / strategies, and, thus, helps to ensure adaptability in the general plan, and in planning and management process. Adaptability is necessary to cope with change and uncertainty in policymaking: it is not known what scenario will unfold, but it is still necessary to make decisions (Brugge and Bruggeman 2019, p.4). ADM offers a structured management process that considers uncertain future conditions and changes into planning and decision-making (Zevenbergen et al. 2018).

ADM process differs from the traditional adaptation planning approaches as the starting point of the analysis is the question of ‘*how long will the current strategies be effective under different scenarios*’, which leads to the exploration of alternative measures and the design of multiple solution pathways (rather than a single solution that is designed for a ‘business-as-usual’ scenario or worst scenario). The basic idea underlying the APs approach is *to generate a wide array of pathways* (each pathway *consisting of a series of measures*) *through which the policy objectives are achieved under changing climate and socioeconomic conditions* (Deltares 2018, p.4, 3). The set of pathways presented in an AP’s map provides a range of options that may be used in future planning, thus, the APs approach helps to enhance the plan’s and the system’s flexibility and adaptability (capacity to adapt to multiple plausible futures and changes) (Deltares 2018, p. 4, 2).

## 2.2. Annual cycle of development of the DP: steps until the *Delta Decisions* and *Preferential Strategies*

The DP has followed an annual development cycle[[39]](#footnote-39), and, in this cycle, the DP has carried out the main steps of ADM process (or similar steps to those prescribed in ADM) (DP 2016, p.6). In the year 2011/2012, each regional Subprogramme explored *Possible Strategies* for its region, i.e. possible measures / solutions. In the year 2012/2013, these *Possible Strategies* were further developed into *Promising Strategies*, which consist of the measures and, in some cases, the initial *paths*, which are presented in the DP2013. In the year 2013/2014, the *Promising Strategies* were further detailed which led to a *Preferential Strategy*, which shows the final *adaptation path(s)* chosen, and such *Preferential Strategy* is presented in the DP2014. The *Preferential Strategies* correspond to the final strategies proposed, which were adopted in 2015 (DP 2013, p.56, 78).

The DP has followed the ADM process, which has consisted of a phased planning / decision-making and management cycle with six main steps (inspired by the DAPP approach), including the design of adaptation pathways. **Figure 2** illustrates the process of development of the *Preferential Strategies* of the DP Subprogrammes, using ADM (it provides a detailed depiction of the process of development of robust adaptive Strategies in the Subprogrammes).


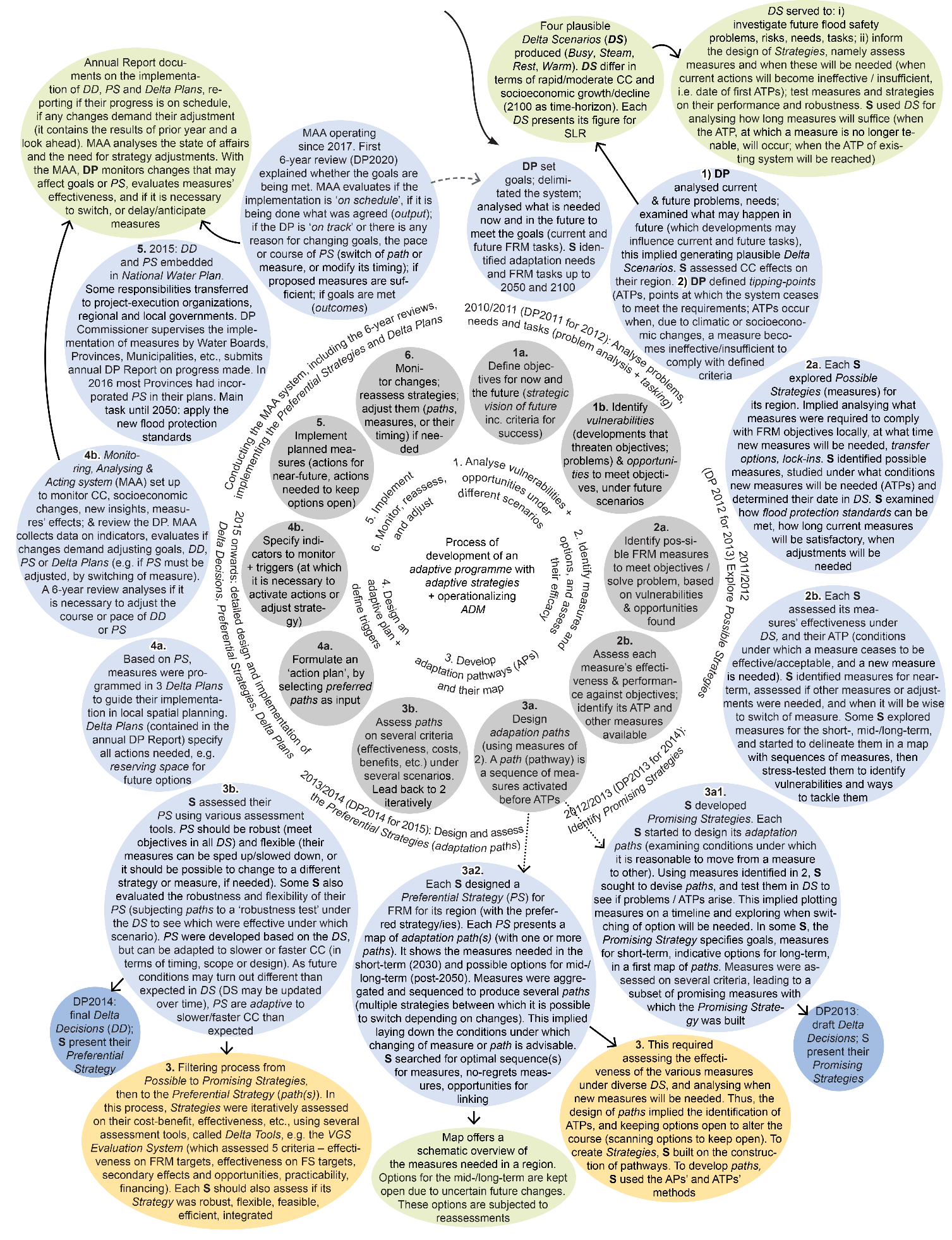


**Fig. 2** ADM process in the DP and its Subprogrammes, showing the various steps of development of the Preferential Strategies devised by each Subprogramme. Source: own elaboration, based on DP 2011, 2012, 2013, 2014, 2016, etc.

**Table 2** summarizes the process of ADM followed in the DP: it identifies its main steps, and explains how these were applied in the DP and its Subprogrammes, the main years of elaboration of important DP contents, and examples of the work carried out in each phase in the Subprogramme for the Rhine Estuary-Drechtsteden (RE-D).

| **Table 2. Steps of the ADM process, and their application in the DP and its Subprogrammes** | | | |
| --- | --- | --- | --- |
| **STEP OF ADM** | **Main step explanation** | **Sub-steps as applied in ADM** | **Main years** |
| *Step 1. Analyse vulnerabilities and opportunities, under different scenarios (also called ‘problem analysis’)* | This step involves: (a) the definition of the objectives for now and the future (a strategic vision for the future, goals and criteria for success), and (b) the identification of current and future vulnerabilities (external developments to which the objectives are vulnerable or that threaten them, i.e. current and future problems / needs / challenges) and ‘opportunities’ to achieve the objectives (Jeuken et al. 2014, p.4-5), under relevant future scenarios (Haasnoot and Jeuken; Marchand and Ludwig 2014; Zevenbergen et al. 2018; Gersonius et al. 2016; Zandvoort et al. 2018). Sub-step 1b requires analysing the amount of change that the system can handle (critical levels) and specifying (what are the) *Adaptation Tipping-points* (i.e. ATPs, conditions / points under which the objectives cease to be met (e.g. an unacceptable level of SLR) (Kwadjik et al. 2010), and assessing when these occur (by confronting such ATPs with a range of plausible futures scenarios to estimate the moment when they might occur), and, in specific, assessing /estimating when the first ATP might occur using various scenarios (Jeuken et al. 2014, p.4-5; Gersonius et al.2016, p.204-206), e.g. ‘*how long the current management strategies continue to be effective under different CC scenarios*’) (Deltares 2018, p.2). Sub-step 1b also implies the identification of constraints and uncertainties relevant for decision-making (e.g. uncertainties about future climatic and socioeconomic changes), and such uncertainties must be considered in the generation of an ensemble of plausible future scenarios (Haasnoot and Jeuken; Gersonius et al. 2016). Such scenarios are then compared to the defined objectives to see if problems (vulnerabilities) or opportunities arise, and to determine when the first ATP may occur and a new measure will be needed (Haasnoot and Jeuken).  Overall, Step 1, also called ‘*problem analysis*’, serves to examine the occurrence of possible problems (risks, hazards, impacts), their nature, extent, and timing, under diverse future scenarios (Deltares 2018, p.2-3; Brugge and Bruggeman 2019). It consists of analysing when and where problems will occur under the different scenarios. A problem occurs if the policy objectives (e.g. flood protection standards, required water levels, flood safety) are no longer met; this is also denominated a ‘tipping-point’. Step 1 requires analysing the nature, extent, and timing of the problems in diverse scenarios (Brugge and Bruggeman 2019, p.4-5).  ………………  Step 1 involves: (a) the definition of objectives, i.e. a ‘*strategic vision of the future*’, goals and criteria for success (also called ‘*agenda setting*’); (b) the identification of current and future problems and needs, based on relevant future scenarios (also called ‘*problem analysis*’, it requires using scenarios) (Marchand and Ludwig 2014; Zevenbergen et al. 2018; Haasnoot and Jeuken; Gersonius et al. 2016). Step 1a implies defining a *vision* (a framework of policy goals); ADM assumes that clear goals can be defined to then find measures and design ‘*development trajectories’* (Zandvoort et al. 2018, p.190).  In Step 1b, it is necessary to specify (define) what are ‘adaptation tipping-points’, i.e. the ‘boundary conditions’ / points under which the objectives are no longer met (e.g. an unacceptable level of SLR) (Kwadjik et al. 2010), and then, confront such ATPs with a range plausible futures to estimate the moment when such ATPs might occur. It is important to use different futures with changing conditions to gain insight into the possible occurrence of ATPs, and estimate when the first ATP might happen (the point at which the current measure will no longer meet the objectives and new measures will be required) (Gersonius et al.2016, p.204-206). In Step 1, it also is important to: analyse the current policy measure; translate the objectives into tasks for the short- and long-term; and define metrics / parameters to describe changing conditions, e.g. the rate of SLR in cm/year (Gersonius et al.2016, p.204-206). | Substep 1a (definition of a *vision*, i.e. objectives, ambitions, and tasks) The Team elaborated a vision of how to deal with the risks and problems expected the future.  The DP was initiated in 2010.In the year 2010/2011, the Subprogrammes identified the *tasks* of FRM and FS up to 2050 and 2100 (DP 2014, p.132; 2012, p.43). To identify the tasks, the Teams examined ‘*what is needed, now and in the future*’ to meet the objectives (DP 2011, p.14). Joint-fact finding was used to identify the tasks for the 9 Subprogrammes, and the various *Delta Scenarios* were used (DP 2011, p.14). The DP 2011 presents the tasks for the short-, mid- and long-term, and outlines the process to be followed each year to develop the *Delta Decisions* and regional *Strategies* until 2015 (DP 2011, p.14).  In 2010, the DP started to develop a set of *Delta Scenarios* to be used in all studies in the DP, and an evaluation system to assess solutions (DP 2010, p.4). The *Delta Scenarios* were issued in 2011, enabling further agreement on the range of future effects that should be considered (Werners et al. 2016).  Moreover, in this period of ‘problem definition’, the Regional Subprogrammes sought to identify adaptation needs, by analysing ‘*what are the effects of CC on the region’* and ‘*how can the long-term safety of the region be safeguarded*’ (Werners et al. 2016).[[40]](#footnote-40)  Substep 1b (analysis of vulnerabilities and opportunities under different scenarios)  The DP analysed current and future situation and problems (Gersonius et al. 2016, p.205); in specific the DP analysed which developments or changes might influence the current and future tasks on FRM and FS (DP 2012, p.88). The analysis of *what might happen in the future* required the generation of the four *Delta Scenarios* (Klijn et al. 2016).  Working with several plausible futures: the four *Delta Scenarios*  To deal with uncertainties about future changes, the DP devised and used four different plausible futures, so-called *Delta Scenarios* (DP 2014, p.135; 2013, p.6). The *Delta Scenarios* are different plausible scenarios which show how the climatic and socioeconomic conditions might change until 2050 and 2100 (Brugge and Bruggeman 2019 p.3). The *Delta Scenarios* assume 2050 and 2100 as time-horizons (DP 2013, p.100; 2011, p.48, 70).  The *Delta Scenarios* (*Busy*, *Steam*, *Rest* and *Warm*) combine rapid / moderate CC with socioeconomic growth / decline, and present their corresponding figures (DP 2011, p.14, 46; 2012, p.35; 2013, 100; Marchand and Ludwig 2014; Deltares2018; Brugge and Bruggeman 2019).// The four *Delta Scenarios* differ in terms of moderate/ rapid CC (X axis), and of socioeconomic growth / decline (Y axis) (DP 2012, p.35; 2014, p.168; Marchand and Ludwig 2014; Haegen and Wieriks 2015; Alphen 2015; Restemeyer et al. 2017; Bloemen et al. 2018). Each scenario has its corresponding climatological and socioeconomic circumstances and presents its respective figures for SLR, drought and soil subsidence (DP 2016, p.6, 2014, p.136). The climatic parameters considered in these scenarios were SLR, extreme river discharge, precipitation, evaporation; whereas the socioeconomic parameters were future size and spatial distribution of population and land uses (Alphen 2015; Haegen and Wieriks 2015; Jeuken et al. 2014).  Though each *Delta Scenario* has its respective climatological and socioeconomic figures, future circumstances may turn out differently than expected in the *Delta Scenarios*, therefore, the Preferential Strategies were designed to be *adaptive to slower and faster CC* than expected (DP 2014, p.136). Moreover, the *Delta Scenarios* may be periodically updated over time (DP 2014, p.168).  The *Delta Scenarios* served to assess possible future risks and impacts (Jeuken et al. 2014, p.10, 11); investigate future flood- and water-related problems, risks and needs by 2050 and 2100 (they represented the ‘corner flags of the playing field of plausible futures’) (Alphen 2015, p.312). These scenarios translate the most relevant uncertainties about plausible future changes and diverse sociocultural perspectives (Marchand and Ludwig 2014, p.15). In 2012, the *Delta Scenarios* were used by the Subprogrammes to explore what problems of flood safety and freshwater supply might occur in the future (Brugge and Bruggeman 2019, p.5; Haegen and Wieriks 2015). The *Delta Scenarios* were also used as the basis for analysing the future tasks (DP 2011, p.14, 20).  In 2010/2011, the DP Teams used the *Scenarios* to assess how much longer the current measures (policy and management actions) are expected to suffice and when adjustments will be required – i.e. when the tipping-point of the existing system will be reached (DP 2010, p.3-4, 32). The main issue was analysing if and for how long the current measures will still be satisfactory under a changing climate (more than determining the exact levels of SLR) (DP 2010, p.36). The DP defines *tipping-points* as points at which the existing system ceases to meet the requirements (DP 2011, p.8); a tipping-point occurs when, due to changes in climate or socioeconomic circumstances, the existing measure, policy, or infrastructure, becomes insufficient to comply with the defined criteria (due to physical, technical, or financial constraints or socially unacceptable effects) (DP 2011, p.71). The analysis of ATPs sets out when decisions and new measures need to be taken (DP 2011, p.55). The DP Teams analysed for how long the current policy measure will suffice and when the first tipping-point (at which such measure is no longer tenable) will be reached. To answer this, scenarios were used (DP 2010, p.68). | Year 1 (2010/2011): Analysis of tasks, problems, and needs (*tasking*/ *problem analysis* / *problem definition*). It resulted in the DP 2011 (for 2012).  This year involved: setting the goals and ambitions and delimitating the system, and b) identifying uncertainties, generating ‘*Delta Scenarios*’, and specifying Tipping-points (Rhee 2012, p.9; Werners et al. 2016).  In 2010/2011, the RE-D Subprogramme analysed the future tasks. The tasks for short-term (until 2028) were based on findings of the Statutory Assessment of primary flood defences, and on the new flood protection standards defined. In this region, the main pre-existent FRM measures were *‘dyke improvements*’ and ‘*repairs of storm surge barriers*’. New measures will be required in the mid-term (until 2050) due to expected changes, e.g. SLR.  The Team used two *Delta Scenarios* (*Steam* and *Rest*) to analyse the tasks stemming from these changes. Moreover, the Subprogramme examined if the existing levels of protection (and standards) were still sufficient, and found that several parts had to increase their protection level; the DP 2014 proposed new protection standards in the *Delta Decision on FRM* (Gersonius et al. 2016, p.207).  Thus, the tasks resulted from external developments expected in the mid-term (e.g. SLR) and internal changes (new flood protection standards set in the *Delta Decision on FRM*).  A key question that arose was whether the existing strategy / measure, i.e. ‘dyke improvements’, was adequate to meet the objectives and tasks and the new protection standards in a cost-effective way, or whether a shift to another measure was required (Gersonius et al. 2016, p.208). The complexity of improving dykes, or the desire to reduce investments in dykes, among other factors, led to the need to explore and develop other strategies (than traditional ‘dyke strengthening’, for example, based on the three layers of the ‘ML FRM’). |
| *2. Identify measures and options, and assess their efficacy* | This Step involves: a) identifying measures / actions to solve the problem and meet the objectives and tasks, i.e. management / adaptation actions, based on the vulnerabilities and opportunities found in Step 1 (it implies exploring possible measures to manage risk, namely to reduce the risk probability, or system’s vulnerability, or increase its resilience); and b) assessing the performance and efficacy of each measure in light of the predefined objectives to determine its tipping-point (Haasnoot and Jeuken; Deltares 2018, p.2-3; Marchand and Ludwig 2014, p.3; Brugge and Bruggeman 2019, p.4-5; Gersonius et al. 2016, p.204-206, 202; Gersonius et al. 2016, p.204-206, 202). The *plan* should contain measures for the short-term and options for the long-term, including measures that should be taken now to keep options open to adapt the plan (its strategies) if necessary in the future (e.g. by changing/switching of measure) (Haasnoot and Jeuken). Step 2b requires assessing the efficacy of measures and for how long a given measure will be effective (the timing of this is largely influenced by the scenario considered); and, then, exploring which other measures are available (Brugge and Bruggeman 2019, p.4-5). Through an iterative assessment of measures’ efficacy and tipping-point, it is possible to identify the most successful measures; and, once a set of measures seems adequate, it is possible to build various possible pathways (Jeuken et al. 2014, p.4). It is necessary to efficiently link measures for the short- and the long-term, thus, short-term measures must be coherently linked to long-term objectives and tasks (i.e. *must be logical in the long-term*), which implies preparing measures to keep open options for the long-term, and avoiding decisions that lead to unnecessary increases of future costs (Haegen and Wieriks 2015).  …………….  Step 2 involves: (a) the exploration of all possible or relevant measures around an ATP; and (b) the evaluation of each measure’s performance under different future scenarios (and changing conditions) and against pre-defined criteria and standards. In Step 2, it is necessary to identify measures that can be used to meet the objectives and tasks, and assess their efficiency and cost-effectiveness (costs, effects, benefits) over different scenarios (Gersonius et al. 2016, p.204-206, 202). | Step 2) Exploring and identifying possible measures, and assessing their efficacy and ATPs  (2a) This step involved the exploration of possible measures for the short-, mid- and long-term, and their delineation in a schematic map. First, the Teams identified measures for the short-term, and then they assessed if other measures, or eventual amendments / adjustments, will be needed in the mid- or long-term, and looked at the conditions under which it will be wise to switch to an alternative measure. Each Subprogramme focused on identifying which possible measures and options were required to comply with the FRM objectives at local level (Gersonius et al. 2016, p.205).  …….  (2a) In 2011/2012, each Subprogramme explored *Possible Strategies* for its region (i.e. possible measures / solutions) (DP 2014, p.132, 12; 2013, p.56, 78). The *Possible Strategies* show the playing field of measures available (DP 2012, p.43).  A *Possible Strategy* contains targets, related measures, and a *development path* (also called *solution path* or *adaptation path*) (DP 2012, p.12, 43; 2011, p.14). To develop such *adaptation paths*, the Subprogrammes analysed at what time new measures will be required, i.e. the moment of tipping-points (DP 2012, p.89).  During 2011/2012, the Subprogrammes devised (started to devise) several *development paths*. A path is generated by ‘*putting the first steps and the long-term possibilities in a logical chronological order*’, and, in this ‘*adaptive strategy*’, the steps envisioned for the short-term should leave sufficient scope for different follow-up measures, in line with ADM (DP 2012, p.61, 69). The *adaptation paths* offered a powerful way of understanding what measures can be taken, when, and how tasks and options for the long-term affect short-term decisions and measures (DP 2012, p.82).  (2a) In 2011/2012, most Subprogrammes focused on the identification of measures, by analysing how the flood protection standards could be met under CC; in addition, during this period, the DP stimulated the Subprogrammes to assess ‘*how much longer current policies and management practices were expected to suffice and when adjustments would be required’* (Werners et al. 2016) – which relates to the identification of the first ATPs (a failure of pre-existing policy / management measures). The DP acknowledged that adaptation will be necessary if the amount of change is unacceptable or if the objectives can be realized more effectively with other measures. Hence, each Team identified a range of possible measures, as broad strategic alternatives to be further studied (e.g. innovative flood defences) (Werners et al. 2016).  Moreover, in 2011/2012, the DP developed its *VGS evaluation system*, a system to assess and compare the *Strategies* (DP 2012, p.87).  During this year, research continued to *(re)identify adaptation needs* and CC effects on each region.  The DP also decided that it would create a monitoring system to provide early signals of CC and detect thresholds (Werners et al. 2016).  ………………………….  Step 2 also required assessing the effectiveness of the diverse measures under different scenarios and determining their ATPs (Zandvoort et al. 2018, p.190-191). In ADM, tipping-points correspond to points / conditions under which the current management actions / measures or policies become ‘*too expensive, technically impossible or societally unacceptable*’; the date of an ATP indicates an ‘expiration date’ at which a given measure or policy ceases to be feasible (Rhee 2012, p.18, *in* Zandvoort et al. 2018, p.190). More precisely, tipping-points indicate conditions under which a given measure or policy action ceases to be effective or acceptable (or no longer suffices) and a new measure is required. The moment of an ATP could be measured in relation to scenarios: by placing alternative measures against diverse future scenarios, their tipping-points emerge. Despite the difficulty of determining the exact moment of ATPs, the ADM approach primarily seeks to inform about the time needed to implement measures, and what are the measures available (Zandvoort et al. 2018, p.190-191).  Moreover, in the DP, *Strategies* should be made of a combination of diverse FRM measures. | Year 2 (2011/2012): Exploration of ‘Possible Strategies’ (measures/ solutions for problems and to address tasks). It resulted in the DP 2012 (for 2013). This year involved: setting guiding principles, identifying measures and possible ‘development paths’, which required analysing time-windows, lock-ins and -outs, transfer possibilities and bottlenecks (Rhee 2012, p.9; Werners et al. 2016).  …………………  In 2011/2012, the Subprogramme for Rhine Estuary-Drechtsteden started to develop its *adaptation paths* by describing under what conditions new measures will be required, i.e. tipping-points (e.g. dykes that no longer meet the standard) (DP 2012, p.89), while the Subprogrammes for the IJsselmeer Region and for Southwest Delta already presented their ‘possible development paths’ in the DP2012 (DP 2012, p.59, 69).  ………….  In 2011/2012, the RE-D Subprogramme had already defined (qualitatively) the tipping-points of the existing policy and system, and determined their timing / date in the *Delta Scenarios*; and it also started to develop ‘*interrelated packages of measures*’, i.e. strategies, which were then subjected to a ‘*stress test’* in order to identify potential vulnerabilities and ways to tackle them in the short-term (Brugge and Bruggeman 2019, p.6). These steps were undertaken in two workshops with experts and planners. Moreover, during this year, the RE-D Subprogramme explored how ADM (concept and approach) should be applied.  The RE-D Subprogramme searched for tipping-points, i.e. moments in which a measure / strategy does not work anymore, and it is necessary to shift to another (however, such moments were difficult to find or do not exist, thus, the exercise was more about ‘*spreading measures in time*’ than ‘*exploring alternative measures to shift to*’; and, in worse scenarios, measures would be implemented earlier than in ‘lighter’ scenarios (Restemeyer et al. 2017, p.932). In the case of Dordrecht, for example, to evaluate to what extent the proposed measures meet the objectives in the short, mid- and long-term, the ATPs’ method was used. The performance of measures was assessed under the most extreme *Delta Scenarios* (the *Rest* and *Steam*) (Gersonius et al. 2016 p.212). |
| *Step 3. Develop adaptation pathways and APs’ map* | This Step consists of developing (designing) ‘adaptation pathways’ (APs) (Jeuken et al. 2014, p.4-5; Haasnoot and Jeuken; Gersonius et al. 2016, p.204; Brugge and Bruggeman 2019, p.4-5). A pathway is a sequence of measures (which is assembled with measures identified in Step 2, it is made up of sequenced measures that are activated before ATPs occur) (Jeuken et al. 2014, p.4-5; Gersonius et al. 2016, p.204), thus, adaptation pathways are series of sequenced measures that provide a solution to problems (Brugge and Bruggeman 2019, p.4-5). This step involves/requires constructing / assembling several possible adaptation pathways (sequences of measures), by using the promising measures identified in Step 2 as ‘building blocks’, and then, representing such pathways in an APs’ map (Jeuken et al. 2014, p.4-5; Haasnoot and Jeuken; Zevenbergen et al. 2018). Crucial to design the pathways are the ATPs[[41]](#footnote-41) (points/ conditions under which a measure no longer meets predefined objectives): when an ATP is reached, other / additional measures are required to achieve the objectives.  The APs’ map shows the points in time for implementing measures, and the points at which a decision should be made on the potential option, and which measures are available (Deltares 2018, p.2-4; Gersonius et al. 2016).  This step requires building adaptation pathways as flexible routes to achieve the goals (Zevenbergen et al. 2018). The various pathways provide flexibility to adapt to a wide range of future changes. (Gersonius et al. 2016).  In this Step, it is necessary to design various adaptation pathways in a map (based on the assessment of the efficiency of the individual measures identified in step 2) (Gersonius et al. 2016). To design the pathways in a map, the method of Haasnoot et al. (2012, 2013) is used (*in* Brugge and Bruggeman 2019, p.4-5).  This Step also requires an assessment of the proposed pathways (strategies) on their *robustness* and *flexibility*, under different scenarios, and other criteria may be used (e.g. impacts, uncertainty, desire to keep options) (Gersonius et al. 2016, p.204-206; Zandvoort et al. 2018, p.189; Deltares 2018, p.2-3). The results of this assessment will help to select a subset of promising pathways. It is important to include inputs from different actors and seize opportunities to link different investment agendas (Gersonius et al. 2016, p.204-206). The APs’ map provides insight into potential lock-ins and options that should be kept open (scanning options to keep open) (Gersonius et al. 2016, p.204-206; Zandvoort et al. 2018, p.189).  The pathways are generated (assembled), and then assessed, which may lead back to Step 2 in an iterative way. Pathways are assessed on their effectiveness, costs, and benefits, and it is also necessary to consider each pathway’s *robustness* and *flexibility*[[42]](#footnote-42) as the goal is to elaborate *robust flexible strategies* (a *robust strategy* is one that achieves the desired objectives under diverse future scenarios, whereas flexibility is related to the possibility to adapt strategies as necessary over time – e.g. adjusting measures or implementing new measures – and still meet the objectives). The APs’ map shows *transition-points* (under which it is necessary to switch from a measure to another (or their timing in a given scenario) (Brugge and Bruggeman 2019, p.4-5; Deltares 2018, p.2-3). Several methods can be used to assess measures and pathways, e.g. cost-benefit, cost-effectiveness, and multi-criteria analyses; robustness analysis (focused on measures’ performance under diverse scenarios); feasibility analysis (focused on barriers and facilitators) (Deltares 2018).  The costs and benefits of each pathway can be presented in a scorecard (Haasnoot and Jeuken). | Step 3 (developing the Strategies, with their map of *adaptation paths*)  In this step, each Subprogramme developed a *Strategy* for FRM for its region. The *Preferential Strategies* correspond to the preferred strategies for each region. Each of the regional *Strategies* contains a map of adaptation pathways, called *‘adaptation paths’.* (Buuren et al. 2016).  To develop the *adaptation paths*, the Subprogrammes used the APs’ and ATPs’ methods. Each Team devised various possible adaptation pathways (trajectories to achieve the objectives). This required assessing the effectiveness, costs, and benefits of the various measures under different scenarios, and analysing when new measures will be needed to achieve the objectives (Zandvoort et al.2018). Thus, the design of the ‘adaptation paths’ implied the identification of tipping-points (Buuren et al. 2016). It was also necessary to *keep options open* (possibilities to alter the course) (Zandvoort et al.2018, p.191).  ……….  Developing the ‘*Promising Strategies*’  In 2012/2013, the *Possible Strategies* were further developed into *Promising Strategies* (presented in the DP2013) (DP 2013, p.56, 78).  ……  In 2012/2013, the Subprogrammes narrowed down the range of ‘*Possible Strategies*’ and further developed them into the ‘*Promising Strategies*’ (presented in the DP 2013) (DP 2013, p.56; 2014, p.132). During this year, the Subprogrammes refined and detailed further the *Possible Strategies*, which resulted in the *Promising Strategies* (with their *adaptation paths*) (DP 2013, p.56; 2012, p.80; 2011, p.14) (i.e. the *Promising Strategies* were elaborated through a filtration and refinement of the *Possible Strategies*, as less cost-effective solutions were abandoned) (DP 2012, p.80; 2014, p.132).  Moreover, in 2012/2013, the DP Team developed five *draft Delta Decisions* (issued in the DP 2013) (DP 2014, p.132).  Then, the *Promising Strategies* (and their measures) were assessed on their cost-benefit (DP 2012, p.80). The *Promising Strategies* consist of promising sets of measures (‘*promising*’ means that objectives are achieved cost-effectively, with the maximum benefits possible) (DP 2013, p.56; 94; 2012, p.80).  Each Subprogramme developed its *Promising Strategies* (for its region) in coordination with the other Subprogrammes. The *Promising Strategies* were based on the *draft Delta Decisions*, but also provided input for them (DP 2013, p.56).  In each of the *Promising Strategies*, the Subprogramme Team sought to design *development paths* (later called *adaptation paths*, i.e. pathways), which implied analysing conditions under which it is advisable to move from a measure to another ~~(ATPs)~~ and how options could be kept open to allow future transitions (DP 2012, p.81). The development of *adaptation paths* required: plotting concrete measures on a timeline, visualizing multiple options, and exploring when it will be necessary to shift from an option to another (DP 2013, p.56). A *Promising Strategy* identifies the objectives and describes concrete measures for the short-term and indicative measures for the long-term (including *options that need to be kept open*), and the resultant *development path* in a schematic schedule (DP 2012, p.81).  In specific, the year 2012/2013 consisted mainly of: 1) detailing and assessing measures and options (the Subprogrammes analysed which measures were promising to meet the objectives); 2) developing strategies, i.e. designing ‘*adaptation paths*’, and 3) appraising them (Werners et al. 2016). FRM measures were aggregated (*clustered*) and sequenced, to produce several *adaptation paths*, as alternative strategies that may be progressively implemented according to the speed of CC effects, namely of SLR. For each *Strategy*, diverse FRM measures were studied in detail. To assess the *paths* (and their respective measures) several criteria (set by DP staff) were used (e.g. flood risk reduction, cost, opportunity for other functions, feasibility, trade-offs, etc.). The possible measures were assessed on these criteria, resulting in a subset of promising measures, with which the *Promising Strategy* was built. Moreover, studies on possible site-specific measures (their qualitative effects), and quantitative modelling studies on measures’ performance and spatial effects, were conducted, whose results were essential to the cost-benefit and multi-criteria analyses.  Besides this, in 2012/2013, each Subprogramme continued to conduct research (which focussed on further exploring risks, adaptation and monitoring needs), and refine its research agenda (underlining the need to investigate adaptation options) (Werners et al. 2016).  ……….  In line with ADM, various *adaptation paths* were designed in each of the *Strategies* elaborated (DP 2012, p.81). Each *Preferential Strategy* presents a map of ‘*adaptation path(s)*’, which provides a schematic overview of the measures needed in a region. The map shows, from the current situation and moving ahead into the future, the various measures available for the short-term and possible options for the mid- and long-term (e.g. possible adjustments / adaptations that may be necessary in strategies), and indicates the conditions under which it is advisable to shift from a strategy (or measure) to another (DP 2011, p.48; Gersonius et al.2016, p.205).  A key feature of the *Preferential Strategies* (and *Delta Decisions*) is that ‘*it should be possible to take additional measures in the long term (after 2050) to address the challenges following from climatological and socioeconomic developments*’, and *‘the options for these*’ should be ready and *‘included in the adaptation paths of the Preferential Strategies*’ (DP 2014, p.148). Depending on future developments, it may be necessary to reconsider the options for long-term. To be able to take other measures after 2050, these must be considered in spatial policy, which implied the designation of ‘*spatial and policy-based reservations*’ for long-term options (DP 2014, p.148-149).  The Teams sought to maintain the possibility of taking new measures in the long-term, presenting them in the ‘*adaptation path’* of each *Preferential Strategy*. Each *path* contains options for the long-term that are intentionally kept open due to uncertain future climatic and socioeconomic conditions. These options, and the possibility of keeping them open, will be subjected to scheduled reassessments over time (Petersen and Bloemen 2015).  The DP also envisioned (short-term) measures to enable / prepare options for the long-term, e.g. ‘reserving space now’ (e.g. for dyke realignments, bypasses, or water retention), and large-scale interventions should not occur until they are inevitable (DP 2014, p.136).  The *adaptation paths* designed were assessed under different scenarios. *Strategies* had to be flexible in terms of: timing (possibility of postponing or advancing measures in time), possibility of choosing another measure or strategy, and avoidance of lock-ins. The *path*s were also assessed on their adequacy to context (physical, socioeconomic, and institutional) (Zandvoort et al. 2018, p.191).  The *Preferential Strategies* needed to be: *robust* (i.e. objectives should be achieved with the *Strategy* under all *Delta Scenarios*) and *flexible* (i.e. the Strategy, or its measures, could be sped up or slowed down and it should be possible to change to a different strategy / measure, if necessary) (DP 2013, p.94). Flexibility is, thus, provided *‘through the types of measures selected, by leaving options for adjustment or switching to other measures open for the future; in this way, measures can be adjusted to new insights and circumstances*’ (DP 2014, p.169).  The *Preferential Strategies* were developed on the basis of the four *Delta Scenarios*, but are, by nature, *adaptive*: they can be adapted *to* *slower and faster CC* than expected in the *Delta Scenarios*. The ‘*adaptive nature of the strategies’* makes it possible to act according to evolving conditions and changes, for example, *‘by accelerating or slowing down the implementation of measures*’ – e.g. the number and amount of sand replenishments per year along the coast can be adjusted to the measured SLR. Based on new knowledge and monitoring, the strategies, and / or measures within them, may be advanced or postponed, and adjusted in terms of scope and design (DP 2014, p.136).  In sum, to create ‘strategies’, the ADM process builds on the construction of pathways, and this implies scanning options to keep open, and assessing the flexibility of pathways. These aspects were patent in the development of the *Strategies* for FRM and FS of the DP (Zandvoort et al. 2018, p.189, 191). Based on the map of *adaptation paths*, the Subprogrammes looked for possibilities to link the implementation of the *Strategies* with other investment agendas of other fields (integration of FRM measures with other investments), in line with the 4^th^ principle of ADM (DP 2011, p.48; Gersonius et al.2016, p.205).  Defining the ‘*final Delta Decisions*’ and ‘*Preferential Strategies*’  In 2013/2014, the *Promising Strategies* were further developed into *Preferential Strategies* (presented in the DP2014, these are final strategies which were adopted in 2015) (DP 2013, p.56, 78).  In 2013/2014, the DP staff developed the *final Delta Decisions*, and the regional Subprogrammes developed the *Preferential Strategies*. During this period, the DP Team further elaborated the previous *draft Delta Decisions* into the final *Delta Decisions* (presented in the DP 2014); and the Subprogrammes further developed and narrowed down the *Promisi* *ng Strategies* into *Preferential Strategies* (also presented in DP2014) (DP 2014, p.132, 8; 2013, p.94, 97). Each Subprogramme has made up a *Preferential Strategy* for FRM for its region.  Most of the *Delta Decisions*, and all the *Preferential Strategies*, include ‘adaptation path(s)’ (DP 2014, p.8, 46).  The Subprogrammes elaborated *adaptation paths* for each *Preferential Strategy*, which implied defining the conditions under which changing measures is necessary (DP 2013, p.95). Each *Preferential Strategy* contains measures for the short-term and options for the long-term. Short-term measures are often linked with other planned investments or objectives of other policy fields or regions. In the development of *adaptation paths*, it was important to keep open options for long-term, i.e. possibilities to switch to other measures if future climatic or socioeconomic changes, or new knowledge, require so.[[43]](#footnote-43) The Teams also identified short-term measures needed to be able to change strategies later and analysed if it was necessary to make changes in laws or institutional practices (DP 2013, p.95).  In line with the 1^st^ principle of ADM, the DP sought to identify measures for the short- and mid-term that *agree with* the long-term tasks (DP 2013, p.102).[[44]](#footnote-44) Decisions planned for the short-term are linked to long-term tasks (DP 2017, p.22). Each *Preferential Strategy* contains short-term measures linked to options for the mid- (2050) and long-term (2100).  The *Delta Decisions* and *Preferential Strategies* were developed based on the four *Delta Scenarios* (DP2014, p.136), and with 2100 as their time-horizon.  Each *Preferential Strategy*, and its measures, was analysed under the *Delta Scenarios* (DP 2016, p.6). The diverse *Strategies* devised were subjected to a ‘robustness test’ to see which were effective under the different scenarios; the ‘*Preferential*’ *Strategies* are those that perform well even in more drastic climatic scenarios (DP 2014, p.8). Moreover, the costs of the *Preferential Strategies* were estimated, especially of the measures until 2050 (DP 2014, p.47). | Year 3 (2012/2013): Identification of ‘Promising Strategies’ (strategies that link with other agendas). It resulted in the DP 2013 (for 2014). This year involved: studying opportunities to link with other agendas and windows of opportunity and synergy; searching for / identifying the optimal sequence for measures / decisions, specifying risks and indicators, analysing existing strategies, identifying no-regrets measures and opportunities for coupling in the near-future, and anticipating measures for the short-term. This year, the DP report presented the ‘*draft Delta Decisions*’ and ‘*Promising Strategies*’ (Rhee 2012, p.9; Werners et al. 2016).  …………….  Using the measures pre-identified, the RE-D Subprogramme developed its ‘*adaptation paths*’ (pathways) for FRM for the region. This implied designing sequences of interrelated measures over time, and testing them under the *Delta Scenarios*, to see assess if future problems or ATPs arise, and identify solutions to tackle them (Brugge and Bruggeman 2019, p.6).  In the case of Dordrecht, the measures identified were assessed on their efficiency, cost-effectiveness, legitimacy, and social feasibility; and measures that may be regretted later were avoided as much as possible, and alternatives to ‘dyke strengthening’ were explored, e.g. green adaptation measures (Gersonius et al. 2016 p.212).  The APs’ map shows which measures need to be taken and when, and shows how the tasks for the long-term may influence short-term decisions (Gersonius et al. 2016 p. 211). The scheme of ‘adaptation paths’ shows the measures available for the short-term (2030) and mid-term (2050), and possible options to keep open for the long-term (up to 2100); it also includes anticipatory or preparatory) measures required to keep open options for the long-term (e.g. research studies on a particular option (Gersonius et al. 2016 p. 211).  The pathways deliver ‘flexibility’ in several ways: the strategies can be accelerated, slowed down, or adjusted, by using other options that are kept open (i.e. by shifting from one measure to another available) (Gersonius et al. 2016 p.212-213).  Though the Subprogramme worked with the four *Delta Scenarios* (translated to the regional context), the effectiveness of the strategies was only assessed in the *Steam* and *Rest*. The question of ‘which scenario should be used’ did not matter too much, because whatever scenario happens, the existing flood defence system (with its dykes and storm surge barriers) could cope with it, and it would require some improvements but not radical modifications (Restemeyer et al. 2017, p.931).  In the Dordrecht case, the ADM approach, and, in particular, the APs method, served to develop a ‘*robust flexible strategy*’ for FRM. The APs ensures flexibility, since the pathways can be accelerated, slowed down, or adjusted (through shifts from a measure to other), and options to keep open are shown. ADM was also crucial for tailoring the Strategy to the local context, its spatial and economic characteristics. The Team collected perspectives of various stakeholders and examined how measures could be linked with investments and urban projects in the area (Gersonius et al. 2016 p.12, 213, 209).  ……………..  The RE-D Subprogramme developed its ‘adaptation path’ for FRM, which indicates diverse measures and at what times these will be required (DP 2012, p.90). |
|  |  | Substep 3b (assessing the *Strategies* and their ‘*adaptation paths*’)  The Subprogrammes followed a screening process from *Possible Strategies* to *Promising Strategies*, and then to *Preferential Strategies* (preferred pathways); in this process, the *Strategies* were iteratively assessed on their cost-benefit, effectiveness in meeting the objectives, and secondary effects (on nature, shipping, etc.) (Restemeyer et al. 2017, p.933).  To assess the measures and *paths*, and, in this way, substantiate the *Preferential Strategies* (and *Delta Decisions*), the Subprogrammes used several economic assessment tools, namely cost-effectiveness analysis, cost-benefit analysis, and social cost-benefit analysis[[45]](#footnote-45), all tools provided within a set of *Delta Tools* – i.e. methods and instruments that Subprogrammes could use to substantiate their strategies, including a *Delta Model* (one- and two-dimensional models) and the *VGS Evaluation System* (which served to assess Strategies on five criteria – effectiveness on FRM targets, effectiveness on water safety targets, secondary effects and opportunities, practicability, and financing) (DP 2013, p.105, 101, 90, 84; 2014, p.137, 135; 2012, p.87). With these tools, the DP conducted a sound analysis of the *Preferential Strategies* (and *Delta Decisions*). The costs of each *Preferential Strategy* were estimated, especially of measures until 2050 (DP 2014, p.47).  It was important to assess the robustness and flexibility of the proposed strategies (pathways and their measures) against several scenarios (Gersonius et al. 2016, p.4). The Teams assessed the flexibility of the measures in a simplified way, e,g. examining if it was easy to realize measures step-by-step or adjust them to changes as these occur (DP 2012, p.88). The flexibility of strategies could be assessed using the *VGS Evaluation System*; the VGS considers the flexibility of the strategy itself and of its measures, the possibility to link agendas, the added value of the latter in terms cost reduction, practicability, and effects. Thus, the added value of flexibility was quantified in a simple way: it was incorporated in economic analyses. (DP 2012, p.90).[[46]](#footnote-46)  Each Subprogramme should assess if its Strategy was robust, flexible, feasible, efficient (cost-effective), integrated, among other aspects, using the evaluation system (DP 2013, p.95). The *Preferential Strategies* were subjected to a *robustness assessment* under worst-case events beyond the scope of the *Delta Scenarios* (e.g. a SLR greater than 85cm in 2100), which showed that the *Delta Decision on the Rhine-Meuse Delta*, the *Decision on Sand*, and all *Preferential Strategies*, were satisfactory even in worst-case events (DP 2014, p.135, 8).  Moreover, in 2013/2014, the *Delta Decisions* and *Preferential Strategies* were assessed in an *Environmental Impact Assessment*, whose results were also used to decide on the *Preferential Strategies* (based on their environmental, natural, and cultural-historical effects) (DP 2013, p.100). | Year 4 (2013/2014): Selection of ‘Preferential Strategies’ (pathways). It resulted in the DP 2014 (for 2015). This year involved: the design of the latest ‘*adaptation paths*’ (also called ‘*development paths*’), and their appraisal using CBA and CEA; the evaluation of the possible options (paths) on their *robustness* and *flexibility*, and also the comparative assessment of options (paths) with a comparison system. The DP 2014 Report presents the ‘*final Delta Decisions*’ and ‘*Preferential Strategies*’ (Rhee 2012, p.9; Werners et al. 2016). |
| *Step 4. Design an adaptive plan, and define triggers* | This step consists of: a) formulating an *adaptive plan* (an ‘action plan’), which requires selecting one or more preferred pathways as input for a *dynamic adaptive plan* (an ‘action plan’) (based on / following the evaluation of the various pathways regarding their social and economic feasibility, governance, *robustness*, *flexibility*); and b) specifying indicators (signposts) and triggers (triggers act as early warning signals for the implementation of actions or for reassessment of the plan[[47]](#footnote-47), they are critical values at which / that indicate that it is necessary to activate *contingency actions*; triggers are critical values beyond which it is necessary to make adjustments in the strategy (e.g. by shifting to another measure or pathway, i~~.e. f~~or modifying the strategies or measures) which must be monitored (Haasnoot and Jeuken; Deltares 2018, p.2-3; Gersonius et al. 2016, p.204-206; Brugge and Bruggeman 2019, p.4-5; Jeuken et al. 2014, p.4-5; Marchand and Ludwig 2014). Thus, Step 4 implies the definition of a monitoring system that defines, and collects information on, indicators and triggers (Haasnoot and Jeuken), and mechanisms to adjust the plan (its strategies) if necessary (e.g. preparatory actions such as adjustments in legislation, spatial reservations, etc.) (Deltares 2018, p.2-3; Gersonius et al. 2016, p.204-206).  ………..  Step 3 usually results in a map showing a subset of ‘promising pathways’ (including the possibilities for transferring from a pathway to another), and Step 4a implies the translation of such APs’ map into a ‘*plan of action*’, which requires deciding which pathways should be followed and kept open and analysing their implications for short-term actions (Gersonius et al. 2016, p.204-206). | Substep 4a (selecting the preferred pathways as input for an adaptive plan – an *action plan*)  The main outcome of ADM is a pathway, or a set of pathways, with which planners can schedule measures in the face of uncertain future changes, i.e. ADM ‘*leads to a composite strategy, or a set of alternative strategies with intermediate possibilities for revisions*’ (Rhee 2012, p.14, *in* Zandvoort et al. 2018, p.191). Within a Strategy, there may be: i) measures that are still part of the current strategy, ii) measures that are part of an improved strategy, iii) measures beneficial through coupling with other agendas and whose timing is optimized, and iv) measures to keep options open for a future choice (allowing different strategic directions) (Zandvoort et al. 2018, p.191).  The *Preferential Strategies* constituted guides for programming the measures to be implemented (DP 2013, p.56). The measures planned in each of the *Preferential Strategies* for the next decades are programmed – as ‘*projects*’ or ‘*implementation programmes*’ – in the *Delta Plan on FRM*, in the *Delta Plan on FS*, and in the *Delta Plan on Spatial Adaptation* (DP 2014, p.46, 19, 56). Since 2014, the annual DP report describes all *programmed measures* in such *Delta Plans* (DP 2014, p.8, 86).[[48]](#footnote-48) The Delta Plans are like ‘*action plans*’ to guide the implementation of planned measures and their incorporation in regional or local spatial planning (Marchand and Ludwig 2014, p.27).  In this step, the Teams identified decisions and measures to be taken in the short-term (including measures necessary to ensure that enough options are available in the future). The DP sought that the first measures taken were ‘no-regret measures’ (worthwhile in any scenario) (DP 2012, p.88).  In 2014, the *Delta Decisions* and *Preferential Strategies* were approved by the national government (Buuren et al. 2016). By the end of 2014, the government set down the *Delta Decisions* and *Preferential Strategies* as policy in the ‘*National Water Plan*’ (DP 2017, p.7). The *Delta Decisions* and *Preferential Strategies* form the main content of the DP from 2015 onwards.  Substep 4b (developing the monitoring and evaluation system)  ADM implies an adequate monitoring of changes and developments (namely climatic and socioeconomic developments), of new knowledge and insights, and of implemented measures (i.e. of ‘*what is being done and with what effects*’), and, based on the monitoring results, regular evaluations of the strategies followed (implemented and planned) (DP 2014, p.149; 2016, p.6). Thus, the ADM process presupposes the existence of a ‘monitoring and evaluation system’, which is necessary to: observe how climatic, physical, and socioeconomic conditions are changing, and ensure that ‘*response to these changes is an adaptive one’* (DP 2014, p.9); to allow ‘systematic learning’ and the adaptation/ adjustment of strategies, if this is prompted by changes or new insights (DP 2016, p. 6,11), namely to ‘*know in good time when the strategy has to change and when other measures that have already been prepared should be put into effect*’ (DP 2014, p.13).  After 2014, the DP started to develop its own monitoring and evaluation system, the ‘*Monitoring, Analysing, Acting* (MAA) system’, which is deemed the *engine* of ADM (DP 2016, p.6, 59; 2014, p.149). The MAA should serve to:   - monitor changes and developments (internal and external), new insights and knowledge that may emerge, and measures’ effects, and, in accordance, properly act on this information; and, regularly revaluate the Strategies and measures implemented and planned (DP 2016, p.6, 9). - collect information necessary to examine if and how the *Preferential Strategies* need to be adjusted, and to operate an adaptive approach. The MAA generates insights into the progress made and into changes or developments that may constitute a reason for adjustment (DP 2016, p.6, 9, 11). The system should keep track of developments, to allow a timely adjustment of Strategies (a change of course or measure), being indispensable to ensure an adaptive approach (DP 2016, p.59; 2017, p.13). With the MAA, the DP aims to evaluate, every year, whether any new developments demand the adjustment or fine-tuning of the *Preferential Strategies* and *Delta Plans*; and keep track of any new conditions or changes that may impact the goals and measures, and if necessary, adjust them (DP 2016, p.6-7). - monitor ‘linkage opportunities’ available (DP 2014, p.149). - foster a co-learning process in which results, lessons and successes were shared, and progresses reported (in the DP Report), the spent resources justified. MAA results should underpin the DP report (DP 2016, p.59, 11; 2017, p.13).   The MAA gives inputs to, and receives inputs from, the *Delta Decisions* and *Preferential Strategies*. MAA results must be used to update the DP on observed and forecasted effects and must be considered in any revision of programmed measures (*projects* and *implementation programmes*) (DP 2014, p.149).  The MAA focusses on three specific questions:   1. *is the implementation on schedule*, and *are we doing what we agreed* (*output*). 2. *are we achieving our goals* (*outcome*); i.e. whether FRM and FS targets are being achieved. 3. *are the preconditions still in order* (*input for follow-up*) (DP 2016, p.59, 11).   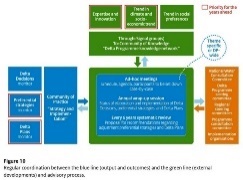The ‘*output*’ concerns the measures agreed in the *Preferential Strategies* and *Delta Plans*; the ‘*outcome’* concerns the goals defined in the *Delta Decisions* and *Preferential Strategies*; and the ‘*input for follow-up*’ concerns conditions like funding, expertise, effectiveness, collaboration, etc. (DP 2016, p.59).  To answer these questions, the MAA relies on two lines: the blue line (*output* + *outcome*), and the green line (external developments) – which act in coordination in an advisory process (DP 2016, p.59-60).  The ‘blue line’ addresses the ‘*output’*, ‘*outcome’* and ‘*input for follow-up’* questions mentioned above, for example, examines if the implementation of the *Delta Decisions*, *Preferential Strategies* and *Delta Plans* is proceeding on schedule and as planned. Within the blue line, there is a *Community of Practice* which reviews the progress on the *Preferential Strategies* and *Delta Plans* several times per year (it meets several times per year), evaluates if the objectives (for FRM, FS, and spatial adaptation) are being met, and explores ‘linkage opportunities’ (DP 2016, p.59).  The ‘green line’ identifies changes or developments that may affect the implementation or elaboration of the *Preferential Strategies* and *Delta Plans* or require their adjustment or refinement, and assesses ‘*if the DP is still on track*’. This line monitors three developments: *knowledge and innovation*; *climate and socioeconomic developments* (e.g. new climate scenarios); and *societal preferences* (DP 2016, p.60). Within the green line, there is *Signal Group* that monitors these three developments (it meets once or twice a year), and examines which external developments (e.g. pace of CC) may be a reason for revising the *Preferential Strategies* and *Delta Plans* (focussing on whether and when *Preferential Strategies* or *Delta Plans* might need adjustments) (DP 2017, p.13). The results of such analyses are then sent to, discussed with, the *Community of Knowledge* and the *DP Knowledge Network* (DP 2016, p.60).  Moreover, the *Signal Group* (in the green line) and the *Community of Practice* (in the blue line) meet once a year to connect the results of the two lines (and discuss the implementation of the *Delta Decisions*, *Preferential Strategies* and *Delta Plans*), and define recommendations (and to whom). This meeting results in recommendations about ‘progress’ (*are we still on schedule*) and ‘direction’ (*are we still on track*) (DP 2016, p.61). The DP every year reports on the progress in the elaboration and implementation of the Delta Decisions, Preferential Strategies and Delta Plans (DP 2016, p.6).  Furthermore, every six years, the DP carries out a review to analyse if the monitoring results indicate that it is necessary to maintain or adjust the course or the pace (e.g. the measures or their timing), and check ‘*whether it managed to keep up the pace and adjust its course on time*’ (DP 2016, p.6). Based on new knowledge and monitoring results, Strategies, or their measures, may be anticipated or postponed, or adjusted in terms of scope and design (DP 2014, p.136).  A 6-year review, and the eventual adaptation, of the Preferential Strategies  The *Delta Decisions* and *Preferential Strategies* are subjected to review every six years. Besides this, ‘*in the event of new insights showing that the current course will not enable achievement of the FRM, FS, and spatial adaptation goals by 2050, the Preferential Strategies may need to be adjusted*’, and ‘*the Preferential Strategies are open to annual adjustment*’ (DP 2018, p.22). If the 6-year review prompts so, the DP Commissioner may propose adjustments to the *Delta Decisions* or *Preferential Strategies*. In the 6-year review, it will also be assessed the need of keeping the envisioned long-term options open or adding new options. The results of the first 6-year review were expected to be published in DP 2020, and should explain to which extent the goals set in DP 2014 are being achieved in time (this was measured with criteria defined to gauge goal attainment) (DP 2018, p.22).  Although the DP operates in *a dynamic environment* with ongoing developments, a ‘*continuous adaptation of all the strategies to those developments is neither feasible nor necessary’*, which implied finding an appropriate ‘*rhythmicity for the adaptation of strategies and plans’* (DP 2016, p.61). Thus, it is important to assess the effects of new developments for the *Preferential Strategies*, their significance and certainty: if effects are *significant* and *certain* (and occur in the near-term), the recommendation may be that the Strategy needs to be adapted in the short-term; if effects are *significant* but *uncertain* (in terms of scope or time), the recommendation will be that further research must be conducted; in other situations, the recommendation might be to take a decision regarding a possible adjustment in the next 6-year review (DP 2016, p.61).  The MAA was developed since 2015, and it started to operate in 2017 (DP 2016, p.3, 63).  Since 2017, the MAA system has addressed four main questions (DP 2017, p.13, 8; 2018, p.19):   1. ‘*on schedule’* (*is the implementation on schedule*, are the measures being implemented within the timeframe and budget agreed. This relates to the progress of implementation, i.e. ‘output’; 2. *on track*’ (are we on the right track or do external developments constitute a reason for reconsideration of goals or measures). This relates to the attainment of goals, i.e. ‘*outcomes*’ (it implies analysing if the DP is ‘on track’ or whether there is any reason for changing the pace or course of measures or goals; if the proposed measures are sufficient); 3. ‘*integrated approach*’ (*are we addressing the taskings in an integrated manner*); 4. ‘*participation*’ (*are governments, businesses, NGO’s, and residents involved on a wide scale, where such is called for*).   With these 4 questions in mind, progress is reviewed in the *Delta Decisions* and *Preferential Strategies*. The *Community of Practice* focuses on questions 1, 3 and 4, while the *Signal Group* focuses on question 2 (DP 2017, p.13).  Indicators to be monitored: in 2017/2018, the *Signal Group* defined 8 *indicators* to be monitored in order to detect signals that may prompt an adjustment of the *Preferential Strategies* and developments that could jeopardise the attainment of goals, namely: SRL, extreme river discharges, land use, etc. (DP 2018, p.111; 20).[[49]](#footnote-49)  An indicator might require: more targeted research; monitoring and scheduled evaluations; analysis of consequences; or, if necessary, the adaptation of measures or policy amendment. Thus, indicators / signals must be identified in a *sufficiently timely* manner (given *‘the time required for plan preparation, design, and construction’*) and in a *reliable manner* (which detects *‘a significant trend or warning signal from an observation series that often features a great deal of noise from annual fluctuations*’) (DP 2018, p.111).  In addition, the *Signal Group* and the DP also meet at least twice a year (DP 2018, p.111-112):   - In January of year X, the *Signal Group* and the DP discuss which developments constitute a reason for adapting the *Preferential Strategies*. The *Signal Group* presents generic developments (e.g. accelerated SLR), while the regional Subprogrammes present theme- and region-specific developments (e.g. changes in water demand). The conclusions are submitted to the *DP Consultation Committee* that meets in March and chooses which developments deserve further elaboration; in addition, knowledge institutes and managers analyse such developments and determine where adaptations are required in which themes or regions (this phase occurs from March to August). These developments are then reported in the DP Report of year X (developed for the year X + 1). - In September of year X, the *Signal Group* and the DP discuss the topics (developments already elaborated) that require adaptation of the current Strategies, and if such adaptation must be made in that year or can wait until the next 6-year review. Their conclusions are submitted to the *Consultation Committee*, and, if necessary, to the *Steering Group*, to see which elements of which Strategies need to be adapted. These adaptations are implemented by the DP offices. The debate and decision about adaptations occur between October of year X and February of year X + 1 (included), and the adaptations are reported in the Report elaborated in the year X + 1 (for year X + 2).   This annual process generates a progress report: the DP annual report. Thus, a ‘signal / indicator’ may activate a multi-year process, which may require a ‘policy evaluation’ and lead to a decision to adapt *Decisions* or *Strategies* (DP 2018, p.112). | |
| *Step 5. Implement the plan* | This Step consists of implementing the plan’s measures, namely the selected measures for the short-term and measures necessary to keep open options that may be needed in the future (Deltares 2018, p.2-3; Jeuken et al. 2014). | The DP presented the *Delta Decisions* and *Preferential Strategies* in the 2014 Report (DP 2013, p.112, 125), and in late 2014, the Central Government embedded the national policies resultant from the *Delta Decisions* and *Preferential Strategies* in an interim amendment of the *National Water Plan 2010-2015*, and, in late 2015, in the *National Water Plan 2016-2021* (DP 2016, p.64; 2014, p.8).  After 2014, the DP entered in a new phase focused on the detailed elaboration and implementation of the *Delta Decisions* and *Preferential Strategies*, and the observation of how the climatic, physical and societal conditions are changing, and ensuring the continuation of the adaptive approach (DP 2014 p.9, 144; 150; 2013, p.114; Alphen 2015; Bloemen et al. 2018; Zevenbergen et al. 2018).  The DP Commissioner has supervised the implementation of the measures by the Water Boards, Provinces, Municipalities, disaster management organizations, and Rijkswaterstaat (the National Authority for Water Management and Public Works) (Alphen 2015) and has submitted the annual DP report (documenting the progress of the proposed measures) (DP 2013, p.112).  By 2016, most Provinces had incorporated the *Delta Decisions* and *Preferential Strategies* into their policy (namely in their *framework visions* or *environmental visions*, for instance in the ‘*Spatial Planning and Mobility Vision*’ of Zuid-Holland Province which includes Rotterdam); while the Water Boards anchored policies resultant from the *Delta Decisions* and *Preferential Strategies* in their new *Water Management Plans* (DP 2016, p.65).  ADM required the DP to deal with new insights and changes as part of the implementation of the *Delta Decisions* and *Preferential Strategies*. This implied the creation of ‘*a properly embedded monitoring and evaluation programme*’ and ‘*a steering model that is in line with the adaptive approach*’ (DP 2014, p.148).  Moreover, ADM implied an ‘*adaptive implementation*’ of the measures planned in the *Preferential Strategies*, an ongoing search for more efficient strategies (or measures), a combined programming of projects in the fields of FRM, FS and spatial adaptation; the consideration of expected future conditions in the design of measures (*future-oriented dimensioning*); and ‘*targeted monitoring and periodically adjusting*’ (DP 2014, p.145). In the programming and implementation of measures, the DP has sought to link the measures of FRM, FS and space, and seize opportunities, e.g. by combining dyke improvements with spatial adaptation measures (DP 2014, p.9).  Alongside regular maintenance of flood defences, the main FRM tasks for the next decades (up to 2050) involve: applying the new flood protection standards; improving the primary flood defence systems that fail the Statutory Assessments (through the *Flood Protection Programme HWBP*) (DP 2013, p.124).  With the publication of the DP 2014, the phase of ‘strategy development’ formally ended, and the phase of further elaboration and implementation of the Strategies began; some responsibilities were transferred to ministries and project-execution organizations; and regional and local governments were charged to coordinate activities in their region. ADM and ‘thinking in APs’ were important in the phase of ‘strategy development’, but should also be preserved in the phase of implementation: ‘*the necessity to maintain the adaptive character of the DP in the implementation phase was stressed by several organizations including research institutes’* (Bloemen et al. 2018, p.13). Gradually, the emphasis shifted from the development of *adaptive strategies* towards their implementation in practice. The effectiveness of ADM must also be proved in this implementation phase, namely by monitoring of external drivers, regularly evaluating the effectiveness of measures applied and proposed, and assessing if it is necessary an anticipation or delay of measures, or a transition to another strategy (Zevenbergen et al. 2018). Adaptivity should be conserved in the next phase (Bloemen et al. 2018). | |
| *Step 6. Monitor, reassess the plan accordingly, and, if necessary, adjust it (its strategies/ measures)* | This step consists of monitoring and evaluating external changes, implemented measures, and progresses in knowledge, and regular reassessing the plan, and, if necessary, reviewing it (Haasnoot and Jeuken). It requires a monitoring and evaluation system to keep track of climatic, physical and socioeconomic developments, and assess if and when it is necessary to review the plan (adjusting or changing its strategies or measures, e.g. shifting from a measure to another) (Deltares 2018, p.2-3, 5). Step 6 is essential to allow a truly adaptive planning and management. Variables that induce to triggers must be monitored (Jeuken et al. 2014, p. 4).  ………  In this step, the monitoring and evaluation system is conducted: it tracks climatic and socioeconomic changes relevant for adapting the plan’s strategies or measures, e.g. for anticipating / delaying the implementation of measures. For example, if SLR unfolds at a faster rate than expected and a tipping-point is expected to be reached earlier, it may be necessary to implement a measure earlier than projected (if SLR evolves slower, then, the measure can be delayed). The monitoring system indicates the rate and direction of risks and implications for adjusting the plan. It allows for adjusting the plan - its measures or their timing (Brugge and Bruggeman 2019, p.4-5). This adjustment implies adaptive capacity in institutions involved. | Step 6 (carrying the MAA system)  Since 2015, the DP has reported every year on the elaboration and implementation of the *Delta Decisions*, *Preferential Strategies* and *Delta Plans*, and has assessed (every year) if their progress is on schedule, whether any new developments demand their adjustment or fine-tuning (the annual report contains the results of the prior year and a look ahead). It has also monitored new developments / changes that may affect the goals and strategies defined, and, if necessary, it adjusts them (DP 2016, p.6-7).  The MAA system has helped to get a clear picture of the annual status of the DP, analyse the progress made, the state of affairs, and the need for strategy adjustments (DP 2017, p.13; 2016, p.6; 2018, p.19). In this sense, it underpins the adaptive approach of the DP.  Since 2016, using the MAA system, the DP has monitored external developments (e.g. climate change effects) that may affect the pace or direction of the *Preferential Strategies*, and evaluated whether the DP is ‘on track’ or whether there is any reason to adjust the course or pace, or reconsider targets or measures (e.g. whether external developments demand a switch of Strategy / measures, or a modification in their timing). The DP also specified the design of the *Preferential Strategies*: it defined interim benchmarks for 2020-2050 to assess if the Strategies need to be adjusted (DP 2017, p.8, 17). From 2016 onwards, the DP annual reports have presented an overview of the progress made (by the DP and Subprogrammes) in implementing the *Preferential Strategies* *and Delta Plans*, based on the MAA: information is organized according to the four questions previously mentioned (*on schedule*, *on track*, *integrated approach*, and *participation*) (DP 2017, p.13, 15). In 2016-2017, the DP was, in general, ‘well on track’: most of the measures agreed were being implemented as planned. In 2017, the implementation of *Delta Plans* was ‘*on schedule’* (the measures envisioned in them have been mostly accomplished within the timeframe and budget agreed).  Moreover, in 2016-2017, the Signal Group monitored external developments (climatic and socioeconomic changes) that may affect the pace or direction of the Preferential Strategies, and examined whether external developments demanded an adjustment of the course (DP 2017, p.8, 17).  In 2017-2018, most of the measures scheduled in the DP 2014 were well ‘*on schedule*’(DP 2018, p.15). The Signal Group continued to monitor developments relevant for deciding the future course (DP 2018, p.14, 20).  The first 6-year review (in 2020) assessed the need of keeping the envisioned long-term options open or adding new options (DP 2018, p.22-23).  ADM, and its ongoing nature, are reflected in the annual update of the DP (every year a new DP report is issued, including a reprogramming of measures, and submitted to the Parliament) (DP 2014, p.95; Bloemen et al. 2018). In this process, monitoring and research, and the design and implementation phase, can be conducted at the same time and influence each other (DP 2014, p.149). | |

**Table 2.** ADM process in the DP, including the development of *Strategies*. Source: own elaboration, based on DP 2011, 2012, 2013, 2014, 2016, etc.

# Note 3: Key-elements of the Adaptive Planning approach of the TE2100

As mentioned, the TE2100 Project has developed and applied a *Dynamic Adaptive Planning approach* (also known as *Iterative Risk Management* or *Managed Adaptive approach*) based on concept of *Dynamic Robustness* (Ranger et al. 2013, p.249-250, 254, 257-258; Ramsbottom and Sheppard 2017, p.1, 18). This *Dynamic Adaptive Planning approach* aimed at building a *dynamic adaptive plan* / *strategy* that was both *robust* and ‘*flexible*’ (Ranger et al. 239, 249, 254, 233). *Robust* means that the plan ‘*performs adequately well against a set of decision criteria under a wide range of possible future states of the world’* (Ranger et al.2013, p.247). *Flexible* means that the plan (its strategies, measures, and their timing) can be changed over time as conditions change or new knowledge arises, thus, it is ‘*adaptable*’ / adaptive (EA 2012, p.35-38; Ranger et al. 2013, p.233, 247, 249, 239).

As a constituent part of its *Dynamic Adaptive Planning approach*, the TE2100 developed a new methodological approach to construct a *dynamic adaptive plan*: the *Adaptation Pathways approach*, also called *Route-map approach* or *Decision Pathways approach* (EA 2009b, p.200, 3-4; Ranger et al. 2013, p.249; Reeder and Ranger 2011, p.5, 8).

This Section identifies the elements of the TE2100’s *Dynamic Adaptive Planning approach* that were essential to develop a *dynamic adaptive plan* and explains how these elements were developed in practice. Drawing on the previous analysis and on prior studies[[50]](#footnote-50), it was possible to identify (derive) the main elements of this Adaptive Planning approach that were essential to design and implement a *dynamic adaptive plan,* as well as the fundamental ‘ingredients’ that make this Plan a *robust adaptable plan*. These elements and ingredients are summarized in **Table A.2**.

## 3.1. Deriving the key-elements of the TE2100’s ‘Dynamic Adaptive Planning approach’ essential to develop a *dynamic adaptive plan*

#### Key-element 1

Since an early phase, the TE2100 Project Team decided to incorporate the uncertainties inherent to CC projections into the planning and decision-making process (EA 2009A, p.71). The existence of deep uncertainties about future effects of CC and SLR on flood risk in the TE led the EA Team to commission research studies to the *Met Office Hadley Centre* and other organizations, to better grasp the uncertainties around future CC projections, and analyse the current flood risk in the TE (considering the existent defence system) and how it might change in the future (EA 2012, p.1, 6, 28; EA 2009b; EA 2009A, p.11). In specific, research was commissioned to better understand CC effects on relative SLR, storm surge and river flows, and the plausible future increases in extreme water levels in the TE, and to produce a range of future scenarios of water level rise (WLR) until 2100 (EA 2012, p.28; EA 2009A, p.71).

To deal with uncertainty (namely in the phase of *Options development*), the TE2100 Team has developed diverse climatic future scenarios which covered a wide range of plausible future conditions (EA 2009A, p.11). Therefore, the TE2100 Project has considered and worked with a wide range of plausible future scenarios of water level rise (WLR) until 2100, rather than a single ‘*most probable*’ scenario (Jeuken et al. 2014, p.10, 11; Jeuken and Reeder 2011, p.6).

The existence of uncertainties around future CC and SLR effects led to the consideration of various plausible scenarios with a long temporal horizon (2100 and beyond) (Jeuken et al. 2014, p.14; Jeuken and Reeder 2011, p.6). WLR scenarios for the TE were generated based on GCMs, RCMs, climate projections, modelling studies, past observation, expert judgement, and other sources (Ranger et al. 2013, p.233, 239, 258). The main climate parameters considered in these scenarios were SLR, storm surges and river discharges, although projections showed no trends in river discharges, thus the work focused on SLR and storm surge (Jeuken et al. 2014, p.11, 14; Jeuken and Reeder 2011, p.6). The range of scenarios included ‘high-impact / low-probability scenarios’ and moderate scenarios, and, later in the Project, it was identified a ‘*most likely* scenario’ (the *Defra06 central*) (Jeuken et al. 2014, p.10, 11). In addition, four socioeconomic scenarios were specifically developed for the TE (Jeuken et al. 2014, p.13).

Initially, an extreme SLR scenario, the *High++*, was used, as an upper-bound figure, to support and inform the exploration of possible measures and the development of the *High-Level Options* (*HLOs* / pathways) – it served for exploratory purposes – and, later other (four) scenarios were used for refining and detailing the *HLOs* (into *Detailed Options*) and for appraising them. Two climate scenarios (*Defra06 central* and *High+*) and two socioeconomic scenarios (A and D) were used to appraise the *Options*, and, in this way, examine their long-term robustness (Jeuken et al. 2014, p.10, 14).

The range of generated scenarios was used to grasp how flood risk might change in future, but also to identify of different measures and set long-term adaptation requirements (for the *HLOs*), and to develop the pathways (*HLOs* / *Options*) and appraise them (Ramsbottom et and Sheppard 2017, p.7, 9).

The use of a wide range of plausible future scenarios of WLR, namely to test measures and design pathways and appraise them, has been deemed one of the main innovations of the TE2100 (Ranger et al. 2013, p.233, 239, 258; Reeder and Ranger 2011, p.7), but it was also a crucial element to develop a *long-term* *adaptive plan* – both *robust* and *adaptable* to an uncertain future and changing risk. From the outset, the Plan was devised to cope with change (namely a changing climate), hence, the Team sought to gauge ‘*how much adaptation will be needed for different scenarios*’ (EA 2012, p.28). Therefore, working with a wide range of plausible future scenarios, to assess possible measures and pathways, can be deemed a key-element of the TE2100’s *Dynamic Adaptive Planning approach* that was essential to craft and operationalize the *adaptive plan*.

#### Key-element 2

From an early phase, the Project Team decided to incorporate the uncertainties inherent to the projections of CC into the planning and decision-making process (EA 2009A, p.71). Through the EU-funded partnership ESPACE 33 and further developments/advancement within the TE2100 Project, the Team developed *techniques of scenario-neutral analysis* that involved identifying *critical thresholds* in the FRM system (EA 2009A, p.71), as well as the *Decision Pathways approach* (EA 2009b, p.199-200, 3).

The TE2100 Team has identified *critical thresholds* in terms of/for the sensitivity and vulnerability of the FRM system to flood risk and its impacts, namely limits that would be disruptive for the flood defence system and which may occur between the present and the upper-bound figure / scenario of SLR considered (the *High++*) and which would imply new measures (e.g. modification to existing defences) (Ranger et al. 2013, p. 241, 242, 255; Reeder and Ranger 2011, p.6; Bloemen et al. 2018, p.7, 8; Ramsbottom and Sheppard 2017, p.4; EA 2009A, p.149-151).

Overall, in the TE2100, critical thresholds correspond to conditions under which the FRM system with its current FRM measures or policies, or other measures, *fail* – i.e. no longer meet the objectives – and new measures are needed (the driver for taking action is not CC *per se*, but mainly being unable to meet the objectives) (Jeuken and Reeder 2011, p.4; Bloemen et al. 2018, p.7, 8; Jeuken et al. 2014, p.17).

The Team sought to identify key thresholds (major change points) in terms of vulnerability and sensitivity of the FRM system, including critical levels of SLR (the level of SLR at which existing defences fail), limits to adapting the existing defences, and any limits to adaptation (e.g. level of SLR that represents a critical engineering limit to adapting defences) (Reeder and Ranger 2011, p.6, 5).

The Team focused on examining *what* (amount of) *change can the system handle before it runs into trouble*, and, specifically, what increase(s) in SLR and peak river discharge could cause a technical failure or overtopping of the defence system, including all its embankments, floodwalls and the TB (Jeuken and Reeder 2011, p.4).

The analysis of thresholds (*threshold analysis*) was the starting point (the *first step and core*) of the following planning process (namely for the exploration of possible measures and pathways) (EA 2009b, p.3; Jeuken and Reeder 2011, p.3, 4; Reeder and Ranger 2011, p.5). It helped to identify limits for the existing FRM system and its current measures and signalize conditions under which new measures will be needed (Reeder and Ranger 2011, p.5).

The thresholds identified were also used to define *acceptable flood probabilities* (e.g. acceptable return periods for flood events) which were translated into *design criteria* for flood defences (e.g. into diverse FRM policies) (Jeuken et al. 2014, p.17; Sayers et al. 2012, 282, 285). To identify thresholds, the Team examined physical and technical limits of the defence system, quantified its overcapacity, and, based on these and on projections, it defined *design criteria* (Jeuken and Reeder p.6; Ramsbottom et and Sheppard 2017, p.4). The Team analysed *what are the thresholds where interventions will be needed* (for instance, flood probability will increase until a certain threshold level is reached – e.g. a maximum acceptable flood probability – and new measures will be needed to reduce such probability).[[51]](#footnote-51) Modelling of future scenarios was undertaken *to determine the* (timing of) *thresholds when measures are needed to keep the flood probability within acceptable limits*, and investigate possible measures to manage flood risk (Ramsbottom et and Sheppard 2017, p.4,5).

The identification of thresholds (and the determination of their moment/timing in different scenarios) informed the development of *Options* (pathways) (EA 2009b, p.3, 10). The thresholds were crucial (served) to sequence and assemble the possible measures into ‘*packages* of sequenced measures’ (i.e. to design the pathways); the final route-map shows the thresholds at which it is necessary to switch from a measure to another. In this way, the identification of thresholds contributed to safeguard and enhance the *dynamic robustness* (flexibility) and adaptability of the Plan (ibid).

The method for identifying thresholds – also called *threshold analysis*, *threshold approach*, or *scenario-neutral analysis approach* – is deemed one of the main successes of the TE2100, as it allowed a more ‘scenario-neutral’ approach (EA 2009b, p.3, 10; Lowe et al. 2009, p.86; EA 2009A, p.71). It made the APs / Route-map approach itself more *scenario neutral* because decisions did not require information about the likelihood of different scenarios (Ranger et al. 2013, p.254; Reeder and Ranger 2011, p.9). Furthermore, drawing on the prior analysis, it can be induced that the identification of critical thresholds – as conditions under which the current or a proposed measure ceases to be effective (no longer meets the objectives), or the current system performs unacceptably, and a new measure is needed – is another element that was fundamental to design the TE2100’s Plan as a *robust adaptive plan* and essential to allow and streamline its adaptations over time.

#### Key-element 3

Given the uncertainties around future changes in the TE and the high stakes involved in developing a FRM plan up to 2100, the TE2100 Team sought to develop a *dynamic adaptive plan* that was both *robust* and *flexible* or *adaptable* (EA 2012, p.36, 39, 1. 29; EA 2009A, p.11; Ranger et al. 2013, p.233, 239, 247, 249-250, 254, 257-258; Ramsbottom and Sheppard 2017, p.1, 18).

In the light of the TE2100’s *Dynamic Adaptive Planning approach*, a *dynamic adaptive plan / strategy* should be: *robust* (perform well under a wide range of plausible futures), and flexible or *adaptive* (the Plan and its strategies, their measures, or their timing, can be changed over time as changes or new information arise) (EA 2012, p.35-38, 39, 1. 29; EA 2009A, p.11; Ranger et al. 2013, p.233, 239, 247, 249-250, 254, 257-258; Ramsbottom and Sheppard 2017, p.1, 18).[[52]](#footnote-52) The TE2100’s *Dynamic Adaptive Planning approach* aimed at building a *dynamic adaptive plan* that was both robust and flexible. The Team sought that the Plan was *as robust as possible* (namely to uncertainties about future water levels) and *adaptable to change* (EA 2012, p.35-38; Ranger et al. 2013, p.239, 254).

To develop a *dynamic robust adaptive plan*, the TE2100 devised and applied a new methodological approach: the *Adaptation Pathways approach*, initially called *Route-map* or *Decision Pathways approach*, which belongs itself to the recent family of Adaptive Planning approaches (EA 2009b, p.200, 3-4; Ranger et al. 2013, p.249; Reeder and Ranger 2011, p.5, 8).

In the TE2100’s *Dynamic Adaptive Planning approach*, and particularly in the APs approach, the Plan and its strategies *are designed to be adjusted over time as more is learnt about the future* or as changes occur (Ranger et al. 2013, p.249). Moreover, in the TE2100’s APs approach (characteristics):

- measures are implemented iteratively over time to maintain/keep risk below target/acceptable levels cost-effectively (i.e. to continuously manage risk), while keeping open alternatives (options) to manage future risk (keeping options open for the future), thus, maintaining *flexibility*, instead of designing an optimal solution for a particular level of risk (Ranger et al. 2013, 249, 247, 239, 233, 258; Reeder and Ranger 2011, p.13, 4; Jeuken and Reeder 2011, p.4).
- the timing of new measures, and the measures themselves, can be changed over time, in this way, *flexibility is built into the long-term strategy itself* (Ranger et al. 2013, p.249). Moreover, the approach allows for switching between different measures or pathways in the future (Jeuken et al. 2014, p.10).

In the TE2100 Plan, each pathway – denominated *High-Level Option* (*HLO*) or *Option* – is a ‘*package*’ of measures sequenced and implemented over time (*package* of sequenced measures that can be implemented over time) (Ranger et al. 2013, p.249; Reeder and Ranger 2011, p.8; Jeuken and Reeder 2011, p.4; EA 2009b, p.3-4)*.* More specifically, each pathway consists of (is made up of) a *number* (set) of measures (individual measures and / or *portfolios*) that are implemented in sequence over time to manage flood risk in a staged way (Bloemen et al. 2018, p.8, 7; Ramsbottom and Sheppard 2017, p.2, 5).

*An Option is a number of portfolios which, when implemented in sequence, provide a complete FRM solution for the next 100 years* (for the duration of the FRM plan); a *Portfolio* is *a number of responses which, when combined together, provide a complete FRM solution for a particular increase in sea level and/or fluvial flow; a Response is an individual FRM measure, e.g. a barrier, a length of raised defense, or an emergency plan for a community* (EA 2012b – SEA – Strategic Environmental Asssessment*, in* Bloemen et al. 2018, p.8; Ramsbottom and Sheppard 2017, p.2, 5; EA 2009A, p.138).

Overall, the Team developed an *adaptable Plan* made up of a set of *Options*, each *Option* made up *with interventions sequenced through the century* (EA 2009A, p.73, xv)**.** E*ach Option consists of a sequence of portfolios of responses* (EA 2009A, p.152). Hence, each *Option* is *made up of sequenced portfolios over time* (each portfolio reduces flood risk so that it stays below the target level), as illustrated in the scheme of the *Managed Adaptive approach* of the TE2100 (EA 2009A, p.139, 149-150, xi-xii). *Each Option* *includes* (comprises) *a sequence of interventions* or *portfolios* over the next 100 years; and the *dates* (points) *at which one portfolio changes to another portfolio depend on key thresholds for FRM assumed* (EA 2009A, p.277, 297, 149-150; 262).

Five HLOs/pathways were drawn up in the route-map of the TE2100 (EA 2009A, p.149). The route-map shows a **set / range / series / suite of** five different possible pathways (*HLOs*) (EA 2009A, p.149). By using the APs approach, the Team has developed ***a set of Options***, i.e. ***a range of options to manage flood risk*** (a range of different FRM options) (EA 2009A, p.x, 8, 72, 142, 149, 259, 279, 290; EA 2012, p.32, 34, 6, 32; 2009b, p.2; Bloemen et al. 2018, p.7). The Team has developed ***a series of generic Estuary-wide options*** *which can successfully manage flood risk through the century*; the Plan sets out ***the range of options*** *which can manage a change in water levels through this century* (EA 2012, p.34, 6). The Team could identify ***a series of adaptation pathways***, a route-map, that are appropriate to cope with the plausible range of climatic changes that could be seen by 2100 (Reeder and Ranger 2011, p.5). The TE2100 Plan has ***a set of options*** *(adaptation pathways)* (Bloemen et al. 2018, p.7).

Using the APs approach, the TE2100 Team has designed different possible pathways (the five *HLOs*, later refined and named *Options*) (Jeuken and Reeder 2011, p.4), where each pathway is itself a *robust flexible set of measures*. The APs approach was used for identifying possible (FRM/adaptation) measures and exploring their timing and sequencing over time, and, in this way, designing several different possible pathways of adaptation, which were then tested on their suitability under different scenarios and refined (Ranger et al. 2013, p.249). In this way, the APs allowed the design of a route-map of *Options* (pathways), i.e. a route-map showing a *robust flexible set* of *Options*.

The APs approach involved sequencing the implementation of diverse measures in a way that allows the FRM system to be adapted to changes over time (e.g. SLR), and at the same time keeping alternatives (options) open to cope with plausible different future climate scenarios (Reeder and Ranger 2011, p.3). The approach focused on *sequencing a suite of measures in order to cost-effectively manage current risk while maintaining the flexibility to cope with the range of possible future SLR* (Reeder and Ranger 2011, p.13).

The design of pathways (*Options*) required a *threshold analysis* – i.e. identifying points under which a measure no longer meets the predefined decision-criteria and it is necessary to either take a new measure or switch to other pathway; and, more broadly, examining under what conditions the Plan might fail and preparing actions to safeguard against this (Ranger et al. 2013, p.250).

The *HLOs*/pathways were designed to cope with the estimated plausible rises in water level until 2100 up to a 4,2m SLR (providing a FRM solution throughout the Plan’s life) (Bloemen et al. 2018, p.7). Together, the *HLOs* were designed to span (cover) the estimated range of plausible future water level rises in the TE until 2100 (up to a 4,2m) (Jeuken and Reeder 2011, p.4; Ranger et al. 2013, p.250; Reeder and Ranger 2011, p.8). Each pathway (*HLO*) is able to cope with a certain level of water level rise (each pathway was tested under various scenarios to assess its robustness) (Jeuken and Reeder 2011, p.4).

The development of *robust flexible strategies* with the APs method is considered one of the main elements of the TE2100’s *Dynamic Adaptive Planning approach* (Jeuken and Reeder 2011, p.2, 4). The TE2100 used the APs approach as a method of designing *robust flexible strategies*, where *robustness* to uncertain future changes and *flexibility* could be ‘built-in’ in the adaptation strategy itself (Jeuken and Reeder 2011, p.4, 2; Reeder and Ranger 2011, p.3, 8, 11). Thus, it can be argued/postulated that using the APs approach was crucial for designing *robust flexible strategies* as pathways (also named *flexible adaptable Options*). Using the APs method, a series of pathways – a robust flexible set of pathways/*Options* – could be designed, where each pathway is itself a *robust flexible set of measures*.

With the APs approach, it was possible to make the plan more robust to deep uncertainties about future climate; the APs offered a way of incorporating robustness to CC uncertainties into the adaptation Plan itself and its strategies / *Options* (to assess the robustness of each pathway, the Team tested its suitability under different scenarios) (Jeuken and Reeder 2011, p.4). Importantly, all the *HLOs* will remain under consideration, and a decision between them will only be made in the future when there is better information as a result of continued monitoring and new insights into SLR (Ranger et al. 2013, p.251).

Overall, the TE2100 Team sought to *build-in* (incorporate) *dynamic robustness*, *flexibility* and *adaptability*, into the general Plan and its strategies, in order to deal with uncertain future changes (Ranger et al. 2013, p.249), which was done through the APs approach. By using the APs, the Team sought to safeguard and enhance the *robustness* and *flexibility* of the Plan (Jeuken et al. 2014, p.10), (i.e. in the TE2100, the *robustness* and *flexibility* of the Plan were built-in and safeguarded by using the APs method). The APs /helped to incorporate and enhance *robustness* to uncertain future changes and *flexibility* to cope with change, into the Plan and its *strategies* (pathways).

In the TE2100’s APs approach, *robustness* and *flexibility* were built-in / ensured in the following ways:

- Through the ability to change between measures and between *Options* (i.e. pathways’ flexibility). In the TE2100’s route-map, each *HLO*/pathway is *flexible* as it is, e.g., possible to move from a measure to another, and it is also possible to move from a *HLO* to another one, depending on the rate of change experienced (EA 2009b, p.4; Reeder and Ranger 2011, p.9; Lowe et al. 2009, p.89; Jeuken and Reeder 2011, p.4). The APs approach allows the switching between different measures, but also between different pathways (*HLOs*) (Jeuken and Reeder 2011, p.4; Jeuken et al. 2014, p.18).[[53]](#footnote-53) More precisely, the APs approach allows switching from a measure to a new one (within the same pathway) or switching to a different pathway, but also adjusting (adapting) the pathways, their measures, or their timing, if necessary. As mentioned, in the TE2100, strategies (*Options* / pathways) were designed to be adjustable as changes occur or new information arises (Ranger et al. 2013, p. 249, 233, 227). The APs approach carry the ability to change between measures and/or *Options*, for instance: if the observed rate of change in an indicator differs significantly from the expected rate, it may be necessary to switch to an alternative *Option* that can cope better with the new conditions (EA 2012, p.35, 36, 38-39; EA 2009A, p.xvi, 267, 278-279).[[54]](#footnote-54) By allowing switching to another measure or pathway (i.e. shifting of measure or pathway) or altering the measures themselves, or their timing, the APs approach contributed to increase the overall Plan’s flexibility.

As noted in the Technical Report, *a* *key feature of the Options is that they can be adapted to respond to change* (EA 2009A, p.11): each *HLO* can be adapted (according) to the rate of change (water level rise) that is experienced, e.g.: in *HLO1*, a 0,20m SLR implies raising small defences; a 0,6m SLR implies over-rotating the TB and restoring interim defences; a 0,9m SLR implies improving the TB and raising downstream defences (EA 2009b, p.3; 2009a, p.11; Reeder and Ranger 2011, p.9; Ranger et al. 2013, p.250; Lowe et al. 2009, p.89; Jeuken and Reeder 2011, p.5). A key characteristic of the *Options* is that they can be adapted to address changes over time, for instance: *if the amount of change is relatively small, the number of interventions required during the century would also be small*, but *if change occurred more rapidly, the frequency and magnitude of interventions would increase* (EA 2009A, p.11).

- By changing the timing of new measures/interventions (by allowing adjusting the timing for new measures), or adjusting measures or Options themselves. The Plan allows changing the timing of new interventions (e.g. if rates of change increase, interventions will be brought forward; if they are slower, then interventions will be delayed) (EA 2012, p.35, 36, 38-39; EA 2009A, p.xvi, 267, 278-279). The timing of new measures, and measures themselves, can be changed (adjusted) over time, which builds-in and enhances *flexibility* of the long-term strategy (Ranger et al. 2013, p.249). In the APs approach, it is possible to adjust the timing for implementing a measure as new information emerges (Jeuken et al. 2014, p.18), and it also is possible to adjust (adapt) a given measure, or switch to other measure, if necessary. The Plan itself explains how planned measures can be adapted, or new measures adopted, if critical indicators (e.g. SLR rate) change significantly or differ from the estimated in the Plan (Jeuken et al. 2014, p.18, 6). The TE2100 Plan considers the possibility of altering the timing for new measures or measures themselves, e.g. a measure can be postponed or advanced in time according to changes observed. The Plan not only allows adjusting the timing of interventions (actions), but also of decisions (*implementation-points* and *decision-points*, respectively). The Plan’s reviews must be informed by monitoring outputs, and if a relevant change happens in one or more indicators, it may be necessary to anticipate or postpone a decision or a measure (certain values in the indicators will act as triggers of changes, e.g. a rapid change in an indicator may trigger an earlier decision) (EA 2012, p.35, 36, 38-39).[[55]](#footnote-55) Thus, the date of an *implementation-point* and *decision-point* for a given measure or *portfolio* can be adjusted. As mentioned in the Technical Report, *the Estuary-wide Options can be adapted to different amounts of future change by changing the dates when the ‘portfolios’ are implemented; and, if at some point in the future, physical, environmental or socio-economic changes in the TE are outside the limits which can be accommodated by a chosen Option, an alternative Option will be implemented* (EA 2009A, p.297). Importantly, the preferred Options and the alternative Options must be re-appraised, using the updated best estimates of future change, which may lead to a change in the selected Option. Moreover, while alternative Options are included in the Plan, it is necessary to consider whether there are any other alternative Options whenever the Options are reviewed (EA 2009A, p.280). The final Plan’s recommendations are based on 2009 Government guidance on CC; however, if CC predictions (or other pressures) worsen, then the date of an intervention might be changed – this was allowed for in the Plan (EA 2009A, p.xiii). Moreover, if the10-yearly review of the Plan identifies that a different *Option* is then preferred, this is possible as long as the final decision is made with sufficient lead-in time to implement it (e.g. an act of parliament may be required); whatever the current *end-of-the-century Option* is recommended, it was important to have a preferred Plan (*preferred Option(s)*) as a basis from which stakeholders could plan for the future (EA 2009A, p.xiii).
- Availability of alternative *Options* (multiplicity / diversity of *Options*). The Plan provides various *Options* (pathways); its route-map shows various *HLOs*/pathways and allows for switching between them in the future (Jeuken and Reeder 2011, p.4; Jeuken et al. 2014, p.18). The Plan identifies different possible *Options* (distinct alternative pathways), which can cope with different levels of SLR, and the thresholds at which each *Option* will be needed (HM Treasury 2009, p.13); together, the *Options* can cover a water level rise up to 4,2m. The *Options* consist of *alternative ways of managing flood risk* on the TE (EA 2009A, p.138). The final Plan provides ‘*a set of Options*’ which includes the *preferred Estuary-wide Options* (*needed to set the direction for FRM*) and *alternative Options* (*needed in case the preferred Options are no longer able to manage flood risk because the actual rates of future change are different from the rates assumed in the Plan*) – this is *a key output of the TE2100 Plan* (EA 2009A, p.279).
- By sequencing the implementation of several measures over time (i.e. through a stepwise implementation of diverse measures throughout time), and by keeping open possibilities to switch to other measures. In the TE2100, flood risk is managed iteratively: the route-map displays short-term measures linked to possible future measures (alternatives), thus, fostering *flexibility* (Ranger et al. 2013, p.248-249). In this APs approach, measures are implemented stepwise over time (as each pathway is itself a *package*/*set* of several sequenced measures); therefore, rather than making a decision now on the ‘best’ measure for a particular future scenario (which could result in maladaptation if this scenario did not occur), the APs encouraged the Team to consider multiple plausible scenarios and adopt a more flexible stance in which decisions and measures are taken over time to continuously adapt while maintaining as much flexibility as possible about future measures and *Options* (Jeuken and Reeder 2011, p.4; Ranger et al. 2013, p.250; Isoard and Winograd 2013).[[56]](#footnote-56) The APs approach ensured a stepwise implementation of measures throughout time, ~~but also~~ keeps open the possibility to switch to other measures. Actually, one way of building-in *flexibility* into the Plan was keeping options open to manage future risk (alternatives available) (Ranger et al. 2013, p.249) and not foreclosing or constraining possible future options unnecessarily (Jeuken et al. 2014, p.3; Sayers et al. 2012), which required finding measures that do not block further measures that might be needed in the future and avoiding irreversible investments as much as possible (Jeuken and Reeder 2011, p.2; Ramsbottom and Sheppard 2017, p.5). The possibility of implementing certain measures or pathways should not be prematurely closed-off, e.g. by actions of third parties (HM Treasury 2009, p.28). As an example, although the final Plan was developed based the *most probable scenario* assumed in 2009 Government guidance, it did not reduce the available *Options* for the case the future evolves differently; i.e. while the final Plan is based on the expected *most likely envelope of change*, it is flexible and adaptable to an uncertain future (EA 2009b, p.3,4). The final Plan’s *Options* were developed based on the *Defra06 scenario*, but such *Options* were tested under other (more severe) CC scenarios to ensure their ‘*robustness and adaptability to future CC*’; the overall approach resulted in *a set of FRM Options that are resilient and adaptable to the uncertainty surrounding CC* (EA 2009A, p.72).

The APs method itself involved sequencing the implementation of diverse measures over time in a way that allows the system to adapt to changes over time (e.g. SLR), and, at the same time, keeping options (alternative measures and pathways) open to cope with different plausible futures (Reeder and Ranger 2011, p.3) – this helped to build-in *flexibility* and *adaptability* into the adaptation strategy and the broader plan. The idea underlying the APs approach was *to design ‘packages’ of adaptation measures that can be implemented over time*, and ensure that these (pathways) were *appropriate to cope with the plausible range of climatic changes that could be seen by 2100*, and, at the same time, *being flexible and adaptable to an uncertain future* (Reeder and Ranger 2011, p.8, 5, 13). The outcome of the APs approach was a plan that provides (a suite of) packages of sequenced measures to manage current risk, which have *flexibility to cope with the range of possible future SLR* (Reeder and Ranger 2011, p.13). The Plan presents a series of various possible pathways (i.e. a set of *Options*) – each pathway as a set of sequenced measures to manage flood risk until 2100. Each *HLO*/pathway was drawn up *with interventions sequenced through the century to adapt to future CC* (EA 2009A, p.73).[[57]](#footnote-57)

- By using a wide variety of measures. The Plan contains different types of adaptation/FRM measures, namely measures to address the probability of flooding (e.g. defence works, managed realignment) and measures to reduce the consequences of flooding (e.g. resilience-building measures) (EA 2009A, p. 139, 141-142; Jeuken and Reeder 2011). This enhanced the Plan’s and system’s flexibility but also the system’s resilience (Jeuken et al. 2014, p.1).
- By including ‘no-/low-regret’ measures in the route-map in the near future. In the TE2100, some pathways contain *low-regrets* early measures[[58]](#footnote-58), e.g. upgrading existing defences, which will cost-effectively reduce risk and, at the same time, leave ‘*open the possibility to scale up action in the future*’ and ‘buy time’ to monitor and learn before making a major investment (Ranger et al. 2013, p.248-249; Reeder and Ranger 2011, p.6, 9, 11; Ramsbottom and Sheppard 2017, p.5).[[59]](#footnote-59) The Team strategically placed ‘low-regret measures’ early in the route-map (Reeder and Ranger 2011, p.9). All *Options* (pathways) designed include small incremental measures in the near-term (e.g. upgrades to defences, resilient urban development) and leave more irreversible measures as far as possible into the distant future, to make the best use of information that will arise (HM Treasury 2009, p.13, 27-28).[[60]](#footnote-60). The inclusion of ‘*no / low-regrets’* measures and *win-win* measures early in the route-map helped to increase the *robustness* of the general Plan and its strategies (pathways) – it was a way of ‘buying time’ before making irreversible decisions or investments (e.g. on a new barrier); it helped to gain time to monitor, learn and make better decisions (Reeder and Ranger 2011, p.11-12; Ranger et al. 2013, p.248-249, 255; Ramsbottom and Sheppard 2017, p.5). Thus, using low-regrets measures (and avoiding irreversible investments as much as possible is an important sub-element / ingredient of this key-element of TE2100’s *Dynamic Adaptive Planning* (Ramsbottom and Sheppard 2017, p.5). However, the Plan’s *flexibility* may also be constrained by the *nature* and *lifespan* of measures applied in the past, (e.g. barriers, which “live” for many decades) (Jeuken et al. 2014, p.18).
- Through ‘structural / engineered flexibility’ (i.e. by allowing the *adaptation of engineering responses* / structures). This type of flexibility involves designing an engineering measure / solution in a way that allows it to be adjusted (adapted, changed) over time, e.g. a seawall with large foundations that can be raised in the future instead of replaced (Reeder and Ranger 2011, p.3). To ensure this, structures can be designed so that they can be adapted to changing conditions (e.g. foundations of new defences can be designed so that that can take higher future water loadings, or barriers / defences designed so that they can be modified in the future); their initial cost will be higher than responses that do not allow for future adaptation, but this can bring significant savings over tehri whole life (EA 2012, p.35, 36, 38-39; EA 2009A, p.xvi, 267, 278-279). This type of flexibility might mean higher initial costs (in many cases, costs are compensated by the benefits, but in others, this flexibility may not be feasible due to financial constraints or not solve the whole problem) (Reeder and Ranger 2011, p.3). ‘Engineered flexibility’ is effective if flood defences can be adjusted in the future at limited additional costs (when its additional cost is low) (Ranger et al. 2013, p.248-249). This type of flexibility was delivered or incorporated in several measures of the TE2100, for example: by over-rotating the TB in case of greater SLR than predicted, using safety margins, over-engineering infrastructures to cope with greater change, purchasing land to construct future FRM measures.
- By using *robust measures*, i.e. measures that are suitable over a wide range of plausible future scenarios (e.g. early-warning systems, emergency plans), as well as measures inherently *flexible* (easily adjustable, e.g. sand nourishments) (Reeder and Ranger 2011, p.3, 12).
- By safeguarding land for future FRM measures through land use planning. Many FRM/adaptation measures require land (new defences, enlarged defences, new areas for habitat creation, flood storage areas, managed realignment). In the TE2100, the allocation of land via the spatial planning system must be guided by the requirements of the *Options*, in order to ensure they remain feasible (EA 2012, p.35, 36, 38-39; EA 2009A, p.xvi, 267, 278-279).
- Through the integration (mainstreaming) of the proposed FRM/adaptation measures with other agendas or multi-objective investments, namely into works of maintenance or upgrade of defences (Ramsbottom and Sheppard 2017, p.5; Jeuken et al. 2014, p.1), and through (by ensuring) the adaptation of new infrastructures in the TE (like new transport links, ports, e.g. the expected London Gateway Port at Shell Haven, etc.) that offer opportunities to combine FRM / adaptation measures with these investments and that can bring their implementation forward (if this is justified by synergies and funding opportunities) (EA 2012, p.35, 36, 38-39; EA 2009A, p.xvi, 267, 278-279).

These were the main ways of incorporating and enhancing *robustness* and *flexibility* into the TE2100Plan or its pathways. These aspects also correspond to various ways to address / respond to changes as these are monitored or each time the Plan is reviewed or updated[[61]](#footnote-61), and thus, reflect diverse ways through which the Plan is *robust* and *adaptable* to uncertain future changes. Indeed, *adaptability* is a key feature of the TE2100 Plan, which makes it possible to achieve the objectives throughout the next 100 years despite uncertain future conditions and changes (EA 2009A, p.84).

As referred in the Technical Report, the TE2100 Plan provides ‘*a set of Options*’ which includes the *preferred Options* but also other alternative *Options* (which might be necessary if the preferred *Options* are no longer effective because the actual rate of change differs from the expected – this is *a key output of the TE2100 Plan* (EA 2009A, p.279).

**Methodological approach *for Option development***

The Team had studied and developed several tools and techniques to develop a range of *Options* to manage flood risk (EA 2009A, p.x). As mentioned in the Technical Report, the Team has developed a *methodology to test different FRM measures against differing CC scenarios, which has led to the concept and development of flexible, adaptable Options for FRM* – this has been ‘*fundamental to, and the basis of, the adaptability of the TE2100 Plan*’ (EA 2009A, p.71; xv; EA 2009b, p.1).

The TE2100 Team devised an approach for the development of the Plan and its strategies (the so-called *Options*) that aimed to deal with the uncertainties surrounding the projections of future CC and developments in the TE. It devised a method of developing various *FRM Options* as pathways (each pathway as a ‘*package’* of sequenced measures, i.e. a set of sequenced measures or portfolios to cope with thresholds over time) (EA 2009b, p.3; Lowe et al. 2009, p.86). Using this approach, the TE2100 Team produced its *High-level Options* (*HLOs*) in 2007 – these were a set of FRM *Options* (HLO1, 2, 3a, 3b, and 4) (EA 2009b, p.3).

Overall, the TE2100 developed a methodology which led to a Plan that is *flexible* (i.e. dynamically robust) and *adaptable* to an uncertain future (EA 2009b, p.4); this methodology allowed the development of *flexible adaptable Options* (EA 2009b, p.1), each *Option* devised as a pathway.

The *Decision Pathways approach* (APs) led to the development of *Options* as ‘*flexible adaptable decision pathways*’ (EA 2009b, p.200).

The development of *robust* *flexible* *strategies* with the APs approach is deemed one of the main elements of the *Dynamic Adaptive Planning approach* of the TE2100 (Jeuken and Reeder 2011, p.2, 4). It can also be considered a fundamental/essential element to craft and operationalize a *robust adaptive plan*. To deal with uncertain future conditions and changes, the TE2100 Project decided to develop a *dynamic adaptive plan* that was *robust* and *adaptable*, and to construct such plan it devised the APs approach. The Team aimed to build-in *dynamic robustness* and *flexibility* into the Plan and its strategies, and the APs approach was fundamental/key to achieve this. The APs approach was essential to create a *robust adaptive plan*, and particularly, to develop a ‘*robust and flexible*’ set of measures – or, more precisely, *robust flexible sets* of measures, as each *Option* (pathway) is itself a *set* of sequenced measures.

It can, therefore, be argued that developing a ‘*robust* *and flexible*’ set of measures (or, more accurately, sets of measures) to deal with uncertain future changes, using the Adaptation Pathways approach (APs), is other key-element of the TE2100’s Dynamic Adaptive Planning that has been essential to develop and operationalize a *robust adaptive plan*. By using the APs approach, it was possible to design of several sets/packages of sequenced measures, i.e. several pathways, where each pathway is itself a *strategy*, i.e. a set of measures sequenced over time to manage changing risk. Together, the various pathways form (compound) a *robust adaptive plan* (EA 2009b, p.200; Ranger et al. 2013, p.249; Reeder and Ranger 2011; Jeuken and Reeder 2011, p.2, 4; Ramsbottom and Sheppard 2017, p.5; Jeuken et al. 2014, p.1, 23). In other words, the development/design of a series of *robust flexible* *strategies* (where each *strategy* is itself a *robust flexible set* of sequenced measures, i.e. a pathway denominated as *HLO*/*Option*), which was done by using the APs approach, can be deemed other key-element of the TE2100’s *Dynamic Adaptive Planning approach* that was essential to draw up a *robust adaptive plan*.

In synthesis, the development of a ‘*robust and flexible*’ set of measures – or, more precisely, of a set of *robust adaptive strategies* as pathways (so-called *Options*) – in order to deal with uncertain future changes, by using the Adaptation Pathways approach (APs), can be deemed a key-element of the TE2100’s approach that was essential to develop and operationalize *a robust adaptive plan*.

The production of ‘*flexible adaptable Options*’ as pathways, and the threshold analyses that it implied, were essential to develop a plan (to manage flood risk until 2100) that was *dynamic robust* and *adaptable* to uncertain future conditions and changes (EA 2009b, p.2-4, 10, 199). *Dynamic robustness* (*flexibility*) and *adaptability* are basic properties of the Plan (EA 2009b, p.2-4, 10, 199). The APs approach – which served to develop a *robust flexible set of Options* (i.e. a suite of pathways) – was fundamental to embed these properties into the Plan.

The development of *robust flexible strategies*, using the APs approach – i.e. the design/creation of a series of *Options* as adaptation pathways – has been deemed one of the essential elements of the *Dynamic Adaptive Planning approach* of the TE2100 (Jeuken and Reeder 2011, p.2; Ramsbottom and Sheppard 2017, p.5). Furthermore, the adoption of the APs approach to design diverse possible adaptation pathways is deemed one of the main innovations of the TE2100 (Ranger et al. 2013, p.233, 239, 258). Notwithstanding, few studies have recognized and approached this element as essential to craft a *robust adaptive plan* (an exception to this is the work of Jeuken et al. 2014), and fewer have investigated why it is essential and which properties of this element make it a ‘key requisite’ / necessary ingredient to make a *robust* and *adaptive* plan.

According to Jeuken et al., to deal with uncertain future change, it is necessary to develop a ‘*robust and* *flexible*’ set of measures (Jeuken et al. 2014, p.23, 1). To do this, the TE2100 devised and applied the *Adaptation Pathways approach* (Jeuken et al. 2014, p.10) – that is, the TE2100 Team has designed a *robust and* *flexible* set of measures – by using / with the APs approach (Jeuken et al. 2014, p.23).

**Theoretical grounds of *Adaptive Planning approaches***

In the light of the ***Adaptive Planning paradigm*,** in the face of deep uncertainties about future changes, a *dynamic adaptive plan* is needed, with *robust policies*/strategies and *adaptive policies*/strategies (Jeuken et al. 2014, p.2). *Robust policies*/strategies are those *perform well under a wide range of plausible futures*, whereas *adaptive policies*/strategies *can be adapted once the future unfolds differently than foreseen* (Jeuken et al. 2014, p.2). According to Jeuken et al. (2014), one of the central elements of an Adaptive Planning approach is *to respond to uncertain change with a robust and flexible set of actions* (Jeuken et al. 2014, p. 23, 1). This set of actions should: create a *robust* system, i.e. one that can cope with several plausible future scenarios, namely extreme scenarios, and remain *flexible* in relation to changes and uncertain future conditions (Jeuken et al. 2014, p.3). Thus, a *dynamic adaptive plan* should contain *robust* and *flexible* measures. In an *Adaptive Planning approach*, measures (or their set) must be: *robust* (i.e. effective under the widest range of plausible future scenarios), and *flexible*, by e.g. not foreclosing or unnecessarily constraining possible future measures (options) (Jeuken et al. 2014, p.3, based on Sayers et al. 2012). Moreover, measures should be ‘*low/no-regrets’* as much as possible (i.e. with properties like robustness, flexibility, reversibility, adaptability) (Jeuken et al. 2014, p.3). This implies considering *robustness*, *flexibility*, and *low-regrets’ content*, when choosing measures and developing strategies (Jeuken et al. 2014, p.3).

The design of a ‘robust flexible set of measures’ requires searching for measures that are *robust* and / or *flexible*. The set of measures should contain the following properties: *robustness* (ability to achieve the objectives despite how the future unfolds and be effective under a wide range of future scenarios), *flexibility* (ability to be changed over time as changes occur or as new information arises) and adaptability (ability to be adapted to changes over time, e.g. by keeping options open / not foreclosing future options unnecessarily), and be *no / low-regret* whenever possible (offer *reversibility*, *adaptability*, etc.).

The APs approach is defined as a (methodological) ***approach for exploring and sequencing a set of possible actions*** (i.e. a set of possible measures) *based on alternative external developments over time* (Haasnoot et al. 2013, p.485). Haasnoot et al. (2012, 2013) and Walker et al. (2013) place the APs within family of Adaptive Planning approaches. The APs offers a methodological approach for designing a *dynamic adaptive plan* that is both *robust* and *adaptive* under uncertain future change.

#### Key-element 4

The TE2100 Project set out (defined) its monitoring and review system, which not only specifies the main indicators that must be monitored, but also ‘*decision-points*’ and explains how to estimate them (Ranger et al. 2013, p.233, 239, 258; Jeuken et al. 2014, p.10, 22).

The TE2100 Plan defined its monitoring programme, it specified 10 indicators that must be monitored over Plan’s lifespan (e.g. mean sea level, extent of erosion, etc.) (EA 2012, p.30, 37; EA 2016; Ranger et al. 2013, p.233, 256; London Councils 2018; Ramsbottom and Sheppard 2017, p.12). These indicators serve to monitor changes in the Estuary and its flood risk (EA 2016). Yet, the monitoring system must not only monitor how the climate and flood risk are changing locally (which implies measuring local changes in the indicators), but also global changes like climate change (CC) progress and the rate of CC effects (which implies continual observation and keeping track of updated future projections and investment in climate science) (EA 2009b; Lowe et al. 2009, p.85, 90; Met Office 2012).

The *Decision-points* (which precede the critical thresholds) can be estimated and detected based on the observation of certain key-indicators: if a trigger-value predefined for certain indicator is reached, this will trigger a decision (Ranger et al. 2013, p.233, 239, 258). The Project Team has estimated some important decision-points; to this end, it used several scenarios to assess when it will be necessary to adapt at earliest and at latest, and took into consideration the lead-time required for designing and constructing a certain measure, as well as the monitoring results on a certain indicator (observed changes) – in this way, it was possible to see if a decision should be postponed or taken earlier (Jeuken and Reeder 2011, p.6). In this sense, the *decision-points* help to ensure that measures (to manage evolving risk) are timely taken and cost-effective (Ranger et al. 2013, p.233, 239, 258).

Besides the monitoring, it is also necessary to carry out regular reviews (reassessments and re-appraisals) and updates of the Plan. The TE2100 Plan must be reviewed (and updated) every 10 years or more frequently if there is a major change in one or more indicators; and the 10 indicators (monitored through a formal programme) must be reviewed against the Plan (EA 2009A, p.xv, 262-263; EA 2012, p.30), i.e. periodic reviews (re-appraisals) of the Plan were set every 10 years at least (based on the monitoring of indicators) or more frequently, if a substantial change happens in one or more indicators (EA 2012, p.30. 39; Ranger et al. 2013, p.254). Moreover, a mid-term monitoring review process must be conducted every 5 years (EA 2009b; Bloemen et al. 2018; Ramsbottom and Sheppard 2017, p.13; HM Treasury 2009, p.26-28; London Councils 2018).[[62]](#footnote-62) The regular reviews (re-appraisals and updates) of the Plan must be informed by results of the monitoring programme (Reeder and Ranger 2011; EA 2012, p.36; EA 2009b; Ranger et al. 2013, p.254; Bloemen et al. 2018; Ramsbottom and Sheppard 2017, p.13). Monitoring results must be used to periodically update of the Plan, namely: the dates when actions are needed, or if necessary, its pathways (*Options*), and / or their actions (Ramsbottom and Sheppard 2017, p.13). In this way, the potential need of periodic updates of the *Options* (pathways) could be addressed (Bloemen et al. 2018). The Plan also sets up a major review for 2050 (EA 2012, p.30, 3, 41, 49, 56).

Importantly, at each 10-yearly review and update of the TE2100 Plan, the *front runner(s) Options* for the period post-2070 will be reviewed – and around 2050 a firm decision must be made on the *end-of-the-century Option* (EA 2009A, p.xxi). As part of the regular monitoring and of the periodic planned revision of the TE2100 Plan, it will be necessary to keep the ranking of *Options* under review (EA 2009A, p.232); i.e. at each 10-year review, the *preferred Options* must be reviewed using the results of the monitoring of indicators (EA 2009A, p.262-263). Moreover, the strategic aims, the design standards of protection for flood defences, the FRM Policy, or floodplain management activities, might need to be changed as time unfolds – these issues will need to be *monitored over time and* *revisited as part of regular reviews* of the TE2100 Plan (EA 2009A, p.232, 262-263).

The monitoring of indicators is, thus, a vital component of the Plan; monitoring results must be used to update the Plan’s contents (e.g. the exact date when major interventions will be required will depend on the rate of CC and other changes as these are monitored; the dates estimated for interventions are likely to change, i.e. the actual rates of change are unlikely to be the same as the assumed rates of change, therefore, indicators must be monitored as part of the Plan, and the intervention-dates must be modified as necessary and the Plan updated) (EA 2009A, p.267).

As indicators are monitored, *monitoring results must be used to update the estimated dates when ‘portfolios’ must be implemented as well as the dates when decisions must be made* (EA 2009A, p.280). The *preferred Options* and the *alternative Options* must be re-appraised, using the updated best estimates of future change, which may lead to a change in the selected *Option*. Moreover, while the Plan contains alternative *Options*, it will be necessary to consider whether there are any other alternative options whenever the *Options* are reviewed (EA 2009A, p.280), i.e. it is wise *to consider whether the preferred options* identified in 2009 *are still the best; appraisal is (…) needed to decide the best way forward whenever the Plan is updated* (EA 2009A, p.280).

The monitoring of local changes and of the progress of climate change at global scale (as it is experienced and forecast) and of new scientific insights (1); and, on the other hand, the periodic reviews of the Plan and adaptations of the Plan and of the physical system to changes early in time as time unfolds (2) are deemed two essential factors for the effectiveness of the Plan as an *adaptive plan* and of its APs approach (Met Office 2012; EA 2009b, p.10; Jeuken and Reeder 2011, p.6).[[63]](#footnote-63) In specific, ongoing monitoring and the regular reviews of the Plan are essential to ensure that changes are timely detected and any necessary alterations to the Plan or its *Options* are made (Reeder and Ranger 2011). For example, monitoring and Plan’s reviews are required to see if a switch to other measure, or another *HLO* (pathway), is needed in light of observed changes in the indicators and new information (and such switch will be possible with no significant wasted investment in the early decades of the Plan) (HM Treasury 2009, p.27-28). As highlighted in the Report of the first 5-year review, the TE and flood risk will change over time, but the Plan *can accommodate future change and can be adjusted when the rate of change is either faster or slower than originally predicted – this flexibility was included to ensure that the actions in the Plan remain appropriate and are implemented at the right time* (EA 2016, p.15).

The monitoring programme has been conducted since the Plan’s publication (in 2012), it has kept track of changes in the indicators, and some indicators have been refined (EA 2016). Besides, in 2016 it was carried the first 5-year review (EA 2016), and in 2022 the first 10-year review.

As mentioned in the Plan itself, the indicators must be monitored, and the Plan must be regularly reassessed and reviewed (based on the monitored indicators), to ensure that the Plan remains ‘*adaptable to change*’, *flexible*, and ‘*fit for purpose*’ over its lifespan, and responds adequately to changes (EA 2012, p.36,38, 39; EA 2016; EA 2009A, p.262, xv). The ongoing monitoring of local and global changes, and the regular reviews of the Plan (including the re-appraisals and updates of its diverse contents) have helped to ensure that the Plan remains *adaptable* (Ramsbottom and Sheppard 2017, p.5, 12-13). Thus, it can be argued that defining and conducting the M&R system is another key-element of the *Dynamic Adaptive Planning approach* of the TE2100 that has been essential to develop and operationalize a *robust adaptive plan* and carry out a real *Dynamic Adaptive Planning*.

Overall, the TE2100 Plan accounted for the monitoring of both local and global changes, and for its own reassessment and review – its M&R system defines the main indicators that must be monitored, specifies key *decision-points* and how these can be estimated (which required predefining a trigger-value for a certain indicator, which will trigger a decision) (Jeuken et al. 2014, p.10, 22), and, importantly, it considers and explains ways through which (how) the Plan (and its contents) might be reviewed and adapted over time (e.g. by changing the timing for measures, i.e. ‘implementation-points’ and/or ‘decision-points’, by switching of measure or pathway, or adjusting a measure itself).[[64]](#footnote-64)

Thus, the definition and implementation of this monitoring and review system – to monitor relevant changes and new knowledge, and to regularly review (re-appraise) the Plan and adapt it accordingly – can be deemed other key-element of the TE2100’s *Dynamic Adaptive Planning approach* that has been essential to develop and operationalize a *dynamic robust adaptive plan*, and fundamental to carry out a true Adaptive Planning.

Overall, the TE2100’s monitoring and review system (M&R) seeks to ensure that the Plan is regularly reviewed and updated to reflect the existing circumstances, thus, it is crucial for the Plan’s *adaptability* (EA 2009A, p.11). Hence, monitoring of indicators is deemed *a key part of the adaptability of the TE2100 Plan to future change* (EA 2009A, p.8). As noted in the Technical Report, given the uncertainty surrounding the effects of CC, *it will be essential to have adaptability and flexibility as a key part of the Plan*, which points to *the need for the ongoing monitoring of CC science and the actual effects of CC, particularly of SLR*, as part of the implementation of the Plan (EA 2009A, p.72).

The monitoring of key indicators (monitoring activities), together with the programme of review and maintenance of the Plan itself, which were established in the Plan, are fundamental to ensure it remains ‘fit for purpose’ during its life (EA 2009A, p.262, xv). The monitoring of indicators (namely sea levels and river flows) is deemed essential, for example, to detect rates of change and identify triggers (and thus, it is an important component of the ‘blueprint for implementation’), and the produced data must be regularly used to update estimates of the dates when new interventions will be required (EA 2009A, p.11). Thus, the phase of implementation has included *monitoring activities together with a programme of review and maintenance of the Plan itself* (EA 2009A, p.262). The Plan was designed to adapt to changing climate or other factors over the next 100 years and be sustainable, thus, it must ‘*ensure that the right actions can be carried out at the right time*’ and ‘*not waste money on over-engineered solutions*’ (EA 2009A, p.262). It is *‘essential that the Plan is adaptable, to take account of future change and to avoid commitment to a course of action that may be sub-optimal because future change differs from the assumptions made in the Plan’* (EA 2009A, p.268).

#### Key-element 5

To develop a long-term *robust adaptive plan* (for FRM) in the face of uncertainties about future changes, the TE2100 Project has followed a ‘decision-centred’ planning process with several steps, which is associated to a continuous and iterative risk management (ongoing process of Adaptive Planning / Managed Adaptive approach). The *Dynamic Adaptive Planning approach* of the TE2100 presupposes an ongoing, iterative, learning-oriented planning and decision-making process (including the management process), which allows that the Plan (and its strategies / *Options*) are progressively and iteratively refined, incorporating new data over time – and thus, *adapted* (Ranger et al. 2013, p.257). This continuous process is deemed one of the main innovations of the TE2100 (and an innovation in the field of climate adaptation) (Ranger et al. 2013, p.233, 239, 258). Notwithstanding, it can also be argued, that it is also essential to operationalize a true / real adaptive planning and management process and to allow the development and implementation of a sustainable adaptive (adaptable) plan.

It is essential to keep *adaptability* and *flexibility* as key parts of the Plan, which highlights the need for the ongoing process of refinement and adaptation of the Plan and FRM system (which itself depends on the monitoring activities that form part of the implementation of the Plan) (EA 2009A, p.72).

The iterative approach taken to develop the Plan (the Plan was developed in an iterative way, involving various stages through the planned phases of the Project, in which feedback from stakeholders was fed into and informed the Plan development) (EA 2009A, p.327), must be maintained.

In sum, the ongoing process of adaptive planning (continuous process of iterative risk management and climate adaptation), which implies feeding back the results of the monitoring and review system into a new cycle of planning – a replanning of the Plan’s contents, can be considered a 5^th^ key-element essential to operationalize a true adaptive plan.

In synthesis, the main elements found in the TE2100’s planning approach that were essential to develop an adaptive plan under uncertain future changes and conditions, were:

1. The generation of a wide range of plausible future scenarios of SLR, and their use to test robustness of measures and pathways. Climate projections were generated during the development of the Plan.
2. The identification of ‘thresholds’ (which implied a ‘threshold analysis’).
3. The creation and use of the ‘Decision Pathways / Route-map / APs approach’ to develop *robust flexible strategies*, i.e. pathways (sets of sequenced measures to manage flood risk).
4. The definition of a monitoring programme, including indicators and decision-points, and the establishment of periodic reviews of the Plan.
5. The ongoing learning-oriented *decision-centred* process associated to the TE2100’s approach of *iterative risk management* (also called *Managed Adaptive approach* or *Dynamic Adaptive Planning approach*) (Ranger et al.2013, p.233, 239, 258; EA 2009b, p.3,4).

The TE2100 has been largely quoted as a leading example of how a major long-term planning project should address the emerging risks and challenges associated with climate change and climate adaptation: developing an *adaptable plan* able to manage changing risk. The elements above-mentioned were essential to develop an *adaptive plan* to manage flood risk in the TE until 2100 (EA 2009b, p.3).[[65]](#footnote-65)

In sum, the use of *threshold analysis*, the development of *flexible adaptable* *Options* (as ‘adaptation pathways’), and the development of better CC predictions, were critical to the production of a *robust adaptable plan* that can manage flood risk in the TE throughout the next 100 years. The Project has been extensively quoted as a leading example of how a major long-term planning project should tackle the challenge of an uncertain future climate and changing risk. The success of the Plan will also strongly depend on *the periodic review and effective monitoring of CC as it is experienced and is forecast* (EA 2009b, p.10; EA 2009A, p.xv, 1, 71).

#### Synthesis table (Table 3)

**Table 3** summarizes the main elements that were essential to design and implement a *dynamic adaptive plan,* as well as the fundamental ‘ingredients’ that make this Plan a *robust adaptable plan*.

| **Table 3. Key-elements of the TE21000’s *Dynamic Adaptive Planning approach*** | | |
| --- | --- | --- |
| Step of process in Fig.8 | Interpretation (deriving key-elements through an induction process) | Key-element essential to design an *adaptive plan* and operationalize a process of Adaptive Planning |
| Step 1b, then Steps 2b and 2c | From an early phase, the TE2100 Project Team decided to incorporate the uncertainties inherent to CC projections into the planning and decision-making process (EA 2009A, p.71). The existence of deep uncertainties about future effects of CC and SLR on flood risk in the TE led the Team to commission research studies to the *Met Office* and other organizations, to better understand CC effects on relative SLR, storm surge and river flows (and the uncertainties associated to CC projections), and the plausible future increases in extreme water levels in the TE, and to produce a range of future scenarios of water level rise (WLR) until 2100 (EA 2012, p.28; EA 2009A, p.71, 11).  The Team commissioned a research study to generate a range of plausible future scenarios of water level rise for TE until 2100. In the meantime, it worked with an extreme SLR scenario (the *High++*, 4,2m), which set an upper-boundary for the identification of possible measures (*responses*) and development of pathways (*HLOs*), and also with four socioeconomic scenarios (A to D). Later, three other SLR scenarios, and two socioeconomic scenarios (A and D), were used for refining and detailing the *HLOs* (into *Detailed Options for appraisal*) and then for appraising them, namely for assessing the *Options*’ robustness (Ranger et al. 2013). | KE1: Working with a wide range of *plausible future scenarios* (diverse SLR scenarios and socioeconomic scenarios, rather than a single probabilistic projection of the future), namely to assess measures and pathways on their effectiveness (Jeuken and Reeder 2011; Jeuken et al. 2014) |
| Step 1c, then in Steps 2a and 2c | The TE2100 Team identified critical thresholds in terms of the sensitivity and vulnerability of the FRM system to flood risk, which may occur between the present and the upper-bound figure of SLR initially considered (*High++*) and which would require new measures (e.g. major change points, levels of SLR at which existing defences fail, limits to the adaptation of existing defences, namely the level of SLR that poses an engineering limit to adapting defences, limits that would be disruptive for the FRM system which would imply modifying existing defences, critical levels for the system, etc.) (EA 2009A, p.149-151; Ranger et al. 2013; Reeder and Ranger 2011; Bloemen et al. 2018; Ramsbottom and Sheppard 2017; Jeuken and Reeder 2011; EA 2009b).  Thresholds served as a starting-point for planning measures and pathways (EA 2009b). To design pathways (*HLOs*), it was necessary to identify conditions under which a measure no longer meets prespecified criteria and it is necessary to either take a new measure or switch to another pathway; and, more broadly, examining under what conditions the Plan might fail and preparing actions to safeguard against this (Ranger et al. 2013, p.250), thus, the design of pathways required the identification of thresholds. The APs approach implied a *threshold analysis* (also called *scenario-neutral* or *threshold approach*). Measures were assessed on their performance and effectiveness under different plausible levels of SLR: when a measure ceased to be effective, a new measure was needed, and a pathway arose. Each *HLO* is made of sequenced measures to prevent that a certain probability of flooding (threshold level) is exceeded. | KE2: Identifying critical thresholds, i.e. conditions under which the current or a proposed measure fails (ceases to be effective / meet the objectives), or the current system performs unacceptably, and a new measure is needed (Reeder and Ranger 2011; Ramsbottom et and Sheppard 2017; Walker et al. 2013; Jeuken et al. 2014). |
| Step 2 | The TE2100 Team decided to develop a *dynamic adaptive plan* that was both *robust* and *flexible* / *adaptable* to uncertain future changes (EA 2012, p.35-39, 1, 29; EA 2009A, p.11; Ranger et al. 2013, p.233, 239, 247, 249-250, 254, 257-258; Ramsbottom and Sheppard 2017, p.1, 18), and to construct such *dynamic adaptive plan*, the Team devised a novel methodological approach: the *Adaptation Pathways approach* (APs, *Route-map* or *Decision Pathways approach* (EA 2009b, p.200, 3-4; Ranger et al. 2013, p.249; Reeder and Ranger 2011, p.5, 8).[[66]](#footnote-66)  In the APs approach, the Plan and its strategies *are designed to be adjusted over time as more is learnt about the future* or as changes occur (Ranger et al. 2013, p.249); in addition, measures are implemented over time to continuously manage risk (i.e. keep risk below acceptable levels), while keeping open options for the future (alternatives to manage future risk), thus, maintaining *flexibility*, rather than designing an optimal solution for a particular level of risk (Ranger et al. 2013, 249, 247, 239, 233, 258; Reeder and Ranger 2011, p.13, 4; Jeuken and Reeder 2011, p.4).  In the TE2100 Plan, each pathway – so-called *HLO* / *Option* – is a ‘*package*’ of sequenced measures that can be implemented over time (Ranger et al. 2013, p.249; Reeder and Ranger 2011, p.8; Jeuken and Reeder 2011, p.4; EA 2009b, p.3-4); hence, each pathway consists of a *number* (set) of measures (individual or in *portfolios*) that are implemented in sequence to manage flood risk in a staged way (Bloemen et al. 2018, p.8; Ramsbottom and Sheppard 2017, p.2, 5).[[67]](#footnote-67) Each *Option* comprises *a sequence of interventions* or *portfolios* over the next 100 years; the *dates at which one portfolio changes to another portfolio depend on key thresholds for FRM assumed* (EA 2009A, p.277, 297, 149-150, 152, 262).  Overall, the Team sought to develop an *adaptable Plan* composed of *Options*, each *Option* made up *with interventions sequenced through the century to adapt to future CC* (EA 2009A, p.xv, 73).  The TE2100’s route-map shows a set / suite of five different possible pathways (*HLOs*) (EA 2009A, p.149). By using the APs approach, the Team could design *a set of Options*, i.e. the Project Team has developed *a range of options to manage flood risk* (a range of different FRM options) (EA 2009A, p.x, 8, 72, 142, 149, 259, 279, 290; EA 2012, p.32, 34, 6, 32; EA 2009b, p.2; Bloemen et al. 2018, p.7).[[68]](#footnote-68) This *set of Options* is a key output of the TE2100 Plan.  Each *HLO*/pathway is *flexible* as it is, e.g., possible to move from a measure to another, and it is also possible to move from a *HLO* to another one, depending on the rate of change experienced (EA 2009b, p.4; Reeder and Ranger 2011, p.9; Lowe et al. 2009, p.89; Jeuken and Reeder 2011, p.4), i.e. it is possible to switch between different measures or pathways (Jeuken et al. 2014, p.10). Moreover, in the TE2100’s APs approach, the timing of new measures, and measures themselves, can be changed over time, in this way, *flexibility is built into the long-term strategy itself* (Ranger et al. 2013, p.249).  The APs approach served to identify possible measures and explore their timing and sequencing over time, and, in this way, design several different pathways (Ranger et al. 2013, p.249; Reeder and Ranger 2011, p.13).[[69]](#footnote-69) Above all, the Team aimed to build-in *dynamic robustness* and *flexibility* into the Plan and its strategies, and the APs approach was key to achieve this.[[70]](#footnote-70) The APs approach was essential to develop a ‘*robust flexible set*’ of Options, where each Option (pathway) is itself a *robust flexible set* of measures.  With the APs approach, the TE2100 Team designed a set of *HLOs* / *Options* (pathways) (Jeuken and Reeder 2011, p.4); the APs allowed the design of a ‘*robust flexible set’* of *Options* – i.e. a route-map showing a robust and flexible set of *Options*, in which each *Option* consists itself of a *robust flexible set of measures*.  Overall, the TE2100 Team devised an approach for the development of the Plan and its strategies – so-called *Options* – that deals with the uncertainties around future CC and socioeconomic developments in the TE; in specific, it devised a method for developing *FRM Options* as pathways (each pathway as a *package* of sequenced measures) (EA 2009b, p.3; Lowe et al. 2009, p.86). Using this method, which involved detecting thresholds (*threshold analysis*), the Team produced the *HLOs* – i.e. a set of different *Options* (HLO1, 2, 3a, 3b, and 4); each *HLO* as a *pathway or route through the century* (EA 2009b, p.3). This method allowed the development of *flexible, adaptable Options*, and led to a Plan that is *flexible* (*dynamically robust*) and *adaptable* to uncertain future changes (EA 2009b, p.1, 4; EA 2009A, p.71; xv).[[71]](#footnote-71) The ‘*Decision Pathways approach*’ (APs) led to the development of *Options* as *flexible adaptable decision pathways* (EA 2009b, p.199-200, 2-4, 10).  With the APs approach, the Team could design different possible routes/pathways, each pathway as a composite *strategy* (a package of measures sequenced to manage changing risk over time). Together, the various pathways formed a *robust adaptive plan*.[[72]](#footnote-72)  By using the APs approach, the TE2100 Team has developed a set of ‘*robust flexible Options*’ (*HLOs*), where each *Option* is itself a *robust and flexible set of measures*, i.e. a pathway (Jeuken and Reeder 2011, p.2-3). The APs method allowed the development of a ‘*robust and flexible*’ set of *Options*, each *Option* as a pathway, i.e. a set of sequenced measures.  It can be concluded that developing a set of *robust flexible Options* – each *Option* devised as a *robust and flexible* set of measures – by using the *Adaptation Pathways approach* (APs), to deal with uncertain future changes, is another key-element of the TE2100’s Dynamic Adaptive Planning that has been essential to draw up and operationalize a *robust adaptive plan* (EA 2009b, p.200; Ranger et al. 2013, p.249; Reeder and Ranger 2011; Jeuken and Reeder 2011, p.2, 4; Ramsbottom and Sheppard 2017, p.5; Jeuken et al. 2014, p.1, 23). In other words, the development of a ‘*robust and flexible*’ set of measures – or, more precisely, *sets of measures* (as each *Option* / pathway is itself a *robust flexible set* of measures) – by using the APs approach, is other key-element of the TE2100’s approach essential to create and implement *a robust adaptive plan*. | KE3: Developing *‘*a robust flexible set of *Options*, where each Option is itself a *robust flexible set* of measures – to deal with uncertain changes, using the *Adaptation Pathways approach* (APs) (also called *Route-map* or *Decision Pathways approach*) (EA 2009A; EA 2009b; Reeder and Ranger 2011; Ranger et al. 2013; Ramsbottom et and Sheppard 2017; Jeuken et al. 2014, p.1, 3, 23, based on Sayers et al. 2012).  This element consisted of designing *robust flexible strategies* – so-called *HLOs* / *Options* – with the APs approach (each strategy can be considered a set of measures, i.e. a pathway made up of *robust* and / or *flexible* measures) (Jeuken and Reeder 2011, p.2-3). |
| Step 3c  +  Step 5 | The TE2100 Project set out its monitoring and review system (M&R), which specifies the indicators to be monitored and how to estimate ‘*decision-points*’ (Ranger et al. 2013, p.233, 239, 258; Jeuken et al. 2014, p.10, 22). The monitoring programme is defined in the TE2100 Plan, including the 10 indicators that must be monitored over Plan’s lifespan (e.g. mean sea level, extent of erosion, etc.), to detect changes in the Estuary and its flood risk (EA 2012, p.30, 37; EA 2016; Ranger et al. 2013, p.233, 256; London Councils 2018; Ramsbottom and Sheppard 2017, p.12). This programme not only monitors how the climate and flood risk are changing locally (by measuring changes in the indicators), but also global changes like climate change and the rate of its effects (observation of updated future projections) (EA 2009b; Lowe et al. 2009, p.85, 90; Met Office 2012). The *decision-points* trigger a decision on the measures to be implemented based on the observation of the indicators.[[73]](#footnote-73)  Besides this, the TE2100 carries out regular reviews (reassessments) of the Plan. The TE2100 Plan must be reviewed every 10 years or more frequently if there is a major change in one or more indicators, i.e. periodic reviews of the Plan were set every 10 years at least (based on monitoring results) or more frequently if a substantial change happens in one or more indicators (EA 2012, p.30, 39; EA 2009A, p.xv, 262-263; Ranger et al. 2013, p.254). Moreover, a mid-term monitoring review process must be conducted every 5 years (EA 2009b; Ramsbottom and Sheppard 2017, p.13).[[74]](#footnote-74)  The monitoring of local changes and of the progress of CC at a global scale (as experienced and forecast) and of new scientific insights, and also the periodic reviews of the Plan and adapting it and the physical system to changes early as time unfolds, are deemed two essential factors for the effectiveness of the Plan as an *adaptive plan* and of its APs approach (Met Office 2012; EA 2009b, p.10; Jeuken and Reeder 2011, p.6).[[75]](#footnote-75) In specific, the ongoing monitoring and the regular reviews of the Plan are essential to ensure that changes are timely detected and any necessary alterations to the Plan (or its *Options*) are made (Reeder and Ranger 2011). For example, monitoring and Plan’s reviews are required to see if a switch to other measure, or *Option* (pathway), is needed given observed changes in the indicators and new information (HM Treasury 2009, p.27-28). [[76]](#footnote-76) Monitoring of local and global changes, in particular, is necessary to examine if *Options* (pathways), their measures, or decisions regarding them, should be taken earlier or postponed, revised or altered, namely in the *Action Plan* (Jeuken and Reeder 2011), thus, it is essential to allow adaptations of the Plan and its pathways (EA 2009A, p.11, 8, 72).[[77]](#footnote-77)  The monitoring programme has been conducted since the Plan’s publication (in 2012), it has kept track of changes in the indicators, and some indicators have been refined (EA 2016).[[78]](#footnote-78)  Overall, the TE2100 Plan accounts for the monitoring local and global changes, and for its own reassessment and review – its M&R system defines the main indicators that must be monitored, specifies key *decision-points* and how these can be estimated (Jeuken et al. 2014, p.10, 22), and it describes ways through which (how) the Plan and its contents can be reviewed and adapted over time (e.g. by changing the timing for measures, i.e. ‘implementation-points’ or ‘decision-points’, by switching of measure or pathway, or adjusting a measure itself).[[79]](#footnote-79)  The definition and implementation of this monitoring and review system – to monitor relevant changes and new knowledge, and to regularly review (re-appraise) the Plan and adapt it accordingly – can be deemed other key-element of the TE2100’s *Dynamic Adaptive Planning approach* that has been essential to develop and operationalize a *robust adaptive plan*, and fundamental to carry out a true Adaptive Planning. | KE4: Continuously monitoring relevant changes and new information, periodically reassessing the Plan, and reviewing and adjusting (adapting) it accordingly (Ranger et al. 2013; Jeuken et al. 2014; Sayers et al. 2012; Walker et al. 2013; Bloemen et al. 2018).  This implies a targeted monitoring of changes (in external conditions, effects of measures) and continual evaluation of new knowledge (e.g. updated scenarios); as well as regular reassessments (reviews) of the Plan, and, if necessary, its adjustment (by updating, redefining or modifying policies, pathways, their measures, or their timing, according to monitoring outputs) (Jeuken et al. 2014; Sayers et al. 2012; Walker et al. 2013; Bloemen et al. 2018). |
| Entire process | The TE2100 has followed an ongoing iterative process of *Adaptive Planning* (also named *Iterative Risk Management*), which involves several steps that allow adapting the Plan to uncertain changes over time. A continuous cycle has been carried out, with several steps: i) assessment of current and potential future risk and impacts, including the definition of critical thresholds for the system’s vulnerability and sensitivity; 2) identification of possible measures and development of *Options* (pathways) considering a wide range of plausible future scenarios; 3) selection of preferred *Option(s)* (decision-making); 4) implementation; 5) monitoring of changes, applied measures and new information, and periodic reviews of the Plan and, if necessary, correct, alter, or change its pathways, measures, or their timing (by feeding back new information into any of the prior steps). This iterative process is learning-oriented and safeguards the adaptability of the Plan over time – the ability to adapt the Plan, its policies, measures, the timing of decisions or actions, as new knowledge or changes arise – so that it can better cope with uncertain future change. It enhances | KE5: ongoing process of *Adaptive Planning* (*Iterative Risk Management* *approach*), which involves several steps necessary to allow adaptations of the Plan to changes. |

**Table 3.** Main elements of the TE2100’s *Dynamic Adaptive Planning approach* that were essential to develop an *adaptive plan* and operationalize a real Adaptive Planning, which were identified and derived from the TE2100 case. Source: own elaboration, based on EA 2012, 2009a, 2009b; Ranger et al. 2013, 2010; Reeder and Ranger 2011; Ramsbottom and Sheppard 2017; Lowe et al. 2009; HM Treasury 2009; Penning-Rowsell et al. 2013; London Councils 2007, 2018; etc.

# NOTE 4. Key-elements of the ADM approach of the DP essential to design robust adaptive strategies

The DP devised and used *Adaptive Delta Management* (ADM) as its Adaptive Planning approach, and with the aim of designing strategies that were both *robust* and *flexible*, and thus, *dynamic adaptive*. This section identifies the key elements of the DP’s ADM approach that were essential to develop *adaptive strategies* and that make the DP an *adaptive programme* ‘*per se*’, and describes how these elements were applied in practice by the DP staff and Subprogrammes. This section builds on the prior analysis of the DP and on studies of other authors.[[80]](#footnote-80) The main elements of ADM that were essential to develop to *dynamic adaptive strategies* are synthesized in **Table D**.

In the light of the paradigm of Adaptive Planning, under deep uncertainty about future changes, it is necessary to develop a ‘*dynamic adaptive plan*’, and such plan must have *robust and* *adaptive policies* (strategies). Robust policies / strategies ‘*perform well under a wide range of plausible futures*’, while adaptive policies / strategies ‘*can be adapted once the future unfolds differently than foreseen*’ (Jeuken et al. 2014, p.2).

In 2010, the DP Commissioner claimed that the DP has introduced a new way of planning, initially called *‘Adaptive Delta Planning’*, which ‘*seeks to maximize flexibility; keep options open and avoid ‘lock-ins’* (Kuijken 2010 *in* Haasnoot et al.2013 p. 490; Werners et al. 2016). The deep uncertainties about future climatic and socioeconomic changes, and, on the other hand, the need to make responsible financial investments, demanded a more ‘*flexible*’ but also *realistic (down-to-earth) approach*, which gave rise to the DP’s *Adaptive Delta Management* (ADM) approach (Rhee 2012 *in* DP 2014, p.138; 2011, p.16, 48, 70; 2013, p.6). The new planning approach should consider, and be able to deal with, deep uncertainties about future changes and conditions, therefore, the DP used an *adaptive way of planning* (Alphen 2015). Such ‘*adaptive approach*’ should be *flexible* enough to adapt the plan / strategy to changing and unexpected future conditions, and at the same time, devise short-term measures and investments under uncertainty (Brugge and Brugeman, p.1). ADM responds to these two aspects: it is *adaptive* to change and it copes with uncertainties about the future (Deltares 2018). Overall, the DP devised ADM as an *adaptive planning approach* to cope with uncertain future changes and conditions (Ritzema and Steensma 2018). With ADM, the DP aims to remain ‘adaptable’ to changing climatic and socioeconomic conditions (Restemeyer et al. 2017, p.921).

Importantly, the *ADM Implementation Guide* (Rhee, 2012) recommends the use of (*suggests working with*) *scenarios*, *Tipping-points* and *Adaptation Pathways* (Restemeyer et al. 2017, 930).

## 4.1. Deriving the key-elements of the DP’s ‘ADM’ required to develop *flexible adaptive strategies*

#### Key-element 1

The ADM approach proposes the use of a spectrum of plausible future scenarios (Gersonius et al. 2016, p.4). According to ADM, it is necessary to work with various *plausible futures* (not predictions) to find out and design *robust flexible* *strategies* (i.e. consider various scenarios when exploring measures and developing strategies) (Marchand and Ludwig 2014, p.12). The *ADM Implementation Guide* (Rhee 2012) itself suggested *working with* *scenarios* (Restemeyer et al. 2017, 930).

Given the uncertainty about the rate of CC, the DP decided to work with several possible future scenarios – denominated *Delta Scenarios* – and draw up strategies for them (DP 2013, p.6). As noted in the DP (2014), to ensure a *sustainable and robust FRM* and cope with climatic extremes, the country should be *prepared for various scenarios* (DP 2014, p.6).

To deal with uncertain future changes and conditions – and *render them manageable* – the DP has worked with four plausible futures, so-called *Delta Scenarios*: *Busy*, *Steam*, *Rest* and *Warm* (DP 2014, p.135; 2013, p.6, 100; 2012, p.35; 2011, p.14, 46, 48,71; Marchand and Ludwig 2014, p.12; Deltares 2018).[[81]](#footnote-81)

As seen, in the DP, the ADM process started with the analysis of current and future problems (Gersonius et al. 2016, p.205); the DP analysed which developments or changes might influence the current and future tasks on FRM and FS (DP 2012, p.88), and the analysis of *what might happen in the future* required the generation of the four *Delta Scenarios* (Klijn et al. 2016).[[82]](#footnote-82)

The DP Team considered[[83]](#footnote-83) and worked with a range of four *plausible futures –* the so-called four *Delta Scenarios* (*Busy*, *Steam*, *Rest* and *Warm*) (DP 2012 p.35; 2011, p.14, 46, 48, 71; 2013, p.6, 100; 2014, p.135; Marchand and Ludwig 2014, p.12; Deltares 2018), and used them, namely, to assess measures and strategies (Jeuken et al. 2014, p.1, 3, 10, 14, 23). All Subprogrammes worked with the same four *Delta Scenarios* (DP 2012, p.35; 2011, p.48, 71; 2013, p.6; Marchand and Ludwig 2014, p.12; Werners et al. 2016).

The *Delta Scenarios* address uncertainties about climate change (CC) and its effects, and socioeconomic development (Bloemen et al. 2018). The *Delta Scenarios* have 2050 and 2100 as time-horizons / reference-years (not fixed endpoints) where CC and socioeconomic developments vary (DP 2013, p.100; 2011, p.48, 70). The four *Delta Scenarios* vary (differ) in terms of rapid or moderate CC and socio-economic growth / decline (DP 2012 p.35; 2014, p.168; Jeuken et al. 2014, p.1, 3, 10, 14, 23; Gersonius et al. 2016, p.208; Marchand and Ludwig 2014; Haegen and Wieriks 2015; Alphen 2015; Restemeyer et al. 2017; Bloemen et al. 2018):

- Regarding CC, the DP decided to use the ‘formal’ Dutch climatic scenarios, which cover a large range of plausible futures but not high-end projections (these scenarios cover extreme flood events up to 1/10 000 years).[[84]](#footnote-84) The main climatic parameters considered in the *Delta Scenarios* were SLR, storm surges, and river discharges (Jeuken et al. 2014, p.10, 11; Alphen 2015; Haegen and Wieriks 2015).
- Regarding socioeconomic development, the Team generated scenarios for economic and population growth until 2100, and for land use until 2050 (Jeuken et al. 2014, p.13). Moreover, maps of the expected expansion of urban, nature and agricultural areas in 2100, were also produced for each *Delta Scenario* (Brugge and Bruggeman 2019, p.3; Deltares 2018).

The *Delta Scenarios* are four different *plausible futures*, which show how the climatic and socioeconomic conditions might change until 2050 and 2100, they are not *most probable scenarios* nor predictions (the DP did not assign any probability to them) (Jeuken and Reeder 2011, p.7; Brugge and Bruggeman 2019 p.3; DP 2014, p.138; 2011, p.71). The *Delta Scenarios* present *a moderate range of scenarios*, i.e. a modest bandwidth of possible future developments, as some developments may fall outside this bandwidth (Jeuken et al. 2014, p.1, 10-11; Brugge and Bruggeman 2019).

The *Delta Scenarios* present their respective figures for SLR, river discharges, and soil subsidence (DP 2011, p.14, 46; 2012, p.35; 2013, p.100; 2016, p.6; 2014, p.136; Marchand and Ludwig 2014; Deltares 2018; Brugge and Bruggeman 2019; Jeuken et al. 2014, p.10, 11).

Overall, for the DP, the four *Delta Scenarios* represented the ‘*corner flags of the playing field of plausible futures*’ (Alphen 2015, p.312), and translate the most relevant uncertainties about plausible future changes and diverse sociocultural perspectives (Marchand and Ludwig 2014, p.15).

Although each *Scenario* has its corresponding climatological and socioeconomic circumstances, the climatological and socioeconomic circumstances may turn out differently than expected in the *Delta Scenarios*, and, in addition, the *Delta Scenarios* may be periodically updated over time, therefore, the *Preferential Strategies* were designed to be *adaptive* and *resistant to slower and faster CC* than the expected (DP 2014, p.168, 136; 2016, p.6).

**Purposes of using a range of future scenarios**

The *Delta Scenarios* served to:

- Investigate future flood- and water-related problems, risks and needs by 2050 and 2100, i.e assess possible future risks and impacts (Jeuken et al. 2014, p.10, 11; Alphen 2015, p.312; Brugge and Bruggeman 2019, p.5; Haegen and Wieriks 2015). As seen, in Sub-step 1b of the ADM process (*identifying current and future ‘vulnerabilities’ and ‘opportunities’ under different future scenarios*), it was necessary to generate an ensemble of plausible future scenarios, in which relevant uncertainties should be considered (Haasnoot and Jeuken; Gersonius et al. 2016), then, such scenarios were compared to the objectives to see if problems (vulnerabilities) or opportunities arise; and, subsequently, were used to determine when the first ATP might occur (and a new measure might be needed) (Haasnoot and Jeuken). In 2012, the *Delta Scenarios* were used by the Subprogrammes to explore what problems of flood safety and freshwater supply might occur in the future (Brugge and Bruggeman 2019, p.5; Haegen and Wieriks 2015).
- Identify future tasks. The *Delta Scenarios* were used as the basis for analysing the future tasks (DP 2011, p.14, 20). The various *Delta Scenarios* were used for identifying the tasks for each of 9 regional Subprogrammes (DP 2011, p.14). In this year of ‘problem definition’ (2010-2011), the Subprogrammes focused on identifying adaptation needs, e.g. by analysing *what are the effects of CC on the region* and *how can the long-term safety of the region be safeguarded* (Werners et al. 2016).
- Support the development of the *Delta Decisions* and *Preferential Strategies* (inform strategy development) (Bloemen et al. 2018; DP 2014, p.136). The *Delta Scenarios* were used as the basis for developing the *Strategies* (DP 2011, p.14, 20), i.e. the Subprogrammes developed their *Strategies* based on the *Delta Scenarios* (DP 2013, p.32).[[85]](#footnote-85) The *Delta Scenarios* were used in the development of *Strategies*, namely to: assess the various measures and determine when these will be needed (Brugge and Bruggeman 2019), and to determine when the current actions will become ineffective or insufficient (the date of first ATP) (Alphen2015). In this sense, the *Delta Scenarios* supported the design of *Strategies*: they were used for testing measures and strategies (*paths*), on their performance, under different future conditions (Marchand and Ludwig 2014, p.12; Alphen 2015). For example, in 2009/2010, the DP Team and the Subprogrammes, used the *Delta Scenarios* to assess how much longer the current measures (policy and management actions) will suffice and when adjustments will be required – i.e. when the first tipping-point of the existing system will be reached (DP 2010, p.3-4, 32). The various scenarios were used in the assessment of individual measures and strategies (Jeuken et al. 2014, p.10, 13), i.e. to test the performance of measures and strategies (*paths*) (Marchand and Ludwig 2014, p.12; Alphen 2015).
- Moreover, the *Delta Scenarios* were used in the assessment of individual measures and strategies (*paths*) in cost-benefit and multi-criteria analyses (Jeuken et al. 2014, p.10, 13), and for appraising their robustness (Seijger et al. 2017). All *Preferential Strategies* were assessed under the *Delta Scenarios* (DP 2016, p.6). Step X implied assessing the proposed strategies (*paths*) on their *robustness* and *flexibility*, under different scenarios, which could lead back to Step 2 in an iterative way (Gersonius et al. 2016, p.204-206; Zandvoort et al. 2018, p.189; Brugge and Bruggeman 2019, p.4-5; Deltares 2018). Each *Preferential Strategy*, with its measures, was analysed under the *Delta Scenarios* (DP 2016, p.6); the diverse strategies (*paths*) devised by each Subprogramme were subjected to a ‘robustness test’ to see which strategies were effective under the different scenarios; the ‘*preferential*’ are those that performed well even in more drastic climatic scenarios (DP 2014, p.8).
- Define the earliest and latest moment to adapt, considering the lead-time necessary for planning and implementing measures (Jeuken and Reeder 2011, p.7) (i.e. implementation-moments and decision-moments).

Overall, the use of several scenarios played a key role in the design of *robust flexible strategies*, as it allowed the anticipation of future changes and the explicit consideration of uncertainties, assisted in identifying which measures must be taken in the short-term and which can be taken later (within a long-term strategy) (Marchand & Ludwig 2014).

Working with (considering and preparing for) a wide range of climatic and socioeconomic scenarios, namely to assess measures and strategies (*paths*), can, therefore, be deemed a key element of the ADM approach that has been essential to develop and implement *robust adaptive strategies* and to operationalize a real *Adaptive Delta Planning and Management* (as also highlighted by Jeuken et al. 2014, p.1, 3, 10, 14, 23).

#### Key-element 2

The *ADM Implementation Guide* recommends using (*suggests working with*) *Tipping-points* (as noted by Restemeyer et al. 2017, 930). The ADM approach itself uses (utilizes/employs) the method of *Adaptation Tipping-Points* (ATPs) (of Kwadjik et al. 2010) (as recognized by Gersonius et al. 2016, p.204, 213). In the ADM process, namely in Step 1, it is necessary to identify external developments to which the defined objectives are most vulnerable to, by using scenarios, and in Step 2, it is necessary to analyse *what amount of change can the system handle*, and in specific, assess *what is the critical level (ATP,* Kwadjik et al. 2010*) before the objectives are not met anymore*, and *when* this occurs in different future scenarios (Jeuken et al. 2014, p.4). In ADM, ATPs refer to critical levels that represent a threat to the objectives (Jeuken et al. 2014, p.4).

In ADM, ATPs are defined as *points where the magnitude of change due to external pressures such as SLR or peak discharges is such that the current strategy* (measure) *will no longer be able to meet the objectives and thus the measure is no longer adequate* (*in* Zevenbergen et al. 2018, p.4, based on Kwadijk et al. 2010).[[86]](#footnote-86) In the ADM approach, ATPs are understood as ‘*points where the magnitude of change due to socioeconomic developments, climate change or sea-level rise is such that the current strategy* (measure) *will no longer be able to meet the objectives*’; an ATP indicates conditions under which a given measure fails and other measure is needed (e.g. a level of SLR at which a storm surge barrier ceases to function); i.e. a switch to a new measure is needed once an adaptation tipping-point is in sight (Haegen and Wieriks 2015, p.51).

In ADM, *tipping-points* correspond to points / conditions under which the existing (current) management actions / measures or policies become ‘*too expensive, technically impossible or societally unacceptable*’; the date of an ATP indicates an ‘expiration date’ at which a given measure or policy ceases to be feasible (Rhee 2012, p.18, *in* Zandvoort et al. 2018, p.190).

To support the application of ADM and its four core principles in the DP and its Subprogrammes, two main methods were used: Adaptation Tipping-Points (ATPs) (Kwadijk et al. 2010) and Adaptation Pathways (APs) (Haasnoot et al. 2013; Haasnoot 2013) (*in* Klijn et al. 2015, p.849; Jeuken and Reeder 2011, p.5; Marchand and Ludwig 2014, p.27).

The DP staff and the Subprogrammes sought to define and identify *Adaptation Tipping-Points* (ATPs). As noted by Jeuken and Reeder, an important aspect of the DP was taking the vulnerability of the existent system as starting point of the analysis (i.e. the analysis of the vulnerability of the current system was the starting point for all planning process), by focusing on the question of ‘*what change can the system handle before it runs into trouble*’ (Jeuken and Reeder 2011, p.2, 3).[[87]](#footnote-87) In this way, the DP and its Subprogrammes could identify and specify *adaptation tipping-points* (i.e. points / conditions at which the objectives of FRM policy along the coast and rivers are no longer met, or points under which the existing management strategies, are no longer able to meet the objectives and alternative strategies are needed)[[88]](#footnote-88), and analyse when these ATPs might be reached (Jeuken and Reeder 2011, p.3, 4). The ATPs’ method helps to determine at which point in time new management measures will be necessary (Jeuken and Reeder 2011, p.4).

First, the DP Subprogrammes sought to identify ATPs in the existing FRM system; to this end, they assessed technical and physical limits of the flood defence system, quantified its current overcapacity, and defined new design criteria based on this and on climate projections (Jeuken and Reeder 2011, p.6). The DP identified limits (thresholds) for the existing flood defence system, but also limits of the existing management measures and policies (Jeuken and Reeder 2011, p.7). [[89]](#footnote-89) Then, the Team used various scenarios to determine the moment of an ATP (at earliest and at latest), and the moment to adapt (i.e. to take a new measure) (Jeuken and Reeder 2011, p.6). Importantly, in the DP, the *implementation-moments* and *decision-moments* depend on the ATPs of measures, but also on the lead-time required for designing and implementing a measure, and on early opportunities to combine measures with other investments (Jeuken and Reeder 2011, p.5).[[90]](#footnote-90)

As seen, Step 1 of ADM process (*analyse objectives, current and future ‘vulnerabilities’ and ‘opportunities’ under different future scenarios*) required assessing what amount of change can the system handle and specifying (what are) *Adaptation Tipping-points* (i.e. assessing what are the critical levels at which the objectives are not met anymore), and assessing when these ATPs will occur by using various scenarios (Jeuken et al. 2014, p.4-5), by examining ‘*how long the current management strategies continue to be effective under different CC scenarios*’ (Deltares 2018, p.2). Thus, in Sub-step 1b, it was necessary to specify (define) what are ‘*adaptation tipping-points*’, i.e. the ‘boundary conditions’/ points under which the objectives are no longer met (e.g. an unacceptable level of SLR) (Kwadjik et al. 2010), and then, confront such ATPs with a range plausible futures to estimate the moment when such ATPs might occur (Gersonius et al.2016, p.204-206). It was important to use different futures with changing conditions to gain insight into the possible occurrence of ATPs, and estimate when the first ATP might happen (the point at which the current measure will no longer meet the objectives and new measures will be required) (Gersonius et al.2016, p.204-206).

In 2009/2010, the DP Team and the Subprogrammes used the *Delta Scenarios* to assess how much longer the current measures (policy and management actions) will suffice and when adjustments will be required – i.e. when the first tipping-point of the existing system will be reached (DP 2010, p.3-4, 32). The main issue was analysing if and for how long the current measures will still be satisfactory under a changing climate (more than determining the exact levels of SLR) (DP 2010, p.36). The DP Team and Subprogrammes analysed for how long the current measure will suffice and when the first tipping-point (at which such measure is no longer tenable) will be reached, to answer this, the *Delta Scenarios* were used (it was not known the rate at which the climate will change, an ATP may be reached at any time) (DP 2010, p.68).[[91]](#footnote-91)

The DP defines *tipping-points* as points at which the existing system ceases to meet the requirements (DP 2011, p.8); a *tipping-point* occurs when, due to changes in climate or socioeconomic circumstances, the existing measure, policy, or infrastructure, becomes insufficient to comply with the defined criteria (due to physical, technical, or financial constraints or socially unacceptable effects) (DP 2011, p.71). The analysis of ATPs sets out which and when decisions and new measures must be taken (DP 2011, p.55).

- *The scenarios are used to map out how much longer current policy and management are expected to suffice and when adjustments will be required. In other words, when will we reach the tipping points for our water system* (DP 2010, p.3-4, 32, 36, 68-69).
- In 2010/2011, the DP stimulated the Subprogrammes to assess *how much longer current policies and management practices were expected to suffice and when adjustments would be required* (Werners et al. 2016, p.116) – this is related with the identification of the first ATPs: a failure of the pre-existing policy or management measures.
- The Rhine Estuary-Drechtsteden Subprogramme developed *adaptation paths*, which explicitly outline at what times interventions will be required (i.e. tipping-points) (DP 2012, p.90).

Overall, in the DP, ATPs describe conditions under which the current or alternative management measures or policies might fail (Kwadijk et al. 2010 *in* Jeuken et al. 2014, p.17); in the DP, ATPs are associated to acceptable return periods for flood events, which were translated into design criteria for flood defences (in line with the DP’s *risk-based approach*) (*in* Jeuken et al. 2014, p.17).

Besides this, in the ADM process, Sub-step 2b implies/involves assessing the performance and effectiveness of each measure and determining its tipping-point. Thus, in ADM, the *adaptation tipping-points* (ATPs, i.e. points/ conditions under which a measure no longer meets the defined objectives) are crucial to the design of adaptation pathways; when an ATP is reached, other / additional measures are required to achieve the objectives, thus, the APs map shows which measures are available and when these will be needed (Deltares 2018, p.2-4). ATPs played a key role in phasing possible measures within each *Strategy* of the DP (in the sequencing and assembling of pathways) (Jeuken et al. 2014, p.17). The reaching of an ATP indicated that a new measure is required. The Subprogrammes sought to identify the ATP for diverse FRM measures, and then analysed when this might occur (i.e. the moment of ATP for different measures).

The design of the *Strategies*, namely of their *adaptation paths*, implied the identification of *Adaptation Tipping-Points* (Kwadijk et al. 2010) (*in* Jeuken et al. 2014, p.17). Hence, the design of adaptation pathways required the identification of ATPs.[[92]](#footnote-92) More precisely, in ADM, ATPs indicate conditions under which a given measure or action ceases to be effective or acceptable (or no longer suffices), and a new measure is required (source).

In 2011/2012, to start developing their *paths*, some Subprogramme analysed at what times measures will be required, which implied examining the moment of *tipping-points* (DP 2012, p.90).

Subsequently, in 2012/2013, each Subprogramme sought to design *development paths* or *adaptation paths* as part of its *Promising Strategies*, and this implied analysing the conditions under which it is logical to move from a measure to another (i.e. ATPs), and what and how options can be kept open to allow such transition (DP 2012, p.81, 80).

It can, thus, be argued that the identification of *Adaptation Tipping-Points* (ATPs) is another key-element of the ADM approach that was essential to develop and operationalize a *robust flexible strategy/plan* in each Subprogramme, and required to deliver a process of *Adaptive Delta Management*. In ADM, ATPs are needed to handle uncertainty about future changes and ensure *adaptiveness* (as noted by Zandvoort et al. 2018).

#### Key-element 3

ADM aims at developing strategies that are *robust* and *flexible*: robustness involves ‘*performing satisfactorily under a wide variety of futures*’, whereas flexibility is related with being ‘*easily adapted to changing or unforeseen future conditions*’ (Haasnoot 2013, *in* Marchand and Ludwig 2014, p.8, 15). Importantly, the 2^nd^ and 3^rd^ principle of ADM are clearly related to *robustness* and *flexibility* (DP 2013, p.103). The *ADM Implementation Guide* claims that strategies should be simultaneously *robust* and *flexible* (Rhee 2012 *in* Restemeyer et al. 2017, p.930): a robust strategy is one that *works in all plausible futures*, while *flexibility* means that *depending on the contextual circumstances - you can cut one strategy off and* *switch to another one* (DP Staff member, *in* Restemeyer et al. 2017, p.930). Moreover, according to this Guide, to find (or develop) *robust* *flexible strategies*, it is necessary to identify tipping-points and make adaptation pathways (the Guide suggests working with scenarios, tipping-points and adaptation pathways) (*in* Restemeyer et al. 2017, p.931, 930).[[93]](#footnote-93) The design of *robust flexible* *strategies* requires identifying and assessing various possible measures under a range of plausible futures and searching for those that are *robust* and *flexible* (Marchand and Ludwig 2014).

The DP assumed that its *Strategies* (the *Strategies* developed) should be both *robust* *and flexible*[[94]](#footnote-94): it defines a *robust strategy* as one that is *future-proof* and provides a solution for the tasks arising in all *Delta Scenarios* considered, and a *flexible strategy* as one that can be relatively easy accelerated / delayed, and that enables switching to other strategies (i.e. *switching between strategies*, by shifting from a strategy, or from a measure to another one), or *stepping up* measures, or *changing strategies* (DP 2013, p.103, 94; 2014, p.6). The ultimate goal is that the FRM system meets the requirements *at all times* (in all scenarios), which required a strategy that was *able to adapt to new insights and circumstances* and that contains *sufficient options to remain open in the future to take the required measures* (DP 2013, p.102). More specifically, each DP’s *Preferential Strategy* should be: *robust* (i.e. with such *Strategy*, the objectives can be achieved in all scenarios of CC and socioeconomic development considered, i.e. under all *Delta Scenarios*) and *flexible* (i.e. the implementation of the strategy(ies), or their measures, can be sped up or slowed down and it should be possible to change to a different measure or a different strategy (*path*), if necessary) (DP 2013, p.94). ‘*Flexibility*’ is related with the capacity to respond to changes, new developments, and knowledge (DP 2013, p.94-95). Flexibility was provided, among other ways, *‘through the types of measures selected, by leaving options for adjustment or switching to other measures open for the future, in this way, measures can be adjusted to new insights and circumstances*’ (DP 2014, p.169).

The *Strategies* developed in the DP should be *robust* and *flexible*, and when developing its *Strategies*, each Subprogramme elaborated on the four basic principles of ADM (DP 2013, p.103, 102, 95; 2012, p.88; 2011, p.48; Werners et al. 2016). ADM was adopted by the DP as a new planning approach to achieve *robust flexible pathways* to manage flood risk and ensure freshwater supply (Werners et al. 2016), and the ADM itself employed an adaptive (methodological) approach for designing strategies under different future scenarios and changes – the APs approach (Jeuken and Reeder 2011, p.5; Haegen and Wieriks 2015, p.56, 54-55, 48; Restemeyer et al. 2017, p.935, 930; Marchand and Ludwig 2014, p.2; Brugge and Bruggeman 2019, p.2; Zandvoort et al. 2018, p.189; Klijn et al. 2015, p.849; Bloemen et al. 2018, p.5).

Flexibility could be delivered in several ways, for example: by designing each strategy as an *adaptation path* (pathway), by using various pathways (i.e. working with various potential strategies, i.e. various *adaptation paths*, between which it is possible to alternate), by using solutions that are flexible *per se*, by leaving options for adjustment, by linking agendas, etc. (DP 2014, p.169; 2013, p.103, 102, 95;2012, p.88; 2011, p.48). These types of flexibility create added value in terms of cost reduction, feasibility, and benefits (DP 2014, p.169).

The four basic principles of ADM have substantiated the *Preferential Strategies* (DP 2016, p.7). More precisely, the *Preferential Strategies* were developed based on the four basic principles of ADM (DP 2014, p.47; 2013, p.102; 2012, p.88; 2011, p.48-49; 2016, p.7; 2017, p.7). While developing their (*Promising*) *Strategies*, the Subprogrammes sought to meet / ensure the four essential principles of ADM (DP 2013, p.102). To support the application of the four basic principles of ADM in the DP, two main methods were used: Adaptation Tipping-Points (ATPs) (Kwadijk et al. 2010) and Adaptation Pathways (APs) (Haasnoot et al. 2013; Haasnoot 2013) (as noted by Klijn et al. 2015, p.849). The ATPs and APs were applied for FRM and FS (Klijn et al. 2015, p.849).

The ADM approach (namely its 3^rd^ basic principle) implied the development of adaptation pathways. The design of pathways is, indeed, one of the main steps of the ADM process (illustrated in its diagram). ADM itself uses the method of *Adaptation Pathways* (APs) (Jeuken and Reeder 2011, p.5; Haegen and Wieriks 2015, p.56, 54-55, 48; Restemeyer et al. 2017, p.935, 930; Marchand and Ludwig 2014, p.2; Brugge and Bruggeman 2019, p.2; Zandvoort et al. 2018, p.189; Klijn et al. 2015, p.849; Bloemen et al. 2018, p.5). The APs approach, as employed in ADM, is an adaptive approach to design ‘strategies’ as adaptation pathways – *to develop a set of measures along adaptive pathways* – which incorporates flexibility to switch to other measures and strategies when needed, in view uncertain future socio-economic developments and climate change (*an adaptive approach that allows for switching between strategies along adaptation pathways when needed in view of socio-economic developments or climate change*) (Haegen and Wieriks 2015, p.55, 54, 56).[[95]](#footnote-95)

To develop their *Preferential Strategies*, the Subprogrammes applied the ADM approach, and ADM itself employs the method of Adaptation Pathways (APs approach) (*in* Jeuken and Reeder 2011, p.5; Restemeyer et al. 2017, p.935, 930; Haegen and Wieriks 2015, p.56, 54-55, 48; Marchand and Ludwig 2014, p.2; Brugge and Bruggeman 2019, p.2; Zandvoort et al. 2018, p.189; Klijn et al. 2015, p.849; Bloemen et al. 2018, p.5). One of the *building blocks* for ADM is *the development of adaptive pathways* (Marchand and Ludwig 2014, p.17). In its process, ADM includes the APs method (of Haasnoot et al. 2012, 2013), indeed, the 3^rd^ step of the ADM process is the development of adaptation pathways and APs’ map. The APs consists of a methodological ‘*approach for* *exploring and sequencing a set of possible actions* (i.e. a set of possible measures) *based on alternative external developments over time*’ (Haasnoot et al. 2012, p.485); it is one of the Adaptive Planning approaches (Haasnoot et al. 2012, 2013; Walker et al. 2013). The ‘APs approach’ that was applied in the DP (as part of the ADM approach) was inspired by the ‘Route-map / APs approach’ created by the TE2100 Project (as part of its *Dynamic Adaptive Planning approach*) (Jeuken and Reeder 2011, p.5; DP 2011, p.48[[96]](#footnote-96)).

To develop *flexible* *strategies*, the DP used the methods of ATPs and APs (the APs served to develop *strategies*) (Jeuken and Reeder 2011, p.5). The methods of tipping points and adaptation pathways have already been applied in all Subprogrammes (working with adaptation pathways) (Marchand and Ludwig 2014, p.27).

To address Principle 1 of ADM (*linking short-term decisions with long-term tasks*) (DP 2012, p.88, 83; 2013, p.102; 2011, p.49; 2014, p.95, 169; 2016, p.5; Bloemen et al. 2018, p.12, and Klijn et al. 2016, 2015, and Werners et al. 2016, based on Rhee 2012; Marchand and Ludwig 2014, p.8), the Subprogrammes looked ahead into the future and sought to connect/link short-term decisions to long-term tasks, factoring in possible future developments in the choice of measures, and also searching for measures for the short-term that *agree with* the tasks for the long-term (DP 2017, p.22; 2016, p.5; 2011, p.49; 2012, p.83; 2013, p.102). This principle also implied looking at future tasks and using that insight to take *(cost)-effective measures in good time* (DP 2014, p.7, 95), i.e. *the right steps at the right time* (DP 2013, p.102; 2012, p.88), that is adequate measures when they are actually needed to achieve the objectives (Zandvoort et al. 2018; Gersonius et al.2016, p.1), and ensuring that actions taken now are ‘*robust under a changing and uncertain future*’ (Gersonius et al.2016, p.14).

In line with ADM (and its principles), in 2011/2012, some Subprogrammes started to devise *development paths*, by ‘*putting the first steps and the long-term possibilities in a logical chronological order*’[[97]](#footnote-97), and, in this *adaptive strategy*, the measures envisioned for the short-term should leave sufficient scope for different follow-up measures (DP 2012, p.61, 69). The *adaptation paths* provided a powerful way of understanding what measures can be taken and when, and how tasks and options for the long-term affect short-term decisions and measures (DP 2012, p.82).

In line with the 1^st^ principle of ADM, the DP sought to identify measures for the short- and mid-term that *agree with* the long-term tasks (DP 2013, p.102), i.e. decisions to be taken in the short-term were coherently linked to long-term tasks and goals (DP 2017, p.22), short-term measures should be *logical in the long-term* and do not hinder long-term options (Haegen and Wieriks 2015). Thus, each *Preferential Strategy* shows short-term measures linked to options for the mid- (2050) and long-term (2100).

In line with Principle 2 (incorporating *flexibility* into possible strategies, to deal with change and new insights over time (DP 2012, p.88; 2013, p.102; Marchand and Ludwig 2014; Brugge and Bruggeman 2019, p.2), the Subprogrammes sought to build-in *flexibility* in their Strategy in several ways, for example:

- By ensuring (incorporating) flexibility in the *strategy* itself, and / or in the individual measures that compound it. The first involved ensuring the *flexibility* of an own strategy (designed) as a *pathway* made up of sequenced measures; the second involved incorporating *flexibility* into measures themselves (e.g. measures that allow their future adjustment) or using inherently *flexible* measures like sand replenishments (DP 2013, p.102, 103; 2010, p.69; 2014, p.169).
- By working with (designing) multiple potential strategies, i.e. several *adaptation paths* (DP 2013, p.102; 2012, p.81; 2011, p.48).
- Through a stepwise implementation of several measures over time, as changes or new knowledge arise (Werners et al. 2016, p.118, based on Rhee 2012), i.e. *taking measures step-by-step* (DP 2014, p.12, 35, 47).
- The flexibility of a *strategy* can be increased *if measures can be implemented stepwise or if there is the possibility of switching to other measures* (Jeuken et al. 2014, p.18); a phased implementation of various smaller projects is one way of increasing *robustness* (Jeuken et al. 2014, p.15). Importantly, the APs method *per se* involves a stepwise implementation of various FRM / adaptation measures (within each pathway), and it helps to maintain possibilities to switch to other measures over time. Adaptation pathways are successions of measures into the future (Haegen and Wieriks 2015, p.50). However, in the DP, the *flexibility* of some Strategies was, in part, limited by the lifetime and nature of prior measures implemented in the past, namely hard protection measures like flood defence structures that are implemented for many decades (e.g. barriers); in addition, measures appropriate to be implemented in small steps – e.g. ‘river widening’, soft protection measures (like coastal sand nourishments), flood-proofing measures – only contributed partially to some *Strategies* like the *Preferential Strategy for the Rhine Estuary-Drechsteden* (this *Strategy* consists mainly of measures to improve the existing defences (barriers, dykes), however, its *adaptation path* seems flexible in the future because new large-scale structural measures could be postponed (Jeuken et al. 2014, p.18).
- Linking decisions / actions for the short-term to long-term options. In the DP’s *Strategies*, short-term measures are chained with (*coupled with*) long-term possible options (alternatives), and this required envisioning options and possibilities for switching between them through adaptation pathways (Jeuken et al. 2014, p.1). In each *Strategy*, short-term measures are linked to (*coupled with*) long-term options, through adaptation pathways, which explicitly indicate tipping-points/thresholds and when it is necessary to decide to switch from a measure to another (Jeuken et al. 2014, p.24). To devise *flexible strategies* (and cope with uncertainty and complexity), it is necessary to ‘*envision and link possible short-term decisions and long-term options for adaptation and their timing across relevant interfering policy-domains*’ (Jeuken et al. 2014, p.17).
- By keeping options open for the future (DP 2013, p.102, 95; 2014, p.12, 47, 169), not foreclosing future options, avoiding lock-ins. ADM implies keeping options open, this means *leaving options open to be able to respond in a flexible manner to new insights and developments* (DP 2014, p.47), which implies ensuring that *alternative measures are available should they be necessary in the future*, without *ruling out future options* (DP 2014, p.7, 150; 2011, p.8; 45). According to ADM, it is advisable to keep options open for the future, i.e. have *‘alternative measures*’ available for the case they are necessary in the future, namely measures not needed now (DP 2014, p.7, 49; 2011, p.8; 2018, p.13). This required the proactive planning (identification and announcement) of possible measures that may be needed in the future (in the long-term), i.e. options that must be left open for a future decision, namely more drastic measures (DP 2014, p.49; 2011, p.8; 2012, p.81). Flexibility is provided, among other ways, *by leaving options for adjustment* (DP 2014, p.169). ADM aims at *keeping options open and avoiding lock-ins* (Alphen 2015; Zandvoort et al. 2018, p.191); indeed, keeping options open is a basic tenet of ADM (Klijn et al. 2016; Rhee 2012, Zandvoort et al. 2018, p.191). As explained in the *ADM Implementation Guide*, ‘*thinking about the first decision, and potential follow-up decisions in the long-run, is important to be prepared on time for the long-term challenges*’, in addition, ‘*being able to adjust flexibly to changing social and climate conditions is necessary to prevent (…) lock-in and lock-out situations*’ (Rhee 2012, *in* Restemeyer et al. 2017, p. 933). In the development of the DP’s Strategies, and their *adaptation paths*, it was important to keep open options for long-term, i.e. have possibilities to switch to other measures if future climatic or socioeconomic changes, or new knowledge, require so (DP 2013, p.95).
- A key feature of the *Preferential Strategies* is that *it should be possible to take additional measures in the long-term (after 2050) to address the challenges following from climatological and socioeconomic developments*, and *the options for these* should be *ready* and *included in the adaptation paths of the Preferential Strategies* (DP 2014, p.148).
- The Subprogrammes sought to safeguard possibility of taking new measures in the long-term, including them into the *adaptation path(s)* of each *Preferential Strategy*. Each *path* contains options for the long-term that are intentionally kept open due to uncertain future climatic and socioeconomic conditions (Petersen and Bloemen 2015).
- In the DP, uncertainties about future changes were expected to be tackled through an *adaptive way of planning* which should *maximize flexibility* by *keeping options open and avoiding lock-ins* (Alphen 2015, p.310).[[98]](#footnote-98) The development of *flexible strategies* implied finding (*flexible*) measures that do not block future strategies (or further measures that might be necessary) and avoiding irreversible measures (namely over- and under-investments) as far as possible (Jeuken and Reeder 2011, p.2, 1). The APs’ method helped to identify ‘lock-ins’ and how these can be avoided (Alphen 2015, p.313). Flexibility means that decisions and measures planned for the near future should not foreclose future options (to act differently, switch of measure, or add measures) if climatic or societal changes require so; and, it is also important to ensure that external developments in the short-term do not foreclose future adaptation options (Jeuken et al. 2014, p.16). Restemeyer et al. argue that prioritizing measures that prevent lock-ins a condition for making a strategy more ‘adaptive’ (Restemeyer et al. 2017).
- By allowing for switching to other measures, or modifying measures (i.e. ensuring the possibility of shifting to other measures, or adjusting measures) (Vink et al. 2013, p.96-97; Brugge and Bruggeman 2019; DP 2014, p.169); or switching to other strategy (DP 2013, p.77, 95; 2014, p.6; 2012, p.81). A key aspect of ADM is having possibilities (*options*) *for switching between strategies* (DP 2013, p.77). It was necessary *to keep options open for the long-term, so that it is possible to switch to another strategy if future socioeconomic developments or CC should give rise to that* (DP 2013, p.95). ADM allows shifting to other strategies (changing of strategy), but also for *changing strategy* or *stepping up* measures (DP 2014, p.6). To ensure a *sustainable and robust FRM*, the DP sought to choose strategies and measures that gave *flexibility* to respond to new insights and changes, for example, by *stepping up measure*s or *changing* of *strategy* (DP 2014, p.6).[[99]](#footnote-99) As noted in the DP (2012), ‘*to be able to move flexibly from one strategy to another in the future*’, it was necessary to identify what additional measures were required to keep options open for the future (DP 2012, p.81). The DP underlines that, to have flexibility in strategies and measures (to deal with uncertain future developments and to timely respond to changes), it is advisable to operate *strategies that offer chances to switch to other strategies*, and/or carry out measures *that enable their later adjustment or expansion* (DP 2011, p.8, 16, 48-49, 70), or measures that can be *easily accelerated or decelerated* (DP 2010, p.70).
- Possibility of switching from a measure, or a strategy (*path*), to another one. By having several possible measures and pathways available. In the DP, a *Strategy* usually contains *multiple pathways* and allows *switching between different options* over time (Jeuken et al. 2014, p.18, 10)[[100]](#footnote-100); in specific, each *Strategy* has a map with one or more adaptation pathways, and it shows when it is necessary to switch from a measure (action) to another one (Jeuken et al. 2014, p.24). A *Strategy* shows what pathway(s) might be followed, i.e. several *paths*, and such *paths* show diverse measures, including the measures to be taken first and *options ahead* (Jeuken and Reeder 2011, p.5). In each *Preferential Strategy*, there is a map of *adaptation paths* (pathways) that shows what measures need to be taken and when (DP 2014, p.47; 2012, p.82), namely when a change of measure, or strategy (*path*), is necessary or possible (Alphen 2015, p.313).[[101]](#footnote-101)

Overall, the ‘APs method’ *allows for switching between measures along the pathways when needed in view of socioeconomic developments or CC*’ (Haegen and Wieriks 2015, p.56, 50, 54). The APs’ map shows a range of possible measures (options) from which one can choose (Haegen and Wieriks 2015, p.50). The design of *adaptation pathways* implied the identification of various possible measures as *strategic alternatives* (Werners et al. 2016). The flexibility of a strategy could be increased *‘if there is the possibility of switching to other measures*’ (Jeuken et al. 2014, p.18).

- By allowing altering the timing of implementation of measures, e.g. by anticipating / postponing measures in time (Brugge and Bruggeman 2019, p.2), or by *accelerating or slowing down the implementation of measures* (DP 2014, p.136), i.e. through the possibility to speed up / slow down measures (Klijn et al. 2015, p.848; 2016, p.2, and Werners et al. 2016, based on Rhee 2012; Vink et al. 2013, p.96-97), by using measures that can be *relatively easily accelerated or decelerated up to the implementation stage* (DP 2010, p.70). The strategies, and / or measures within them, might be advanced or postponed, and/or adjusted in terms of scope and design (DP 2014, p.136). In the APs approach, a measure can be postponed or advanced in time, as conditions change and new information emerges (Haegen and Wieriks 2015, p.54, 56, 48; Alphen 2013 *in* Zevenbergen et al. 2018; Zandvoort et al. 2018, p.191).
- By using measures that are *robust*. The DP searched for short-term measures that are adequate in the long-term and enhance the system’s robustness and flexibility, making it suited for diverse scenarios (DP 2010, p.69). The DP aimed at developing a robust FRM system that is prepared for various scenarios and able to ‘*withstand the (greater) climatic extremes in a resilient manner*’ (DP 2014, p.6).
- Using measures that are flexible and / or robust. In the DP, *adaptive strategies* could benefit from the inclusion of *flexible measures*, i.e. measures that are ‘*easy to alter, speed up or slow down depending on the measured CC*’ (e.g. nature-based measures like beach nourishment, or vegetated foreshores in front of defences, which are more flexible than ‘fixed’ hard defences); or, on the other hand, in some cases, it might be preferable to use *robust measures* (that can deal with high-end scenarios), namely where *‘the cost of adjusting a measure later is high compared to a more robust initial design*’ (Alphen 2015, p.313).
- By using a variety of measures, i.e. diverse types of measures (DP 2014, p.169). The Dutch philosophy on FRM has widened with the introduction of the concept of *Multi-layer Flood Risk Management*, leading to greater *flexibility* and robustness (Haegen and Wieriks 2015). A large heterogeneity of measures, and some ‘planned redundancy’ in solutions, help to increase the robustness and resilience of the FRM system (the risk of failure is distributed over many elements of the system, thus, if one solution fails, the whole system does not fail) (Jeuken et al. 2014, p.15). [[102]](#footnote-102)
- By taking low-/no-regrets measures first. The DP sought that the first steps (first measures taken) were ‘no-regret measures’ (measures that are worthwhile in any scenario) (DP 2012, p.88). Having the long-term vision in mind, the DP sought to prioritize no-regret actions (and s*cenario analysis* was useful to assess the robustness of measures under various scenarios) (Marchand and Ludwig 2014, p.2, 11).[[103]](#footnote-103) In the implementation of an *adaptive plan*, decisions in the short-term should be used to implement first ‘low-regret actions’ (Jeuken et al. 2018, p.19).
- By allocating land that might be needed for measures in the long-term, or setting it for long-term spatial objectives or temporary uses (DP 2010, p.70). The DP defined measures (for the near future) necessary to keep options open in the long-term, namely *spatial reservations* necessary to keep ‘*river widening’* measures open for a long time (Jeuken et al. 2014, p.18).
- According to Principle 4 (*linking FRM and FS measures with other investments and agendas of other actors or sectors*) (DP 2014, p.169, 95, 47; 2012, p.88; 2013, p.102; 169; 2011, p.49; 2010, p.68; 2016, p.5; Marchand and Ludwig 2014; Gersonius et al. 2016; Zandvoort et al. 2018; Brugge and Bruggeman 2019; and, based on Rhee 2012, Werners et al. 2016; Klijn et al. 2016, 2015), the Subprogrammes sought to link FRM measures to other agendas and investments. The Teams sought to link FRM measures envisioned, as far as possible, to other objectives and ambitions in the area (e.g. regarding space or nature), as this can contribute to more innovative, efficient and sustainable solutions, in a *comprehensive / integrated approach* (DP 2014, p.47); the *paths* helped to detect linkages between the measures devised and other investments, agendas, and policy-objectives (DP 2013, p.56). By interlinking agendas and investments, measures could become cheaper and easier to implement (by sharing costs or reducing impediments), yield added value and reduce regret (as other benefits could be achieved) (Marchand and Ludwig 2014; Brugge and Bruggeman 2019; Klijn et al. 2015, 2016).
- By combining agendas, and mainstreaming FRM / adaptation measures into decisions and investments in the short-term. The identification of opportunities to combine the measures envisioned with other agendas and multi-objective investments was one way of increasing flexibility of the *Strategies* (DP 2011, p.48; Jeuken et al. 2014, p.1, 19). The DP sought to ensure the integration of objectives (FRM and FS objectives integrated with spatial planning, spatial adaptation, and nature conservation) (Jeuken and Reeder 2011). It also sought to ensure the integration of agendas (in accordance with the 4^th^ principle of ADM). The combination of measures with other objectives (through e.g. multifunctional flood defences) helps to increase public acceptance of measures and reduce costs (brings added value), but implies the synchronization of different investment agendas, new technical requirements for designing and maintaining defences, and multi-actor arrangements (Alphen 2015). The DP also sought to link short-term decisions to long-term goals (in accordance with the 1^st^ principle of ADM). In the DP, long-term climate adaptation needs were considered in short-term decisions, e.g. in the definition of the new flood protection standards; in areas where dykes need to be reinforced for maintenance, the projected water level rise associated with CC must be considered in their design; works to increase the sluice capacity for shipping must provide an extra drainage capacity (Jeuken et al. 2014).

These were the main ways of embedding *dynamic robustness* and *flexibility* into the DP’s Strategies. These aspects contributed to incorporate and safeguard the *robustness* and *flexibility* of *Strategies*.[[104]](#footnote-104) These types of *flexibility* also helped to prevent over- or under-investments and underperformance (Klijn et al. 2015; 2016, based on Rhee 2012; Brugge and Bruggeman 2019, p.2), and, thus, maladaptations. With its *flexible adaptive approach*, the DP sought to ensure the possibility of shifting to other measures, or modifying them, or switching to other strategies, or anticipating / delaying measures, and, in this way, safeguard that *‘not too many measures, nor too few, and not (…) too early, nor too late*’ are taken (Vink et al. 2013, p.96-97).[[105]](#footnote-105)

Overall, ADM seeks and values *flexibility* in comprehensive strategies and in individual measures, and this required incorporating and appraising *flexibility* in measures and pathways (Rhee 2012, *in* Klijn et al. 2015; 2016 and Werners et al. 2016). Ultimately, Principle 2 aimed at developing *flexible strategies* (Bloemen et al. 2018, p.12).

With its *flexible adaptive approach*, the DP has valued *flexibility* mainly in terms of possible strategies and their possible timing of implementation (Haegen and Wieriks 2015; Zevenbergen et al. 2018), i.e. in terms of the possibility of switching to other measures or to other strategies (or modifying a measure), and possibility of anticipating / delaying measures (Vink et al. 2013, p.92).

The DP underlines that, in accordance with ADM, it is wise and necessary to have flexibility in strategies and measures, to deal with uncertainties around future CC and socioeconomic developments and to allow a timely response to changes (DP 2011, p.16, 48-49, 70, 8). In ADM, strategies and measures must ensure flexibility in the way they respond to new measurements and insights, for example, by intensifying efforts if necessary or by changing strategy, and at the same time, envisaging possible future measures (Petersen and Bloemen 2015). ADM seeks to be able to ‘*temporize*’ (speed up / postpone) measures, or change of measure or strategy, if the actual or expected rate of climatic and socioeconomic developments indicates this is necessary (Dessai and Sluijs [2007](https://link.springer.com/chapter/10.1007/978-3-030-05252-2_14#CR12); Buuren et al. [2013](https://link.springer.com/chapter/10.1007/978-3-030-05252-2_14#CR46); *in* Marchau et al. 2019). Thus, the designed strategies (*adaptation paths*) had to be flexible in terms of: timing (possibility of postponing / advancing measures in time), possibility of choosing another measure or strategy, and avoidance of lock-ins (Zandvoort et al. 2018, p.191).

- Overall, in the APs method, *flexibility* is related with: (a) the possibility (flexibility) to switch to other measures or pathways if and when needed, in the face of uncertain future developments, and thus, with the availability of other possible measures (options/alternatives); and (b) flexibility in relation to the timing of implementation of measures (the possibility of altering the moment for implementing a measure) (Haegen and Wieriks 2015, p.54, 56, 48; Alphen 2013 *in* Zevenbergen et al. 2018; Zandvoort et al. 2018, p.191).

In the DP, one of the four basic principles (*essential aspects* / *key-points*) of ADM was ‘*working with multiple strategies between which it is possible to alternate*, *i.e.* *with* *adaptation paths*’ (DP 2012, p.88, 81; 2013, p.102; 2011, p.48).[[106]](#footnote-106) This implied the design of adaptation pathways (as sets of sequenced measures) that together provided a general *adaptive strategy* (Haegen and Wieriks 2015).

The 3^rd^ principle of ADM (*working with* *multiple strategies that can be alternated between, i.e.* *adaptation paths*) (DP 2012, p.88; 2013, p.102; Marchand and Ludwig 2014, p.8; Gersonius et al. 2016; Rhee 2012 *in* Werners et al. 2016) required developing multiple strategies that can be implemented alternatingly depending on developments, i.e. designing several adaptation pathways, called ‘*adaptation paths*’ in the DP, between which it is possible to switch depending on developments. This principle implied using the method of Adaptation Pathways (APs) to design the strategies (Brugge and Bruggeman 2019).[[107]](#footnote-107)

The 3^rd^ principle of ADM involved *working with several potential strategies* – i.e. with ‘*adaptation paths*’ – and appraising the *flexibility* around such *paths* (DP 2011, p.48). The 3^rd^ principle of ADM has translated into / has reflected in the design of *adaptation paths*’ in each *Preferential Strategy* of the DP (DP 2012, p.81; 2011, p.48; 2014, p.47). In line with this principle, a map with one or more *adaptation paths* was designed in each regional *Strategy* (thus, each *Strategy* presents a map of *adaptation paths*) (DP 2014, p.47; 2012, p.81; 2011, p.48). The *paths* contain diverse measures sequenced over time, which can be implemented alternatively according to the changes that occur (Marchand and Ludwig 2014, p.8).

Each *Strategy* contains a map of *adaptation path(s)*; the term ‘*adaptation path*’ refers to an ‘adaptation pathway’; an *adaptation path* is a logical set of measures that includes measures for the short-term (namely *no-regrets measures*) and options for the mid- and long-term (DP 2013, p.56).

Thus, each *Strategy* contains *sets of measures*: each *set of measures* offers a logical *path*, and *sets of measures* (i.e. several paths) were elaborated separately for each Region (DP 2013, p.56, 95; 2012, p.81).[[108]](#footnote-108) Each *Preferential Strategy* delivers *a set of measures* which includes measures for the short-term (planned with a fair level of certainty) and options for the long-term (DP 2013, p.95).[[109]](#footnote-109)

In accordance with ADM, in each Subprogramme, *various adaptation paths* (initially called *development paths*) were developed, which required studying the circumstances under which it will be logical to move from one *path* to another and options to keep open to allow such transitions (DP 2012, p.81), i.e. laying down the conditions under which changing or switching of measure or strategy (*path*) is reasonable and possibilities for switching between strategies (DP 2013, p.95, 77), that is, looking at the conditions under which it is wise to shift from a strategy, or from a measure, to another one (DP 2011, p.48; Gersonius et al.2016, p.205).

Each *Preferential Strategy* of the DP presents objectives, measures to achieve the objectives, and the associated *adaptation path(s)* (i.e. a set, or sets, of measures) displayed in a map (DP 2013, p.56; Bloemen et al. 2018; Alphen 2015; Rhee 2012, p.18 *in* Zandvoort et al. 2018, p.190).[[110]](#footnote-110)

Thus, each *Strategy*[[111]](#footnote-111) (of each Subprogramme) contains a map with one or more *adaptation paths* (DP 2013, p.56).

All *Preferential Strategies* contain a map with *adaptation path(s)*; each regional Subprogramme drew up a *Preferential Strategy* for FRM for its region (DP 2014, p.8, 46).

In each *Preferential Strategy*, there is a map of ‘*adaptation path(s)*’ (DP 2013, p.56; Zandvoort et al. 2018, p.190).

Each path (pathway) provides a sequence of actions over time to achieve the predefined objectives (Marchand and Ludwig 2014, p.2, 13, based on Haasnoot 2013); the various paths offer ‘*coherent sequences of measures and potential options, which may be triggered before an ATP occurs*’ (Zevenbergen et al. 2018, based on Haasnoot et al. 2012, 2013).

In the DP 2014, a map of *adaptation path(s)* is presented in each *Preferential Strategy*, i.e. each *Preferential Strategy* contains a map with one or more *adaptation path(s)*, which shows the pathway(s) to be followed. Such map indicates which measures can be taken (including measures required now to ensure that options that may be needed in the long-term can be implemented by then) and when they are expected to be necessary (DP 2014, p.47); in specific, the map displays the possibilities (options) for switching of measure or *path*, and it also identifies short-term measures required to be able to change strategies later, if necessary (DP 2013, p.95, 77). Each *Preferential Strategy*, in its *adaptation path(s)*, includes measures for the short-, and options for the mid- and long-term (which may be used after 2050) (DP 2014, p. 49, 148), which implied looking at the conditions under which it is wise to shift from a strategy, or from a measure, to another one (2011, p.48; Gersonius et al.2016, p.205)

In line with ADM, a map with one or more *adaptation paths* was designed in each of the *Strategies* elaborated (DP 2012, p.81), it provides a schematic overview of the measures needed in a certain region (DP 2011, p.48). Thus, each *Preferential Strategy* presents a map of ‘*adaptation path(s)*’, which shows, from the current situation and moving ahead into the future, the various measures available for the short-term and possible options for the mid- and long-term (including possible adjustments / adaptations or amendments that might be necessary in strategies), which required looking at the conditions under which it is advisable (wise) to shift from a strategy, or measure, to another one (DP 2011, p.48; Gersonius et al.2016, p.205). For instance, the Subprogramme for the Rhine Estuary-Drechtsteden, in its *Preferential Strategy for FRM*, developed an *adaptation path* which indicates diverse measures and at what times these will be required (DP 2012, p.90).[[112]](#footnote-112)

In line with the 3^rd^ principle and 2^nd^ principle of ADM, each Subprogramme developed *multiple strategies that can be alternated between*. i.e. several *adaptation paths*, and sought to incorporate and valued (*appreciated* and appraised) the *flexibility* in strategies (DP 2013, p.102; 2011, p.48). In each *Preferential Strategy* (in its map of *adaptation paths*), depending on climatic and socioeconomic developments, measures may be taken sooner or later (DP 2014, p.47), and it may be necessary to switch of measure or strategy (*path*) (DP 2013, p.77, 95, 103; 2014, p.6, 169). In the DP, the APs approach allows for shifting (switching) between different measures or *paths* (i.e. switching from a measure, or *path*, to another one), if necessary, in view of climate change or socioeconomic developments, and (helped)seeks to ensure that options are kept open for the long-term (Bloemen et al. 2018, p.12, 14; Haegen and Wieriks 2015, p.56, 50, 54).

Overall, the *adaptation paths* of the DP contain diverse measures sequenced over time which can be implemented according to the real changes that occur (Marchand and Ludwig 2014, p.8).

To design the adaptation pathways, the method of Haasnoot et al. (2012, 2013) could be used; pathways could be designed as series of sequenced measures that provide a solution to problems (*in* Brugge and Bruggeman 2019). To develop *adaptation paths*, the Subprogrammes used the ATPs and APs methods (Zandvoort et al.2018); the design of the *adaptation paths* implied the identification of tipping-points (Buuren et al. 2016). Each Subprogramme elaborated one or more *adaptation paths* for its *Preferential Strategy*, and this implied defining the conditions under which changing of measure is necessary (DP 2013, p.95).

The design of *paths* implied the consideration of *Adaptation Tipping-Points* (ATPs, conditions under which the current or other management / policy measure fails, and a new measure is required, see Kwadijk et al. 2010); ATPs were essential for phasing the possible measures within each *Strategy* – successive measures were sequenced to assemble pathways that show short-term measures chained with (linked to) long-term options (Jeuken et al. 2014, p.17). To design the pathways, the method of ATPs was applied: once an ATP was in sight, a new measure was needed (Haegen and Wieriks 2015, p.51). Yet, in the DP’s Strategies, relevant decision-moments and implementation-moments not only depend on critical ATPs and on ATPs of measures and, but also on earlier opportunities to combine measures for different purposes (thematically and spatially) (Jeuken and Reeder 2011, p.5).[[113]](#footnote-113)

The basic idea underlying the APs approach (within ADM) is *to generate a wide array of pathways* (each pathway *consisting of a series of measures*) *through which the policy-objectives are achieved under changing climate and socioeconomic conditions* (Deltares 2018, p.4, 3). The set of possible pathways mapped out in an AP’s map provides a range of adaptation options that may be used in future planning and policymaking, in this way, the APs approach helps to enhance the plan’s and the system’s *flexibility* and *adaptability* (capacity to adapt to multiple plausible future changes and conditions) (Deltares 2018, p. 4, 2).

ADM utilizes the APs method as a *flexible, adaptive approach* for designing strategies under plausible future scenarios, which *allows for switching between measures along the pathways* if necessary, *in view of socioeconomic developments or CC* (Haegen and Wieriks 2015, p.56, 50, 54). A key step of the ADM process is *to develop a set of measures along adaptive pathways* (Haegen and Wieriks 2015, p.55).

In sum, to create ‘*strategies*’, the ADM process builds on the construction of pathways, and this implied scanning options to keep open, and assessing the flexibility of measures and pathways – these aspects were patent in the *Strategies* for FRM of the DP (Zandvoort et al. 2018, p.189, 191).[[114]](#footnote-114)

The designed *adaptation paths* were assessed under different scenarios (Zandvoort et al. 2018, p.191). In accordance with ADM, it was important to assess the *robustness* and *flexibility* of the proposed strategies (*paths* and their individual measures) against several scenarios (Gersonius et al. 2016, p.4).[[115]](#footnote-115) As mentioned, a *Preferential Strategy* should be both *robust* and *flexible*; and each Subprogramme should assess if its *Strategy* had these features, among others (DP 2013, p.94 95).

The *Preferential Strategies* were developed on the basis of the four *Delta Scenarios* (DP 2014, p.136), with 2100 as time-horizon (Alphen 2015), but they are, by nature, *adaptive*: they can be adapted *to* *slower and faster CC* than expected in the *Delta Scenarios*. The *adaptive nature of the strategies* makes it possible to act according to evolving conditions and changes, for example, *by accelerating or slowing down the implementation of measures* (e.g. the number and amount of sand replenishments per year along the coast can be adjusted to the measured SLR) (DP 2014, p.136). Based on new knowledge and monitoring, the strategies, and / or measures within them, may be advanced or postponed, and adjusted in terms of scope and design (DP 2014, p.136).

The main outcome of ADM is a single pathway, or a set of pathways, with which it was possible to schedule measures in the face of uncertain future change – that is, ADM ‘*leads to a composite strategy, or a set of alternative strategies with intermediate possibilities for revisions*’ (Rhee 2012, p.14, *in* Zandvoort et al. 2018, p.191).

The APs method helped (contributed) to ensure and safeguard *robustness* and *flexibility* in the DP’s Strategies. With the APs approach, the Subprogrammes could envision alternative measures and possibilities for switching between them through adaptation pathways, and link measures for the short-term to long-term possible alternatives (options) (Jeuken et al. 2014, p.1). The APs approach served to explore possible measures and options, and possibilities for switching from one option to another in the future, and, in this way, remain flexible – this contributed to enhance the *flexibility* of each general Strategy. By using the APs, it was possible develop a ‘*robust and flexible*’ set of measures – or, more precisely, *sets* – each *set* as an adaptation pathway. The maps of *adaptation paths* identify/show the measures available, including options for switching in the future, and when it is necessary to switch of measure (Jeuken et al. 2014, p.10).

The pathways deliver *flexibility* in several ways: the strategies can be accelerated, slowed down, or adjusted, by using other options that are previously kept open (i.e. by shifting from one measure to another which is available) (Gersonius et al. 2016 p.212). The APs method, served to develop a ‘*robust flexible strategy*’ for FRM; the development of APs ensures flexibility, since the pathways can be accelerated / slowed down, or adjusted (through shifts from a measure to other), and options to keep open are shown (Gersonius et al. 2016 p.212, 209).

Overall, as mentioned, the *Preferential Strategies* were developed based on the ADM approach and its four basic principles[[116]](#footnote-116) (DP 2014, p.47, 12; 2017, p.7; 2011, p.18). Such principles are patent in the *Preferential Strategies* in several ways, for example:

- P1. In accordance with P1, the strategies, as *adaptation paths*, connect short-term decisions and measures to long-term tasks (DP 2013, p.56),
- P2 is ensured by *taking (the most cost-effective) measures step-by-step*, and by *leaving options open for the future* to deal with changes and new knowledge (DP 2014, p.47).
- P3 is reflected in the design of *adaptation paths* as *part of the Preferential Strategy*. A map of *adaptation paths* was designed in each *Preferential Strategy*, it indicates *what* and *when* measures are expected to be necessary (including measures required now to ensure that the options that may be necessary in the long-term can be implemented by then); and, depending on the actual changes and climatic and socioeconomic developments, measures may be taken sooner or later (DP 2014, p.47), or changed or switched.
- P4. The Teams sought to link FRM measures envisioned to other objectives and ambitions in the area, in an *integrated approach* (DP 2014, p.47); the *paths* helped to detect linkages between the measures devised and other investments, agendas, and policy-objectives (DP 2013, p.56).

It can, therefore, be deduced that developing a ‘*robust and flexible*’ set of measures, to deal with uncertain future conditions and changes, (with) through the method of ‘Adaptation Pathways’ (APs) is another element of ADM that has been essential to craft and operationalize each strategy as a *robust adaptive strategy/plan*, and the DP itself as a *robust adaptive* policy-programme (as also argued by Jeuken et al. 2014, p.23, 1, 3). More precisely, the development (design) of *robust flexible strategies* – each strategy as a *robust flexible* *set* of measures – by using the *APs approach* (see Haasnoot et al. 2012, 2013) – can be deemed a key-element of the DP’s ADM approach fundamental to create and carry out an *adaptive policy-programme* (in line with the elements proposed by Jeuken et al. 2014, p.23, 1, 3, and by Restemeyer et al. 2017, p.921, 922-925, 935[[117]](#footnote-117), and as suggested in the study on the DP of Jeuken and Reeder 2011, p.2, 5[[118]](#footnote-118)).

**BOX. Theoretical grounds of *Adaptive Planning approaches***

In the light of the ***Adaptive Planning paradigm*,** under deep uncertainties about future changes, a planner must develop a ‘*dynamic adaptive plan/policy*’ (Jeuken et al. 2014, p.2).

The APs approach is defined as a (methodological) ***approach for exploring and sequencing a set of possible actions*** (i.e. a set of possible measures) *based on alternative external developments over time* (Haasnoot et al. 2013, p.485). Haasnoot et al. (2012, 2013) and Walker et al. (2013) place the APs within family of Adaptive Planning approaches. The APs offers a methodological approach for designing a *dynamic adaptive plan* that is both *robust* and *adaptive* under uncertain future change.

- In an Adaptive Planning approach, *flexibility* is a characteristic of the general plan / strategy, which enables it to deal with uncertain future changes, in specific, decisions and actions in the near future should **not foreclose future options (to act differently, to switch of action or to add actions)** if climatic and socioeconomic changes demand so, and, on the other hand, developments occurring in the near future should not foreclose future adaptation options (Jeuken et al. 2014, p.16).
- The development of *flexible strategies* implies finding (*flexible*) measures that do not block future strategies (or further measures that might be necessary) and avoiding irreversible measures (namely over- and under-investments) as far as possible (Jeuken and Reeder 2011, p.1, 2).
- The flexibility of a plan / strategy can be increased *‘if measures can be implemented stepwise, or if there is the possibility of switching to other measures*’ (Jeuken et al. 2014, p.18).
- The flexibility of a *strategy* can be increased *if measures can be implemented stepwise* (Jeuken et al. 2014, p.18); a phased implementation of various smaller projects is also one way of increasing *robustness* (Jeuken et al. 2014, p.15).
- To devise *flexible strategies* (and cope with uncertainty and complexity), it is necessary to ‘*envision and link possible short-term decisions and long-term options for adaptation and their timing across relevant interfering policy-domains*’ (Jeuken et al. 2014, p.17).
- A large heterogeneity of measures, and some ‘planned redundancy’ in solutions, help to increase the robustness and resilience of the FRM system (the risk of failure is distributed over many elements of the system, thus, if one solution fails, the whole system does not fail) (Jeuken et al. 2014, p.15).
- In the implementation of an *adaptive plan*, decisions in the short-term should be used to implement first ‘low-regret actions’ (Jeuken et al. 2018, p.19). Restemeyer et al. argue that prioritizing measures that prevent lock-ins a condition for making a strategy more ‘adaptive’ (Restemeyer et al. 2017).

Drawing on insights and literature on resilience, adaptive planning and governance, Restemeyer et al. have defined (proposed) these 3 theoretically-defined conditions for making a long-term FRM policy / plan more *adaptive* (Restemeyer et al. 2017, p.921, 935). Restemeyer et al. argue that **three conditions/points are necessary and crucial for making long-term plans / policies ‘*adaptive*’**: (a) *an agile governance process* (this implies multi-level, multi-sector, and multi-actor governance arrangements that foster learning, i.e. learning-oriented governance arrangements, as well as *capacity to steer towards a desired direction* and *capacity to adjust based on new insights)*; **(b) make *flexible strategies and plans***.; and (c) prioritize measures that prevent lock-ins (Restemeyer et al. 2017, p.922-925, 935). According to Restemeyer et al., for making long-term plans *adaptive*, and, especially, for creating *flexible strategies and plans*: recent literature indicates three tools (methodological approaches): scenarios, tipping-points, and adaptation pathways (which requires ordering various possible measures in time) (Restemeyer et al. 2017, p.923).

Drawing on their analysis of the Dutch DP and its ADM approach, the authors demonstrate that Dutch policymakers face a dilemma between *adaptability and the urge to control*. To overcome it, the authors suggest a stronger focus on monitoring and learning to strengthen the adaptability of long-term policies, as well as a stronger engagement of local stakeholders (Restemeyer et al. 2017, p.920, 935-936).

As underlined **by Restemeyer et al. (2017)**, a key-question that remains is *how to make long-term policies adaptive so that they are able to deal with uncertainties and changing circumstances* (Restemeyer et al. 2017, p.920). As noted by the authors, in recent years, various authors have called for *adaptive planning and management approach* (e.g. Holling 1978; Folke et al. 2005; Pahl-Wostl 2006; Wilkinson 2011b; Innes and Booher 2010, *in* Restemeyer et al. 2017, p.921), however, what this means (in practice) for elaborating long-term policies has only recently been put on the research agenda (Reeder and Ranger 2011; Walker et al. 2013; Haasnoot et al. 2013); in addition, the focus has been mainly on techniques and tools to make strategies more flexible (e.g. ‘adaptation pathways’, see Haasnoot et al. 2013), without paying much attention to the underlying conditions required for achieving adaptability (and making an adaptive policy/plan), namely in the content of strategies and the governance process in which strategies are made and implemented. Restemeyer et al. (2017), in their paper, sought to address this issue, by defining three theoretically-based conditions / points for making long-term policies *more adaptive* (Restemeyer et al. 2017, p.921).

Restemeyer et al. (2017) also draw some lessons for planning research and practice: 1) it is necessary to investigate ways to embed learning and monitoring in the policy process (before developing strategies), ‘collaborative monitoring’ might be a possible way forwards, but further research is necessary to define and detail such approach; 2) among the techniques and tools to make strategies more flexible, the Adaptation Pathways and Tipping points have been suggested by various authors (e.g. Reeder and Ranger 2011; Walker et al. 2013; Haasnoot et al. 2013) as being useful to think about the long-run, but they are still quire complex and abstract for policymaking on a national and regional scale, and developing adaptation pathways on a local scale seems more feasible (e.g. specific measures can be discussed in depth and better suited to the physical, social and political context); 3) it is very difficult to prioritise measures that avoid lock-ins when the system is already in a lock-in (as shown by the studied case), thus, it is important to find ways to minimize or overcome such lock-in; 4) the pre-existent institutional context matters much in the uptake of adaptive planning and management approaches and in the shift towards adaptability. The authors point out important issues for further research, namely: exploring **how the adaptability discourse has unfolded in other (national) contexts**, and **which conditions and policy arrangements are advantageous or disadvantageous for applying an adaptive approach** (as moving *adaptability* from theory into practice is still key challenge for the future) (Restemeyer et al. 2017, p.936).

#### Key-element 4

As an adaptive approach, ADM entails monitoring, revaluation, and adaptation: ADM requires an ongoing monitoring of external conditions (climatic and socioeconomic changes), of implemented and planned strategies, and the (re)evaluation of strategies (applied or planned) to check if they are proceeding as expected or if it is necessary to adjust them (their *pace*, *direction* or *content*) (DP 2016, p.6; 2014, p.149). As new insights into climatic, socioeconomic and technological changes arise, it might be necessary to adjust strategies (DP 2016, p.6). Monitoring is also needed to detect when an ATP may be reached (this implies keeping track of external changes and effects of measures) (Restemeyer et al. 2017, p.931). ADM involves staying *flexible* to be able to act on new conditions and insights (able to adapt to change) (DP 2014, p.7), which requires *looking ahead to the taskings that are facing us, setting down the measures required in concert* (by *keeping option open*), and also *persistently checking whether we are working at the right pace and in the proper direction*, and, *if need be, adjusting our strategies in time* (i.e. adjusting the pace or course, if necessary, as changes or new knowledge arise) (DP 2018, p.13; 2017, p.7, 13; 2016, p.6). ADM not only requires anticipating future circumstances while choosing *what, when* and *where* measures to implement, but also *adjusting strategies periodically* based on new knowledge, and this implies *a clear view of the possibilities for the future at all times* and ongoing monitoring and regular evaluation – *monitoring and evaluation are essential to know in good time when the strategy has to change and when other measures that have already been prepared should be put into effect* (DP 2014, p.13). In DP, ADM required a proper monitoring of changes and developments (namely climatic and socioeconomic developments), of new insights, and of implemented measures (i.e. of *what we are doing and what results this yields*, that is, *what is being done, with what effects*), and, based thereon, a regular (re)evaluation of the strategies followed (implemented and planned) (DP 2014, p.149; 2016, p.6).

Overall, in the DP, ADM required the monitoring changes or developments, new knowledge, and effects of measures, and the regular reassessing/reviewing the Plan – its Strategies (planned or implemented), their measures, or their schedule – and adapting / adjusting them if changes or new insights prompt so (DP 2016, p.59, 11).

The ADM cycle presupposes the existence of a monitoring and evaluation system and a *joint learning process* – and the DP’s ADM requires reviewing choices, strategies and plans, and adapting/adjusting them if this is prompted by changes or new insights (DP 2016, p.59, 11, 61, 6). Such monitoring and revaluation system should observe how climatic, physical, and socioeconomic conditions are changing, and ensure that ‘*response to these changes is an adaptive one’* (DP 2014, p.9). Monitoring and evaluation are necessary to ‘*know in good time when the strategy has to change and when other measures that have already been prepared should be put into effect*’ (DP 2014, p.13).

- As a *flexible approach*, ADM requires continuous monitoring and evaluation (Zevenbergen et al. 2018, p.6). As noted by Zevenbergen et al., in a *flexible adaptive (management) approach*, flexibility depends on the *capacity of the decision-makers to learn from the arrival of new information and their willingness and ability to revise investment decisions based upon that learning* (Zevenbergen et al. 2018, p.1).
- The DP’s ADM approach is *flexible* enough to adapt the plan / strategy to unexpected or changing conditions and opportunities that might arise, and, at the same time, able to support the planning of investments in the short- and mid-term under deep uncertainty about future changes (Brugge and Bruggeman 2019, p.1).
- In the DP, the ADM approach requires the monitoring of changes and their effects, and, based on it, the regular evaluation of the strategies followed (and new insights must be used in such revaluation) (Petersen and Bloemen 2015, p.225). As noted by Petersen and Bloemen, ADM is based on an approach of *Planned Adaptation* that implies two components in a policy-making process: commitment to submit the plan to ongoing revaluation, and systematic effort to use new information in that revaluation (Petersen and Bloemen 2015, p.222). A policy-programme should *‘plan for future changes in knowledge, by producing new knowledge and revising rules at regular intervals*’ (Petersen and Bloemen 2015, p.221, 223). By introducing its monitoring and evaluation programme, the DP safeguarded future revisions of the new approach (ADM) and of its *Strategies*, at regular intervals, and brings science, engineering, and public policy closer (Petersen and Bloemen 2015, p.222).
- Overall, the DP’s ADM approach builds on ongoing monitoring, and on optimizing the system in relation to predefined goals (and goals tend to be seen as unchangeable, although the natural system is dynamic and complex) (Zandvoort et al. 2018, p.193, 190). Moreover, in the DP’s ADM, uncertain future changes, natural variability of the weather and climatic extremes, demanded a regular reassessment of strategies and measures (Zandvoort et al. 2018, p.192). In the DP, the evolution of climatic, physical, and socioeconomic changes / developments, and the progress of scientific insights, must be carefully monitored, namely, to evaluate if measures should be taken earlier or postponed (Jeuken and Reeder 2011, p.7).

**The role of monitoring and reassessment (evaluation) within the general ADM approach and process**

As illustrated in the ADM process (illustrated in a diagram, for example, by Deltares 2018), **Step 4** requires the specification of indicators (signposts) and triggers (critical values at which it is necessary to activate actions, or make adjustments or change of measure; triggers act as warning signals for the implementation of actions or reassessment of the plan, and must be monitored) (Deltares 2018, p.2-3; Jeuken et al. 2014, p.4-5; Haasnoot and Jeuken; Marchand and Ludwig 2014; Brugge and Bruggeman, p.4-5). Thus, **Step 4b** implies the definition of a monitoring system that collects information on indicators and triggers (Haasnoot and Jeuken) and mechanisms to adjust the plan (e.g. its strategies) if necessary (Deltares 2018, p.2-3): in specific, **Step 4b** implies the **definition of a monitoring and evaluation system that** specifies ‘triggers’ (critical points beyond which it is necessary to make adjustments to the strategy or shift to another measure) and ‘preparatory actions’ (required to enable long-term options, e.g. adjustments in rules / legislation, research, spatial reservations, etc.) (Gersonius et al. 2016, p.204-206).

Moreover, in the ADM process, **Step 6** consists of the monitoring and evaluation of external changes, implemented measures, and progresses in knowledge, and the reassessment of the Plan, and, if necessary, its review (Haasnoot and Jeuken); this requires a monitoring and evaluation system to keep track of climatic and socioeconomic developments, and assess if and when it is necessary to review or adjust the plan (e.g. change its strategies or measures) (Deltares 2018, p.2-3). At this step, the monitoring and evaluation system must keep track of climatic, physical, and socioeconomic trends and developments, namely, to identify when it is necessary to adjust a strategy (e.g. to shift from a measure to another) (Deltares 2018, p.5). Variables that may induce to triggers must be monitored (Jeuken et al. 2014, p. 4). **In this step**, the monitoring and evaluation system is conducted: it tracks climatic and socioeconomic changes relevant for adapting the plan’s strategies or measures, e.g. for anticipating / delaying the implementation of measures; e.g. if SLR unfolds at a faster rate than expected and a tipping-point is expected to be reached earlier, it may be necessary to implement a measure earlier than projected (if SLR evolves slower, then, the measure can be delayed) (Brugge and Bruggeman, p.4-5). The monitoring system indicates the rate and direction of risks and their implications for adjusting the plan, it allows the adjustment of the plan - its measures or their timing (Brugge and Bruggeman, p.4-5). **Step 6** is essential to allow a real adaptive planning and management.

Since 2015, the DP has developed – defined and set – its own monitoring and evaluation (and reassessment) system / programme: the *Monitoring, Analysing, Acting system* (MAA), which is deemed the *engine of ADM* (DP 2016, p.6, 59; 2014, p.149).[[119]](#footnote-119) The MAA was developed after 2015, and it started to operate in 2017 (DP 2016, p.3, 63). The MAA serves mainly to:

- monitor changes and developments, measures’ effects, new insights and knowledge that may emerge, and, in accordance, regularly revaluate (review) the strategies and measures (implemented and planned) (DP 2016, p.6, 9, 11, 59).
- collect information necessary to examine whether and how (and when) the *Preferential Strategies* need to be adjusted, and to operate an adaptive approach. The MAA generates insights into the progresses made and into changes / developments that may constitute reasons for adjustment (*to adjust the pace or the direction*) (DP 2016, p.6, 9, 11). The MAA must keep track of (external and internal) developments to enable a timely adjustment of strategies (e.g. a change of course or measure), thus, it is indispensable to ensure the DP’s adaptive approach (DP 2017, p.13; 2016, p.59).

The MAA underpins the DP’s ADM approach; i.e. in the DP, ADM is systematically substantiated in the MAA (DP 2016, p.9, 6).

Through the MAA, the DP examines whether and how the *Preferential Strategies* need to be adjusted (DP 2016, p.7).

More precisely, with the MAA, the DP aims to: evaluate (review), every year, whether any new developments demand the adjustment or fine-tuning of the *Preferential Strategies* or *Delta Plans*; and check whether any new conditions, developments or changes impact the goals and the measures defined, and if necessary, adjust them (DP 2016, p.6, 7).

The results of the MAA system must be used to update the DP on observed and forecasted effects and must be considered in any revision of the programmed measures and policy (namely of *implementation programmes*) (DP 2014, p.149-150).

In 2017/2018, the *Signal Group* defined 8 *indicators* to be monitored in order to detect signals that may prompt an adjustment of the *Preferential Strategies*, and / or developments that could jeopardise the attainment of goals, e.g. SRL, extreme river discharges, land use, etc. (DP 2018, p.111; 20).

The DP (2018) mentions that ‘*in the event of new insights showing that the current course will not enable achievement of the FRM, FS, and spatial adaptation goals by 2050, the Preferential Strategies may need to be adjusted*; actually, the *Preferential Strategies* are *open to annual adjustment* (DP 2018, p.22).

Besides this, the DP carries a review every 6 years to check (based on monitoring results) if it is necessary to maintain or adjust the *course* or the *pace* (i.e. the measures or their timing) (DP 2016, p.6). That is, every six years, the DP carries a systematic review to check ‘*whether it managed to keep up the pace and adjust the course on time*’ and analyse if the monitoring results indicate that it is necessary to maintain or adjust the *course* or *pace* of Strategies (i.e. the measures or their timing) (DP 2016, p.6). The *Preferential Strategies* are *adaptive in nature* and *resistant* (robust) to slower or faster CC than assumed in the *Delta Scenarios*; based on new knowledge and monitoring results, the strategies, and / or their measures, can be accelerated or slowed down, and can also be adjusted in terms of scope and design (DP 2014, p.136).[[120]](#footnote-120) Thus, the *Delta Decisions* and *Preferential Strategies* are subjected to a review every six years (DP 2018, p.22).[[121]](#footnote-121) This 6-year review also assesses the need of keeping the envisioned long-term options open or adding new options (DP 2018, p.22).

The MAA system specifies the indicators that must be monitored (to keep track of external changes, of the implementation of measures and their effects, and of new information), and explains how the DP and its contents (its *Preferential Strategies*, *Delta Decisions*, *Delta Plans*, and/or their measures) must be reassessed, and adjusted (if necessary). The MAA serves to regularly evaluate if the work is being done *at the right pace and in the proper direction*, and, if necessary, to adjust the DP’s Strategies (their pace or course) as changes occur or new knowledge arises (DP 2018, p.13; 2017, p.7, 13; 2016, p.6).

The MAA is essential to ensure a truly *Adaptive Planning and Management*.

Under its ADM approach, since 2015, the DP has used the MAA to monitor external developments that may affect the *pace* or the *direction* of the *Preferential Strategies*, and to evaluate (verify) whether the Delta Programme is ‘*on track*’ or whether there is any reason for a change of *course* (e.g. if developments demand a switch of strategy or measure, or a modification in their timing, or by reconsidering targets or measures) (DP 2017, p.8), i.e. to regularly examine whether external developments demand an adjustment of the course (DP 2017, p.17),

From 2015 onwards, the DP has conducted its MAA system, and has gained experience on monitoring and reassessment[[122]](#footnote-122); the DP has clarified what should happen if its underlying assumptions change and how to establish feedbacks between the monitoring system and the learning (based on monitoring results) required for policy adjustment (this aspect is deemed necessary by Restemeyer et al. 2017).

For Jeuken et al. (2014), one of the main / central elements of an Adaptive Planning approach consists of monitoring relevant changes and new information and (in accordance) reassessing the Plan, to be able to adjust/adapt it (by redefining its policies, strategies or measures) (Jeuken et al. 2014, p.1, 3, 23). The monitoring and reassessment system should be accounted for in the Plan itself.

Restemeyer et al. (2017) suggest that one of the essential conditions to make a long-term FRM policy / plan more ‘adaptive’ is ‘*an agile governance process*’ with the ‘*capacity to adjust based on new insights*’ and that ensures learning-oriented governance arrangements (Restemeyer et al. 2017, p.922, 935). More specifically, to ensure the *adaptability* of the plan, a sound monitoring and learning system must be in place, and it must evaluate external developments and existing practices and detect moments when the strategies must be adjusted. It is important to clarify *what to monitor*, *with whom to discuss the results* and *when to take action* (Restemeyer et al. 2017). Regarding the DP, the authors advocated that the monitoring and revaluation system should be institutionalized to increase the adaptability of the Plan, and that this system must regularly review, and, if necessary, update the DP’s assumptions and other policy instruments, which implied ways of incorporating monitoring results and learnings into the planning and management process (preferably before reassessing *Strategies*) (Restemeyer et al. 2017, p.xx).

As a *flexible adaptive approach*, ADM requires continuous monitoring and evaluation (Zevenbergen et al. 2018, p.6). As noted by Zevenbergen et al., in a *flexible adaptive (management) approach*, flexibility depends on the *capacity of the decision-makers to learn from the arrival of new information and their willingness and ability to revise investment decisions based upon that learning* (Zevenbergen et al. 2018, p.1).

In sum, the ongoing monitoring of external and internal developments and changes, of measures’ effects, and of new insights, and also the regular reassessments and reviews of the Plan (policy-programme) and its contents (Delta Decisions, Preferential Strategies, Delta Plans, their measures, or their timing), to allow its adjustment / adaptation over time, can be deemed another key-element of the DP’s ADM approach that has been essential to develop *robust adaptive Strategies* (and a robust adaptive policy-programme) and fundamental to operationalize a real process *Adaptive Planning and Management*.

#### Key-element 5

The ADM approach defines its own process for developing an *adaptive plan/strategy* and operationalizing an *Adaptive Delta Management* (illustrated *in* Brugge and Bruggeman 2019 and *in* Deltares 2018, the latter is adapted from Haasnoot et al. 2013). The process of ADM is based on the process of the DAPP approach (presented by Haasnoot et al. 2013 and by Jeuken et al. 2014): it involves a continual (circular) cycle with 6 main steps (inspired in the DAPP steps) (as illustrated in the diagram of ADM process *in* Deltares 2018, adapted from Haasnoot et al. 2013; and *in* Brugge and Bruggeman 2019).

More precisely, ADM involves a cyclical, iterative process of 6 main steps; each step offers the possibility to adjust elements of the plan / strategy, and, thus, helps to safeguard adaptability in the general plan, and in planning and management process, to cope with change and uncertainty in policymaking. ADM offers a structured management process that considers uncertain future conditions and changes into planning and decision-making (Zevenbergen et al. 2018, p.xxx; Marchand and Ludwig 2014, p.2, 10; Brugge and Bruggeman 2019, p.4).

Importantly, the ADM process differs from the traditional adaptation planning approaches because the starting point of the analysis is the question of ‘*how long will the current strategies be effective under different scenarios*’, which leads to the exploration of alternative measures and the design of multiple solution-pathways (rather than a single solution that is designed for a ‘business-as-usual’ scenario or worst scenario) (Deltares 2018, p.4, 3). Moreover, ADM is different from the traditional Dutch practice of flood risk management because it requires an *adaptive flood risk management* (Klijn et al. 2015, p.848); indeed, with ADM, the DP aims to manage risks and respond to potential problems *in a timely and adaptive manner* (DP 2018, p.14). ADM is also different from the traditional *Adaptive Management* approach.[[123]](#footnote-123) ADM also differs from the ‘classical’ approach where a robust solution is often chosen based on business-as-usual and worst-case scenarios (Brugge and Bruggeman 2019, p.2).

As recognised in the DP, the DP has followed ADM, and ADM relies on a phased decision-making process (DP 2012, p.88). Although the DP follows an annual development cycle (DP 2016, p.6), over the years (from 2010 up 2020), the DP has conducted (carried out) the main steps of ADM process (or similar steps to those prescribed in ADM) (DP 2016, p.6).

The stages of *problem analysis* and *strategy development* took 4 years (from 2010 to 2014) – in this period, the six regional Subprogrammes developed a *Preferential Strategy* for FRM for their region (Haegen and Wieriks 2015; Alphen 2015; Zevenbergen et al. 2018). The development of the *Preferential Strategies* involved a gradual process of exploration and assessment of measures, design of *adaptation paths*, and their assessment and refinement (DP 2014, p.132, 135, 136). In the 1^st^ year (2010-2011), the regional Subprogrammes carried out the ‘*Problem Analysis*’, in the 2^nd^ year (2011-2012), explored and identified their *Possible Strategies*; in the 3^rd^ year (2012-2013), developed them into (elaborated) *Promising Strategies*; and in the 4^th^ year (2013-2014), further redefined and chose their *Preferential Strategy* (Restemeyer et al. 2017, p.928; DP 2014, p.132). After 2014, there was shift towards the detailed design of the *Strategies* and implementation of their measures (Zevenbergen et al. 2018, p.1). Since 2015, the DP annual reports have documented the progress made in the implementation of the *Delta Decisions* and *Preferential Strategies* (of the DP 2014), and if they are *on schedule* and *on the* *right track* (DP 2018, p.19; 2016, p.6). Moreover, since 2015, the DP has evaluated, every year, whether any new developments require the adjustment or fine-tuning of the *Preferential Strategies* and the associated *Delta Plans* (DP 2016, p.6).

Furthermore, ADM, and its ongoing nature, are also reflected in the annual update of the DP (every year a new DP report is issued, including a reprogramming of measures, and is submitted to the Parliament) (DP 2014, p.95; Bloemen et al. 2018). In this process, monitoring and research, and the design and implementation phase, are conducted at the same time and influence each other (DP 2014, p.149)

Overall, the DP’s adaptive approach requires an ongoing process of adaptation planning, which implies monitoring and adjustment, as well as *flexibility* and *adaptive capacity*, namely within the strategies and choices made (Gersonius et al. 2016, p.214; Haegen and Wieriks 2015, p.53).[[124]](#footnote-124)

It can, therefore, be concluded that the ongoing process of ADM – with its iterative cycle of planning, management, monitoring and adaptation, and with its several steps required to develop a *dynamic adaptive plan / strategy* – is another key-element of ADM essential to make the DP an adaptive policy-programme with *robust adaptive strategies*, and to operationalize a real *Adaptive Planning and Management*. Importantly, the first four key-elements previously identified are patent in methods that are used in some of the steps of the ADM process (Marchand and Ludwig 2014, p.3, 10).

#### Synthesis table (Table 4)

| **Table 4. Key-elements of the ADM approach of the DP** | | |
| --- | --- | --- |
| Step of process (Fig. 9) | Interpretation (deriving key-elements through induction) | Key-element essential to design an *adaptive strategy* and operationalize ADM |
| 1b1 | To deal with uncertain future changes and conditions – and *render them manageable* – the DP worked with a range of four plausible futures called *Delta Scenarios* (*Busy*, *Steam*, *Rest, Warm*) (DP 2014, p.135; 2013, p.6, 100; 2012, p.35; 2011, p.14, 46, 48,71; Marchand and Ludwig 2014, p.12; Deltares 2018), used them namely to assess measures and strategies (Jeuken et al. 2014, p.1, 3, 10, 14, 23; DP 2013, p.6).[[125]](#footnote-125) All Subprogrammes used the same four *Delta Scenarios* (DP 2012, p.35; 2011, p.48, 71; 2013, p.6; Marchand and Ludwig 2014, p.12; Werners et al. 2016).  Importantly, the ADM approach itself proposed the use of a spectrum of various *plausible futures* (not predictions), to find out and design *robust flexible* *strategies* (i.e. considering various scenarios when exploring measures and developing strategies) (Marchand and Ludwig 2014, p.12; Gersonius et al. 2016, p.4). The *ADM Implementation Guide* suggested *working with* *scenarios* (Rhee 2012 *in* Restemeyer et al. 2017, p.930, 931). In the DP, the ADM process started with the analysis of current and future problems (Gersonius et al. 2016, p.205); the DP analysed which developments or changes might influence the current and future tasks on FRM (DP 2012, p.88; Marchand and Ludwig 2014, p.16-17); the analysis of *what might happen in the future* required the generation of the four *Delta Scenarios* (Klijn et al. 2016).  The four *Delta Scenarios* differ in terms of rapid/ moderate CC and socioeconomic growth / decline, and present their respective figures for SLR, river discharges, and soil subsidence (DP 2011, p.14, 46, 48, 70; 2012, p.35; 2013, p.100; 2016, p.6; 2014, p.136, 168; Marchand and Ludwig 2014; Deltares 2018; Brugge and Bruggeman 2019; Jeuken et al. 2014, p. 1, 3, 10, 11, 14, 23; Gersonius et al. 2016, p.208; Haegen and Wieriks 2015; Alphen 2015; Restemeyer et al. 2017).  The *Delta Scenarios* are four different *plausible futures* (which show how the climatic and socioeconomic conditions might change until 2050 and 2100), they are not *most probable scenarios* nor predictions (no probabilities were assigned to them) (Jeuken and Reeder 2011, p.7; Brugge and Bruggeman 2019 p.3; DP 2014, p.138; 2011, p.71). The *Delta Scenarios* present *a moderate range of scenarios*, as some developments may fall outside this bandwidth (Jeuken et al. 2014, p.1, 10-11; Brugge and Bruggeman 2019); and translate the most relevant uncertainties about plausible future changes (Marchand and Ludwig 2014, p.15).  The *Delta Scenarios* served to investigate future flood-related problems, risks and needs by 2050 and 2100, namely to see if vulnerabilities or opportunities arise; identify future tasks (in each Subprogramme; determine when the first ATP might occur (and a new measure is needed); inform the development of the *Preferential Strategies* (the Subprogrammes developed their *Strategies* based on the *Delta Scenarios*, these were used to determine when the current actions will become ineffective or insufficient, i.e. the date of first ATP) and to assess measures and *paths* on their performance and robustness under different futures (Jeuken et al. 2014, p.10-11, 13; Alphen 2015, p.312; Brugge and Bruggeman 2019, p.5; Haegen and Wieriks 2015; Haasnoot and Jeuken; Gersonius et al. 2016; Bloemen et al. 2018; DP 2011, p.14, 20; 2013, p.32; 2014, p.136; 2016, p.6; 2010, p.3, 32; Alphen2015; Marchand and Ludwig 2014, p.12).  Thus, using a wide range of climatic and socioeconomic scenarios, namely to assess measures and strategies (*paths*), is an element of the ADM approach essential to develop a *robust adaptive strategy* in each Subprogramme and operationalize an *Adaptive Delta Management* (as also highlighted by Jeuken et al. 2014, p.1, 3, 10, 14, 23). | KE1: Working with, and preparing for, a bandwidth of *plausible future scenarios*, i.e. different climatic and socioeconomic scenarios (rather than a single probabilistic projection of the future), and using them to assess the proposed measures and strategies (Jeuken et al. 2014; Walker et al. 2013; Jeuken and Reeder 2011).  It is necessary to consider various plausible futures to assess what measures can be used to achieve the objectives regardless of how the future unfolds (Walker et al. 2013). |
| 1b2  2b | The ADM approach employs the method of *Adaptation Tipping-Points* (ATPs) (of Kwadjik et al. 2010) (*in* Gersonius et al. 2016, p.204, 213). The *ADM Implementation Guide* (Rhee 2012) recommended working with *Tipping-points* (as noted by Restemeyer et al. 2017, 930).[[126]](#footnote-126) In the ADM process, namely in Step 1, it is necessary to identify external developments to which the objectives are most vulnerable to, by using scenarios, and, in Step 2, it is necessary to analyse *what amount of change can the system handle*, and, in specific, *what is the critical level* (*ATP,* Kwadjik et al. 2010) *before the objectives are not met anymore* (in ADM, ATPs refer to critical levels that represent a threat to the objectives) and *when* this might occur in different future scenarios (Jeuken et al. 2014, p.4).[[127]](#footnote-127)  The DP defines *tipping-points* as points at which the existing system ceases to meet the requirements (DP 2011, p.8); a *tipping-point* occurs when, due to changes in climate or socioeconomic circumstances, the existing measure, policy, or infrastructure, becomes insufficient to comply with the defined criteria (due to physical, technical, or financial constraints or socially unacceptable effects); the analysis of ATPs sets out which and when decisions and new measures must be taken (DP 2011, p.71, 55).  An important aspect in the DP was taking the vulnerability of the current system as the starting point of the analysis; by questioning *what change can the system handle before it runs into trouble,* the Teams could identify ATPs in the system’s vulnerability (Jeuken and Reeder 2011, p.2, 3).[[128]](#footnote-128) The DP and its Subprogrammes sought to identify *adaptation tipping-points* (i.e. points / conditions at which the objectives of FRM policy along the coast and rivers are no longer met, or points under which the existing management strategies, are no longer able to meet the objectives, and alternative strategies are needed), and analyse when these ATPs might be reached (Jeuken and Reeder 2011, p.3, 4).  First, the Subprogrammes sought to identify ATPs in the existing FRM system; to this end, they assessed technical and physical limits of the flood defence system, quantified its current overcapacity, and, based on this and on climate projections, defined new design criteria (Jeuken and Reeder 2011, p.6). The Subprogrammes identified limits (thresholds) of the existing flood defence system, but also limits of the existing management measures and FRM policies (Jeuken and Reeder 2011, p.7). Then, the Subprogrammes used various scenarios to determine the moment of an ATP at earliest and latest, and the moment to adapt (to take a new measure) (Jeuken and Reeder 2011, p.6). Overall, in the DP, ATPs describe conditions under which the current or alternative management measures or policies might fail; such ATPs are associated to acceptable return periods for flood events, which were translated into design criteria for flood defences (*in* Jeuken et al. 2014, p.17).  In the ADM process, Step 1 (*analyse objectives, current and future vulnerabilities and opportunities under different future scenarios*) required assessing what amount of change can the system handle and specifying *Adaptation Tipping-points* (i.e. assessing what are the critical levels at which the objectives are not met anymore) and when these ATPs will occur by using various scenarios (Jeuken et al. 2014, p.4-5), namely by examining *how long the current management strategies continue to be effective under different CC scenarios* (Deltares 2018, p.2). Thus, it was necessary to specify (define) what are *adaptation tipping-points*’, i.e. the ‘boundary conditions’/ points under which the objectives are no longer met (e.g. an unacceptable level of SLR) (Kwadjik et al. 2010), and then, confront such ATPs with a range plausible futures to estimate the moment when such ATPs might occur (Gersonius et al.2016, p.204-206).  In 2009/2010, the DP Team and the Subprogrammes assessed how much longer the current measures (policy and management actions) will suffice and when adjustments will be required, that is, when the first tipping-point for the existing system (at which such measures are no longer tenable) will be reached; and, to answer this, the *Delta Scenarios* were used (DP 2010, p.3-4, 32, 68; Werners et al. 2016).[[129]](#footnote-129) The main issue was analysing if and for how long the current measures will still be satisfactory under a changing climate (more than determining the exact levels of SLR) (DP 2010, p.36).  Furthermore, in the ADM process, Step 2 implied assessing the performance and effectiveness of each measure and determining its tipping-point. Thus, the ATPs were crucial to the design of adaptation pathways; when an ATP is reached, other / additional measures are required to achieve the objectives, the APs map shows which measures are available and when these will be needed (Deltares 2018, p.2-4). In the DP, the design of the *Strategies*, i.e. of their *adaptation paths*, required the identification of ATPs; ATPs played a key role in phasing the possible measures within each *Strategy* (successive measures were sequenced to assemble pathways) (Jeuken et al. 2014, p.17). The reaching of an ATP indicated that a new measure is required. The Subprogrammes sought to identify the ATP for diverse FRM measures, and then analysed when this might occur (i.e. the moment of an ATP for different measures).  In 2011/2012, to start developing their *paths*, some Subprogrammes sought to analyse at what times measures will be required, which implied examining the moment of *tipping-points* (DP 2012, p.90).  Subsequently, in 2012/2013, each Subprogramme sought to design *development paths* or *adaptation paths* as part of its *Promising Strategies*, and this implied analysing the conditions under which it is logical to move from a measure to another (i.e. ATPs), and what and how options can be kept open to allow such transition (DP 2012, p.81, 80).  The ATPs’ method helped to determine at which point in time new management measures will be necessary (Jeuken and Reeder 2011, p.4). [[130]](#footnote-130)  It can, thus, be argued that the identification of *Adaptation Tipping-Points* (ATPs) is another key-element of ADM that was essential to develop a *robust flexible strategy* in each Subprogramme, and to operationalize a process of *Adaptive Delta Management*. | KE2: Identifying *Adaptation Tipping-points*, i.e. conditions under which the current or an alternative measure fails (ceases to be effective / no longer meets the objectives, or the current system performs unacceptably) and a new measure is needed (Walker et al. 2013; Jeuken et al. 2014; Jeuken and Reeder 2011; Zandvoort et al. 2018). |
| Step 3 | The ADM approach aims at developing strategies that are *robust* and *flexible* (Haasnoot 2013, *in* Marchand and Ludwig 2014, p.8, 15). The 2^nd^ and 3^rd^ principles of the DP’s ADM are clearly related to *robustness* and *flexibility* (DP 2013, p.103). The *ADM Implementation Guide* advocates that strategies should be simultaneously *robust* and *flexible*, and it suggests working with *Scenarios*, *Tipping-points*, and *Adaptation* *Pathways*, to find (and design) *robust and flexible strategies* (*in* Restemeyer et al. 2017, p.930, 931).  The DP assumed that each Strategy developed should be both *robust* (with it, the objectives can be achieved in all scenarios of CC and socioeconomic development considered, i.e. under all *Delta Scenarios*) and *flexible* (i.e. the implementation of its strategy(ies), or its measures, can be sped up / slowed down and it should be possible to change to a different measure or to a different strategy (*path*), if necessary (it allows for *switching between strategies*, or *stepping up* measures, or *changing strategies*) (DP 2013, p.103, 94-95; 2014, p.6). The ultimate goal is that the FRM system meets the requirements *at all times* (in all scenarios), which required a strategy *able to adapt to new insights and circumstances* and containing *sufficient options to remain open in the future to take the required measures* (DP 2013, p.102).  Hence, the *Strategy* of each Subprogramme should be *robust* and *flexible* and should be based on the four basic principles of ADM (DP 2013, p.103, 102, 95; 2012, p.88; 2011, p.48; Werners et al. 2016).  To develop their *Preferential Strategy*, the Subprogrammes applied the ADM approach, and ADM itself employs the method of Adaptation Pathways (APs) (*in* Haegen and Wieriks 2015, p.56, 54-55, 48; Restemeyer et al. 2017, p.935, 930; Jeuken and Reeder 2011, p.5; Marchand and Ludwig 2014, p.2, 17; Brugge and Bruggeman 2019, p.2; Zandvoort et al. 2018, p.189; Klijn et al. 2015, p.849; Bloemen et al. 2018, p.5).[[131]](#footnote-131) In its process, ADM uses the APs method (of Haasnoot et al. 2012, 2013): the 3^rd^ step of the ADM process is the development of adaptation pathways and their representation in a map. The APs is a methodological *approach for* *exploring and sequencing a set of possible actions based on alternative external developments over time* (Haasnoot et al. 2012, p.485).[[132]](#footnote-132) The APs approach that was applied in the DP (as part of ADM) was inspired by the APs approach of the TE2100 Project (Jeuken and Reeder 2011, p.5; DP 2011, p.48).  The *Preferential Strategies* were developed based on the four basic principles of ADM – these principles substantiated them (DP 2014, p.47; 2013, p.102; 2012, p.88; 2011, p.48-49; 2016, p.7; 2017, p.7).[[133]](#footnote-133)To support the application of ADM and its principles in the DP, two main methods were used: Adaptation Tipping-Points (ATPs) (Kwadijk et al. 2010) and Adaptation Pathways (APs) (Haasnoot et al. 2013; Haasnoot 2013) (*in* Klijn et al. 2015, p.849; Jeuken and Reeder 2011, p.5; Marchand and Ludwig 2014, p.27). The ADM approach (namely its 3^rd^ principle) implied the development of adaptation pathways. The design of pathways is one of the main steps of the ADM process.  The 3^rd^ principle of ADM (*working with multiple strategies that can be alternated between*, *i.e.* *with* *adaptation paths*) (DP 2012, p.88, 81; 2013, p.102; 2011, p.48; Rhee 2012 *in* Werners et al. 2016, p.118) implied developing multiple strategies that can be used alternatingly (in an alternative pattern) (Marchand and Ludwig 2014, p.8; Gersonius et al. 2016, p.202), that is, designing several adaptation pathways, called ‘*adaptation paths*’ in the DP, between which it is possible to alternate (switch) depending on developments. This required the design of adaptation pathways (as sets of sequenced measures) that together provided a general adaptive strategy (Haegen and Wieriks 2015). This principle involved using the method of APs to design the strategies (Brugge and Bruggeman 2019).  For the Subprogrammes, the 3^rd^ principle involved *working with several potential strategies*, i.e. with *adaptation paths*, and appraising the *flexibility* around such *paths* (DP 2011, p.48). The 3^rd^ principle of ADM has translated into the design of *adaptation paths* in each *Preferential Strategy* (DP 2012, p.81; 2011, p.48; 2014, p.47). In line with the 3^rd^ principle, each regional Subprogramme developed a map of *adaptation paths* in its *Strategy* (each *Strategy* contains a map with one or more *adaptation paths*) (DP 2014, p.47; 2013, p.56; 2012, p.81; 2011, p.48).  Each *Preferential Strategy* presents a map of *adaptation path(s)*; the term ‘*adaptation path*’ refers to a ‘pathway’; an *adaptation path* is a logical set of measures that includes measures for the short-term and options for the mid- and long-term (DP 2013, p.56). Thus, a *Strategy* delivers *sets of measures*; each *set of measures* offers a logical *path*, and *sets of measures* (i.e. several *paths*) were elaborated for each Region (DP 2013, p.56, 95; 2012, p.81).[[134]](#footnote-134)  More broadly, each *Preferential Strategy* contains objectives, measures to achieve the objectives, and the associated *adaptation path(s)* (i.e. a set, or sets, of measures) displayed in a map (DP 2013, p.56; Bloemen et al. 2018; Alphen 2015; Rhee 2012, p.18 *in* Zandvoort et al. 2018, p.190). Each regional Subprogramme drew up a *Preferential Strategy* for FRM for its region (DP 2014, p.8, 46), and each *Preferential Strategy* has a map with one or more *adaptation paths* (DP 2013, p.56; 2012, p.81; 2011, p.48).  Each *path* (pathway) is a sequence of actions over time to achieve the predefined objectives (Marchand and Ludwig 2014, p.2, 13, based on Haasnoot 2013); the various paths provide *coherent sequences of measures and potential options,* which may be activated before an ATP occurs (Zevenbergen et al. 2018, p.xx, based on Haasnoot et al. 2012, 2013; Gersonius et al. 2016, p.204).[[135]](#footnote-135)  In each Subprogramme, *various adaptation paths* (initially called *development paths*) were studied and designed, which required studying the circumstances under which it is logical to move from one *path* to another, and which and how options should be kept open to allow such transitions (DP 2012, p.81), i.e. laying down the conditions under which changing or switching of measure or strategy (*path*) is reasonable and possibilities for shifting between strategies (DP 2013, p.95, 77; 2011, p.48; Gersonius et al.2016, p.205).[[136]](#footnote-136)  In the DP 2014, each *Preferential Strategy* presents a map of *adaptation paths*, with one or more *adaptation paths*; the map shows the pathway(s) to be followed; it indicates which measures can be taken (including measures required now to keep options open for the long-term) and when they are expected to be necessary (DP 2014, p.47)[[137]](#footnote-137); in specific, the map displays the possibilities (options) for switching of measure or *path* (strategy), and it also identifies short-term measures required to be able to change strategies later, if necessary (DP 2013, p.95, 77). Each *Preferential Strategy*, in its *adaptation path(s)*, includes measures for the short-term (2030), and possible options for the mid- (2050) and long-term (2100), including possible adjustments/amendments that might be necessary in strategies (DP 2014, p.49, 148) which implied looking at the conditions under which it is wise to shift from a strategy, or from a measure, to another one (DP 2011, p.48; Gersonius et al.2016, p.205).  In line with the 3^rd^ principle of ADM, each Subprogramme developed *multiple strategies that can be alternated between*. i.e. several *adaptation paths*, and *appreciated* (appraised) the *flexibility* of such strategies (DP 2013, p.102; 2011, p.48). In each *Preferential Strategy* (in its *adaptation path(s)*), measures may be taken sooner or later (DP 2014, p.47), and it may be necessary to switch of measure or strategy (*path*) (DP 2013, p.77, 95, 103; 2014, p.6, 169), depending on climatic and socioeconomic developments. In the DP, the APs approach allows for switching between measures (withing each pathway) or between pathways (i.e. shifting from a measure, or pathway, to another one), if necessary, in view of CC or socioeconomic developments, and ensures that options are kept open for the long-term (Haegen and Wieriks 2015, p.56, 50, 54; Bloemen et al. 2018, p.12, 14).  The basic idea underlying the APs approach (in ADM) is *to generate a wide array of pathways* (each pathway *consisting of a series of measures*) *through which the policy-objectives are achieved under changing climate and socioeconomic conditions* (Deltares 2018, p.4, 3). The set of possible pathways mapped out in a map provides a range of adaptation options that may be used in future planning, in this way, the APs approach helps to enhance the plan’s and the system’s *flexibility* and *adaptability* (capacity to adapt to multiple plausible future changes and conditions) (Deltares 2018, p. 4, 2).  ADM utilizes the APs method as a *flexible, adaptive approach* for designing strategies under plausible future scenarios, which allows for switching between measures and strategies along the adaptation pathways if necessary, in view of socioeconomic developments or CC (Haegen and Wieriks 2015, p.56, 50, 54). A key step in the ADM process is *to develop a set of measures along adaptive pathways* (Haegen and Wieriks 2015, p.55).  In sum, in the DP, *strategy-making* was based on the ADM approach, and ADM builds on an important element to develop strategies: the *construction of pathways*, which, in turn, implied assessing the flexibility of measures and pathways, and scanning options to keep open – both aspects are patent the *Strategies* for FRM of the DP (Zandvoort et al. 2018, p.190, 189, 191). The main outcome of ADM was a single pathway, or a set of pathways, with which it was possible to schedule measures in the face of uncertain future change – that is, ADM ‘*leads to a composite strategy, or a set of alternative strategies with intermediate possibilities for revisions*’ (Rhee 2012, p.14, *in* Zandvoort et al. 2018, p.191).  The *Preferential Strategies* were developed on the basis of the four *Delta Scenarios*, but they are, by nature, *adaptive*: they can be adapted *to* *slower and faster CC* than expected in the *Delta Scenarios*; for example, *by accelerating or slowing down the implementation of measures*, that is, the strategies, and / or measures within them, may be advanced or postponed in time, and also adjusted in terms of scope and design (DP 2014, p.136). As the climatological and socioeconomic circumstances may turn out differently than expected in the *Delta Scenarios*, and the *Delta Scenarios* may be periodically updated over time, the *Preferential Strategies* were designed to be *adaptive* and *resistant to slower and faster CC* than the expected (DP 2014, p.168, 136; 2016, p.6).  The APs method contributed to safeguard / ensure *robustness* and *flexibility* in the *Strategies.* With the APs approach, the Subprogrammes could envision alternative measures and possibilities for switching between them through adaptation pathways, and devise measures for the short-term linked (coupled) with long-term possible alternatives (options) (Jeuken et al. 2014, p.1). The APs approach served to explore possible measures and options, and possibilities for switching from one option to another in the future, and, in this way, remain flexible – thus, it enhanced the *flexibility* of each general *Strategy*. By using the APs, it was possible develop, a ‘*robust and flexible*’ set of measures – or, precisely, *sets* – each *set* as an adaptation pathway. The map of *adaptation paths* shows the measures available, including options for switching in the future, and when it is necessary to switch of measure (Jeuken et al. 2014, p.10).  The APs method served to develop a *robust and flexible strategy* for FRM; the development of pathways delivered *flexibility* in several ways, namely: the strategies (paths) can be accelerated / slowed down, changed or adjusted, e.g. by shifting from one measure to another available, by using other options that are kept open (Gersonius et al. 2016 p.212).  It can, therefore, be concluded that developing a *robust* and *flexible* set of measures, to deal with uncertain future changes, (with) through the method of *Adaptation Pathways* (APs) is other key-element of ADM that has been essential to draw up (make) each *Strategy* as a *robust adaptive strategy*, and to operationalize a real *Adaptive Delta Planning and Management.* More precisely, the development (design) of *robust flexible strategies* – each strategy as a *robust flexible* *set* of measures, i.e. a pathway (or a set of pathways) – by using the APs approach, can be deemed another element of the DP’s ADM approach that is key/fundamental to create, in each Subprogramme, a general (*Preferential*) *Strategy* that is *robust* and *adaptive*, and to fulfil the DP as a *robust adaptive policy-programme* (in line with the elements proposed by Jeuken et al. 2014, p.23, 1, 3, and by Restemeyer et al. 2017, p.921, 922-925, 935, and as suggested in the study of Jeuken and Reeder 2011, p.2,5). | KE3: Developing a ‘*robust and flexible’* set of measures (i.e. *robust flexible strategies*), to cope with uncertain future conditions and changes, using the *Adaptation Pathways approach* (APs) (Jeuken et al. 2014).  This element consisted of developing *robust flexible strategies* (each strategy containing a *robust* and *flexible set of measures*), which was done using the APs approach (Jeuken and Reeder 2011, p.2; Restemeyer et al. 2017, p.921-922, 927, 935). It involved designing each *Strategy* as a ‘*robust and flexible*’ set of measures (with robust and/or flexible measures), and required considering the *robustness* and *flexibility* of *Strategies.* |
| Step 4b  +  Step 6 | ADM implies an ongoing monitoring of external conditions (climatic and socioeconomic developments), of implemented measures (i.e. *what we are doing and what results this yields*), and of new knowledge, and, based thereon, the regular (re)evaluation of strategies followed (implemented and planned) to check if they are proceeding as expected and if it is necessary to adjust them – their *pace*, *direction* or *content* – as new insights into climatic and socioeconomic changes arise (it might be necessary to adjust strategies over time) (DP 2016, p.6; 2014, p.149; Zevenbergen et al. 2018, p.6; Petersen and Bloemen 2015, p.225, 221-223; Zandvoort et al. 2018, p.193, 190, 192). Monitoring is also needed to detect when ATPs might be reached (Restemeyer et al. 2017, p.931).  For the DP, ADM requires *persistently checking whether we are working at the right pace and in the proper direction*, and, *if need be, adjusting our strategies in time* (DP 2018, p.13; 2017, p.7, 13; 2016, p.6), i.e. *adjusting strategies periodically* based on new knowledge which demands *a clear view of the possibilities for the future at all times* and ongoing monitoring and evaluation – *monitoring and evaluation are essential to know in good time when the strategy has to change and when other measures that have already been prepared should be put into effect* (DP 2014, p.13).  In specific, in the DP, ADM required monitoring changes and developments, new knowledge, and effects of measures, and regularly reassessing/reviewing the Plan – its Strategies (applied and planned), their measures, or their schedule / timing – and adapting / adjusting them if changes or new insights prompt so; thus, ADM presupposes the existence of a monitoring and evaluation system and a *learning process*, and also reviewing choices, strategies and plans, and adapting them if necessary (DP 2016, p.59, 11, 61, 6).[[138]](#footnote-138)  Since 2015, the DP defined and established its own monitoring, evaluation and reassessment system, called *Monitoring, Analysing, Acting system* (MAA), which is considered the *engine of ADM* (DP 2016, p.6, 59; 2014, p.149). It started to operate in 2017 (DP 2016, p.3, 63). The MAA serves mainly to:   - monitor changes and developments, measures’ effects, new insights and knowledge, and, in accordance, regularly revaluate (review) the strategies and measures (implemented and planned) (DP 2016, p.6, 9, 11, 59); - examine whether and how (and when) the *Preferential Strategies* need to be adjusted (the MAA generates insights into changes and progresses that may constitute reasons to adjust the *pace* or *direction* of Strategies, and it enables their timely adjustment, e.g. by changing of course or measure, hence, it is indispensable to operate the DP’s adaptive approach) (DP 2016, p.6-7, 9, 11, 59; 2017, p.13; 2016). More precisely, the DP uses the MAA to: evaluate (review), every year, whether any new developments demand the adjustment or fine-tuning of the *Preferential Strategies* or *Delta Plans*; and check whether any new conditions, developments or changes impact the goals and measures defined, and if necessary, adjust them (DP 2016, p.6, 7). The results of the MAA system must be used to update the DP on observed and forecasted effects and must be considered in any revision of the programmed measures (namely *implementation programmes*) (DP 2014, p.149-150). The MAA underpins the DP’s ADM approach; ADM is systematically substantiated in the MAA (DP 2016, p.9, 6).   In 2017/2018, the *Signal Group* defined 8 *indicators* to be monitored in order to detect signals that may prompt an adjustment of the *Preferential Strategies*, developments that could jeopardise the attainment of goals, among them, SRL, extreme river discharges, land use, etc. (DP 2018, p.111; 20).  Besides this, the DP must carry a review every 6 years to check (based on monitoring results) if it is necessary to maintain or adjust the *course* or the *pace* of Strategies (e.g. measures or their timing) (DP 2016, p.6) – i.e. every six years, the DP carries a systematic review namely to check ‘*whether it managed to keep up the pace and adjust the course on time*’ and analyse if monitoring results indicate that it is necessary to maintain or adjust the *course* or *pace* of Strategies (i.e. their measures or their timing) (DP 2016, p.6). Thus, the *Delta Decisions* and *Preferential Strategies* are subjected to a review every six years, and such 6-year review also assesses the need of keeping the envisioned long-term options open or adding new options (DP 2018, p.22). Moreover, the *Preferential Strategies* are *open to annual adjustment* (DP 2018, p.22).  The *Preferential Strategies* are *adaptive in nature* and *resistant* (robust) to slower or faster CC than assumed in the *Delta Scenarios*; based on new knowledge and monitoring results, the strategies, and / or their measures, can be accelerated or slowed down, and can also be adjusted in terms of scope and design (DP 2014, p.136).  The MAA system specifies the indicators that must be monitored (to keep track of external changes, implemented measures and their effects, and new information), and explains how the DP and its contents (its *Preferential Strategies*, *Delta Decisions*, *Delta Plans*, and/or their measures) must be reassessed, and adjusted, if necessary. The MAA serves to regularly evaluate if the work is being done *at the right pace and in the proper direction*, and, if necessary, to adjust the DP’s Strategies (their pace or course) as changes occur or new knowledge arises (DP 2018, p.13; 2017, p.7, 13; 2016, p.6).  The MAA is essential to ensure an *Adaptive Planning and Management*. Since 2015, the DP has used the MAA to monitor external developments that may affect the *pace* or the *direction* of the *Preferential Strategies*, and to evaluate whether the DP is ‘*on track*’ or whether there is any reason for a change or adjustment of the *course* (e.g. if developments demand a switch of strategy or measure, or a modification in their timing, or reconsidering targets or measures) (DP 2017, p.8,17).  In sum, the ongoing monitoring of external and internal developments and changes, of implemented measures and their effects, and of new insights, and also the regular reassessments/ reviews of the Plan (policy-programme) and its contents (Delta Decisions, Preferential Strategies, Delta Plans, their measures, or their timing), to allow their adjustment / adaptation over time, can be deemed another key-element of the DP’s ADM approach that has been essential to develop *robust adaptive Strategies* (and a robust adaptive policy-programme) and fundamental to operationalize a real process *Adaptive Delta Management* (in accordance with the elements proposed by Jeuken et al. 2014, Restemeyer et al. 2017, and with the findings of Zevenbergen et al. 2018, p.6, 1). | KE4: Monitoring changes, followed *Strategies*, and new information, and periodically reassessing *Strategies* and *Delta Plans*, and, if necessary, adjust them, their measures or their timing (Jeuken et al. 2014; Jeuken and Reeder 2011, p.7). |
| Whole process | The ADM approach sets out its own process for developing an *adaptive plan/strategy* and operationalizing an *Adaptive Delta Management* (as illustrated *in* Brugge and Bruggeman 2019 and Deltares 2018), which is based on the process of the DAPP approach (presented by Haasnoot et al. 2013 and Jeuken et al. 2014). It involves a continual (circular) cycle – i.e. a cyclical, iterative process – with 6 main steps (inspired in the DAPP steps), and each step offers the possibility to adjust elements of the general plan / strategy, thus, it helps to safeguard the adaptability both in the general plan and in the planning and management process, to cope with change and uncertainty. ADM offers a structured management process that accounts for uncertain future changes in planning and decision-making (Zevenbergen et al. 2018; Marchand and Ludwig 2014, p.2, 10; Brugge and Bruggeman 2019, p.4).[[139]](#footnote-139)  The DP has followed ADM, and ADM relies on a phased decision-making process (DP 2012, p.88). Although the DP follows an annual development cycle, over the years (namely from 2010 up to 2020), the DP has carried out the main steps of ADM process (or similar steps to those prescribed in ADM) (DP 2016, p.6).  The stages of *problem analysis* and *strategy development* took 4 years (from 2010 to 2014) – in this period, the regional Subprogrammes developed a *Preferential Strategy* for FRM for their region (Haegen and Wieriks 2015; Alphen 2015; Zevenbergen et al. 2018). The development of the *Preferential Strategies* involved a gradual process of exploration and assessment of measures, design of *adaptation paths*, and their assessment and refinement (DP 2014, p.132, 135, 136). In the 1^st^ year (2010-2011), the regional Subprogrammes carried out the ‘*Problem Analysis*’; in the 2^nd^ year (2011-2012), they explored and identified their *Possible Strategies*; in the 3^rd^ year (2012-2013), they developed their *Promising Strategies*; and in the 4^th^ year (2013-2014), further redefined and chose their *Preferential Strategy* (Restemeyer et al. 2017, p.928; DP 2014, p.132). After 2014, there was shift towards the detailed design of the *Strategies* and implementation of their measures (Zevenbergen et al. 2018, p.1). Since 2015, the DP annual reports have documented the progress made in the implementation of the *Delta Decisions* and *Preferential Strategies* (of the DP 2014), and if they are *on schedule* and *on the* *right track* (DP 2018, p.19; 2016, p.6). Moreover, since 2015, the DP has evaluated, every year, whether any new developments require the adjustment or fine-tuning of the *Preferential Strategies* (DP 2016, p.6). ADM, and its ongoing nature, are also reflected in the annual update of the DP: every year a new DP report is issued, including a reprogramming of measures and is submitted to the Parliament (DP 2014, p.95; Bloemen et al. 2018). In this process, monitoring and research, and the design and implementation phase, are conducted at the same time and influence each other (DP 2014, p.149)  The DP’s *adaptive approach* required an ongoing process of adaptation planning, which implied monitoring and adjustment, but also *flexibility* and *adaptive capacity* within the strategies and choices made (Gersonius et al. 2016, p.214; Haegen and Wieriks 2015, p.53).[[140]](#footnote-140)  It can, therefore, be concluded that the ongoing process of ADM – with its iterative cycle of planning, management, monitoring and adaptation, and with its several steps required to develop a *dynamic adaptive plan / strategy* – is another key-element of ADM essential to make the DP an adaptive policy-programme with *robust adaptive strategies*, and to operationalize a real *Adaptive Planning and Management*. Importantly, the first four key-elements previously identified are patent in methods that are used in some of the steps of the ADM process (Marchand and Ludwig 2014, p.3, 10).  The DP has adopted ADM and its inherent continual long-term process (its cycle of several steps illustrated in the scheme of the ADM process). This process involves planning, managing risks, and adapting. This continuous iterative process is necessary to develop and implement *adaptive strategies*, and undertake a true *Adaptive Delta Planning and Management*. | KE5: ongoing, iterative, long-term process of ADM, including its various steps required to fulfil a real *Adaptive Delta Planning and Management*. |

These five key-elements must be ensured to truly allow and operationalize an Adaptive Planning and Management, and they are embedded in the steps of the ADM process (Jeuken et al. 2014). The DP, and its Subprogrammes, contain these elements.

In sum, the main elements / ‘building-blocks’ of the ADM approach are: to work with several plausible future scenarios; the develop and use a ‘*robust and flexible*’ set of measures (i.e. *robust flexible strategies*) which requires the identification of tipping-points and the design of adaptation pathways; the monitoring of relevant changes and the revaluation of Strategies and their adaptation, if necessary; and the ongoing process of steps required to achieve operate an Adaptive Planning and Management.[[141]](#footnote-141)

# NOTE 5. Comparative analysis of the two cases on each key-element identified

#### Regarding Key-element 1

While the TE2100 has worked with four SLR scenarios and four socioeconomic scenarios (separately), the DP has created and worked with a matrix /crux of 4 future plausible scenarios that combined different figures for climate change effects and socioeconomic development. The TE2100 has used a wider range of SLR scenarios (0,9m - 2,7m / 4,2m SLR until 2100), and initially an extreme / high-end scenario (4,2m SLR), to assess measures and pathways (on their effectiveness and overall plan’s robustness), whereas the DP2014 used a more moderate range of scenarios (0,35 - 0,85m SLR until 2100) which did not include high-end (high-impact, low probability) scenarios.

Importantly, while the RE-D Subprogramme worked with the four Delta Scenarios (translated to the regional context), the effectiveness of its strategies was only assessed in the *Steam* and *Rest*. The question of ‘which scenario should be used’ did not matter too much, because whatever Delta Scenario happens, the existing flood defence system (its dykes and storm surge barriers) can cope with it, and it will require some improvements but not radical modifications (Restemeyer et al. 2017, p.931). In the RE-D Subprogramme, experience showed that working with scenarios was easier in theory than in practice: it was too difficult for people to think about four possible futures, they tended to focus on the ‘steam’ and ‘rest’ scenarios (Restemeyer et al. 2017, p.931). Overall, in the RE-D Subprogramme, experience showed that working with scenarios was easier in theory than in practice: it was too difficult for people to think about four possible futures, they tended to focus on the ‘Steam’ and ‘Rest’ scenarios (Restemeyer et al. 2017, p.931). The idea that “whatever scenario happens, the existing defence system can cope with it and it will not require radical modifications” hampered the identification of tipping-points and visualization of pathways (Restemeyer et al. 2017, p.931).

#### Regarding Key-element 2

Both cases sought to identify critical thresholds (TE2100) or Adaptation Tipping-points (ATPs) (DP2014) – referring to points / conditions under which the current or alternative (policy and management) measures no longer meet the predefined objectives / fail (Jeuken et al. 2014).

In the TE2100 case, the identified thresholds were crucial in the sequencing of possible measures within the broader plan, and, with such sequenced measures to assemble adaptation pathways that connect decisions and actions in the short-term to options in the long-term (Jeuken et al. 2014).

The design of the maps of adaptation paths with the X-axis representing time (rather than threshold-values), also reflects a difficulty that has been faced by most DP Subprogrammes: the specification of Adaptation Tipping-points was not easy nor straightforward, and ultimately, contributed the lack of clarity regarding what ATPs were identified. In contrast, in the TE2100, not only was the specification of thresholds explored, but it was also devised a way of calculating decision-points (similar to the transfer-stations of a standard APs map) and implementation-points (at which an action must be in place and functioning).

In the DP case, several Subprogrammes faced difficulties in specifying ATPs., indeed the identification of ATPs was deemed one of the most difficult and complex aspects of ADM by the DP staff, particularly by the RE-D Subprogramme (Gersonius et al. 2016). ATPs were difficult to identify in situations where: a) the signals of climate change are weak in relation to the natural variability of the system, as in river discharges; b) in the case of strategies that are inherently flexible, e.g. sand nourishment (which can be extended indefinitely), c) there is an absence of clear policy goals or binding safety norms, which happens in unembanked areas (Gersonius et al. 2016; Zevenbergen et al. 2018; Bloemen et al. 2018).[[142]](#footnote-142)

In the APs, it is implicitly assumed that some physical parameters that influence flood risk (e.g. climatic conditions that influence the probability of floods, or socioeconomic developments that influence flood consequences) change gradually and allow societal systems to react and shift to a new measure. This works well in situations characterized by gradual trends/ slow changes, e.g. SLR, however, evidence shows that, in situations of large natural variability, the determination of tipping-points is challenging; for instance, there is usually a great difficulty in monitoring changes in the patterns of storms, in conjunction with a lack of observations of extreme events (Bloemen et al. 2018, p.14). The detection of CC-induced changes in certain parameters, like river discharges, requires detailed monitoring data and model calculations and often shows that the natural variability is so high that it may take several decades until CC signals can be distinguished accurately. Further research is needed to discern CC signals from ‘common’ measurements, through approaches that use observations and future projections, scenarios and modelling; there is a need of methods to distinguish climate signals from highly variable measurements of physical conditions (Bloemen et al. 2018, p.14). The ATPs method works for slowly changing conditions, but for drivers affected by extreme events/ sudden changes, it runs the risk of being too late (Gersonius et al. 2016; Zevenbergen et al. 2018; Haasnoot et al. 2013). Besides, it also difficult to determine ATPs where there are not precise goals, and when strategies are inherently flexible, e.g. the DP’s strategy to manage coastal flood risk is sand nourishment, which is flexible: the sand volume supplied per year can be increased/ decreased according to observed SLR rate (Bloemen et al. 2018, p.14).

The ATP and APs methods can be quite complex and abstract for strategic policymaking at a national or regional scale (Restemeyer et al. 2017). In the DP, ADM was time-consuming and required detailed knowledge of the FRM system (Gersonius et al. 2016). For example, the RE-D Subprogramme faced several difficulties in working with ATPs and APs; in practice, it was quite complex to identify tipping-points and envision adaptation pathways (Restemeyer et al. 2017). Both ATPs and APs methods assume that tipping-points can be identified beforehand (or, at least, when they are reached), however, practice shows that there are limits to the forecasting capacity and it is quite difficult to define adequate monitoring parameters, namely in cases of large natural variability (Restemeyer et al. 2017). While some indicators / variables can be easily monitored (e.g. SLR), others present more challenges, e.g. river discharge which shows a large natural variability (it can increase or decrease during a period without a pattern). In the DP, it was nearly impossible to distinguish climate change signals from natural variations in river discharge. Hence, the DP decided to assume a fixed value of river discharge (maximum value): 17 000 m^3^/s in 2050 and 18000 m^3^/s in 2100. However, as noted by the DP Staff member responsible for ADM, taking decisions and measures based on ‘artificially-fixed’ worst future conditions (in cases where one cannot rely on monitoring) goes against the idea of ‘flexibility’. Setting a fixed value resembles more a ‘predict-and-control’ approach than an adaptive approach (Restemeyer et al. 2017, p.931). In the RE-D Subprogramme, ATPs were difficult to find or did not exist, thus, the exercise was more about “*spreading measures in time*” than “*exploring alternative measures to shift to*” (Restemeyer et al. 2017, p.932). APs method can be complex and abstract for policymaking at national and regional scales; it is more feasible at a local scale, where “*specific measures can be discussed in depth and better embedded into the physical, social and political reality*” (Restemeyer etal.2017, p.936).

All in all, both the TE2100 case and the DP2014 case have identified thresholds/ ATPs, namely thresholds/ATPs that are related with acceptable flood return periods, which were translated into design criteria for adaptation measures (e.g. flood protection structures) (Jeuken et al. 2014). In the case of the TE2100, FRM policies were set for different “policy units” in the Estuary (based on an assessment of the pre-existing Standards of Protection (SoPs, which correspond to a maximum acceptable flood probability expressed as a flood return period) and whether these SoPs should be increased, maintained or reduced in each policy-unit (Ramsbottom and Sheppard 2017, p.9; 4)[[143]](#footnote-143); whereas in the DP2014, the flood safety standards (expressed a flood probability per year and set out per each defence / dyke section) were updated, and these new standards must be met until 2050 (these new flood protection standards were set in the DD on FRM, and were defined based on the worst scenario of the four Delta Scenarios – i.e. the Steam) (DP2014, p.158; Bloemen et al. 2018; Klijn et al. 2016). In the TE2100 case, the new design SoPs set out a threshold-value of acceptable flood probability for the flood defence system that must not be exceeded (Ramsbottom and Sheppard 2017, p.4), and, similarly, in the DP2014, the new flood protection standards establish new standard specification for the primary flood defences.

#### Regarding Key-element 3

While the TE2100’s route-map clearly displays various pathways available to cope with changing conditions over time, and keep flood risk within an acceptable limit (threshold-values), the maps of adaptation paths of the DP’s Delta Decisions and Preferential Strategies may display a single path or various paths available over time, yet, most often, it is shown a single path with few options (possibilities) for switching to another measures – a single composite strategy or a set of strategies but with less “branching-points” (transfer-points) and less options available after such branching-points, resulting in an apparently more “deterministic” strategy (as noted by Restemeyer et al. 2017). The representation of a single path, yet, is in line with the selection of “one or more preferred pathways as input for a dynamic adaptive plan” suggested in the fourth step of the ADM cycle (Deltares; Brugge and Bruggeman; Haasnoot and Jeuken; Jeuken et al. 2014, p.4-5).

Moreover, the TE2100 case clearly demonstrates its APs map is an evolving map (which has been updated, reviewed and redone over the last decade), whereas the maps of adaptation paths of the DP were presented in the DP2014, and since then no updates were presented in the annual DP reports.

This indicates that it may be useful to have two APs maps that should be regularly updated: a map with several possible pathways available to cope changing conditions (e.g. SLR) which is thresholds-/ATPs- dependent (i.e. with threshold-values indicated in the x-axis), and an additional map with one (or more) preferred pathways which is time-/years-dependent (i.e. with relevant time-horizons represented in the x-axis, according to a specific scenario (e.g. deemed most likely).

In TE2100 case, the developed route-map contains multiple (five) possible alternative pathways (HLOs/ Options), which allow for switching between different measures or between different pathways (Options). In the TE2100 case, if new information (e.g. updated scenarios) becomes available, it is possible to switch to other measures or pathways (Options), or adjust the Plan itself.

In the DP 2014, each of DD and PS developed contains one or more “adaptation paths”, which allow shifting between measures (and, in the cases where more than one path is available, from a path to another one). For example, the DD of the Rhine-Meuse Delta (**Fig. 5**) provides several alternative paths, and the DD on Sand (**Fig.6**) illustrates two main alternative “paths” after 2040. The PS for the Rhine Estuary-Drechtsteden (**Fig. 4**) presents a sequence of simultaneous (parallel, combinations of) measures that must be taken in three time-periods (short-, mid-, and long-term), with the possibility to choose between two different measures, or using both, after 2050, thereby offering three distinct possible options for the long-term. Overall, in the DP2014 case, the maps of adaptation paths (of each DD and PS) safeguard the flexibility (of the general plan) by envisioning of a stepwise, staged implementation of several measures over time, and ensuring the possibility of switching to other possible measures (Jeuken et al. 2014, p.18).

The PS for the RE-D, several ‘parallel trajectories’ (horizontal lines) were drawn, such horizontal lines do not constitute alternative pathways, but instead correspond to diverse measures to be implemented over each time-period, i.e. simultaneously to other horizontal lines (e.g. at different locations). These horizontal, parallel “lines of action” interact with each other (e.g. they are complementary, concurrent), and are scheduled over the time-periods. Thus, a map can contain several ‘parallel” horizontal lines. Each horizontal line consists of one measure that is adjusted over time.

In the TE2100 case, these “parallel, simultaneous measures” were combined/ grouped into “portfolios of measures” (each box of the route-map represents a “portfolio of measures”).

In theory and conceptually, adaptation pathways are various alternative routes available that show *“possibilities for switching from one trajectory to another when conditions indicate it is wise to do so*”, and thus, only one trajectory is followed according to the existing and expected conditions, however, in some PS, the map of the ‘adaptation path’ contains several parallel horizontal lines that consist of simultaneous (concurrent) measures (Bloemen et al. 2018, p.14). Though these horizontal lines, at first glance, appear to be different pathways, most often, they correspond to diverse measures that will all be followed simultaneously and that are interrelated (e.g. optimizing each other). Thus, in each Preferential Strategy, there can be different measures envisioned for different zones at the same time-period, e.g.: dyke reinforcement projects, improvements of a storm surge barrier, and pilot projects on multi-layer FRM. The use of parallel measures / lines of action is deemed advantageous: a plan/ strategy with parallel lines of action enhances the system’s resilience, since it has more fallback options if one measure does not perform as expected. However, these parallel measures often concern different actors in diverse zones or fields, thus, the interrelatedness of their outcomes tends to be disregarded, and their successful (effective) implementation becomes more uncertain (Bloemen et al. 2018).

In the PS of the RE-D (**Figure 4**), the only option (alternative measure) indicated is ‘adjusting the river discharge distribution’. This option, which requires a prior analysis of how much the dykes need to be strengthened, was deemed *adaptive* since it is possible to do more or less of it depending on the climatic and socioeconomic changes, however, it is not clear how to evaluate whether more or less of this measure should be done (Restemeyer et al. 2017, p.932).[[144]](#footnote-144) For Restemeyer et al., the general Strategy looks quite “determined and not so flexible”: it provides “little room for adjustments along the way”, and it focusses mostly on ‘prevention’ through the ‘gradual adjustment’ of the existing defence system (“maintaining and improving the existing system”) (Restemeyer et al. 2017, p.932, 934). Although the Team recognized the need for spatial adaptation measures in addition to the traditional preventive measures, in this Strategy, “adaptability mainly gets down to gradual adjustments of certain measures”, e.g. dyke strengthening (Restemeyer et al.2017, p.932, 934-935). As noted by these authors, this Strategy is “predominantly preventive” (strongly based on measures to reduce the probability of flooding, e.g. dyke improvements, optimization and replacement of storm surge barriers), and the measure “river widening” is only envisioned for Dordrecht; thus, further attention should have been paid to the diversity of measures, including measures to reduce the probability of flooding and measures to reduce flood consequences. (Restemeyer et al.2017, p.933-935).[[145]](#footnote-145) Power and money issues also made the strategy-making process less rational than the APs assumes; in cases where there are two options but there is willingness to invest now, the political reality tends to be determining (Restemeyer et al.2017).

In the RE-D Subprogramme, the development of Strategies followed a quite linear filtering (screening) process, from “Possible” to “Promising” strategies, and then to a “Preferential Strategy” (Restemeyer et al.2017, p.929). During this process, some measures discussed in the phase of Possible Strategies, e.g. a ring of weirs or a closed dam on the seaside, were excluded, because they raised controversy (engineer groups and the Agricultural and Horticultural Organization were favorable, while some nature organizations were in favor of allowing more natural estuarine dynamics in the seaside). Thus, the Subprogramme opted to “maintain and improve the existing flood defence system” as a middle-course solution. The Subprogramme Team considers that this Preferential Strategy is *robust* because it can cope with the most extreme Delta Scenario used (the *Steam*) and *flexible* because it does not imply large-scale interventions but only gradual adjustments to the existing system. However, for Restemeyer et al., this Strategy may not necessarily lead to an improved adaptability and resilience, because the measures of gradual adjustment of the existing system (like dyke improvements, heightening or strengthening, or the optimization and eventual replacement of storm surge barrier) aim at reducing the probability of flooding; and measures to reduce flood consequences (e.g. flood-proofing buildings and evacuation measures) were only considered for a few unembanked areas (e.g. Dordrecht). The measure “river widening” is only set out for one part of the region (east of Dordrecht).[[146]](#footnote-146) Thus, further attention could have been paid to the use of diversified set of measures, including both measures to reduce the probability of flooding and measures to reduce flood impacts / consequences (Restemeyer et al. 2017). This Strategy calls for the integration of FRM and spatial planning (more than other subprogrammes), but it focusses mostly on improving the integration of dykes into the physical landscape (e.g. multifunctional dykes that incorporate car parks) and less on making the landscape resilient to floods and reducing impacts. The integration of FRM with spatial planning is weak (Restemeyer et al. 2017, p.934). All in all, this Subprogramme developed a Strategy that is essentially preventive, as it is strongly based on improvements to existing defences (dykes and barriers) (Restemeyer et al.2017, p.934).

The entire map, thus, seems to present an “optimal” sequence of decisions and measures (a preferred pathway), rather than depict a robust and flexible set of alternative possible pathways.

As noted by Restemeyer et al., the DP2014 case shows that decision-makers and policymakers faced a dilemma between the aspired ‘adaptability’ and the ‘urge-to-control’. For example, in the elaboration of the Preferential Strategy of the RE-D, the idea of adaptability was adopted, but partially (Restemeyer et al. 2017, p.920, 935). One of the main conditions for making a plan ‘adaptive’ is the elaboration of flexible strategies, which, in turn, implies identifying tipping-points, and designing adaptation pathways (by ordering various possible measures in time) (Restemeyer et al. 2017, p.921, 927, 935). ADM explicitly recommended using Tipping-points and Adaptation Pathways to make strategies “more robust and flexible” (Restemeyer et al. 2017, p.935, 931). However, the Preferential Strategy of the RE-D looks quite “*determined and not so flexible”*: it offers little room for adjustments over time, it focusses mainly on ‘prevention’ measures (to reduce flood probability) through *gradual adjustment*s of the existing defences, and although the Team recognized the need for spatial adaptation measures in addition to the traditional preventive measures, in this Strategy “*adaptability mainly gets down to gradual adjustments of certain measures*”, e.g. dyke strengthening (Restemeyer et al.2017, p.932, 934-9355).

Both the TE2100 and the DP2014 have considered and envisioned a stepwise / staged implementation of several measures over time (rather than a single measure optimized for a particular level of risk). Both cases envisaged short-term decisions and actions linked (coupled) with possible long-term options, and considered their timing (Jeuken et al. 2014, p.17, 24). In both cases, the APs maps designed display short-term measures coupled with (linked with) long-term options through adaptation pathways, which in the TE2100’s routemap clearly indicate thresholds (and Figure 2 shows decision-points for taking a decision of switching of measure, and implementation-points), whereas in the DP2014 maps moments when it is necessary to switch from a measure to another one, thus ensuring and enhancing flexibility.[[147]](#footnote-147)

Regarding the flexibility of the FRM measures (themselves) used in the maps, in both cases, it should be noted that although soft protection measures are included, there seems to be an over-reliance on measures involving the improvement of existing hard defences, reflecting a certain path-dependency which compromises the flexibility of the broader plan and FRM system[[148]](#footnote-148), and which, in the future, might demand a greater variety of (and greater balance between diverse) coastal adaptation measures, including hard and soft protection measures, retreat / managed realignment measures, green / nature-based adaptation measures, non-structural measures. The FRM measures taken in the past are thus, quite determinant and decisive of the spectrum of options available in the future (i.e. for the widening / shrinking (narrowing) solution-space). In both the TE2100 case and the DP 2014 case, the APs maps are apparently flexible into the future, because large-scale (potentially irreversible) hard defence / structural measures were put postponed to the long-term. Both the TE2100 and the Delta Program cases, the maps of adaptation pathways appear relatively flexible in the future, primarily because the implementation of new large-scale structural (hard defenses) measures could be delayed (Jeuken et al. 2014, p.18).

Besides, in the TE2100 Plan, each of the HLOs/ pathways envisioned was mapped in a georeferenced map, which significantly helped to understand the implications of each pathway in space and over time.

#### Regarding Key-element 4

In AP&M approaches the monitoring and reevaluation system (M&R) is developed to inform about measures that can be taken in response to new conditions and safeguards the “learning component” that ensures the dynamic robustness to uncertain futures and the flexibility to adapt to evolving conditions over time. The M&R system defines indicators/ signposts that must be tracked to know e.g. whether the initial plan is currently achieving its goals and whether ATPs/ thresholds or any threats to the objectives are in sight, as well as trigger-vales (that signal the need to decide on or implement a new measure) (Marchau et al. 2019). Moreover, the M&E serves to regularly review or update plans over time based on emerging knowledge and ongoing changes. The definition of the M&E, thus, makes adaptation over time explicit since the outset of plan’s formulation (Walker et al. 2001; Marchau et al. 2019). A well-designed M&E system is a “condition sine qua non” for the implementation of an adaptive plan. An adaptive approach requires that “a system for the timely detection and interpretation of relevant signals has to be installed, and that the decision-making process has to be designed to translate these signals directly into suggestions for adjustments of policy frameworks, strategies, and plans at different levels of government” (Marchau et al. 2019, p.341). Monitoring is critical to realize adaptive plans (Marchau et al. 2019).

Since the outset, the TE2100 has defined a monitoring and review system, including key-indicators to be monitored and a method to calculate decision-points and implementation-points (dependent on thresholds, lead-time for building a measure, and monitoring results); and this system started to operate since the Plan’s publication in 2012. In the DP, a monitoring and evaluation system called “Monitoring, Analyzing, Acting” (MAA) was created from 2016 onwards and has been operating since 2017, the MAA also specifies indicators to be monitored.

In the TE2100, the M&R system must inform a scheduled review and reassessment of the TE 2100 Plan every 10 years, with a mid-term review every 5 years. Similarly, the DP’s MAA system sets out a systematic review of all regional Strategies every 6 years, with an analysis every 12 years of practical experience and research findings to determine whether the new flood safety standards need to be revised (Bloemen et al. 2018, p.21).

The final TE2100 Plan explains how the timing of measures might need to be adjusted (changed) based on the monitoring of 10 key indicators (e.g. SLR, river flow, erosion rates). The results of the M&R program must inform the scheduled 10-year reviews and re-appraisals of the TE2100 Plan, with an interim monitoring review conducted every 5 years (Bloemen et al. 2018, p.9). The TE2100 5-Year Monitoring Review provided in-depth information and insights into the monitoring of the 10 key indicators, e.g. it underlined the need to distinguish changes in indicator trends from natural variability, thus guiding subsequent research. This was the first monitoring review of an APs-based plan (Bloemen et al. 2018, p.6), demonstrating the value of the key-elements 4 and 5, and which are essential to operationalize an adaptive plan.

In the DP, the monitoring and reassessment have been challenged by difficulties in detecting trends for certain variables, though offering valuable insights for advancing AP&M approaches in dynamic coastal environments (Jeuken et al. 2014). In the DP of the major difficulties was defining the right (adequate) parameters / indicators that could be monitored / tracked (and that clearly signal when it is time to take a new measure, based on a prior decision). While this works well for gradual, slowly changing parameters (like SLR for other factors (like river discharge, which is characterized by high natural variability) the identification of tipping-points and trigger-values is quite more complicated (Restemeyer et al. 2017, p.931).

River discharge, unlike SLR, can gradually increase for a period before decreasing again, fluctuating without a clear pattern. In practice, identifying signals of climate change in river discharge through monitoring is considered nearly impossible due to its high natural variability. As a result, the DP opted to assume a fixed river discharge (17,000 m³/s by 2050 and 18,000 m³/s by 2100). While the DP staff acknowledges that this contradicts the notion of flexibility: as it is not possible to rely on monitoring, decisions are based on “artificially fixed' worst-case future conditions” (Restemeyer et al. 2017, p.931).

In the DP case, a “Signal Group” has been tasked with providing information on developments and potential tipping points. The group includes knowledge institutes specialized in water, spatial planning, and climate. It reports on developments in three areas: “knowledge and innovation”, “climate change and socioeconomic developments”, and “societal preferences” (Bloemen et al. 2018, p.10).

The implementation of the MAA system within the DP reflects an effort to rethink and tailor monitoring and evaluation (to address deep uncertainties and support adaptive approaches. Since it started to operate (2017) and until 2019, several key insights and challenges have emerged:

- The MAA has promoted technical and strategic learning through annual and six-yearly reviews, which are closely tied to its information-collection and discussion procedures. Concentrating on a limited number of core questions has effectively engaged policymakers, researchers, and steering groups at both national and regional level. However, the process of gathering data and discussing findings has highlighted differing perspectives among stakeholders due to distributed responsibilities.
- The outputs of the MAA system need to better support decision-making at the regional level, as regional subprogrammes provide critical information on implementation processes.
- The initial planning of the MAA system proved to be too ambitious, some of its parts were still being developed in 2019.
- It is necessary to clarify and refine outcome criteria to evaluating whether implemented and planned measures meet the DP’s goals. This requires further research, and elaboration of the objectives to enable meaningful outcome assessments.
- Consideration is needed to better align criteria that are directly measurable with those that rely on modelling (Marchau et al. 2019, p.347).

In the DP case, until 2014/2015, the capacity to adjust (adapt) based on new insights was still quite underdeveloped in the governance process of the DP. The ‘Delta Decisions’ were created in a quite linear filtering process. Only later, between 2016 and 2018, the DP has set out its monitoring and evaluation system, clarifying what will happen if the underlying assumptions change, and explaining how monitoring will inform and allow learning from monitoring results and policy adjustment (and defining how these feedback moments might occur, in line with the ADM approach) (Restemeyer et al. 2017, p.929).

The DP was predominantly government- and expert-driven; it heavily relied on expert knowledge from the water sector, with minimal involvement from local stakeholders and citizens. As a result, the approach remained largely technocratic in nature (Van Buuren 2013; Restemeyer et al. 2017, p.929).

In the DP case, the “institutionalization” of the monitoring system only occurred between 2016-2018, including the definition of what to monitor, who should review the results, and determining when to take action. To address the dilemma between adaptability and the urge to control, Restemeyer et al. propose a greater emphasis on monitoring and learning, and assessing both current practices and external developments (Restemeyer et al. 2017, p.935). For these authors, it is essential to consider and incorporate learning and monitoring into the policy planning process, before strategies are developed (Restemeyer et al. 2017, p.936).

#### Regarding Key-element 5

Implementing adaptive plans requires a decision-making process that directly links the output of the M&E system to the prior steps of the process (Marchau et al. 2019, p.321). An adaptive plan based on formal review and ongoing learning will ideally evolve in response to some predefined triggers but also as stakeholders’ values, interests and preferences evolve over time (Marchau et al. 2019, p.147). Moreover, all AP&M approaches organize a decision-making process that is devised to adapt the plan based on evolving conditions (Marchau et al. 2019, p.394-395), making *adaptations explicit at the outset of plan design* (Marchau et al. 2019, p.66-67). In this sense, AP&M approaches imply repeating multiple iterations of their main steps over time (Marchau et al. 2019, p.147).

**Need for periodically updating the adaptation pathways.** Another key point identified is the need for regular updates of the pathways: adaptation pathways are not set in stone (fixed); their adaptations might occur according to updates to acceptable risk levels and, thus, they must be reassessed over time, instead of relying on an approach that establishes rigid (inflexible) standards (Bloemen et al. 2018, p.19).

This has been effectively ensured in the TE2100 case: new versions of its route-map have been issued since 2012, with new (Figure 3). In the DP case, despite its annual reports, the maps of the PS and DD have not been updated since 2014 (up until DP2023).

A study on the governance of the DP, following the release of the DP2014 (for 2015), concluded that ADM and the use of adaptation pathways are key outcomes of the DP. Among the top three "core qualities" of the DP, which must be maintained in the next phases, are shared ownership, coherence, and adaptivity. Several organizations, including research institutes, emphasized the importance of preserving the adaptive nature of the DP during the implementation and subsequent phases (van Buuren and Teisman 2014, *in* Bloemen et al. 2018, p.12). A formal legislative evaluation of the DP conducted in 2016 emphasized the need to preserve adaptivity within the programme: maintaining adaptivity is regarded as essential (Bloemen et al. 2018, p.13).

#### General findings

The findings from this analysis suggested that **five elements were key / essential to develop and implement a robust adaptive plan / strategies**, and operationalize AP&M, in the studied cases: 1) considering and using of a wide range of plausible future scenarios, to assess measures and pathways on their robustness and effectiveness over time; 2) specifying thresholds / Adaptation Tipping-points (as critical levels / conditions at which a given measure ceases to meet to objectives, and another measure is needed); 3) developing a robust and flexible set of measures (or, more precisely, sets of measures, each set as a pathway) to respond to changing conditions, through the design of adaptation pathways; 4) monitoring changes and effects of implemented measures and regularly reassessing / reviewing the plan; and 5) following the ongoing iterative process with its several steps that allow adapting the plan and its contents as changes occur over time. The key-elements 1, 3 and 4 are in line with the central elements of Adaptive Planning suggested by Sayers et al. (2012); Jeuken et al. (2014), and Restemeyer et al. (2017).[[149]](#footnote-149)

Importantly, ingredients that confer dynamic robustness and flexibility to the general plan, including sub-elements of Key-element 3, could also be identified, for example: choosing measures that do not foreclose, constrain or hinder future options unnecessarily, using measures that are low-/no-regrets (which have properties of reversibility, adjustability, or correctability), envisioning a phased / step-wise implementation of various projects / measures over time; using diverse types of FRM measures (measures to reduce probability, measures to reduce exposure and vulnerability, measures to reduce flood consequences) and ensuring heterogeneity, or safeguarding planned redundancy (where the risk of failure is distributed among diverse component elements of the FRM system).

The key-elements and ingredients, individually and in conjunction, have built-in and safeguarded the dynamic robustness, flexibility, and adaptability, in the general plan / strategies in both cases.

The TE2100 Project sought to guarantee ‘dynamic robustness’ (recommended by the UK Government) and adaptability mainly through: ensuring the ability to change between Options (i.e. pathways’ flexibility, the plan allows for switching of measure or pathway if necessary, the plan must be managed iteratively, and its measures or their timing can be changed); changing the timing of new measures (bringing forward / delaying measures according to the rate of change observed); using low-regrets measures (i.e. cost-efficient and beneficial under multiple scenarios), structural flexibility (i.e. adjustable engineering solutions, structures designed so that they can be adapted to changing conditions, embedding / mainstreaming adaptation into new infrastructure projects), safeguarding land for future FRM measures (to ensure that future options remain possible) (Ranger et al. 2013; Environment Agency 2012).

The DP’s ADM approach values flexibility mainly in terms of possible strategies (measures) and possible timing of implementation (Haegen and Wieriks 2015; Zevenbergen et al. 2018), i.e. possibility of switching to other measures (or modifying a measure) and anticipating / delaying measures (Vink et al. 2013, p.92). Flexibility is delivered in several ways, e.g.: by designing each strategy as an *adaptation path* (pathway), by using solutions that are ‘flexible’ *per se*, by using various pathways, by linking agendas, etc. These types of flexibility create added value in terms of cost reduction, feasibility, and benefits (DP 2014, p.90, 139). The development of *adaptation paths* contributed to ensure flexibility: the pathways can be accelerated / slowed down, or adjusted (by shifting from a measure to another), and options are kept open for the future (Gersonius et al. 2016, p.12).

##### A. Ways of ensuring, delivering and enhancing dynamic robustness and flexibility in the Plan / Strategy

| **Table A. Ways of ensuring, delivering and enhancing dynamic robustness and flexibility in the Plan / Strategy** | | |
| --- | --- | --- |
|  | **TE2100 case** | **DP 2014 case** |
| Choosing actions/ measures that remain flexible with respect to uncertain future changes and/or to create a robust system that may better cope with extreme events (Jeuken et al. 2014, p.3).  Design measures that are able to cope with multiple potential future conditions (robust actions), or design measures that leave room for adaptation if needed (Jeuken et al. 2014, p.24).  Choosing measures that are “effective under the widest set of all plausible future scenarios’ (Jeuken et al. 2014, p.3, based on Sayers et al. 2012).  Choosing measures that do not foreclose future options or unnecessarily constrain future choices’ (Jeuken et al. 2014, p.3, based on Sayers et al. 2012). | **Yes**, e.g. measure to enhance system robustness | **Yes**, e.g. measure to enhance system robustness. Flexible measures like river widening, beach nourishment |
| Use robust measures that increase a system’s resilience (capacity to ‘cope with and recover from uncertain future flood hazards’), or ‘incorporate large safety margins and increase the resistance of the system’ (Jeuken et al. 2014, p.10, 16, 18). |  |  |
| Envisioning alternative options (i.e. alternative measures, and alternative pathways) and possibilities for switching between them, through adaptation pathways (Jeuken et al. 2014, p.1). Allowing for switching between different options in the future’, and, in this way, remaining flexible (Jeuken et al. 2014, p.10).  Containing / having multiple pathways (which allow switching between different options in the future) (Jeuken et al. 2014, p.18). | **Yes.** | Partially. Some PS only have one path. Yet, the possibility of switching to different measures is safeguard. |
| Short-term decisions and measures are coupled with / linked to long-term options (Jeuken et al. 2014, p.1) | **Yes** | **Yes** |
| APs map shows “clearly marked thresholds defining when to decide to switch from one action to another”, which “ensures maximum flexibility” (Jeuken et al. 2014, p.24). | Yes | No |
| Envision a phased / stepwise implementation of several adaptation measures or various smaller projects (though there may be cases where a single large-scale investment is more cost-effective) (Jeuken et al. 2014, p.10, 16, 18).  Ensure that measures can be implemented stepwise, or that there is the possibility of switching to other measures (Jeuken et al. 2014, p.18). | Yes | Yes |
| Several measures are implemented iteratively over time to keep risk below target levels, while **keeping open** **options (alternatives)** to manage future risk (Ranger et al. 2013, 247, 249, 239; Reeder and Ranger 2011, p.13)  In the Route-map approach, diverse measures are implemented over time, and alternatives (options) are left open to cope with plausible (different) future conditions (Reeder and Ranger 2011, p.3) | Yes | Partially met (few alternative options in some PS) |
| Each pathway contains a set of measures (individual or grouped) that are implemented over time to manage changing risk (Reeder and Ranger 2011, p.9; Lowe et al. 2009, p.89; Jeuken and Reeder 2011, p.4; Jeuken et al. 2014, p.18). | Yes | Yes |
| Several pathways are usually designed in an APs’ map. Each pathway is itself flexible (e.g. it is possible to switch from a measure to another), but it is also possible to move from a pathway to another, depending on the rate of change that actually occurs (Reeder and Ranger 2011, p.9; Lowe et al. 2009, p.89; Jeuken and Reeder 2011, p.4; Jeuken et al. 2014, p.18). | Yes | Partially met. Not all PS contain more than one pathway |
| Use / promote diversity of measures (within and across sectors and actors) (Jeuken et al. 2014, p.10, 16, 18). | Yes. | Partially met (in some PS there is a predominance of preventive (protection) measures to reduce flood probability, over other types of FRM measures |
| Link (potential) future actions to current problems, e.g. by searching for **win-win options** (Jeuken et al. 2014, p.24). | Yes | Yes |
| Use low-regrets measures (with properties like reversibility, adjustability, correctability) (Jeuken et al. 2014). | Yes | Yes |
| Integrate adaptation into coastal planning and management (mainstreaming) (Jeuken et al. 2014, p.10, 16, 18). | Yes | Yes |
| Seize opportunities once they arise, to integrate FRM / adaptation measures (Jeuken et al. 2014, p.24). | Yes | Yes |
| targeted monitoring, continual reassessment of scenarios of the future, and the redefinition of policies, strategies and structure plans, are accounted for in the Plan (Jeuken et al. 2014, p.3, based on Sayers et al. 2012). | Yes | Yes |
| The Plan / strategy is ‘designed to be adjusted over time as more is learnt about the future’ or as changes occur. The timing for new interventions, and the interventions themselves, can be changed (modified) over time, therefore, flexibility is built-in into the long-term strategy itself (Ranger et al. 2013, p.249). | Yes | Yes (but less clear how such adjustments will be translated in the APs map) |

##### B. Key concepts from APs / AP&M literature, and their use and conceptualization (understanding and meaning) in each case

| **Table B. Key concepts in / from APs-based AP&M literature, and their use and conceptualization (understanding and meaning) in each case** | | |
| --- | --- | --- |
| **Key concepts** | **TE2100 case** | **DP 2014 case** |
| **Name of AP&M approach** | Dynamic Adaptive Planning, also called Managed Adaptive approach, or Iterative Risk Management. It was based on the concept of ‘Dynamic Robustness’. | Adaptive Delta Management (initially called Adaptive Delta Planning)  ADM builds on the DAPP approach (of Haasnoot et al. 2013) and was inspired by the TE2100 Project. |
| **Sub-methods within it:**  **Adaptation Pathways (APs)**  **Thresholds/ ATPs approach** | It included the Adaptation Pathways approach, also called Route-map approach, or Decision Pathways approach; as well as the “Threshold approach / Scenario-neutral approach”  The APs approach allowed exploring possible adaptation measures over time and sequencing them (in pathways); it also allows identifying the timing for such measures under different scenarios (Ranger et al. 2010).  The APs’ map identifies thresholds (points where an action no longer meets the predefined criteria, and where it is necessary to take an additional action, or switch to other action or pathway (Ranger et al. 2013). | ADM explicitly implied using the APs and ATPs methods. |
| **Process of steps provided;** | It was not provided a pre-established process / cycle of steps to be followed. However the Adaptive Planning approach of the TE2100 was based on the approach provided by Willows and Connell 2003. | It was provided a diagram of the ADM process to the DP and its Subprogrammes, which is based on (adapted from) the diagram of the DAPP process (Haasnoot et al..2013). |
|  |  |  |
| **Scenarios** | Four SLR scenarios + four socioeconomic scenarios considered.  Four plausible future SLR scenarios specifically produced for the TE up to 2100, namely: the *DEFRA* 06 central (0,9m, deemed the “most likely”), initial *High++* (4,2m), *Medium-High* (1,5m), and the later revised High ++ called *High+* (2,7m). + Four socioeconomic scenarios (A to D) | Four Delta Scenarios, each combining climatic and socioeconomic changes (i.e. merged climate and socioeconomic scenarios). However, the maps of adaptation paths were developed taking into account one of the four Delta Scenarios.  Four *Delta Scenarios* (*Busy*, *Steam*, *Rest, Warm*), which consist of four different plausible futures that show how the climatic and socioeconomic conditions might change until 2050 and 2100, not predictions. They differ in terms of rapid / moderate CC and socioeconomic growth / decline. Each presents its figures for SLR, river discharges, and soil subsidence.  Due to uncertainty about the rate of CC, the DP decided to work with several possible future *Delta Scenarios* and draw up strategies for them (DP 2013, p.6). |
| ***Sell-by date* of measures (Terminal Station in APs map)**  **Thresholds / Adaptation Tipping-points** | The term “threshold” most often refers to critical threshold levels expressed in terms of SLR or rising water levels. Represent critical limits / values for the existing FRM system, namely for its sensitivity and vulnerability. For example, the level of SLR at which a flood defence or any FRM measure ceases to be effective, and which requires switching to a new measure (before the threshold in reached).  In the case of the TE, thresholds refer to critical levels, i.e. critical threshold levels / values for (in) the vulnerability and sensitivity of the FRM system, namely the level of SLR at which pre-existing sea defences fail and any other levels that pose limits to adaptation. These included, for example, the limit for the existing system of walls and embankments, the level of SLR at which the existing Thames Barrier (as it is designed) no longer meets the target protection level (1 in 1000); the engineering limit of the Thames Barrier with modification works; and the limit at which it will be difficult to continue to protect the Estuary and London in the current ways and retreat would be required. Thresholds levels are indicated in the x-axis of the route-map which reflects the increase in extreme water levels in the TE. The Team examined key thresholds of climate change and water level rise at which other measure or Option will be required.  The identification of thresholds (and detection of their date in different scenarios) informed the development of *Options* (pathways) (TE2100 Appendix L 2009, p.5, 10, 12). The thresholds were used to sequence and assemble the possible measures into ‘*packages* of measures’ (i.e. to design pathways) (Jeuken et al. 2014, p.17). The route-map shows the thresholds at which it is necessary to switch from a measure to another. The identification of thresholds helped to enhance the flexibility of the Plan (ibid).  This ‘threshold analysis’ allowed a more ‘*scenario neutral approach*’, and it is deemed one of the main successes of the TE2100 (TE2100 Appendix L 2009, p.5, 10, 12). The Route-map approach is more *scenario neutral* because decisions do not require information about the likelihood of different scenarios (Ranger et al. 2013, p.254; Reeder and Ranger 2011, p.9).  The ‘APs approach’ required the analysis of thresholds – i.e. points under which a measure no longer meets the predefined decision criteria (Kwadijk et al. 2010) and it is necessary to take a new measure or switch to other pathway – and examining under what conditions the Plan fails and preparing actions to guard against this (Ranger et al. 2013, p.250). | Adaptation Tipping-Points of Kwadijk et al. 2010). Refer to points at which the existing system ceases to meet the requirements; a *tipping-point* occurs when, due to climatic or socioeconomic changes, an existing measure, policy, or infrastructure, becomes insufficient to comply with defined criteria (due to physical, technical, financial constraints or socially unacceptable effects); hence, ATPs’ analysis helps determining when decisions and new measures must be taken.  The ATPs method, as used in the DP, implied specifying the tipping-point of diverse possible measures (the degree of change under which each measure starts to perform inadequately), and assessing when this might occur at earliest and latest, and analysing whether it is necessary to adjust / change such measure or shift to another measure. Once a tipping-point is in sight, a switch to a new measure is needed.  In the DP2014, **the maps of adaptation paths do not explicitly show / illustrate the ATPs**, the X-axis displays a timeline (with the short-, mid- and long-term periods represented). By crossing ATPs with scenarios, the Subprogrammes and DP Staff could obtain information on the timing for new measure. Initially, the development of alternative paths required examining “how long the current policy will suffice” and “when the tipping-point (at which such policy is no longer tenable) will be reached” (its moment in different scenarios.  ATPs / terminal stations can be implicitly understood as the end of each box that represents a measure. |
| **Adaptation Pathways** | Adaptation pathways are called ***High-level Options* (HLOs)**, also called *Options*, routes, or decision pathways. The pathways for the post-2070 period are called *End-of-the-Century Options*.  Each pathway consists of a set of sequenced individual and / or combined adaptation measures.  Each HLO is a package of FRM measures sequenced that can be implemented over time to manage risk stepwise. Each measure (individual or portfolio) is represented in a box that finishes at the SLR level at which it ceases to be effective (threshold for such measure); the arrows link to other measures available (which must be implemented / in place before the prior measure reaches a threshold (or before the threshold level is reached). Each HLO comprises a sequence of measures and / or portfolios, and the points at which one portfolio changes to another portfolio depend on key thresholds for FRM assumed. Each HLO is made up of a set / number of measures (individual and / or in portfolios) that are implemented in sequence to manage flood risk in a staged way, up to 2100.  In the first maps of the HLOs (Figure 1), the x-axis illustrated rising water levels (the map of APs was a threshold-based map). In more recent versions (Figure 3), the map is time-based (timeline / years represented in the x-axis).  The approach ‘*focuses on sequencing a suite of measures in order to cost-effectively manage current risk while maintaining the flexibility to cope with the range of possible future sea level rise*’ (Reeder and Ranger 2011, p.13).  To deal with uncertain future change, the TE2100 Team sought to *build-in* (embed) flexibility (i.e. *dynamic robustness*) into the Plan and its measures (Ranger et al. 2013, p.249). This was done with the ‘Route-map / APs approach’. By using the ‘APs approach’, the TE2100 sought to safeguard the *robustness* and *flexibility* of the Plan (Jeuken et al. 2014, p.10). In the TE2100, the robustness and flexibility of the Plan was safeguarded by using the ‘Route-map / APs method’; this approach allows for switching between different measures or pathways in the future (Jeuken et al. 2014, p.10). The ‘Route-map approach’ helped to incorporate (*build-in*) and enhance the *robustness* to uncertain future conditions and *flexibility* to deal with change, into the plan itself and its *strategies* (pathways).  The adoption of the ‘APs approach’ to design several possible pathways (of adaptation / FRM) is one of the main and most innovative elements of the TE2100 (Ranger et al. 2013, p.233, 239, 258). The development of adaptation pathways (*Options*) is one of the essential elements of its *Adaptive Planning approach* (Ramsbottom and Sheppard 2017, p.5). APs approach allowed the development of *robust flexible strategies* / Options, it is one of the main elements of the Adaptive Planning approach of the TE2100 (Jeuken and Reeder 2011, p.2).  This element involved developing ‘*robust* *and flexible*’ sets of measures to deal with uncertain changes, using the APs approach’, i.e. devising each set of measures as an alternative pathway. This required searching for measures that are *robust* and / or *flexible* and preparing a ‘*robust flexible*’ set of measures. The set of measures should contain the following properties: *robustness* (ability to achieve the objectives despite how the future unfolds and be effective under a wide range of future scenarios), *flexibility* (ability to be changed over time as changes occur or as new information arises, and keep options open / not foreclose future options), and be *no / low-regret* whenever possible (with properties like *reversibility*, adaptability, etc.). By using the ‘APs approach’, it was possible to design of several packages of sequenced measures, i.e. pathways. Each pathway can be deemed a set of measures sequenced and implemented over time to manage changing risk; and several pathways were designed. Together, the various pathways offer robust and flexible sets of measures (a set of various robust flexible strategies), which form a *robust* and *adaptive* plan. | ***Adaptation paths*,** i.e. multiple strategies that can be alternated between (i.e. different strategies that can be implemented alternatingly depending on climatic and socioeconomic developments)  Each path is a sequence of measures over time that can be activated before ATPs occur. The various paths provide coherent sequences of measures to achieve predefined objectives.  Each (regional) Preferential Strategy (PS), and most Delta Decisions (DD), contain a map of adaptation paths, with one or more paths (i.e. the pathway to be followed or the pathways available). Each *path* is a logical set of measures including measures for the short-term and options for the mid- and long-term. Most often, each PS *provides sets of measures* – each *set* offers a *path* and sets of measures (several *paths*) were elaborated separately for each Region. The diverse paths offer different possible trajectories the system may follow to meet objectives.  The maps of adaptation pathways of the PS and DD are time-dependent (time/ years are illustrated in the x-axis, rather than ATPs). Although 4 Delta Scenarios were available, each map of adaptation paths was developed considering one (a single) Delta Scenario – Steam??? |
| **Transfer station / transfer-point**  **(prior to reaching an ATP, as represented in APs map)**  **Triggers / trigger-points / trigger-values** | The concept of “decision-points” used in the TE2100 resembles the “transfer station” of a standard APs map.  A decision-point is a point at which a given action (pertaining to an HLO) must be chosen and approved; its estimation considers the “lead-time” required for designing and constructing such action. It is expressed as a value in an indicator that is monitored (e.g. SLR), and it is related with the threshold set for such indicator. The decision-points help to ensure that measures are timely taken and cost-effective. A decision-point is a point at which a decision on a given measure must be made, which triggers its planning and construction.  TE2100 Plan explains how to estimate a decision-point for a given measure; it is necessary to: identify the threshold-value in an indicator (e.g. the water level) at which the measure will be needed; analyze when such threshold will occur in a given scenario; then, add the lead-time required to design and construct such measure and also an uncertainty band (e.g. about SLR rate), and also consider the observed/ recorded values in the indicator. In this way, decision-point can be expressed as a value in an indicator. Hence, the decision-point is conditioned by the monitoring and observation of actual changes in indicators (e.g. recorded values of SLR), thus, the timing of this decision-point (and of the implementation-point) may change depending on the monitoring results. A decision-point can also be derived under different scenarios, including under updated projections that might arise, which may require updating the lead-times, implementation-points, or decision points. Certain values in the indicators will act as triggers of changes, e.g. a rapid change in an indicator may trigger an earlier decision. | A similar concept to “Transfer stations” is used in some maps of adaptation paths (for the Preferential Strategies): the concept of “branching-point”, where one of the following measures can be taken or two can be combined.  Triggers refer to certain values in indicators at which it is necessary to activate actions or reassess PS.  DP adopted the definition provided by ADM (and based on DAPP): critical values of the signpost variables, beyond which additional / new actions should be taken, or decisions should be reconsidered.  Triggers serve to indicate when a new action planned in the PS should be activated; most often refer to critical values at which it is necessary to activate actions, which act as warning signals for the implementation of actions or reassessment of the PS or DD.  Triggers’ (critical values beyond which it is necessary to make adjustments or change of measure, and which must be monitored. |
| **Monitoring, revalution and review system** | Monitoring and Review System. It sets out 10 indicators of changes that must be monitored throughout the Plan’s life, as well as a 10-year review of the Plan, and a 5-year mid-term monitoring review. It allows for timely adapting the Plan, its pathways, their measures or their timing, as new knowledge becomes available, and as changes are observed. The first 10-year review has led to adjustment to the End of the Century Options considered, demonstrating the adaptive character of the Plan and its pathways. Some decision-points have also been modified.  The timing for new measures, and the measures themselves, can be modified (changed) over time, in this way, *flexibility* is built-in into the long-term strategy itself (Ranger et al. 2013, p.249).  The TE and flood risk will change over time, but the Plan *“can accommodate future change and can be adjusted when the rate of change is either faster or slower than originally predicted*. *This flexibility was included to ensure that the actions in the Plan remain appropriate and are implemented at the right time”* (EA 2016, p.15). | Monitoring, Analysing and Action system (MAA). Since 2017, the MAA has been used to monitor developments that may affect the pace or direction (course) of the PS, and to evaluate if the DP is ‘on track’ or if it is necessary to adjust the course or pace, or reconsider targets or measures (e.g. if developments demand a switch of strategy or measures, or a modification in their timing). Moreover, the DP will carry a review every 6 years to check, based on monitoring results, if it is necessary to maintain or adjust the course or the pace (i.e. the measures or their timing). Based on new knowledge and monitoring, the strategies and / or their measures may be anticipated or postponed, or adjusted in terms of scope and design.  ADM requires ongoing monitoring of drivers of change, and the regular reassessment of strategies and measures. The monitoring system keeps track of changes and detect ATPs in a useful timescale. The DP’s *monitoring* *and revaluation* system specifies the variables to be monitored to allow a timely adaptation of the strategies (their measures, or timing). Adaptiveness is patent in this system and in the yearly updated DP reports (Zandvoort et al. 2018, p.191-192). The DP’s approach is ‘flexible’ since it allows the adjustment (adaptation) of a strategy to changing conditions or new insights that may arise (e.g. climate change) (Jeuken and Reeder 2011).  ADM requires the monitoring of changes or developments and effects of measures, and the reassessment/review of the Plan (its strategies, or measures, planned or implemented), and their adjustment if changes prompt so (DP 2016, p.59). |
| **Robust plan** (requisite for the plan) | A plan that performs well against several decision criteria under a wide range of possible futures.  To deal with uncertain change and future conditions, the TE2100 Project decided to develop a ‘*dynamic adaptive plan*’ that was *robust* and *adaptive*, and, to construct such plan, it devised the ‘Route-map / APs approach’. The Team sought to *build-in* ‘*dynamic robustness’* (*flexibility*) into the Plan and its measures, and the ‘APs approach’ was essential to achieve this. Such ‘*dynamic adaptive plan’* should include a ‘*robust and flexible*’ set of measures.  The production of ‘*flexible adaptable Options*’, i.e. pathways, and the analysis of thresholds that it implied, were essential to develop a plan (manage flood risk until 2100) that was ‘*adaptable* / *adaptive*’ and ‘*dynamic robust*’ in the face of change and uncertain future conditions. *Dynamic robustness* (*flexibility*) and *adaptability* are basic properties of the Plan (TE2100 Appendix L 2009, p.2-4, 10, 199). | A strategy that performs satisfactorily under a wide variety of futures. It is future-proof and provides a solution for tasks in all *Delta Scenarios.*  In 2010, the DP Commissioner underlined that one of the main challenges of DP was “*dealing with uncertainties in the future climate, but also in population, economy and society. This requires a new way of planning, which we call adaptive delta planning’* as a ‘*new way of planning* *that seeks to maximize flexibility; keeping options open and avoiding ‘lock-in’”* (Kuijken 2010 *in* Haasnoot et al.2013 p. 490; Werners et al. 2016). The Commissioner called for an *Adaptive Delta Planning* that fosters flexibility by *keeping options open and avoiding lock-in* (Kuijken 2010).  To ensure a *‘sustainable and robust FRM and FS’* and cope with climatic extremes, the country should be ‘*prepared for various scenarios*’, and measures and strategies should provide *flexibility* to respond to new insights and changing conditions, either by ‘*stepping up measure*s’ or ‘*changing of strategy*’ if necessary (DP 2014, p.6).  The 2^nd^ and 3^rd^ principle of ADM are related to *robustness and flexibility* (DP 2013, p.103). ADM aims at developing strategies that are *robust* (i.e. that can deal with multiple futures), and *flexible* or *adaptable* (Marchand and Ludwig 2014). **Robustness involves “*performing satisfactorily under a wide variety of futures*’, and flexibility is related with the capacity of being “*easily adapted to changing or unforeseen future conditions*” (Haasnoot 2013, *in* Marchand and Ludwig 2014, p.8).** The *ADM Implementation Guide* claims that strategies should be both *robust* and *flexible*: a robust strategy is one that “*works in all plausible futures*”, while flexibility means that “*depending on the contextual circumstances - you can cut one strategy off and* *switch to another one*” (DP Staff member, *in* Restemeyer et al. 2017, p.930). According to the Guide, to find or develop *robust* *flexible strategies*, it is necessary to identify tipping-points and design adaptation pathways (Restemeyer et al. 2017). The design of *robust flexible* *strategies* requires exploring various possible measures under a range of plausible futures and searching for those that are *robust* and *flexible* (Marchand and Ludwig 2014). |
| **Flexible adaptive / adaptable plan**  (requisite for the plan) | A plan that can be changed over time as new insights and knowledge are gained or as conditions change, and thus, is adaptable / adaptive.  As noted in the Technical Report, ‘*to continue to manage flood risk from the tidal Thames will require substantial investment over the next 50 years and it will be essential that adaptability and flexibility are key parts of the plan* (TE2100 Appendix L 2009, p.2). The TE2100 devised and applied a new method for designing a Plan that is *flexible* and *adaptable* to an uncertain future and change (TE2100 Appendix L 2009, p.4) – i.e. an ‘adaptive plan’ for FRM (Ramsbottom and Sheppard 2017, p.19).  The TE2100 Project sought to guarantee ‘dynamic robustness’ (recommended by the UK Government) through: pathways’ flexibility (the plan should be managed iteratively, and its Options (pathways), their measures and their timing could be changed); the use of low-regrets measures (i.e. cost-efficient and beneficial under multiple scenarios), structural flexibility (i.e. adjustable engineering solutions).  In the TE2100’s *Dynamic Adaptive Planning approach*, a plan (and its strategies) is ‘*implemented iteratively*’ and is ‘*designed to be adjusted over time as more is learnt about the future*’ or as changes occur (Ranger et al. 2013, p.249). In this approach, the timing of new measures (*interventions*), and the measures themselves, can be changed over time – in this way, flexibility is built-in into the long-term strategy itself (Ranger et al. 2013, p.249). | A strategy that can be easily adapted to changing and unforeseen future conditions. One that can be speeded up / slowed down and allows shifting to another measure or *path*, if necessary.  The *ADM Implementation Guide* claimed that strategies should be both *robust* and *flexible*: a robust strategy is one that ‘*works in all plausible futures*’, while flexibility means that ‘*depending on the contextual circumstances - you can cut one strategy off and* *switch to another one*’ (DP Staff member, *in* Restemeyer et al. 2017, p.930). According to the Guide, to find or develop *robust* *flexible strategies*, it is necessary to identify tipping-points and design adaptation pathways (Restemeyer et al. 2017).  Consequently, the DP has assumed that the strategies developed should be *robust* *and flexible* (DP 2013, p.103). The DP defines a *robust strategy* as one that is *future-proof* and provides a solution for the tasks arising in all the scenarios considered, while a *flexible strategy* is one that can be relatively easy accelerated or delayed, or that allows a switch to other strategy (a shift from a measure to another) (DP 2013, p.103). The ultimate goal is that the FRM system meets the requirements ‘*at all times*’ (all scenarios), which requires solution ‘*able to adapt to new insights and circumstances*’ and ‘*sufficient options to remain open in the future to take the required measures*’ (DP 2013, p.102). The DP searched for short-term measures that are adequate in the long-term and enhance the system’s robustness and flexibility, making it suited for diverse scenarios (DP 2010, p.69).  The DP has used ADM to devise *robust* *flexible strategies*. Flexibility is delivered in several ways, e.g.: by designing each strategy as an *adaptation path* (pathway), by using solutions that are ‘flexible’ *per se*, by using various pathways, by linking agendas, etc. These types of flexibility create added value in terms of cost reduction, feasibility, and benefits (DP 2014, p.90, 139).  The DP’s *flexible adaptive approach* values flexibility mainly in terms of possible strategies and possible timing of implementation (Haegen and Wieriks 2015; Zevenbergen et al. 2018), i.e. possibility of switching to other measures (or modifying a measure) and anticipating / delaying measures (Vink et al. 2013, p.92).  **One of the goals of the DP is to make the country ‘robust’ to withstand *greater climatic extremes* and, thus, ‘*prepared for various scenarios’*; and strategies should provide flexibility to respond to change, by ‘*stepping up measures*’ or ‘*changing strategy*’ if necessary (DP 2014, p.6). The DP 2010 defines a *robust system* as ‘*one that can withstand extreme events and accommodates different future developments*’, and a *flexible system* as ‘*one that can easily adapt or be adapted to changing circumstances*’, e.g. ‘giving room to rivers’ is a measure that increases robustness, while sand replenishment is a measure that maintains flexibility (DP 2010, p.69). Strategies should ensure ‘*flexibility’* (also called *‘adaptability’*) and ‘*robustness*’ (also called *resistance to extreme events*) (DP 2010, p.4).** |
| **Ways of delivering Flexibility;**  **Dynamic robustness ,**  **Adaptability** | The TE2100 Project sought to guarantee ‘dynamic robustness’ (recommended by the UK Government) and adaptability mainly through: ensuring the ability to change between Options (i.e. pathways’ flexibility, the plan allows for switching of measure or pathway if necessary, the plan must be managed iteratively, and its measures or their timing can be changed); changing the timing of new measures (bringing forward / delaying measures according to the rate of change observed); using low-regrets measures (i.e. cost-efficient and beneficial under multiple scenarios), structural flexibility (i.e. adjustable engineering solutions, structures designed so that they can be adapted to changing conditions, embedding / mainstreaming adaptation into new infrastructure projects), safeguarding land for future FRM measures (to ensure that future options remain possible) (Ranger et al. 2013; Environment Agency 2012).  The *Dynamic Adaptive Planning approach*, also called ‘*managed adaptive approach*’, is reflected in the way of managing flood risk (EA 2012, p.34; HM Treasury 2009, p.22). The *Dynamic Adaptive Planning approach* (*managed adaptive approach*) seeks to manage (reduce) risk iteratively over time: it defines short-term measures and, at the same time, lays out possible future measures, which fosters flexibility (Ranger et al. 2013, p.248). Under uncertain future changes and conditions, flood risk management required an adaptive approach where *‘measures are introduced at different times to limit the increase in flood risk* (*due to climate change*) *to acceptable values*’ (Ramsbottom and Sheppard 2017, p.1, 3).  The ‘*managed adaptive approach*’ involves planning several measures to manage (reduce) flood risk over time (EA 2012, p.34). (…) An *Option* (pathway) is a package of measures (actions) implemented over time to achieve the policy recommended throughout the century. The *managed adaptive approach* is realized by sequencing several measures over time. It was also necessary to determine critical conditions under which each measure will be needed (EA 2012, p.34).  In the ‘Route-map approach’, measures are implemented iteratively over time to keep risk below acceptable levels cost-effectively, while keeping open options (alternative measures) to manage future risk, thus, maintaining *flexibility* (Ranger et al. 2013, p.249, 239).  The Route-map / APs approach resulted in an *adaptive plan* that provides a suite of pathways (sequences of measures to manage risk) that maintain *the flexibility to cope with the range of possible future sea level rise* (Reeder and Ranger 2011, p.13, 8).  In the Route-map / APs approach, the timing of new measures, and measures themselves, can be changed (adjusted, adapted) over time – in this way, *flexibility* is built-in in the long-term strategy (pathway) (Ranger et al. 2013, p.249; Reeder and Ranger 2011, p.9). Not only are the HLOs (pathways) flexible (for example, it is possible to move from a given measure to a new one), but it is also possible to move from one pathway (HLO) to another depending on the actual rate of change that occurs (TE2100 Appendix L 2009, p.3; Lowe et al. 2009, p.89; Reeder and Ranger 2011, p.9).  In the ‘APs approach’, *dynamic robustness* / *flexibility* is *built-in* and ensured in the following ways:   - **Through pathways’ flexibility** (Ranger et al. 2013). In the ‘APs approach’, the Plan’s strategies are designed to be adjusted as changes occur or more is learnt about the future (Ranger et al. 2013). In specific, the timing of new measures, and the measures themselves, can be changed (adjusted, adapted) over time, in this way, *flexibility* is built-in into the long-term strategy (Ranger et al. 2013). - The APs approach involves a **stepwise implementation of measures** and **keeping open the possibility to switch to other (alternative) measures** (Jeuken et al. 2014). In this approach, flood risk is managed (reduced) iteratively: the approach displays short-term measures and possible future measures (alternatives), which fosters flexibility (Ranger et al. 2013). In the APs, measures are implemented stepwise over time. Instead of taking a decision now on the ‘best’ measure for given future scenario (which could result in maladaptation if this scenario did not occur), the ‘APs approach’ encourages planners to reflect on *what if* scenarios and adopt a ‘more flexible approach’ where decisions and measures are taken over time to continuously adapt while maintaining as much flexibility as possible about future measures (options) (Jeuken and Reeder 2011; Isoard and Winograd 2013; Ranger et al. 2013).^[150]^ One way of building-in *‘flexibility*’ is keeping open options (alternatives available) to manage future risk (Ranger et al. 2013), not foreclosing or constraining possible future measures unnecessarily (Jeuken et al. 2014; Sayers et al. 2012). This implies finding measures that do not block further measures that might be needed in the future and avoiding irreversible investments as much as possible (Jeuken and Reeder 2011; Ramsbottom and Sheppard 2017). The possibility of implementing certain measures or pathways should not be prematurely closed-off, e.g. by actions of third parties (HM Treasury 2009). - By using **‘no-/low-regret’ measures**^[151]^ in the route-map, in the near-term (Ranger et al. 2013; Reeder and Ranger 2011; Ramsbottom and Sheppard 2017). In the TE2100, some pathways contain *low-regrets* early measures, e.g. improving / upgrading existing defences^[152]^ (ibid). This measure will cost-effectively reduce risks and, at the same time, leave ‘*open the possibility to scale up action in the future*’ and ‘buy time’ to monitor and learn before making a major investment (Ranger et al. 2013). The Team strategically placed ‘low-regret measures’ early in the route-map (Reeder and Ranger 2011). For example, the measure ‘*raising other defences in the TE*’ (present in several HLOs) is a low-regret measure that helps to extend the lifetime of the existing defence system and ‘buy time’ before it is necessary to take a more irreversible decision on the long-term measure (instead of taking an more ‘inflexible’ decision now) (Reeder and Ranger 2011; Ranger et al. 2013). All the *Options* (pathways) designed include small incremental measures in the near-term (e.g. upgrades to existing defences) and leave irreversible measures as far as possible into the long-term future, to make the best use of information that will arise (HM Treasury 2009,). *Flexibility* was available, in part, because the TB was designed to provide a high standard of protection up to 2030 (adaptation is not urgent) and cost-effective *low-regret* measures were available (Ranger et al. 2013,). Delaying a major investment is justified when its benefits are highly uncertain now (Ranger et al. 2013). Hence, using low-regrets measures, and avoiding irreversible investments as much as possible, are important sub-elements of this key element of Adaptive Planning (Ramsbottom and Sheppard 2017). - Through **‘structural / engineered flexibility’**. This type of flexibility was incorporated in several measures. Structural flexibility can be delivered, e.g. by over-rotating the TB in case of greater SLR than predicted, by using ‘safety margins’ (over-engineering infrastructures to cope with greater change than predicted), by purchasing land to construct FRM measures in the future, etc. If structural / engineered flexibility is considered, flood defence structures can be adjusted or improved in the future at limited additional costs. Including structural flexibility is effective where the additional cost is low (Ranger et al. 2013). - **By seizing of opportunities to integrate** the proposed measures with other agendas or multi-objective investments (Jeuken et al. 201), and mainstreaming climate adaptation into works for maintaining or improving defences (Ramsbottom and Sheppard 2017). - **By having the possibility to alter the timing for implementing measures**. In the APs approach, a measure can be postponed or advanced in time, according to changes and new information that arises.   These were the main ways of incorporating and enhancing the robustness and flexibility into the general Plan, its pathways and measures. However, in a FRM plan, *flexibility* tends to be limited by the *nature* and *lifespan* of measures already applied, namely flood protection structures (e.g. barriers) which are implemented for many decades. The pathways of the TE2100 are apparently flexible into the future, in part, because new large-scale structural measures could be postponed. Other types of measures (e.g. soft protection, flood-proofing buildings), which can be applied in small steps, only contributed partially to the Plan, it contains mostly measures to improve the existing defence system (Jeuken et al. 2014). | The central idea of ADM is developing diverse trajectories that the dynamic system may follow as time unfolds – i.e. pathways. ***Flexibility*** is ensured through adaptation pathways that are planned in anticipation to deal with future changes (Zandvoort et al. 2018, p.190, 189). ADM ‘*leads to a composite strategy, or a set of alternative strategies with intermediate possibilities for revisions*’ (Rhee 2012, p.14 *in* Zandvoort et al.2018); its main outcome is a pathway or a set of pathways that identifies the measures available and schedules them (according to uncertain drivers of change).  In ADM, adaptiveness is related with: having (the availability of) different trajectories that can be applied to achieve long-term goals, in different plausible scenarios, depending on developments over time (Zandvoort et al. 2018, p.191).  Flexibility is valued in terms of: (1) allowing the switching between measures (or pathways), through Adaptation Pathways (i.e. the possibility to switch to other measures or pathways when needed, in face of uncertain future changes) (2) the possibility of altering the timing for implementing measures (Alphen 2013 *in* Zevenbergen et al. 2018; Zandvoort et al. 2018, p. 191; Haegen and Wieriks 2015). **The DP sought to ensure that the chosen strategies and measures provide flexibility to respond to new insights and changes, in terms of “stepping up measures” and / or “changing strategy” if necessary (DP 2014, p.6).**  In ADM, a Strategy must be flexible in terms of: the possibility of alternating of measure or pathway (trajectory). Flexibility also refers to the possibility to alter the course if an ATP of the current measure (or a given measure) comes earlier than expected (Zandvoort et al. 2018, p.191), i.e. the flexibility to deliberately choose a different measure or pathway. A flexible approach allows the switching between strategies through APs (Zevenbergen et al. 2018). This implies having the possibility (*options*) for switching between strategies (DP 2013, p.77).  ADM aims to ensure flexibility. The ‘APs method’ delivers flexibility in terms of having alternative measures and the possibility to switch from a measure, or path, to another, if developments and changes prompt so.  *Flexibility* is ensured through adaptation paths that are planned now (in anticipation) to deal with future changes (Zandvoort et al. 2018, p.190, 189).  **The development of *adaptation paths* contributed to ensure flexibility: the pathways can be accelerated / slowed down or adjusted (e.g. by shifting from a measure to another), and options are kept open for the future (Gersonius et al. 2016 p.12).**  In ADM, a strategy should also be flexible in terms of timing, i.e. allow the possibility to postpone or advance a measure in time (Zandvoort et al. 2018, p.191). This means flexibility to alter the timing for implementing a measure (Alphen 2013, *in* Zevenbergen et al. 2018). ADM seeks to *take the right measures at the right moment* (when they are really needed to achieve the desired objectives). Adaptiveness and flexibility are related with the possibility to modify a decision about the timing for a measure (Zandvoort et al. 2018, p.191).  Zandvoort et al. (2018) ADM uses two types of adaptiveness: *adaptive planning* and *adaptive capacity*. Adaptive planning requires preparing short-term measures considering long-term effects and avoiding lock-ins/-outs; which implied assessing measures under different scenarios and identifying those with greater cost-benefit in the long-term. Adaptive capacity of the managed system can be enhanced by enlarging the decision space (which increases flexibility); this implies *keeping options open* / possibilities of altering the course. These types of adaptiveness are related: one enables the other (Zandvoort et al. 2018).  In ADM, **adaptiveness** is also related with *keeping options open*, i.e. enlarging the decision space, which enhances flexibility and creates adaptiveness to handle “*currently unknown future changes*” (Zandvoort et al.2018, p.191).  The DP has used ADM to devise *robust* *flexible strategies*. Flexibility is needed to deal with uncertain future conditions and change, and, at the same time, make responsible investments. **Flexibility can be delivered in several ways, e.g.: by designing each strategy as an *adaptation path* (pathway), by using solutions that are ‘flexible’ *per se*, by using various pathways, by linking agendas, etc. These types of flexibility create added value in terms of cost reduction, feasibility, and benefits (DP 2014, p.90, 139).**  **An adaptive plan / policy is considered robust if the desired objectives can be achieved under a variety of circumstances (i.e. scenarios), whereas flexibility is related with the easiness with which a plan can be adapted to changing conditions and the capacity to adapt to a multitude of possible future conditions and changes. The flexibility of a plan is enhanced if its measures can be adapted, implemented earlier or later than planned and meanwhile the plan’s goal will still be reached (Deltares, p.2-3), if it is possible to shift from one measure or pathway to another.**  Flexibility (capacity to be flexible) may be *flexibility* to switch to other strategies when needed, in the face of uncertain future changes, it has elements of experimentation and learning. Adaptive capacity refers to capacities, attributes, and resources to undertake adaptation; it concerns the ability to anticipate, innovate and generate experience in dealing with change and learn from mistakes (Haegen and Wieriks 2015).   - In ADM, a strategy should also be flexible in terms of timing, i.e. allow the possibility to postpone or advance a measure in time (Zandvoort et al. 2018, p.191). This means flexibility to alter the timing for implementing a measure (Alphen 2013, *in* Zevenbergen et al. 2018). ADM seeks to *take the right measures at the right moment* (when they are really needed to achieve the desired objectives). Adaptiveness and flexibility are related with the possibility to modify a decision about the timing for a measure (Zandvoort et al. 2018, p.191). - In ADM, adaptiveness is also related with *keeping options open*, i.e. enlarging the decision space, which enhances flexibility and creates adaptiveness to handle *currently unknown future changes* (Zandvoort et al.2018, p.191). ‘Keeping options open’ means having possibilities of altering the course. It implies keeping the possibility to switch to other measures, and a stepwise implementation of various measures – both contribute to increase flexibility (*dynamic robustness*) (Jeuken et al. 2014; Alphen 2015). It implies finding measures that do not block further measures that may be necessary in the future (Jeuken and Reeder 2011, p.2). The DP specified measures needed to keep options open for the future, e.g. ‘spatial reservations’ required to keep ‘river widening’ measures open for a long time (Jeuken et al. 2014, p.18). - Flexibility is also related with avoiding lock-ins and lock-outs (Zandvoort et al. 2018; Alphen 2015, p.310). ADM seeks to avoid lock-ins. In ADM, planners make short-term decisions and interventions taking into consideration their possible long-term effects on the system (Zandvoort et al. 2018, p.191, 194, 183). To avoid lock-ins, the DP identified spatial adaptation measures in addition to the traditional prevention measures (Restemeyer et al.2017). - A flexible approach (that allows the switching between strategies) requires ongoing monitoring and evaluation. ADM implies clear goals with corresponding indicators and thresholds to evaluate the performance of system’s components. Flexibility also depends on the ‘*capacity (…) to learn from the arrival of new information and (…) ability to revise investment decisions based upon that learning’* (Alphen 2013, *in* Zevenbergen et al. 2018). - To operationalize ADM, the DP sought to interlink diverse investment agendas and seize opportunities to mainstream FRM measures into other investments (e.g. in urban development, nature restoration). ADM calls for *adaptive integrated* strategies that logically combine investment decisions in areas marked by uncertainty and multi-scalar complexity*.* By merging various investment agendas and mainstreaming adaptation into planned investments, ADM generates added value (Zevenbergen et al. 2018; Alphen 2013). The combination of proposed measures with other agendas and multi-objective investments increases flexibility and reduces costs (Jeuken et al. 2014, p.1, 19; DP 2011, p.48; Jeuken and Reeder 2011; Alphen 2015). The DP sought to ensure an *integrated* delivery of policies (to enhance legitimacy and feasibility) (Zevenbergen et al. 2018). The DP indicates which and how measures can be connected to other investments and agendas in other fields (e.g. infrastructure, industry, etc.) (DP 2017, p.22). Flexibility implies seizing opportunities to couple or integrate the proposed FRM measures with other investments or agendas; ADM also explains how the Strategies should be embedded into existing institutional structures (Zandvoort et al. 2018, p.191,189, 194). |
|  |  | The DP shows signs of an ‘*integrated and* *adaptive approach’* that seeks to deal with uncertain future change, and, on the other hand, it still shows traces of a more ‘predict-and-control’ approach that subsists in the Dutch FRM culture resultant from centuries of ‘*more* *technocratic-inspired policies’* (Restemeyer et al. 2017, p.920, 935). ADM reflects the particular way in which the Dutch interpret ‘adaptability’: ‘*with a strong reliance on governments, experts, techniques and tools’*, and based on their past. Continuing with the traditional practice may be advantageous (continuing something that they are good at) or disadvantageous (being tied to old patterns or dismiss the need to increase adaptability on land) (Restemeyer et al. 2017). |

**Table B.** Important concepts of the Adaptive Planning and Management approaches, and their conceptualization and understanding in each of the selected cases. Source: Own elaboration based on several authors, Reeder and Ranger 2011; Ranger et al. 2013; HM Treasury 2009; Bloemen et al. 2018; Lowe et al. 2009; Ramsbottom and Sheppard 2017; Environment Agency 2009a; DP 2014, 2013, 2012, 2011, 2010, 2016; 2017; Klijn et al. 2015; Deltares; Gersonius et al. 2016; Zandvoort et al. 2018, etc.

##### C. Positive outcomes and advantages of using APs-based AP&M approaches in the studied cases

| **Table C. Positive outcomes and advantages of using APs-based AP&M approaches in the studied cases** | **Cases** | **Relation w/ key-elements** |
| --- | --- | --- |
| Improving the awareness and the management of uncertainties about future changes and about (new) dynamics of climate change, and inter-temporal complexities, at the local scale. By showing how long-term objectives and tasks can be related to short-term decisions and investments, the APs stimulates policymakers to ‘*incorporate*’ uncertainties about future changes in their plans (Bloemen et al. 2018).  Provided a way to understand inter-temporal complexities and uncertainties related to the (new) dynamics of climate change (Bloemen et al. 2018, p.3).  **APs approach can significantly enhance the pace and quality of decision-making in situations marked by high uncertainty about future changes** (Bloemen et al. 2018, p.21). | TE2100; DP2014 | K1 |
| Experiences in the two cases demonstrated that the **APs approach was valuable and successful** in moving adaptation decision-making process forward, leading to the approval of a long-term plan, and its follow-up. It also helped to raise awareness about uncertainties, to visualize multiple alternatives (i.e. alternative measures and pathways), garnered political support for maintaining long-term options open, and encouraged decision-makers to adjust their plans to better address future and evolving conditions. By clearly showing how short-term decisions and actions are connected to long-term objectives, it motivated and supported politicians, policymakers and other actors, to factor in uncertainties about future conditions and changes into their decisions and plans (Bloemen et al. 2018, p.11).  Adaptation pathways are effective in guiding and engaging decision-makers (Bloemen et al. 2018, p.21) | TE2100; DP2014 | K5; AP&M |
| Both cases demonstrate that long-term adaptation pathways planning is particularly valuable when applied at a strategic level of decision-making. It is important to promote a “free thinking space” that allows for considering measures that may not be politically or financially feasible in the short-term. APs approaches can help foster the development and evaluation of innovative solutions (Bloemen et al. 2018, p.11). | TE2100; DP2014 | K3 |
| Providing a way of visualizing multiple alternatives. The APs served to position measures in time (a *timeframe*) and in the physical space. It allowed the development of a plan / strategies that can be adjusted to changes (Bloemen et al. 2018). | TE2100; DP2014 | K3 |
| Though it was not easy to build consensus on *which* and *how* measures should be implemented, **having a ‘maquette’ of adaptation pathways early in the planning process** is useful for communicating concepts and crucial to get stakeholders’ support and buy-in of the Plan / Strategy (Bloemen et al. 2018). | TE2100; DP2014 | K3 |
| **Enhancing and fostering political commitment to keeping options open for the future**. The method stimulated decision-makers to include in their plans alternative measures (options) to adapt to uncertain and changing conditions over time. The APs served to explore ‘options to keep open for the long-term’, e.g. the TE2100 demarcated land areas for future flood defences, and the DP set the reservation of areas for possible future options (e.g. retention area in the Rhine) (Bloemen et al. 2018). | TE2100; DP2014 | K3; its sub-elements |
| Moving the adaptation planning and decision-making process forward up to the approval of the long-term plan, improving the pace and quality of decision-making processes under uncertain future changes, but also to guide the implementation of this plan, and its adaptation / regular adjustment over time (Bloemen et al. 2018, p.2). | TE2100; DP2014 | K5 |
| Articulate a ‘combination of objectives’ of FRM, FS, and spatial adaptation, and coordination articulation between the national and regional scale (Jeuken and Reeder 2011). | DP2014 | K5 |
| By using the Route-map / APs approach, it is possible to make decisions more ‘robust’ under deeply uncertain future conditions and changes (namely decisions involving high-stakes) (Reeder and Ranger 2011, p.2, 4; Jeuken and Reeder 2011, p.5). APs approach constitutes a way of incorporating (building-in) and designing ***dynamic robustness*** (*flexibility*) into the adaptation plan itself and its strategies (pathways), as a way of dealing with deep uncertainties about future (climatic and socioeconomic) conditions and changes (Reeder and Ranger 2011). Instead of making a potentially irreversible decision now about the ‘best’ measure(s) to manage future risk based on a given scenario (which could lead to **maladaptation** if the expected scenario did not occur), the approach encouraged a more flexible stance regarding climate adaptation (Reeder and Ranger 2011; Ranger et al. 2013), since “*decisions are made over time to continuously adapt while maintaining as much flexibility* *as is desirable about future options”* (Reeder and Ranger 2011, p.8).  The APs approach helped to ensure that whatever short- / mid-term measure is taken, it is set within a framework (pathway) that will not result in maladaptation if climate change progresses at a different rate from what was predicted as ‘the most probable future’ when the Plan was designed (Reeder and Ranger 2011, p.8; Ranger et al. 2013, p.255). It promotes an adaptive approach regarding climate adaptation that is more cost-effective and avoids *maladaptations* since the outset (Reeder and Ranger 2011, p.13; Jeuken and Reeder 2011, p.4). | TE2100 | K3, flexibility / dynamic robustness |
| The Route-map approach provided a way forward to tackle decision-making in the face of incomplete or ‘imperfect knowledge’ – i.e. deep uncertainty about future conditions and changes (Reeder and Ranger 2011, p.13). The approach for developing the Plan explicitly addressed uncertainties around the projection of future climate, physical and socioeconomic changes in the TE (Lowe et al. 2009, p.86). The adaptive planning approach of the TE2100 is deemed a sensible way of moving forward in face of uncertainties about future conditions and changing risk, and it can cope with a ‘*large range of change*’ if needed (Lowe et al. 2009, p. 85). | TE2100 | AP&M approach; K3; K1 |
| APs approach provided clear information on the timing and effectiveness of measures, by assessing under what conditions a measure could fail, and on what timescale (Ranger et al. 2013, p.255). | TE2100 | K2 |
| The Route-map approach reduces the dependence of decisions on any climate scenario, therefore, stimulates a *more robust planning* (Reeder and Ranger 2011; Jeuken and Reeder 2011). The approach, and the resulting plan, are more ‘*scenario neutral*’: decisions are not so dependent on information about the likelihood of different climate scenarios (Reeder and Ranger 2011, p.9; Ranger et al. 2013, p.254). | TE2100 | K1 |
| In APs approach, adaptation over time is not only determined by what is anticipated now, but also by what will be observed and learnt as time unfolds (Ranger et al. 2013, p.255). | TE2100 | K4 |
| APs approach helps in identifying low-regret measures and opportunities, but also potential lock-ins (Ranger et al. 2013, p.255). | TE2100;  DP2014 | K3 sub-elements |
| The ‘decision-centred’ planning process, including the Route-map approach, did not take much time nor intensive studies, it helped to get an idea of the adaptation needs and types of measures and options that are relevant, and identify information gaps; and the exercise can be repeated several times, with growing detail (Reeder and Ranger 2011). | TE2100 | K5 |
| Clear communication of Project outcomes, e.g. the draft and final Plan, the list of actions needed to undertake it, information on the works needed, defense crest levels, etc.) (Ramsbottom and Sheppard 2017). | TE2100 | K5 |
| Involvement of stakeholders from the TE and London was crucial for the success of the TE2100, especially to ensure that the final Plan was compatible (as far as possible) with different interests concentrated in the TE. The ongoing dialogue with a broad range of actors and sectors, alongside with the large study-program undertaken, allowed a deeper understanding of issues that were critical to develop and assess the pathways (Lowe et al. 2009, p.87). | TE2100 | K5 |
| By specifying the physical thresholds that require a transition of measure, the ATP method is less sensitive to updates of scenarios that commonly emerge every few years; if new scenarios arise, it is only necessary to compare the threshold values against such scenarios (Klijn et al. 2015). | DP2014 | K2 |
| Working with APs facilitated making choices in areas where measures were already needed in the short-term (Rhee 2012, *in* Marchand and Ludwig 2014, p.27). | DP2014 | K3 |
| Using APs, it was possible to synchronize decisions in time, and find optimized implementation sequences (Rhee 2012, *in* Marchand and Ludwig 2014, p.27). | DP2014 | K3 |
| It was possible to link measures with other investment agendas, which stimulated win-win situations (Rhee 2012, *in* Marchand and Ludwig 2014, p.27). ADM offered a valuable means for framing short-term decisions in the context of long-term objectives and tasks and identifying possibilities for linking measures with other planned investments or merging different investment agendas (Gersonius et al. 2016 p.13-14). ADM is an adaptive but also integrated / comprehensive approach that aims to combine various investment agendas and seize opportunities to mainstream FRM into planned investments (Haegen and Wieriks 2015). | DP2014 | ADM |
| The APs method was essential to develop strategies that can be adjusted to changing external conditions and to position measures in a timeframe (Bloemen et al. 2018). | DP2014 | K3; adaptabili. |
| The APs contributed to embed long-term objectives into decisions in the near future in several sectors (urban planning, water management, nature, infrastructure, shipping) (Bloemen et al. 2018). | DP2014 | K3 |
| APs contributed to increase the awareness of uncertainties (Bloemen et al. 2018). ADM renders uncertainties about future tasks more manageable (DP 2014, p.144) | DP2014 | ADM + APs |
| Positive outcomes from using this approach have led to its wider adoption (Bloemen et al. 2018, p.21). The adoption of a flexible APs-based approach for planning climate adaptation was crucial in securing approval and support for the plan from key actors (e.g. in London and the wider South East; and in the stakeholders involved in the six regional sub-programs of the DP) (Bloemen et al. 2018, p.11).  The principles of an adaptive approach to planning for climate adaptation have been incorporated into the guidance for FRM provided by both the UK’s Environment Agency and Lead Local Flood Authorities (Kuklicke and Demeritt 2016), as well as in the annual DP reports submitted to parliament (DP 2015, 2015, 2016) (Bloemen et al. 2018, p.12).  The value and potential applicability of the TE2100’s approach have gained recognition both in the UK and internationally. It has been considered and applied to stimulate discussions in several initiatives, e.g. **Coast Communities 2150 project**, which developed a long-term adaptation plan for Newhaven on the South Coast of the UK; the London Climate Change Partnership (which supports the use of APs approaches); and the London Assembly (which urged the mayor to prepare adaptation options as ‘pathways’, if possible) (London Environment Committee (LEC) 2015) (Bloemen et al. 2018, p.12). | TE2100  DP2014 | AP&M APs |

##### D. Difficulties and barriers in applying an APs-based approach

| **Table D. Difficulties and barriers in applying an APs-based approach** | **Cases** | **Relation w/ key-elements** |
| --- | --- | --- |
| The use of a broad range of scenarios is more acceptable in the exploratory phase of the process (Jeuken et al. 2014, p.1). | TE2100 | K1 |
| The identification of thresholds for some systems can be problematic or difficult for some systems (e.g. ecosystems) or if there is insufficient knowledge. In the TE2100, it was difficult to determine thresholds for impacts on local ecosystems. Even though, it is possible to advance, by gradually postulating such thresholds and then subjecting them to more detailed modelling. If a threshold is potentially critical, it can be further researched and monitored, so that the route-map can be adjusted (e.g. a threshold is postulated and subjected to sensitivity analyses). Provided that the APs approach accounts for *potential surprises* and ongoing learning, it allows for adjustments to be planned in (Reeder and Ranger 2011; Ranger et al. 2013). | TE2100 | K2 |
| In the ‘formal appraisal’ of pathways, a potential drawback is that the decision analysis methods used in it are often resource intensive. However, when high stakes are involved, a careful assessment (considering vulnerabilities and uncertainties) is usually justified (Reeder and Ranger 2011).  During the public consultations, many stakeholders felt that it was difficult to comment on measures for the late century and easier to plan decisions for the near- and mid-term (EA 2012, p.9). | TE2100 | K3 |
| Some structural flood protection measures taken in the past do constrain future options and choices. In part, the flexibility of the Plan (its pathways) was constrained by the nature and lifespan of some pre-existent measures (e.g. barriers that are in place for many decades) (Jeuken et al. 2014). | TE2100 | K3 |
| A disadvantage of the Route-map approach (as other approaches that seek to build in flexibility into the adaptive plan) is related to its greater overall costs. For instance, delaying a measure (e.g. the construction of a defence) can leave people temporarily exposed to storm surges, or imply expensive repairs to existing defences (as in the TE2100 case). Such trade-offs should be carefully assessed in during the Options’ appraisal. In addition, the APs often requires detailed information about the local system (the FRM system, ecosystems), namely for identifying thresholds (Reeder and Ranger 2011). | TE2100 | K3 |
| The monitoring and review (e.g. the reappraisal of Options) are complicated by the fact that the trends in some variables cannot be easily detected (Jeuken et al. 2014, p.1). The TE2100 case suggests that it is necessary to shift from probabilistic modelling to a greater focus on monitoring and observation, on an improved understanding of historical climatic variability, physical systems and processes, their representation in models, in order to improve projections/ scenarios (Ranger et al. 2013, p.234). | TE2100 | K4 |
| Difficulties in ensuring that land will be available for creating new intertidal habitat areas. It has been difficult to obtain land for this purpose; in some areas this will require managed realignment (breaching and realigning existing flood defences). Agreement on the availability of land is needed (Ramsbottom and Sheppard 2017, p.17). | TE2100 | K3 sub-elements |
| The scarce ‘integration’ of studies about different sources of flooding: tidal and fluvial flood risk have been examined in detail in the TE2100; while studies focused on surface water and groundwater. However, their results have not been fully ‘joined up’. The TE2100 Plan has not been linked with drainage plans for the defended areas (Ramsbottom and Sheppard 2017, p.17). | TE2100 | K1 + K2 |
| **Difficulty in working with a wide range of scenarios.** The use of a broad range of scenarios was, in general, more viable in the exploratory phase of the planning process (as in the TE2100 case) (Jeuken et al. 2014, p.1). The DP worked with a moderate range of climate scenarios that does not include high-end projections. In the beginning of the DP, the consideration of an ‘high-end scenario for SRL’ (of 1,3m) generated scientific and public controversy, thus, it was abandoned. The scientific willingness to communicate uncertainties may not always be compatible with their political recognition (Jeuken et al. 2014, p.11). In the RE-D Subprogramme, experience showed that working with scenarios was easier in theory than in practice: it was too difficult for people to think about four possible futures, they tended to focus on the ‘steam’ and ‘rest’ scenarios (Restemeyer et al. 2017, p.931). The idea that ‘whatever scenario happens, the existing defence system can cope with it and it will not require radical modifications’ hampered the identification of tipping-points and visualization of pathways (Restemeyer et al. 2017, p.931).  The Delta Scenarios disregard the institutional and governance aspects, e.g. what may be the role of the state, market and society under different scenarios (Restemeyer et al.2017, p.932; Brugge and Bruggeman). | DP2014 | K1 |
| **Difficulty in and complexity of identifying ATPs**. The identification of ATPs was deemed one of the most challenging aspects of ADM by the DP staff (Gersonius et al. 2016). Some hindrances that hampered the identification of ATPs (in the DP) were:   - ATPs are difficult to identify where the signals of climate change are weak in relation to the natural variability of the system, as in river discharges. - It is not straightforward to determine threshold values in the absence of binding safety norms, which happens in unembanked areas. - ATPs are difficult to identify in the case of strategies that are inherently flexible, e.g. sand nourishment (which can be extended indefinitely). - the ATP works for slowly changing conditions, but for drivers affected by extreme events / sudden changes, it runs the risk of being too late (Gersonius et al. 2016; Zevenbergen et al. 2018; Haasnoot et al. 2013).   **Difficulty in determining tipping-points** **in situations where: a) there is large natural variability, b) intrinsically flexible strategies are used, c) there are no clear policy goals defined** (Bloemen et al. 2018). In the APs, it is implicitly assumed that some physical parameters that influence flood risk (e.g. climatic conditions that influence the probability of floods, or socioeconomic developments that influence the consequences of a flood) change gradually and allow societal systems to react and shift to a new measure. This works well in situations characterized by gradual trends / slow changes, e.g. SLR, however, evidence shows that, in situations of large natural variability, the determination of tipping-points is challenging. For instance, there is usually a great difficulty in monitoring changes in the patterns of storms, droughts, etc., in conjunction with a lack of observations of extreme events. The detection of climate change-induced changes in certain parameters, e.g. in river discharges, requires detailed monitoring data and model calculations and often shows that the natural variability is so high that it may take 3 or 4 decades until CC signals can be distinguished accurately. Further research is needed to discern CC signals from ‘common’ monitoring measurements, through approaches that use observations and future projections, scenarios and modelling; there is a need of methods to distinguish climate signals from highly variable measurements of physical conditions (Bloemen et al. 2018, p.14).  It is also difficult to determine ATPs where there are not precise goals, and when strategies are inherently flexible. The DP’s strategy to manage coastal flood risk is sand nourishment, which is flexible: the sand volume supplied per year can be increased / decreased according to the observed SLR rate (Bloemen et al. 2018, p.14). | DP2014 | K2 |
| While some variables / indicators can be easily monitored (e.g. SLR), others present more challenges, e.g. river discharge. River discharge shows a large natural variability (it can increase or decrease during a period without a pattern). In the DP, it was nearly impossible to distinguish climate change signals from natural variations in river discharge. Hence, the DP decided to assume a fixed value of river discharge (maximum value): 17 000 m^3^/s in 2050 and 18000 m^3^/s in 2100. However, as noted by the DP Staff member responsible for ADM, taking decisions and measures based on ‘artificially-fixed’ worst future conditions (in cases where one cannot rely on monitoring) goes against the idea of ‘flexibility’. Setting a fixed value resembles more a ‘predict-and-control’ approach than an adaptive approach (Restemeyer et al. 2017, p.931) | DP2014 | K2 + K4 |
| **Complexity of the ATPs and APs methods *per se*.** The ATP and APs methods may become quite complex and abstract for strategic policymaking at a national or regional scale (Restemeyer et al. 2017, p.936). In the DP, ADM was time-consuming and required detailed knowledge of the FRM system (Gersonius et al. 2016). For example, the Subprogramme for the RE-D faced several difficulties in working with Scenarios, ATPs, and APs. In practice, it was quite complex to identify tipping-points and envision adaptation pathways (Restemeyer et al. 2017, p.931). Both ATPs and APs methods assume that tipping-points can be identified beforehand (or, at least, when they are reached), however, practice shows that there are limits to the forecasting capacity and it is quite difficult to define adequate monitoring parameters, namely in cases of large natural variability (Restemeyer et al. 2017, p.931). While some indicators can be easily monitored (e.g. SLR), others present more challenges, e.g. river discharge. River discharge shows a large natural variability (it can increase or decrease during a period without a pattern). In the DP, it was nearly impossible to distinguish climate change signals from natural variations in river discharge. Hence, the DP decided to assume a fixed value of river discharge (maximum value): 17 000 m^3^/s in 2050 and 18000 m^3^/s in 2100. However, as noted by the DP Staff member responsible for ADM, taking decisions and measures based on ‘artificially-fixed’ worst future conditions (in cases where one cannot rely on monitoring) goes against the idea of ‘flexibility’. Setting a fixed value resembles more a ‘predict-and-control’ approach than an adaptive approach (Restemeyer et al. 2017, p.931). In the RE-D Subprogramme, ATPs were difficult to find or did not exist, thus, the exercise was more about ‘*spreading measures in time*’ than ‘*exploring alternative measures to shift to*’ (Restemeyer et al. 2017, p.932).  APs method can be complex and abstract for policymaking at national and regional scales; it is more feasible at a local scale, where ‘*specific measures can be discussed in depth and better embedded into the physical, social and political reality*’ (Restemeyer etal.2017, p. 936). | DP2014 | K2 + K3 |
| **Gaps in the assessment of measures and paths** (strategies): the lack of criteria specific of climate adaptation, namely *flexibility* and *robustness*; measures and paths were mainly assessed quantitatively (time and costs) in detriment of qualitative aspects (time and costs largely influenced the selection of measures), some strategies were left out at an early stage due to political reasons (rather than formal appraisal criteria); in the strategy-making and in the assessment, the identification of measures could have been broader and foster consensus (consensus-building was not explicitly addressed in the appraisal of measures) (Werners et al. 2016). | DP2014 | K3 |
| **Difficulty in quantifying / valuating flexibility in measures.** Another aspect of the ADM approach that was difficult to apply in the DP was the *‘quantification of the added-value of flexibility’* (‘Real Options Analysis’ was too complex in many cases) (Bloemen et al. 2018, p.14). Regarding the assessment of pathways, traditional cost-benefit analyses present shortcomings, e.g. the non-consideration of uncertainties about the future, and the non-valuation of the added value of flexibility, which can lead to suboptimal decisions. Several authors have investigated methods to valuate flexibility economically, based on ‘Real Options Analysis’ (Brugge and Bruggeman, p.5) | DP2014 | K3 |
| **Difficulty in untangling (grasping) relationships between measures that are implemented in parallel, i.e. simultaneously in time (at different areas).** A difficulty presented by the APs approach lies in understanding and disentangling interdependences between measures that are defined for the same time period, but in different zones or in different fields. As seen, in the DP’s Preferential Strategies, several ‘*parallel trajectories*’ were often drawn, to be implemented at the same time. These different trajectories often concern different actors, hence, the interrelatedness of their outcomes tends to be disregarded and their successful implementation becomes more uncertain. In addition, while the assessment of the effectiveness of individual measures is a complex issue *per se*, the use of simultaneous measures in ‘parallel trajectories’ further complicates it. Bloemen et al. suggest that the effectiveness of the general Strategy could be examined by evaluating (ex-ante) how the different parallel (concurrent) ‘trajectories of action’ strengthen/ weaken each other, and by recombining or eliminating trajectories (measures). The DP shows that, in the preparation of a strategy that contains several *parallel trajectories* (i.e. parallel / complementary measures), it would be useful to assess if such trajectories perform well under diverse conditions, and examine if the general Strategy covers a wide spectrum of plausible futures evenly, or if it is skewed towards a specific scenario and compensation is needed (Bloemen et al. 2018, p.14-15).  It was difficult to grasp interdependences of measures in different policy-fields and in different parts of catchment areas, and to coordinate investment agendas of other actors (Bloemen et al. 2018, p.14). | DP2014 | K3 |
| **Difficulty in maintaining commitment to prepare and decide on possible options for the long-term**. APs maps define measures to be taken in the short-term and indicate possible future measures for the long-term for which decisions will need to be taken in due time. However, decisions on such options and their implementation are often postponed until the (climatic, physical, socioeconomic) conditions that justify them are reached or predicted with greater certainty. This hinders the adequate anticipation of these measures. The postponing of decisions about future options is usually related to **avoiding the ‘unwanted anticipation’ of long-term measures** (Bloemen et al. 2018, p.15). On the other hand, taking a decision now to do not implement a certain future measure can lead to a search for other measures. Thus, postponing decisions about future options can be advantageous or disadvantageous depending on the nature of the measure, costs and benefits of anticipating it for diverse actors, the direction that the anticipation follows in relation to the objectives of the measure, etc. These aspects must be considered when taking decisions about, and planning, future measures. | TE2100+  DP2014 | K3; sub-elements of K3 |
| **Political resistance to ‘keep options open’, and to take preparatory measures needed for keeping options open**, was found in several Subprogrammes of the DP (Gersonius et al. 2016 p.13). While ‘keeping options open’ is logical in theory, it is often difficult for decision-makers to commit to this. The reflection on future options can lead to greater cost-effectiveness in the long-term, but the traditional focus of decision-makers on the status quo, and their risk-aversion (and uncertainty-avoidance), tend to reduce their willpower to keep options open. These are barriers to the implementation of adaptive approaches (Gersonius et al. 2016). This can be explained by: (a) lack of clearness on whether options are realistic or will ever be realised; (b) keeping options open usually entails ‘spatial claims’ that put constraints on spatial developments; (c) decision-makers’ desire and habit to take visible short-term actions (rather than inaction that facilitates future action) (Gersonius et al. 2016 p.13). *Keeping options open*, and using measures that allow their own adjustment over time, may also entail higher costs (DP 2011, p.49). | DP2014 | K3; sub-elements of K3 |
| **Difficulty of preparing and making shifts from incremental to transformational measures**. Both the TE2100 and the DP sought to secure the possibility to shift from incremental to transformational measures, however, this point could be further improved and contribute to improve the APs approach, by clarifying how such shifts can be enabled. In both cases, often, the selected pathway contains incremental measures in the short-term, and ‘system-changing’ transformational measures in the long-term. Incremental measures usually correspond to investments in further / gradual improvement of the protection level provided by the current system. Yet, continuing in a path of incremental measures can lead to an increase or a transference of costs to a future system, greater path-dependency and sunk-costs (by augmenting the point for switching from incremental to transformational measures) (Bloemen et al. 2018, p.17). | TE2100+  DP2014 | K3 |
| **The flexibility of the Strategies may be constrained** by pre-existing structural flood protection measures. Some structural flood protection measures taken in the past do constrain future options and choices (Jeuken et al. 2014, p.1). In the DP, the flexibility of strategies was often limited by the nature and lifespan of some measures – e.g. protection measures involving barriers that are implemented for several decades (Jeuken et al. 2014, p.18). It is quite difficult to prioritise measures that prevent lock-ins when such system *is already caught in a lock-in* (Restemeyer et al. 2017, p.936). | DP2014 | K3; overall plan’s flexibility |
| **Some spatial developments or investments hamper or lock out future options.** In some situations, spatial developments contributed to narrow down future options, e.g. past investments in hard defences in the Netherlands have attracted economic activity to low-lying flood-prone areas (and this development usually does not account for long-term adaptation needs), this will hamper a switch to measures substantially different from hard defences. In the future, measures involving spatial planning instruments that shape development patterns (e.g. risk-zoning plans, spatial reservations, building codes) will become essential to reduce flood risk (Jeuken et al. 2014, p.21). | DP2014 | K3; K3 sub-elements |
| **Difficulties in the monitoring and reassessment.** Monitoring and the reassessment of pathways are complicated by the fact that trends in some variables cannot be detected (Jeuken et al. 2014, p.1). For Zevenbergen et al., the detection of climate change effects within the timescales of decision-making remains difficult, and it still needs to be seen whether the adaptive approach is being effectively applied in practice.  The potential for monitoring and reassessment of options is hampered by the fact that trends in some variables cannot be detected’ (Jeuken et al. 2014, p.1). In situations **where: there is large natural variability, intrinsically flexible strategies are used, or there are no clear policy goals defined**), the monitoring and detection, in a timely way, of tipping-points, although challenging, will be essential. This implies the development of a monitoring and evaluation system able to detect tipping-points in such situations (Bloemen et al. 2018). | TE2100+  DP2014 | K4 |
| **Scarce public engagement.** The involvement of many stakeholders in a joint decision-making process was not easy. It was difficult to foster the engagement of citizens in decisions (unless decisions strongly affected in their ‘backyards’), and public awareness of flood risk in the country is low (Haegen and Wieriks 2015; Ritzema and Steensma 2018, p.7). The DP was quite expert- and government-driven, with strong reliance on expert knowledge from water domain and scarce engagement of civil society (citizens and business had not a formal role in the strategy-making process) (Restemeyer et al. 2017, p.929; Werners et al. 2016; Seijger et al. 2017) | DP2014 | K5 |
| **Difficulties in the implementation phase**. The success of ADM and the effectiveness of proposed pathways can only be measured as the future unfolds (Gersonius et a. 2016). ADM also implies an ‘*adaptive implementation*’ of the measures proposed in the Strategies (DP 2014, p.145). Yet, a recent survey showed that some actors responsible for the implementation of Strategies in their regions are concerned about their ability to maintain, in the implementation phase, the adaptive approach and to deal with unexpected conditions as time unfolds (Bloemen et al. 2018). It is also important to ensure that measures are enabled by legislation, however, at European level there several directives aimed at ensuring ‘preservation’ which can hamper an adaptive approach; thus the DP has made efforts to tackle such hindrances (DP 2011, p.54). | DP2014 | K5 |
| **Different conceptualizations of flood risk and of the FRM challenge****.** According to Klijn et al., in the Netherlands, defence engineers and spatial planners have commonly relied on different conceptualizations of flood risk and of the FRM challenge, and thus, on different measures; i.e. they use different framings of risk and how it can be tackled (Klijn et al. 2015, p.860). According to Klijn et al. (2015), there are two common definitions of flood risk in the Netherlands: 1) Flood risk = probability of flooding x consequences of flooding; 2) Flood risk = flood hazard x vulnerability of society / an area.  The 1^st^ definition is usually preferred by engineers and natural scientists, who seek to reduce the probability of flooding via flood protection measures (e.g. engineers of Rijkswaterstaat); it was quite aligned with the country’s situation in which 3000 km of defences protect flood-prone areas). The 2^nd^ definition is preferred by spatial planners, who assume the ‘hazard’ as a given and spatial planning-related measures as a means to adapt to that given. In this definition, the ‘hazard’ covers all flood characteristics (flood probability, extent, depth, etc). The underlying thought is ‘without people, no risk’, thus, it uses other measures than defence measures only (Klijn et al. 2015, p.850).  These two definitions of flood risk are present in the DP Subprogrammes, however, the most common tends to be the first one (it is subjacent to the definition of the new protection standards and the Multi-layered FRM concept). Nevertheless, in both conceptualizations, ‘exposure’ (which refers to flood characteristics like flood depth, extent, water inflow rate, trajectory, volume) is disregarded: defence engineers see ‘exposure’ as part of the ‘consequences’ and not their responsibility, while spatial planners see ‘exposure’ as part of the ‘hazard’ (not their responsibility). Hence, no one can be held responsible for influencing exposure determinants (Klijn et al. 2015). A limited understanding of dynamic interactions among diverse components of risk may hinder an effective FRM. Moreover, there is no information to discern if a decrease in risk that results from reducing the probability of flooding (with defences) is nullified by an increase in the consequences associated with a growing population and investments in floodable zones (Bloemen et al. 2018). | DP2014 | K5 |
| **Path-dependencies****: mechanisms of path-dependency and ‘*policy stability or stickiness*’**. The term ‘path-dependency’ is useful to explain the stability of a flood policy, as noted by Buuren et al. (2016). Several authors have used it to understand ‘*why some policies are resistant to change’* and ‘*why some new policies are more of the same’* (Buuren et al. 2016). There are still path-dependencies rooted in the Dutch flood policy. Until the 1990s, the Dutch FRM approach has been dominated by flood defences to protect the country from floods. Some authors denominate this continuity as ‘institutional inertia’ or ‘regime stability’ and explain it with the existence of ‘path-dependency’ (dependence on the past path of investment in defence structures). More recently, there has been a shift from an ‘exclusive reliance on flood defences’ to a more integrated FRM. For centuries, Dutch FRM was equivalent to flood protection in the form of dykes and dams, and wetlands, marshes and intertidal areas were disregarded as ‘wasteland’. The predominant paradigm has been ‘*to drain, dredge, build dykes, and reclaim’*, and it is deeply embodied in Dutch culture. The practice of ‘draining, dredging and diking’ implied modifications and adjustments of natural landscapes, but offered little flexibility. It may take several years to take forward other solutions (Marchand and Ludwig 2014). This is a type of path-dependency. More recently, there has been a shift from an ‘exclusive reliance on flood defences’ to a more ‘integrated’ FRM, in the Netherlands and Western European countries (where several floods in the 2000’s showed the ineffectiveness of technological and institutional arrangements). The Room for the River and the Multi-layered FRM (in 2009) represented the first steps of a shift towards a more integrated approach (Buuren et al. 2016). | DP2014 | K3 + K5 |
| There are still several mechanisms of path-dependency (technical, cultural, financial or institutional) and ‘policy stickiness / stability’ in the Dutch FRM system, which hinder policy learning and change, preclude a ‘system transformation’, i.e. a shift to a more *resilient* *adaptive* FRM, and show the difficulty of changing the FRM paradigm (Buuren et al. 2016)). Path-dependencies that may constitute barriers to a true AP&M are:   - Lack of knowledge and experience in applying alternative innovative FRM measures (other than those traditionally used) namely measures that are adaptable. While the effectiveness of traditional engineering solutions can be assessed with known methods, in the case of new measures (e.g. ‘*building with nature’* measures) there are no ‘tried-and-tested’ methods yet, and it is necessary to further investigate the behaviour and protective capacity of inherently dynamic and adaptable solutions (e.g. a saltmarsh). The lack of scientific evidence on alternative measures generates uncertainty. Moreover, in coastal and deltaic systems (complex dynamic systems), it is necessary to use sophisticated (morphological, hydrodynamic and climate change) models to predict the performance of measures, and these models usually have deep uncertainty associated to them. Uncertainties regarding the future performance of new measures may easily become an excuse for not taking such options seriously (Marchand and Ludwig 2014, p.36). - Governance problems that complicate or prolong decision-making, e.g.: existence of non-integrated approaches between national, regional, and local planning levels, and strong vested interests in the engineering domain that act as counter forces and emergence of ‘hydraulic bureaucracy’ when proposing non-engineering measures. Moreover, business-as-usual measures are often sustained by, and engrained into, legal and financial institutions (these institutions often rely on a particular type of measure, which leads them, and the organizations they protect, to reject new solutions as less secure or more expensive (Marchand and Ludwig 2014, p.36). - Legacy and institutional reliance on hard protection measures (defences). For Buuren et al., there is a *path-dependency of institutions and policies, but also of the measure used* in the FRM field (hard protection)^[153]^: past choices led to a vicious cycle of investment in more advanced dyke-technology, which limits the scope for ‘softer’ forms of FRM. This shows the ‘strong stickiness’ of policy and measures employed and leads to a conflict between those favouring the ML FRM and those favouring an exclusive reliance on defences and flood risk assessments. Despite the increasing recognition of the need to increase the spatial and societal resilience, the main principle underlying Dutch FRM has been ‘*it is better to prevent than to cure*’ (*better safe, than sorry*) (Buuren et al. 2016). The Dutch have traditionally focused on reducing the ‘probability of flooding’ by reinforcing dykes; this led to a *technological lock-in*, *a vicious cycle of investment* (…) *to protect land against flooding’* (Ritzema and Steensma 2018, p.4). There is a tendency for framing new policy ideas and solutions into the models / approaches of the old paradigm. A strong epistemic community remains stick to ‘*tried and tested*’ strategies (also rooted in national mindset), which hampers policy learning and change (Buuren et al. 2016). - Difficulty in mobilizing enough impetus and resources at the right time for ML FRM. The DP created a momentum for a radical policy change, but, despite the introduction of the Multi-layered FRM concept, most efforts were directed to setting the new protection standards and defining strategies to achieve them (Buuren et al. 2016; Klijn et al. 2016). Proponents of the traditional approach exaggerated the complexity of the Multi-layered FRM, argue that the government is responsible for preventing floods (as the public expects), and emphasize the potential disastrous consequences if prevention is underestimated. Moreover, the adoption of the ML FRM approach was hindered by: administrative restrictions on the use of budgets earmarked for flood prevention (Layer 1) for spatial planning measures (Layer 2); lack of proper instruments to attribute a legal status to measures of Layers 2 and 3; and difficulty in persuading agencies about the feasibility and added value of Layers 2 and 3 (Buuren et al. 2016). Though the DP includes spatial adaptation measures, almost no budget is earmarked in the Delta Fund for spatial adaptation measures (the Delta Fund is mostly earmarked for protection); this shows a continuation of past practices that calls into question the strength of public support and the room for manoeuvre allowed in terms of moving away from previous practices (Seijger et al. 2017).   Despite these path-dependencies, the Dutch FRM system has gradually changed, as the limitations of the prior approach become salient (higher costs, increasing implementation problems) and climate change effects emerge on political agendas. Path-dependencies have not completely precluded that an adaptive approach slowly gains ground (Buuren et al. 2016). | DP2014 | K3 + K5 |
| **Difficulty in articulating the new flood protection standards with ADM.** Although the DP worked with the four Delta Scenarios, the new protection standards were defined based on the analysis of a single, relatively pessimistic, scenario for 2050 (such scenario assumes ‘*the strictest of the outcomes*’), and for some stretches, the proposed standards were even stricter due to potential societal disruption. This scenario was useful for exploring how big the problem might be, but it is quite challenging to match the new standards (based on such single scenario) with the Government’s claims of having adopted ‘*a policy of Adaptive Planning and Management*’ (Klijn et al. 2016). In 2016-2017, the new standards were adopted, and gradually, the implications of adopting them have become clearer. Due to the new standards, hundreds of km of primary defences will need to be reinforced or raised. Though the ADM principles seem straightforward, their practical application in DP’s policy for FRM was not without complications (Klijn et al. 2016):   - First, the adoption of the new flood protection standards brought problems. The prioritization of measures and defence works became very difficult (especially in the *Flood Protection programme,* HWBP). In 2015, defence reinforcements were being analysed by the order they ‘come on the list’: the first reinforcements may spend much time and money while other locations may be delayed, thus, risk might not be addressed in the most effective way in the various areas. The decision-making on dyke reinforcement projects (in 2016), were marked by multiple uncertainties about future: 1) how to balance ‘*what is optimal from a national perspective with what is practical from a local and regional perspective’*; 2) whether one should immediately meet the new standard in each site or gradually grow towards it; 3) should one invest in cost-effective probability reduction measures or keep other options open (Klijn et al. 2016). Optimization (i.e. implementing now the most cost-effective measure) may not always be the most useful answer to the problem. - Second, there are tensions between ADM and existing rules, regulations and legal procedures (e.g. the traditional engineering practice of designing protection measures that ‘last’). The adoption of the new standards raised a dilemma between ADM (and its emphasis on the possibility of shifting to other measures or strategies as conditions change over time), and the traditional engineering practice that relies on a ‘*better safe than sorry*’ approach and designs defence reinforcement works that last (i.e. the most cost-effective measure for a given scenario). ADM calls for prudency regarding the speed, timing, and amount, of measures: ‘*not too early* / *late’*, nor *too much* / *little*. Somehow, it demands a new variant of the precautionary principle, as the future effects of measures are unknown. On the other hand, the engineering practice of designing defences calls for prudency regarding performance, i.e. a ‘*better safe than sorry*’ approach, even if it is more expensive. It is common practice to build for several decades in advance (and avoid undertaking large renewals or upgrades), thus, the initial costs of defences are usually high (there is a preference for major *‘maintenance-free constructions*’ and a ‘*tendency to negligence afterwards’* (Klijn et al. 2016).^[154]^ The two approaches can conflict with each other due to environmental side-effects of hard defences, and the influence of reinforcement works on the possibility to apply and switch to other measures advocated in ADM. This raised a dilemma: if dykes were reinforced to the standards required for 2050, the rationale for certain measures (e.g. *making room for river*) is undermined (their benefit-cost is low in comparison to reinforcements); conversely, if dykes were reinforced to the old standards (pre-2017), it would be logic to take other measures to achieve the objectives in the long-term. In either case, it was crucial to avoid spending money twice or unwisely. Thus, lawfulness (working according to rules and laws) may undermine the intentions of ADM (Klijn et al. 2016). | DP2014 | K2 + ADM |
| **Dilemma between ‘adaptability’ and the ‘urge-to-control’.** The Netherlands was pioneer in the adoption of an adaptive planning approach (ADM) to an entire country, not only a region or city, but policymakers faced a dilemma between ‘*adaptability*’ and ‘*the urge-to-control*’ (Restemeyer et al. 2017), e.g. in the elaboration of the Preferential Strategy of the RE-D. The idea of adaptability was adopted, but partially. A major dilemma existed between the aspired ‘*adaptability’* and the traditional ‘*urge-to-control’*: the DP shows signs of an ‘*integrated and* *adaptive approach’* that seeks to deal with uncertain future change, and, on the other hand, it shows traces of a more ‘predict-and-control’ approach that subsists in the Dutch FRM culture resultant from centuries of ‘*more* *technocratic-inspired policies’* (Restemeyer et al. 2017, p.920, 935). ADM reflects the particular way in which the Dutch interpret ‘adaptability’: ‘*with a strong reliance on governments, experts, techniques and tools’*, and based on their past. Continuing with the traditional practice may be advantageous (continuing something that they are good at) or disadvantageous (being tied to old patterns or dismiss the need to increase adaptability on land) (Restemeyer et al. 2017) | DP2014 | ADM,  Dynamic robustness |

##### E. Gaps, needs, and issues for further research and work on APs-based AP&M approaches and their applicability for coastal climate adaptation, drawing on the two cases

Drawing on the analysis of the practical applications of an APs-based AP&M approach in the two studied cases, and the literature review conducted on this topic, the following challenges and issues for further research and work have been identified for the future advancement of this approach and to improve their applicability in real-world planning and policy-making practices and instruments.

| **Table E. Gaps, needs, and issues for further research and work on APs-based AP&M approaches and their applicability for coastal climate adaptation** | **Sources of info.** | **Relation w/ key-elements** |
| --- | --- | --- |
| *Foster ‘free thinking space’*; Create an environment that encourages open-minded thinking, allowing for the exploration of actions that may not be politically or financially feasible in the short term (Bloemen et al. 2018).  Creating a maquette of adaptation pathway diagrams early in the planning process is helpful for conveying ideas and gaining support from stakeholders (Bloemen et al. 2018). | TE2100+  DP2014 | K3 |
| Identifying tipping points in situations in which there are no clearly defined policy objectives, for inherently flexible strategies, and in contexts of high natural variability (Bloemen et al. 2018; Gersonius et al. 2013; Restemeyer et al. 2017).  Need to further improve the specification and determination, but also the detection of ATPs/critical thresholds, namely in contexts of high natural variability, where inherently flexible measures are envisioned (e.g. coastal sand nourishments). This can be facilitated by the use of local “lived values” (Barnett et al. 2014); It is also important to connect this literature to the literature on “soft” and “hard” adaptation limits (New et al. 2022) | TE2100+  DP2014 | K2 |
| Need to improve APs-based AP&M approaches to better prepare the needed shifts from incremental to transformational measures**. Need to prepare and make shifts from incremental to transformational measures**. Both the TE2100 and the DP sought to secure the possibility to shift from incremental to transformational measures, however, this point could be further improved and contribute to improve the APs approach, by clarifying how such shifts can be enabled. Further research is needed on this issue, namely to compare alternative pathways in a cost-benefit analysis that covers the entire period of the pathways, sunk and transfer costs. Under climate change, a shift to more transformational measures will be required at some point. The difficulties of making such shift in a planned way must be tackled (Bloemen et al. 2018, p.17). | TE2100;  DP2014; literature review | K3 |
| **A key challenge in the ‘APs approach’ is to detect eventual increases in path-dependency and increases in the ‘transfer costs’** (costs of switching from one measure to another, in the same pathway or in a different one). There are ‘*mechanisms of path-dependency’* that hinder shifts from incremental to more transformational measures, or block transformational pathways (Bloemen et al. 2018, p.17), but it is difficult to notice them. The necessary shifts from incremental to transformational measures must be prepared, which implies the identification and avoidance of situations where incremental measures increase path-dependency or hinder the transition to transformational measures (Bloemen et al. 2018). Often, structural flood protection measures carried out in the past do constrain future options (Jeuken et al. 2014, p.1). Moreover, certain investments or autonomous developments can hamper / lock out future measures, e.g. some past spatial developments contributed to narrow down possible options (by attracting economic activity to low lying areas). Some developments ‘*may make it difficult to switch to pathways that promote substantial different solutions*’ (Jeuken et al. 2014, p.21). It is important to build an evidence base with examples of overcoming lock-ins and of transformative and radical adaptation of the physical environment (e.g. small-scale pilots and experiments) (Restemeyer et al. 2017, p. 936). | TE2100+  DP2014 | K3 |
| Understanding the relations and interconnections between parallel measures (concurrent strategies) implemented at the same time (Bloemen et al. 2018). | DP2014 | K3 |
| Need to improve APs method to explore and assess (ex-ante and ex-post) the potential / risk of maladaptation / successful adaptation of diverse pathways (New et al. 2022) | TE2100; DP2014; lit.review | K3 |
| Need for a more integrated risk management, that addressed multiple, interacting, cascading and compounding risks (multi-risks) (New et al. 2022; Portner et al. 2022; Oppenheimer et al. 2022) | TE2100; DP2014; lit.review | K3 |
| **Little attention has been paid to the institutional and governance issues surrounding adaptation planning and decision-making, and to the political and sociocultural conditions required for the implementation of certain measures**. In adaptation realms, it is often assumed that decision-making is a more-or-less rational, scientific data-driven process, however, real-life decision-making is often marked by institutional and political considerations and power issues (Bloemen et al. 2018, p.17). Although decisions to implement a given measure or pathway are substantiated on a cost-benefit analysis of measures, political considerations can lead, for example, to decisions to implement measures with low benefit-cost (ibid, p.5). The APs approach (like other approaches that seek to rationalize decision-making in the face of uncertainty) does not explicitly address the political aspects of decision-making. Moreover, the institutional and sociocultural conditions can be critical constraining factors, especially in situations of transfer that entail larger changes. ‘Resistance to change’ in the social-technical system should not be disregarded (Bloemen et al. 2018). Certain measures or pathways will require institutional changes (as the climatic and socioeconomic scenarios that favour a given measure may not necessarily contain the sociocultural and institutional conditions required for its implementation); and governance challenges will have to be addressed ‘to keep options open for future’ (a sub-element of the APs of the DP). Hence, it is necessary to analyse the institutional and political issues that plague transformational pathways. The APs should stimulate a ‘free-thinking space’ that does not restrict measures, including measures that are not politically or financially acceptable in the short-term and innovative solutions (Bloemen et al. 2018). | TE2100; DP2014; lit.review | K5 |
| Improve the usefulness of APs approach to minimize regret and maladaptation stemming from over- / under-investment; and to provide other benefits (than adaptation), e.g. increased biodiversity, liveability, etc. (Gersonius et al. 2016). | TE2100+  DP2014 | K3 |
| “*Incorporate local information in the design of the adaptation pathways. Organize stakeholder participation in pathway development*”, and “*Organize coordination at a higher level to ensure consistency*”. i.e. Integrate local knowledge into the design of adaptation pathways and ensure active stakeholder involvement in the development process (Bloemen et al. 2018). Inclusion of different types of knowledge, including local, indigenous, scientific (expert-), and practitioner knowledges (New et al. 2022). | TE2100; DP2014; lit.review | K5 |
| Regularly updating the adaptation pathways (Bloemen et al. 2018). | TE2100+  DP2014 | K5 |
| Need to embed / incorporate AP&M approaches into local coastal contexts, and their governance, institutional specific conditions, and thus, the need of tailoring these approaches to the local contexts (Oppenheimer et al. 2019; Portner et al. 2022). | TE2100; DP2014; lit.review | AP&M approach |
| Need to develop guidance on how to apply AP&M approaches, namely in contexts marked by complex (wicked) problems, ambiguity, conflict and contentiousness around coastal adaptation issues (Wise et al. 2014; Oppenheimer et al. 2019; Bosomworth and Gaillard 2019). In designing (charting) adaptation pathways, a key priority is to reconcile divergent values, interests and preferences (Portner et al. 2022, p.94). | TE2100; DP2014; lit.review | AP&M approach |

Despite the recent efforts to transition to more adaptive planning approaches (and ongoing steps towards more adaptive forms of planning and management of coastal zones and deltas), with new cases inspired by the TE2100 and DP emerging in several coastal zones worldwide, the practical implementation of AP&M approaches requires much more effort to realize a shift to a new paradigm of “monitoring and adapting”. There are still several difficulties in applying AP&M approaches including shortcomings / gaps in the methods themselves, but also barriers to their implementation, which require further research. There is a great potential to learn more from the analysis of other cases of AP&M in coastal regions with different sociocultural and governance contexts, and thus generate a wider body of knowledge on adaptive coastal planning and management (Marchand and Ludwig 2014).

1. Ranger et al. (2010; 2013) and Reeder and Ranger (2011) provide an example of a *decision-centred process* to FRM planning, which is based on the framework developed by Willows and Connell (2003) and refined by ESPACE programme (*in* Reeder and Ranger 2011). The TE2100’s *decision-centred* *planning process*, differs from the traditional *science-first process* (Ranger et al. 2013; Reeder and Ranger 2011; Ranger et al. 2010). In a *decision-centred process*, planners start from the adaptation problem itself (need to reduce flood risk) rather than with climate projections, then define objectives and constraints, identify possible measures and assess their effectiveness under a range of plausible scenarios (Reeder and Ranger 2011). This stimulates decision-makers to focus on the decision problem and its characteristics (objectives, actors’ interests, constraints, system’s vulnerability, decision criteria) and on the solutions (measures to reduce risk) – in-depth understanding of the problem – instead of focusing on future climate projections. In the TE2100, climate projections entered in the planning process at a later stage than in a science-first approach (Ranger et al. 2013).
2. David Ramsbottom was the Environment Agency’s technical leader for the development of the TE2100.
3. Pending on research work that was being conducted, the TE2100 Team developed four interim future scenarios of plausible water level rise (Lowe et al. 2009, p.88; EA 2009b).
4. In the following sub-steps of identification of measures (conceptual analysis) and development of *HLOs*, possible measures were identified and Options were designed, based on the initial range of SLR considered (the *High++ (first guess)*) (Ranger et al. 2013, p.242). Moreover, for *sensitivity testing*, it was used ‘*an upper-bound of 4,2’* that represented a *catastrophic SLR scenario (intentionally pessimistic* and that took into account most known sources)’, and which was used for assessing *known or estimated key thresholds in between now and this upper-bound figure in terms of vulnerability to impacts, i.e. key thresholds for sensitivity in the system)* (Reeder and Ranger 2011, p.6).

   In the TE2100, CC projections entered the planning and decision-making process at a later stage: firstly, a preliminary ‘worst-case’ scenario of future water level rise was developed [*High++ (first guess)*], which was used in Step 2a, to inform the identification of possible measures, and in Step 2b, to inform the design of HLOs, creating boundaries on the route-map; subsequently, in Step 2c, a ‘central’ scenario [*Defra06*] was used in the CBA and MCDA and a set of refined scenarios was then used to explore the sensitivity of the results of the CBA and MCDA to deep uncertainties around CC; and later, these refined scenarios were also used to estimate the timing of relevant *decision-points* (Ranger et al. 2013, p.243).
5. The Early Conceptual Options study report was issued in April 2006 (EA 2009A, p.141).
6. Estuary-wide measures are, e.g.: improving existing defences, constructing a new flood barrier / barrier with locks, or convert the TB to a barrier with locks (EA 2012, p.31; HM Treasury 2009, p.23).
7. In the route-map, each box represents a *portfolio*, and sequences of *portfolios* were assembled to produce the HLOs 1-4; the SLR scenarios were introduced in the map later to determine which Options are able to deal with which scenario (HM Treasury 2009, p.23).
8. The HLOs (pathways) were developed in an iterative process, in which the measures identified were tested (to detect thresholds, through a *threshold analysis*) and used to assemble several pathways, which were progressively assessed on several criteria and refined and, subsequently, tested under different SLR scenarios (EA 2009a, p.x-xii; 2009b, p.3, 1; Lowe et al. 2009, p.86).
9. The Options consist of *alternative ways of managing flood risk on the TE*, and *alternative ways of delivering the FRM policies*; each Option is *a number of portfolios which, when implemented in sequence, provide a complete FRM solution for the next 100 years* (EA 2009A, p.138). *Each option consists of a sequence of portfolios of responses* (EA 2009A, p.150-151). *The Options are designed to achieve a range of different criteria* (namely the FRM policy set and the key-thresholds assumed) (EA 2009A, p.150-151, 95, 116). Each Option is made up of *portfolios* that are sequenced and implemented over time *(each portfolio of responses reduces flood risk so that it stays below the target level)* (EA 2009A, p.139). *The dates* (points) *at which one portfolio changes to another portfolio depend on key thresholds for FRM assumed* (EA 2009A, p.149-150). Thus, *FRM activities or interventions are sequenced through the century (the sequencing of these interventions)* (EA 2009A, p.262). *Each Option includes a sequence of interventions over the next 100 years* (EA 2009A, p.277). *Each Estuary-wide Option comprises a sequence of ‘portfolios of responses’ over the next 100 years* (EA 2009A, p.297).
10. The *Estuary-wide* *Options* were elaborated based on the HLOs, which were further refined and detailed assuming the *Defra06 scenario* (as the *most likely* scenario) (EA 2009b, p.4; Ramsbottom and Sheppard 2017, p.6, 8, 10). These *Options* were derived from (and correspond to) the HLOs developed in Step 2b, which were refined for the *Defra06 scenario* (i.e. the HLOs were further developed into generic *Estuary-wide options* (EA 2012, p.35). These *Options* are *tangible ways of delivering the assigned policies* with different costs and levels of complexity (Penning-Rowsell et al. 2013), and they also consider other objectives of the TE2100 beyond FRM, e.g.: the need to maintain the existing defence system, habitat creation requirements, links with riverside developments, etc. (Ramsbottom and Sheppard 2017, p.11). These *Options* (presented in the *Technical Report*) were derived from the HLOs and detailed assuming a 0,9m rise by 2100; under this scenario, it will not be necessary to build a new barrier, or rebuild the TB, until 2070 (Jeuken et al. 2014, p.6).
11. *In order to decide on the Plan, the Team had to understand and assess the impacts of all Options (including all Estuary-wide Options) and has used two main methods– economic appraisal and strategic environmental assessment* (EA 2012, p.40). *SEA was carried out at the same time as developing and drafting the Plan; it helped us develop and assess strategic alternatives and identify opportunities for policy amendments and environmental mitigation, while shaping our early thoughts on the content of the Plan* (EA 2009A, p.xx).

    The *Estuary-wide FRM Options* were appraised to identify which Options have the highest ratio of benefits against costs (Options Appraisal described in Chapter 8) (EA 2009A, p.186, 188-189). The appraisal period was extended up to 2169, to cover the full Options appraisal period and enable the long-term economic cost of the different Options to be analysed (EA 2009A, p.190, 202).
12. The CBA and MCA methods used in the *Formal Options Appraisal* were consistent with the EA’s *Flood and Coastal Risk Management Appraisal Guidance* (FCRM-AG) (EA 2012, p.46; Ranger et al. 2013, p.251). The appraisal was similar to a *Real Options Analysis* (Reeder and Ranger 2011).
13. The method of appraising Options implied: 1) the estimation of the **cost** of the different *Options* (including capital, maintenance and GHG costs); 2) the estimation of benefits of *Options* in terms of avoidance of flood damages to property and businesses, prevention of risk to life and losses, other economic, environmental and social benefits; 3) the estimation, in the MCA, of economic, social, environmental, and technical impacts of *Options*; 4) the comparison (weighting) of *Options* (based on costs, benefits, and impacts previously calculated), 5 ) the elaboration of recommendations for the choice of Options based on the benefits-cost ratio (Penning-Rowsell et al. 2013, p.1387, 1395; EA 2009A, P.200).

    Although the ‘value-for-money’ (cost-benefit ratio) was a central aspect to the Project, the Plan should satisfy economic, social, and environmental objectives that were addressed in the MCA and SEA (Ranger et al. 2013, p.239). The Appraisal considered monetised and non-monetised factors (Ranger et al. 2013, p.251). The TE2100 appraised the different HLOs using multiple criteria such as the *net present value* of investments (from cost-benefit analysis) and environmental impact. The Options development and appraisal process *has in practice complied closely with a “Real Options” approach, deemed a useful appraisal method in situations of systematic uncertainty* (Reeder and Ranger 2011, p.11).
14. The Options Appraisal was extended to the year 2170 *to allow for a whole-life appraisal of these “end of the century” structures. (…) This will be helpful in understanding the consequence of major decision-making during this century* (EA 2009A, p.xiii).
15. The Team ranked the *Options* (based on decision-makers’ preferences) under the *central* and the other scenarios, and identified the ‘preferred route / pathway’ under the *central* and the other scenarios (Ranger et al. 2013, p.243).
16. In sum, it is expected that a major intervention in the system will be required by 2070, and there are two ‘*front-runner*’ options: a major upgrade of the TB (within Option 1.4 / HLO1), or a new barrier at Long Reach (within Option 3.2 / HLO3a); the design and construction of such intervention should start in 2050; but these dates may change if the SLR rate differs from the projection assumed in the Plan (Ramsbottom and Sheppard 2017, p.11).

    The Plan’s strategy to manage flood risk throughout this century provides two main alternative options for the long-term future: upgrading the TB (within *Option 1.4*), or replacing it with a new barrier (within *Option 3.2*) (Ranger et al. 2013, p.237). Given the uncertainty about future changes and conditions, it would be risky to commit to a given pathway based on the current best projection. Until 2050, all the four *Options* (pathways) will be considered; a decision between them only needs to be made in the future, and, then, there will be more knowledge (monitoring and advancements in SLR modelling) (Ranger et al. 2013, p.252).

    *Assessing the ratio of benefits to costs for all of the Options considered in this stage (…) lead to our two “front runners” being determined: optimised maintenance and enhancement of the existing system with modifications made to the Thames Barrier post-2070 (Option 1.4),* and *optimised maintenance and enhancement of the existing system to 2070 and building a new barrier at Long Reach post 2070 (Option 3.2).* (EA 2009A, p.xxi). *For the period up to 2070, maintaining and enhancing the current system is strongly preferred, regardless of the “end-of century” approach selected thereafter. Uncertainty in the assessment post-2070, and the absence of an immediate need to decide on the preferred strategy beyond that point, mean that a single preferred “end of century” Option is not being promoted at this time although we present a preferred Plan which will generate a preferred option at the right time* (EA 2009A, p.xxi).

    The Plan recommendations are the following: *for the first 60 years of the Plan, the approach to FRM should be to maintain, adapt and improve the existing system including optimising repair and replacement of defences (Option 1.4). The estimated cost is £4.5bn excluding preparations for the major intervention in 2070; from 2070 the approach to FRM should be to adopt one of the two ‘front runners’ i.e. continuation of Option 1.4 or construction of a new barrier at Long Reach with associated works (Option 3.2). (…) This end of the century choice may be changed as a result of a Plan review based on changed conditions* (EA 2009A, p.262).

    The Plan contains *two preferred Estuary-wide Options*, although the choice between Options does not have to be made immediately, *because the interventions required for each of the preferred options over the next 60 years (covered by the short- and medium term investment plans) are identical. In addition, whilst the Plan includes major interventions that must be operational in 2070, the exact date when these major interventions are required will depend on the rate of climate and other change* (EA 2009A, p.267).
17. The Options Appraisal has identified ‘*Improving the existing defences’* (Option 1.4) and ‘*a New barrier at Long Reach*’ (Option 3.2) as ‘front runners’ for the period beyond 2070, with ‘Improving the existing system’ (Option 1.4) being recommended until 2070 (EA 2012, p.49).
18. The *Options Appraisal* showed that taking no-regrets measures first would cost-effectively ‘buy-time’ before it was necessary to make more irreversible investments (e.g. a new barrier), and give time to monitor and gain additional knowledge to make better decisions (Reeder and Ranger 2011, p.11). An investment in a new barrier was delayed, the Plan suggests 2070 as the date for such barrier to be operating under the *Defra06 central scenario* (Bloemen et al. 2018).
19. The *Action Plan* presents recommendations on how the TE2100 Plan must be implemented, what, where and when diverse actions and activities must take place, and who will be involved (EA 2012, p.35). The Action Plan describes the actions and activities required, their location in the TE (at estuary-wide level and local level), the time-horizon when these will be implemented (short-, medium- or long-term), the nature of the proposed works, and the actors involved (*implementation partners*, their responsibilities, partnerships, and arrangements) (EA 2012, p.41). Drawing on the *Estuary-wide Options*, the Action Plan specifies the actions that must be taken at local level, within each ‘policy-unit’(p.36).
20. Together, the *route-map*, the *decision-points* and the monitoring system, formed the basis of the first 40-year investment plan, which details the works needed for upgrading the FRM system (Ranger et al. 2013, p.252). The first 40-year investment plan outlines the actions to upgrade the existing flood defence system (Ranger et al. 2013, p.252), and it will guide the implementation of actions even under more extreme scenarios (Reeder and Ranger 2011, p.10).
21. A decision will need to be made around 2050 (based on Government guidance) so that changes to the FRM system can be planned and ready for use by 2070. Although the climate and other conditions may change until the 2050 review, the Team had a high degree of certainty about FRM requirements for the next 40 years, hence, it prepared a detailed investment programme up to 2049, and a high-level programme up to 2100. The detailed planning for the next 40 years was based on *Option 1.4*. (EA 2012, p.56). The Team carried out a detailed planning for the next 40 years based on *Option 1.4* (EA 2012, p.56). The Team had high confidence in the short- and medium-term programme (up to 2049), and it expected that, by 2070, a major intervention in the FRM system will be required (based on the scenario considered), such intervention (which will provide a FRM solution from 2070 up to the 22^nd^ century) will be chosen by 2050, and planned, designed, and built between 2050 and 2070 (EA 2012, p.41).

    It is expected that major interventions in the existing flood defence system will not be necessary until 2070, and that the TB, with some modifications, will continue to provide flood protection up to 2070 (EA 2012, p.32, 5), yet, it is necessary to maintain the high standards of operation and maintenance of, and also improve, the existing defence system. Substantial improvements in the existing system will be needed before 2070 (e.g. *raising the crest level of most flood defences*, and *replacing a large proportion of the defence structures as they reach the end of their lives*), thus, the Plan contains a programme of continual maintenance and improvement works needed in first 40 years (EA 2012, p.32). Regardless of the measure chosen for the ‘end of century’, until 2070 it will be necessary to raise defences upriver and downriver of TB (Ramsbottom and Sheppard 2017, p.11). *Long term planning - looking beyond the year 2100:* The Plan includes *a continuous programme of activities and interventions from 2010 to 2069*, but *in 2070 a major change will be required, potentially requiring the construction of different flood defence structures* (EA 2009A, p.xiii)
22. The final Plan (2012) defined the *Options* (pathways) assuming the *Defra06 scenario* (*most likely* scenario), but it is ***flexible and adaptable*** to change and uncertain future: the Team sought to ensure that options were not limited if the future evolves in different ways than expected when the Plan was developed (EA 2009b, p.4).
23. The horizontal bars in the route-map showed when an intervention (a measure or portfolio of measures) starts and when it ceases to be of value and a new intervention must be implemented, yet, the decision to implement it must be made years or decades earlier, in this way, it was possible to estimate the date of implementation-points and decision-points under the scenario used (based on Government guidance) (EA 2012, p.38; EA 2009A, p.xiv). Many decisions should be made ahead of when measures are needed, as measures have a lead-time of years or decades (EA 2012, p.34, 38). The estimation of a decision-point considers the lead-time required for designing and constructing such action (HM Treasury 2009, p.26).

    Each of the Estuary-wide FRM Options contains a sequence of interventions over the next 100 years, and to develop the Plan and its associated investment plan, it was necessary to estimate the dates when these interventions must take place; however, in practice, the rate of future changes is unknown (it may be faster or slower than the rates assumed, and thus, the dates when interventions are required will change), therefore, an approach to estimating decision-points and implementation-points was needed which takes account of uncertainty in future change, and enabled decisions to be made based on actual rates of change (EA 2009A, p.277).
24. Relative SLR was a critical indicator, but other nine indicators were also considered (Ranger et al. 2013, p.253).
25. If one or more indicators change significantly, this might trigger a movement of the implementation-point and decision-point of each intervention. Notwithstanding, there are several different ways through which the Plan can respond to changes identified each time the Plan is reviewed and updated, i.e. different ways through which the Plan is adaptable to change (EA 2012, p.39; EA 2009A, p.267, 278-279).

    The Plan’s recommendations were based on Government’s 2009 guidance on CC. If CC predictions (or other pressures) worsen, then the date of a major intervention might occur earlier than 2070 - this is allowed for in the Plan (EA 2009A, p.xiii). Moreover, if a 10-yearly review of the Plan identifies that a different Option is then preferred, this is possible as long as the final decision is made with sufficient lead-in time to implement the recommendation (an act of parliament may be required, for instance); whatever the current end of the century recommendation, it is important to have a preferred Plan as basis from which stakeholders can plan for the future (EA 2009A, p.xiii).

    *The TE2100 Plan* ***will be reviewed every 10 years*** *so that it remains fit for purpose and* *to review the strategic aims to ensure they remain relevant; the* ***10 indicators will be monitored*** *through a formal programme and* *reviewed against the Plan* (EA 2009A, p.xv). *At each 10-yearly review and update of the TE2100 Plan, the front runner(s) Options* (for the period post-2070) *will be reviewed – up until the time (about 2050) when a firm decision must be made on the end of the century Option* (EA 2009A, p.xxi). The monitoring of “triggers for change”, together with a programme of review and maintenance of the Plan itself was included in the Plan, to ensure it is fit for purpose during its life (EA 2009A, p.262). *The monitoring programme of indicators for change must be maintained, and the preferred approaches (Options) to FRM should be reviewed using the TE2100 indicators for change and the Plan should be updated every ten years (or more frequently if there is a major change in one or more of the indicators)* (EA 2009A, p.263). Monitoring of key indicators (e.g. SLR) forms a vital component of the Plan; monitoring results must be used to update the Plan (e.g. the exact date when major interventions will be required will depend on the rate of CC and other changes as these are monitored) (EA 2009A, p.267). *It is therefore essential that the Plan is adaptable, to take account of future change and to avoid commitment to a course of action that may be sub-optimal because future change differs from the assumptions made in the Plan* (EA 2009A, p.267). The dates estimated for interventions are likely to change (the actual rates of change are unlikely to be the same as the assumed rates of change), thus, key indicators of change will be monitored as part of the Plan, and the dates will be modified as the Plan is updated (EA 2009A, p.268). *If significant changes occur in the expected dates when thresholds will be reached, the choice of Options should be reviewed* (as an alternative Option may be more effective under the changed circumstances). The preferred Options and the alternative Options must be re-appraised, using the updated best estimates of future change, which may lead to a change in the selected Option. Moreover, while alternative Options are included in the Plan, it is wise to consider whether there are any other alternative options whenever the Options are reviewed. (EA 2009A, p.279-280). *As the rates of change in the indicators are monitored, it is possible to assess their impacts on dates when interventions will be needed*; in this way, decision-points and changes in the dates when decisions are needed can be made (e.g. changes in the rate of increase in peak surge tide level may have an impact on the dates (timing) when major interventions may be needed) (EA 2009A, p.286, 287-289). *The monitoring results must be used to estimate the dates of new decision-points* (the actual future change may have impacts on the date of decision-points of interventions pertaining to an Option) (EA 2009A, p.287). *It is also necessary to consider whether the preferred options that were identified in the 2009 are still the best; appraisal will be needed to decide the best way forward whenever the Plan is updated (if alternative is adopted, some of the works in the preferred Option may no longer be required, thus the design and construction work should take account of the possibility of future change)* (EA 2009A, p.290). *As changes in climate and the TE occur in the future (differing from the baseline assumptions, the fundamental requirement for applying the method for adapting the Plan* (outlined in the decision-points) *is to understand how the estuary functions and responds to change* (EA 2009A, p.290-291).

    *The Estuary-wide Options can be adapted to different amounts of future change by changing the dates when the ‘portfolios’ are implemented; and, if at some point in the future, physical, environmental or socio-economic changes in the TE are outside the limits which can be accommodated by a chosen Option, an alternative Option will be implemented* (EA 2009A, p.297). (the two main ways through which the Option / pathway can be adapted)
26. A key factor for the efficacy of the APs approach is the development of long-term records of climate information and data on sea levels, so that changes can be detected and any necessary alterations to the Plan (and route-map) can be timely made (Reeder and Ranger 2011, p.12). The TE2100 implies an ongoing monitoring, and a review of decisions over time, as observations and new knowledge (e.g. updated projections) arise.
27. The Plan was designed to be adaptable to demands put on the FRM system through changing climate, socioeconomic change and fragile natural environments (EA 2009A, p.xv); the *Plan is designed to adapt to changing climate or other factors over the next 100 years. This means that our Plan is sustainable, will not waste money on over-engineered solutions and will ensure that the right actions can be carried out at the right time* (EA 2009A, p.262).
28. At each 5-year or 10-year review, depending on the magnitude of change observed in a given indicator through monitoring, the whole strategy (Plan) could be reappraised in the light of new information, to see if a switch to one of the other HLOs is recommended by CBA (HM Treasury 2009, p.27-28). Every 5-10 years, the strategy is revisited, if monitoring in the intervening period reveals that CC is happening more quickly (or slowly) than predicted at the time the Option was developed, the decision-points may be brought forward (or put back). This ensures that adaptation decisions are made at the right time, to keep benefit-cost relationships close to those envisaged at initial appraisal (HM Treasury 2009, p.26).
29. In Step 1, it also is important to translate objectives into tasks for the short- and long-term; and define metrics / parameters to describe changing conditions, e.g. the rate of SLR in cm/year (Gersonius et al.2016, p.204-206).
30. These uncertainties concern external factors and their possible future changes, e.g. uncertainties about future (climatic and socioeconomic) changes that may influence the future decisions taken, namely about SLR, river discharges, etc. (Gersonius et al. 2016, p.205). In Step 1, it also is important to: analyse the current policy measure; and define metrics / parameters to describe changing conditions, e.g. the rate of SLR in cm/year (Gersonius et al.2016, p.204-206).
31. Step 2 consists of the identification of possible measures to manage risk – i.e. risk management / adaptation measures (to reduce the risk probability, or the systems’ vulnerability, and / or increase resilience) – for short- and long-term future conditions (Marchand and Ludwig 2014, p.3).
32. Step 2 involves: (a) exploring all possible or relevant measures around an ATP, and identifying measures that can be used to meet the objectives and tasks.; and (b) evaluating each measure’s performance under different future scenarios (and changing conditions) and against pre-defined criteria and standards, this includes assessing their efficiency and cost-effectiveness (costs, effects and benefits) over the different scenarios); and the cost-effectiveness and efficiency of measures are influenced by mid- / long-term developments (e.g. land use changes, or construction in areas for FRM measures) (Gersonius et al. 2016, p.204-206, 202).
33. Short-term decisions should avoid an unnecessary increase of future costs; and it is necessary to efficiently link measures for the short- and long-term, i.e. short-term measures must be coherently linked to long-term objectives and tasks, they *must be logical in the long-term*; and this implies preparing measures to keep open options for the long-term (Haegen and Wieriks 2015), in addition, measures can be grouped in sets with a specific rationale (e.g. protection via hard defences).
34. ADM is based on DAPP. In DAPP, one of the main ingredients to design an adaptive plan is the development of adaptation pathways (APs). The AP method requires exploring the ATPs of measures. AP and ATP are key methods in DAPP (Haasnoot and Jeuken), as they are in ADM.

    This step requires building adaptation pathways as flexible routes to achieve the goals, rather than ‘blueprints’ (Zevenbergen et al. 2018).
35. This step requires an assessment of the *flexibility* of the pathways, and a scanning of options to keep open (Zandvoort et al. 2018, p.189). The APs’ map provides insight into potential lock-ins and options that should be kept open. It is important to explore opportunities to mainstream FRM / adaptation measures with other planned investments, e.g. in urban renewal. The consideration of opportunities to mainstream adaptation may require adjusting the timing for implementing measures (Gersonius et al.2016).
36. The pathways are assessed on their effectiveness, costs and side-benefits. In addition, in the assessment of the pathways, two important factors must be considered: a pathway’s *robustness* and *flexibility*. The goal is to elaborate *robust flexible strategies*. A *robust strategy* is one that achieves the desired objectives under diverse future scenarios. An important tool for making a strategy robust is flexibility; flexibility can be delivered by having the possibility to adapt strategies as necessary over time – e.g. adjusting measures or implementing new measures – and still meet the objectives. The APs map shows *transition-points* (conditions under which it is necessary to switch from one measure to another, or their moment in a scenario). It is important to assess which measures must be taken now and which can be postponed (Brugge and Bruggeman, p.4-5)..
37. An adaptive plan / policy is considered **robust** if the desired objectives can be achieved under a variety of circumstances (i.e. scenarios), whereas flexibility is related with the easiness with which a plan can be adapted to changing conditions and the capacity to adapt to a multitude of possible future conditions and changes. The flexibility of a plan is enhanced if its measures can be adapted, implemented earlier or later than planned and meanwhile the plan’s goal will still be reached (Deltares 2018, p.2-3), if it is possible to shift from one measure or pathway to another.
38. In Step 4, the results of the prior steps are used to formulate an *adaptive plan* that contains possible measures and specifies ‘triggers’ for modifying the strategies or measures; decisions will be made based on this adaptive plan (Brugge and Bruggeman, p.4-5). While the result of Step 3 is usually a map showing a subset of ‘promising adaptation pathways’, which includes possibilities for transferring from one pathway to another; Step 4a implies translating such map into a ‘plan of action’, which requires deciding which pathways should be followed and kept open and analysing the implications for short-term actions (Gersonius et al. 2016, p.204-206).
39. Importantly, in each annual cycle, research work (its concretization and scheduling) is conducted in parallel with decision-making (which is based on research results of the prior year); e.g. the elaboration of the DP2012 was done in parallel with research work for the DP2013 (Werners et al. 2016).
40. This implied gathering studies on climate change effects, prioritizing effects according to their likeliness, severity and impact on DP’s objectives, and consulting experts in research institutes, ministries, etc.

    Werners et al. (2016) analysed the process of policymaking of the DP, focusing on the Subprogramme for the Wadden Region.
41. ADM is based on DAPP. In DAPP, one of the main ingredients to design an adaptive plan is the development of adaptation pathways (APs). The AP method requires exploring the ATPs of measures. AP and ATP are key methods in DAPP (Haasnoot and Jeuken), as they are in ADM.
42. An adaptive plan / policy is considered **robust** if the desired objectives can be achieved under a variety of circumstances (i.e. scenarios), whereas flexibility is related with the easiness with which a plan can be adapted to changing conditions and the capacity to adapt to a multitude of possible future conditions and changes. The flexibility of a plan is enhanced if its measures can be adapted, implemented earlier or later than planned and meanwhile the plan’s goal will still be reached (Deltares 2018, p.2-3), if it is possible to shift from one measure or pathway to another.
43. Most *Preferential Strategies* include possible options for the long-term, some may not be required after 2050 but are included anyway as an ‘option to be kept open’ (DP 2013, p.102-103). In 2012/2013, during the development of the *Promising Strategies*, some options for the long-term were eliminated (e.g. a dam with locks in the Nieuwe Waterweg, a ring of floodgates in rivers around Rotterdam). Other options were kept open and used in the *Preferential Strategies* of 2013/2014 (e.g. new design requisites for the Maeslantkering, flood storage, etc.) (DP 2013, p.95).
44. In ADM, short-term decisions must be coherently linked to long-term tasks and goals, short-term measures *must be logical in the long-term* and do not hinder long-term options, and it may be necessary to prepare short-term measures to keep options open for the future (Haegen and Wieriks 2015)
45. The ‘social cost-benefit analysis’ (MKBA) takes into account the long-term costs and benefits of measures, using a discount rate that sets out a relationship between the current and the future value of the costs and benefits of a measure (DP 2013, p.105; 2014, p.137-138).
46. Flexibility, alongside other criteria, could play a role in the ultimate choice. The DP used ADM to design flexible strategies: flexibility is deemed crucial to be able to respond to uncertain futures (represented in the Delta Scenarios) (DP 2012, p.90, 107). By maximising robustness and flexibility, it is possible to avoid later costs. The DP commissioned a study on how flexibility could be weighted in investment decisions under uncertain futures (DP 2014, p.138). As the Route-map approach of the TE2100, ADM seeks to ensure that any short-term decision is taken within a framing that does not lead to maladaptation if the future unfolds differently from what is considered ‘the most probable future’ now (Gersonius et al. 2016, p.4). The appraisal of the pathways raised issues about how to valuate flexibility (the methods to do it) and what are its costs and benefits (Brugge and Bruggeman, p.7).
47. A trigger may indicate that it is necessary to activate *contingency actions* (to keep pathways open as long as possible) (Haasnoot and Jeuken). To keep open the preferred pathway as long as possible, it may be necessary to specify *contingency actions* (Jeuken et al. 2014, p. 4-5).
48. Every year the DP must issue its proposed *programme of measures*: with detail for the first 6 years, with an indicative outline for the next 12 years and a broad general view until 2050. This led to the creation of the *Delta Plans* on FRM and FS (DP 2014, p.8, 86).
49. It also defined specific *variables* to measure such indicators, namely: a) ‘*driving forces*’ (*drivers variables* that provide early signals for the tasks), and b) ‘*impacts of changes*’ on the system (i*mpact variables* that indicate consequences of a particular change and constitute a strong incentive to take action) (DP 2018, p.111).
50. This Section builds on: (i) the prior analysis of the TE2100 Project and Plan, its planning approach and process; the prior work of authors who examined the TE2100 to extract essential elements of its methodological approach and its ‘adaptive plan’ (e.g. Jeuken and Reeder 2011; Reeder and Ranger 2011; Ranger et al. 2013) or elements that allow the classification of the TE2100 as a successful example of an Adaptive Planning to FRM (Ramsbottom and Sheppard 2017); as well as studies that first identify elements of an Adaptive Planning and an *dynamic adaptive plan*, and then examine if these elements are present in diverse cases worldwide, e.g. TE2100 (Jeuken et al. 2014).
51. It defined a maximum acceptable flood probability (represented by the ‘Standard of Protection’), which is the level of protection provided by the flood defences expressed either as an annual flood probability (%) or a return period in years. This sets the threshold values of acceptable flood probability for the flood defence system that should not be exceeded. The Team identified the thresholds when the measures / portfolios of measures reached their maximum limit of flood risk (Ramsbottom et and Sheppard 2017, p.4-5).
52. The TE2100’s *Dynamic Adaptive Planning approach* aimed at building a *dynamic adaptive plan / strategy* that was: *robust* (i.e. ‘*performs adequately well against a set of decision criteria under a wide range of possible future states of the world*’), and *flexible* / *dynamically robust*’ (i.e. the Plan / Strategy, its measures, or their timing, *can be changed over time as more is learnt or as conditions change*) (Ranger et al. 2013, p. 247, 233, 249), and, thus *adaptable* (Ranger et al.239, 249, 254; EA 2012, p.35-38).
53. In the APs, it is possible to switch from a measure to a different measure, but also to switch from a HLO (pathway) to another (Jeuken et al. 2014, p.18).
54. Importantly, at each 5-year monitoring review and 10-year review, it is necessary to evaluate if a switch to another action or pathway is recommended by new cost-benefit analyses, given the rate of change observed in indicators (through monitoring) and new information available (e.g. updated scenarios). If SLR accelerates beyond expectations, it might be advisable to switch to *a more interventionist Option* (e.g. HLO4); such switch will be possible with no significant wasted money in early decades of the Plan (all HLOs contain small incremental measures first and leave major irreversible investments as far as possible into the long-term) (HM Treasury 2009, p.27-28). Overall, at each review, the chosen pathway may be changed, if the monitored indicators indicate so (a pathway suitable for a higher or lower scenario may be taken) (London Councils 2018).
55. The real pathway undertaken will result from a ‘mix’ of the best decisions taken at each moment over the Plan’s lifetime.
56. The adaptive approach is reflected in the way of managing flood risk - the *managed adaptive approach* – where several measures are introduced over time (at different moments) to keep risk below the target level, and alternatives are kept open to manage future risk (Ranger et al. 2013, 247-249; HM Treasury 2009, p.22; EA 2012, p.34; Ramsbottom and Sheppard 2017, p.3). The TE2100 is an *example of a well-founded decision to postpone* a major ‘irreversible’ action; the APs approach showed that there is still time before making a major intervention (Jeuken et al. 2014, p.18). Instead of rushing into the construction of major engineering defences, the TE2100 applied an adaptive approach (Penning-Rowsell et al. 2013, p.1383). In the APs approach, ‘*large-scale decisions can be made robust in the face of deep uncertainty* *over future climate* (Jeuken and Reeder 2011, p.4).
57. The Team developed an ‘*adaptable Plan with interventions sequenced through the century to adapt to future CC* (EA 2009A, p.73, xv). Each *Option* contains a *number of portfolios* to be *implemented in sequence*, to *provide a complete FRM solution for the next 100 years*, i.e. e*ach Option consists of a sequence of portfolios of responses* (EA 2009A, p.138, 152). Each *Option* is made up of sequenced portfolios over time *(each portfolio of responses reduces flood risk so that it stays below the target level)*, and *the dates at which one portfolio changes to another portfolio depend on key thresholds for FRM assumed* (*key-thresholds* include, e.g. *the maximum design water level at the TB; the maximum design water level at other parts of the defence system; the number of closures per year of the TB or other new barriers reaching 50*) (EA 2009A, p.139, 149-150, xi-xii). The scheme of the TE2100’s *Managed Adaptive approach* illustrates this: flood risk will increase over time, thus, a target level of risk is established; then *‘each ‘portfolio of responses’ reduces flood risk so that it stays below the target level’ (it is necessary to implement a portfolio before the target level of flood risk is reached*); and, in this way, an *Option* will emerge (each *Option* consists of a sequence of *portfolios* over time) (EA 2009A, p.139, xii, 150-151). Each *Estuary-wide Option* comprises a *sequenc*e of interventions (or a sequence ‘*portfolios of responses*’) over the next 100 years (EA 2009A, p.277, 297), hence, *FRM activities or interventions are sequenced through the century* (EA 2009A, p.262). Overall, the Team sought to develop an *adaptable Plan* composed of *Options*, each Option made up *with interventions sequenced through the century to adapt to future climate change* (EA 2009A, p.xv, 73). Thus, consideration was given to *how to manage the response to future change when the magnitude and nature of future change is not yet known, and how to turn this into an* ***adaptable programme of activities sequenced through the century*** (EA 2009A, p.82).
58. Low-regrets measures are measures that *work through the whole range of change* (Reeder and Ranger 2011, p.6). In the TE2100, ‘low-regret measures’ were those that reduce risk immediately and cost-efficiently under a wide range of SLR scenarios. They are ‘low-regret’ rather than ‘no-regret’ because they may involve some opportunity cost (in Ranger et al. 2013).
59. E,g. the measure ‘*raising other defences in the TE*’ (present in several *HLOs*) is a low-regret measure that helps to extend the lifetime of the existing defence system and ‘buy time’ before it is necessary to make a more irreversible decision on the long-term measure (rather than making an more ‘inflexible’ decision now) (Reeder and Ranger 2011, p.9, 11; Ranger et al. 2013, p.255). The measure ‘upgrade existing flood protection infrastructure’ was detailed in the 40-year investment plan (Reeder and Ranger 2011, p.9).
60. *Flexibility* was possible, in part, because the TB was designed to provide a high standard of protection up to 2030 (adaptation was not urgent, and delaying a major investment is justifiable as its benefits are highly uncertain now) and *low-regret* measures were available (Ranger et al. 2013, p.255).

    The *Options* (pathways) are apparently flexible into the future, in part, because new large-scale structural measures could be postponed. Other types of measures (e.g. soft protection, flood-proofing buildings), which can be applied in small steps, contribute in part to the Plan (it contains mostly measures to improve the existing defence system) (Jeuken et al. 2014, p.18).
61. The main way of updating the Plan will be to change the dates of a measure, and not the measure, but if a significant change occurs in the date when a threshold is expected to be reached, the choice between measures or between pathways (*Options*) may be reviewed, especially if an alternative pathway is more effective under the new changed circumstances. Thus, both the *preferred options* and the alternative options must be re-appraised (using updated estimates of future change), and a different *Option* may be chosen – this is why alternative *Options* have been included in the Plan (Ramsbottom and Sheppard 2017, p.13).
62. The mid-term monitoring review process (every 5 years) assesses the changes (physical and socioeconomic) occurring in the Estuary over time, based on the 10 indicators. This monitoring review process is essential to evaluate if the FRM policies and recommendations of the Plan need to be adjusted (EA 2016, p.5). Although the monitoring review occurs every 5 years, if the monitoring (in the interim period) reveals that a given indicator is changing faster (or slower) than what was expected, the ‘implementation-point’ and ‘decision-point’ of a measure may be anticipated (or postponed) (HM Treasury 2009, p.26-27; Ranger et al. 2013, p.254; Reeder and Ranger 2011, p.10). In practice, the decision-points and implementation points of measures (and the measures themselves, and the pathways) may be modified in light of new information that arises on indicators (HM Treasury 2009, p.27).
63. The success and effectiveness of the TE2100 Plan strongly depend on this process of: continuous monitoring of local and global changes and conditions, and regular revaluation (reassessment) and review of the Plan, and adjustment as time unfolds (adapting the Plan and the physical system to changes early in time) (EA 2009b, p.10; Ranger et al. 2013, p.254; Reeder and Ranger 2011; Lowe et al. 2009, p.85, 90; Met Office 2012).
64. There are various ways to respond to changes, as these are monitored, or each time the TE2100 Plan is reviewed or updated (EA 2012, p.39), e.g. by adjusting the date of *implementation-points* and *decision-points* of measures, or by shifting of measure (or pathway), or altering (changing) the measures themselves.
65. These elements are interrelated and are also deemed the main innovations of the TE2100, and arose from a long process of public engagement and learning (Ranger et al. 2013).
66. The TE2100’s *Dynamic Adaptive Planning approach* contained the APs method as a component and as a novel method for drawing up a *dynamic adaptive plan* both *robust* and *flexible* (Ranger et al. 2013, p.249; EA 2009b, p.200).
67. Each *Option* is *a number of portfolios which, when implemented in sequence, provide a complete FRM solution for the next 100 years* (for the duration of the Plan) (EA 2012b – SEA – Strategic Environmental Asssessment *in* Bloemen et al. 2018, p.8; Ramsbottom and Sheppard 2017, p.2, 5; EA 2009A, p.138). Hence, each *Option* is *made up of sequenced portfolios over time* (each portfolio reduces flood risk so that it stays below the target level, as illustrated in the scheme of the *Managed Adaptive approach* of the TE2100) (EA 2009A, p.139, 149-150, xi-xii).
68. The Team has developed *a series of generic Estuary-wide options which can successfully manage flood risk through the century*; the Plan sets out *the range of options which can manage a change in water levels through this century* (EA 2012, p.34, 6). The Team could identify *a series of adaptation pathways*, a route-map, that are appropriate to cope with the plausible range of climatic changes that could be seen by 2100 (Reeder and Ranger 2011, p.5). The TE2100 Plan has *a set of options (adaptation pathways)* (Bloemen et al. 2018, p.7). The TE2100 Plan provides ‘*a set of Options*’, including the *preferred Options* but also alternative *Options* (which might be necessary if the preferred *Options* are no longer effective because the actual rate of change differs from the expected (EA 2009A, p.279).
69. The APs approach served to identify possible measures and explore their timing and sequencing over time, and, in this way, design several different possible pathways of adaptation, which were then tested on their suitability under different scenarios and refined (Ranger et al. 2013, p.249). Importantly, the APs approach focused on *sequencing a suite of measures in order to cost-effectively manage current risk while maintaining the flexibility to cope with the range of possible future SLR* (Reeder and Ranger 2011, p.13).
70. The TE2100 Team sought to safeguard and *build-in dynamic robustness*, *flexibility* and *adaptability*, into the general Plan and its strategies (Ranger et al. 2013, p.249), which was done through the APs approach. The APs helped to incorporate *robustness* and into the Plan and its *strategies* (pathways) in several ways, for example: through the ability to change between measures and/or *Options*, by allowing adapting Options (e.g. adjusting the timing of new measures, according to the rate of change experienced, or adjusting measures themselves), by ensuring the availability of alternative *Options* (*alternative ways of managing flood risk*), by envisioning a sequenced / stepwise implementation of several measures over time (rather than an optimal solution for a particular level of risk) and keeping open possibilities to switch to other measures or *Options*.
71. The Technical Report also highlights that the Team devised a *methodology which has led to the concept and development of ‘flexible, adaptable Options’ for FRM* – this has been ‘*fundamental to, and the basis of, the adaptability of the TE2100 Plan*’ (EA 2009A, p.71; xv). The production of ‘*adaptable Options*’ as ‘*flexible decision pathways*’, and the threshold analyses that it implied, were essential to develop a plan (to manage flood risk until 2100) that was *dynamic robust* and *adaptable* to uncertain future conditions and changes – and fundamental to make *dynamic robustness* (*flexibility*) and *adaptability* basic properties of the Plan (EA 2009b, p.2-4, 10, 199). The APs approach was crucial to draw up a Plan with these properties.
72. The development of *robust flexible strategies*, which was done using the APs method, is considered one of the main elements of the TE2100’s *Dynamic Adaptive Planning approach* (Jeuken and Reeder 2011, p.2, 4). The TE2100 used the APs as a method of designing *robust flexible strategies*, where *robustness* to uncertain future changes and *flexibility* could be ‘built-in’ in the strategy itself (Jeuken and Reeder 2011, p.4, 2; Reeder and Ranger 2011, p.3, 8, 11). Thus, it can be argued that the design of *robust flexible strategies* as pathways (*flexible adaptable Options*), by using the APs method, is not only a key element of the TE2100’s approach but also an essential element to develop a *robust adaptive plan*.
73. The decision-points can be estimated based on the observation of certain key-indicators: if a trigger-value predefined for certain indicator is reached, this will trigger a decision (Ranger et al. 2013, p.233, 239, 258). To estimate decision-points, the Team used several scenarios to assess when it will be necessary to adapt at earliest and at latest, and took into consideration the lead-time required for designing and constructing a certain measure, as well as the monitoring results on a certain indicator (observed changes) – in this way, it was possible to see if a decision should be postponed or taken earlier (Jeuken and Reeder 2011, p.6). *Decision-points* help to ensure that measures (to manage evolving risk) are timely taken and cost-effective (Ranger et al. 2013, p.233, 239, 258).
74. The regular reviews of the Plan must be informed by monitoring results (Reeder and Ranger 2011; EA 2012, p.36; EA 2009b; Ranger et al. 2013, p.254; Bloemen et al. 2018; Ramsbottom and Sheppard 2017, p.13). The monitoring results must be used to periodically update of the Plan, e.g.: the dates when actions are needed, or if necessary, its pathways (Options), and / or their actions (Ramsbottom and Sheppard 2017, p.13). In this way, the potential need of periodic updates of the *Options* (adaptation pathways) could be addressed (Bloemen et al. 2018).

    At each 10-yearly review and update of the TE2100 Plan, the *front runner(s) Options* for the period post-2070 will be reviewed – and around 2050 a firm decision must be made on the *end-of-the-century Option* (EA 2009A, p.xxi). As part of the regular monitoring and of the periodic planned revision of the TE2100 Plan, it will be necessary to keep the ranking of *Options* under review (EA 2009A, p.232); thus, at each 10-year review, the *preferred Options* must be reviewed using the results of the monitoring of indicators (EA 2009A, p.262-263). Moreover, the design standards of protection for flood defences, the FRM Policy, or floodplain management activities, might need to be changed, as time unfolds – these issues will need to be monitored over time and revisited as part of regular reviews of the TE2100 Plan (EA 2009A, p.232).

    The monitoring of indicators is **vital component** of the Plan; monitoring results must be used to update the Plan’s contents (e.g. the exact date when major interventions will be required will depend on the rate of CC and other changes as these are monitored) (the dates estimated for interventions are likely to change, as the actual rates of change are unlikely to be the same as the assumed rates of change, the indicators must be monitored as part of the Plan, and the intervention-dates must be modified as necessary and the Plan updated) (EA 2009A, p.267).

    As indicators are monitored, *monitoring results must be used to update the estimated dates when ‘portfolios’ must be implemented as well as the dates when decisions must be made* (EA 2009A, p.280). The *preferred Options* and the *alternative Options* must be re-appraised, using the updated best estimates of future change, which may lead to a change in the selected *Option*. Moreover, while the Plan contains alternative Options, it will be necessary to consider whether there are any other alternative options whenever the *Options* are reviewed (EA 2009A, p.280), i.e. it is necessary *to consider whether the preferred options* identified in 2009 *are still the best; appraisal is (…) needed to decide the best way forward whenever the Plan is updated* (EA 2009A, p.290).
75. The indicators must be monitored, and the Plan must be regularly reviewed (based on the monitored outputs), to ensure that the Plan remains *adaptable to change*, *flexible*, and *fit-for-purpose* over its lifespan, and responds adequately to changes (EA 2012, p.36,38, 39; EA 2016; EA 2009A, p.262, xv). The ongoing monitoring of local and global changes, and the regular reviews of the Plan (including the re-appraisals and updates of its diverse contents) help to ensure that the Plan remains *adaptable* (Ramsbottom and Sheppard 2017, p.5, 12-13).
76. Overall, the Plan *can accommodate future change and can be adjusted when the rate of change is either faster or slower than originally predicted* (EA 2016, p.15).
77. The TE2100’s monitoring and review system (M&R) seeks to ensure that the Plan is regularly reviewed and updated to reflect the existing circumstances, and it is crucial for the Plan’s ***adaptability*** (EA 2009A, p.11); the monitoring of indicators, in particular, is deemed *a key part of the adaptability of the TE2100 Plan to future change* (EA 2009A, p.8). As noted in the Technical Report, given the uncertainty surrounding the effects of CC, *it will be essential to have* ***adaptability and flexibility*** *as a key part of the Plan*, which points to *the need for the ongoing monitoring of CC science and the actual effects of CC, particularly on SLR*, as part of the implementation of the Plan (EA 2009A, p.72).
78. The phase of implementation has included *monitoring activities together with a programme of review and maintenance of the Plan itself to ensure it is fit for purpose during its life* (EA 2009A, p.262). The monitoring programme established in the Plan is deemed essential to detect rates of change and identify triggers (thus, it is an important component of the ‘blueprint for implementation’), and the produced data must be regularly used to update estimates of the dates when new interventions will be required (EA 2009A, p.11). The phase of implementation has included *monitoring activities together with a programme of review and maintenance of the Plan itself to ensure it is fit for purpose for the duration of its life* (EA 2009A, p.262). The Plan was designed to adapt to changing climate or other factors over the next 100 years and be *sustainable*, thus, it must ‘*ensure that the right actions can be carried out at the right time*’ and ‘*not waste money on over-engineered solutions*’ (EA 2009A, p.262), and the M&R system is fundamental to achieve this.

    Thus, it can be argued that defining and conducting the M&R programme is another key-element of the TE2100’s *approach* required to develop and operationalize a *robust adaptive plan* and fulfil a real *Dynamic Adaptive Planning*.
79. There are various ways to respond to changes, as these are monitored, or each time the TE2100 Plan is reviewed or updated (EA 2012, p.39), e.g. by adjusting the date of *implementation-points* and *decision-points* of measures, or by shifting of measure (or pathway), or altering (changing) the measures themselves.
80. This section builds on the prior analysis of the DP, of ADM and its process, and on studies that analysed the application of ADM in the DP to extract essential elements of this approach (Jeuken and Reeder 2011), and on authors who identify the main elements an Adaptive Planning to then check if these are present in the DP (e.g. Jeuken et al. 2014; Restemeyer et al. 2017).
81. In 2010, the DP started to develop a set of plausible future scenarios, so-called *Delta Scenarios*, which would be used for all studies in the DP (DP 2010, p.4). The *Delta Scenarios* were issued in 2011**,** enabling further agreement on the range of future effects that should be considered (Werners et al. 2016).
82. In the DP, *scenario development* started with the formulation of a central question: which external events, circumstances, and changes / developments, are critical to FRM and FS (Marchand and Ludwig 2014, p.16-17).
83. The *Delta Scenarios* were generated by the scientific community based on climatic and hydrological model calculations (Jeuken et al. 2014, p.11, 14). The *Delta Scenarios* correspond to plausible futures and were based on the KNMIs climate scenarios (2006) and the socio-economic scenarios of the collaborating planning bureaus (2006) (DP 2011, p.71). The first version of these scenarios was created in 2011 by combining the climate scenarios from the KNMI (Royal Netherlands Meteorological Institute) with socio-economic scenarios from collaborating planning offices (DP 2012, p.35). In 2013 and 2014, the latest insights from the IPCC Report which were also incorporated into the 2014 KNMI climate scenarios; such scenarios are very similar to the 2006 KNMI climate scenarios, which formed the basis for the *Delta Scenarios* (DP 2014, p.135).
84. Prior to the DP, the 2^nd^ Delta Commission considered ‘high-end’ scenario of SLR of 1,3 m (Jeuken and Reeder 2011, p.7), however, it caused public debate, hence, when the DP was initiated, it was abandoned. Instead, the DP decided to use the formal Dutch climatic scenarios, which cover a large range of plausible futures but not high-end projections (Jeuken et al. 2014; Vink et al. 2013, p.97). Although worse / more extreme scenarios were available, the DP Staff decided to work with the *Delta Scenarios* (plausible scenarios) to develop the *Strategies*, and then the selected strategies were tested under more extreme scenarios (Restemeyer et al. 2017, p.930).
85. All subprogrammes worked with the same 4 *Delta Scenarios* (DP 2012, p.35; Werners et al. 2016).
86. A new element of ADM compared to AM is the inclusion of *Adaptation Tipping-Points* (ATPs). ATPs are defined as *points where the magnitude of change due to external pressures such as sea level rise or peak discharges is such that the current strategy will no longer be able to meet the objectives and thus the measure is no longer adequate* (Kwadijk et al. 2010). Combining the defined ATPs with CC scenarios provides information about the need for additional measures. The ATP analysis can, therefore, help to develop Adaptation Pathways (Haasnoot et al. 2012, 2013).
87. According to Jeuken and Reeder, planning for adaptation requires questioning how vulnerable is a certain context (e.g. a FRM system) to CC, how much CC can the system handle, and whether it is necessary to take new / additional measures because of CC or socioeconomic developments (Jeuken and Reeder 2011, p.2).
88. Jeuken and Reeder (2011, p.4) define ATPs as ‘*points where the magnitude of change due to CC or SLR is such that the current strategies will no longer be able to meet the objectives and alternative strategies are needed*’. The driver for taking measures is not CC *per se* but being unable to meet objectives, indeed, CC is only one of the issues that may alter the date of an ATP, e.g. socioeconomic developments may also lead to earlier or later ATP (Jeuken and Reeder 2011, p.4).
89. When the DP was initiated, the Netherlands already had FRM and FS policies / plans for long-term in place, e.g. the ‘Room for the River’, the ‘Coastal Defence Strategy’, and the ‘Water Management Programme for the 21^st^ century (WB21)’. These policies and their measures had to be taken into account by the DP when assessing thresholds (Jeuken and Reeder 2011, p.7).
90. To determine moment to take a decision (so-called ‘decision-point’), the Teams used various scenarios, and took into account the lead-time required for planning and implementing a measure and its complexity*.* The ‘decision-point’ is reached earlier than the ATP (Jeuken and Reeder 2011, p.6). The decision-moments and implementation-moments depend on the ATPs of measures, but also on earlier opportunities to combine measures with other objectives, investments, and agendas (Jeuken and Reeder 2011, p.5).
91. To identify ATPs, the DP Team and Subprogrammes addressed the question of ‘*how long the current policy will suffice*’ and ‘*when the tipping-point (at which a policy is no longer tenable) will be reached*’ (DP 2010, p.68).
92. As mentioned, the design the adaptation pathways required the use of the ATP’s method, which involved analysing at which level of a climate-related variable (or other relevant parameter) a given measure is no longer suitable or acceptable and must be substituted by an alternative measure (e.g. specifying a physical threshold at which a transition of measure is needed), and, then, identifying the moment in time of such ATP by placing it (the threshold level) under different (climatic and socioeconomic) scenarios (Klijn et al. 2015). By crossing ATPs with scenarios, it was possible to obtain information on the timing for new measures. The analysis of ATPs was, thus, essential to develop pathways (Zevenbergen et al. 2018).

    According to the *ADM Implementation Guide*, to develop *robust* *flexible strategies*, it is necessary to identify tipping-points and design adaptation pathways (by ordering possible measures in time); in specific, to make strategies *flexible*, it was important to know ‘*when to take action or change course*’, i.e. when an ATP might be reached (Rhee 2012, *in* Restemeyer et al. 2017, p. 931).
93. The *ADM Implementation Guide* (Rhee 2012) suggests working with Scenarios, Tipping-points, and Adaptation Pathways (*in* Restemeyer et al. 2017, p.930); identifying tipping-points and making adaptation pathways (which involved ordering various possible measures in time) were supposed to help in finding (devising) *robust and flexible strategies* (Restemeyer et al. 2017, p.931). The DP’s ADM approach used scenarios, tipping-points and adaptation pathways *to make strategies more ‘robust’ and ‘flexible’* (Restemeyer et al. 2017, p.935).
94. The DP aimed to develop *strategies* that were both *robust* and *flexible* (DP 2013, p.103; 94).
95. To design the adaptation pathways, the method of Haasnoot et al. (2012, 2013) could be used; pathways could be designed as series of sequenced measures that provide a solution to problems (*in* Brugge and Bruggeman).
96. The DP’s ADM approach combines existing insights into working with several potential strategies, called *adaptation paths*, and appraising flexibility around such solution-paths. In the DP, ADM involved developing a schematic overview with adaptation paths for tackling the tasks in a relevant area, and, in this, it assumed a bandwidth of plausible futures (Delta Scenarios), thus, starting from the current situation, the scheme charts out the next decision, moves further ahead and looks at possible amending or adaptive strategies at a later stage, including conditions under which it will be wise to shift strategies (DP 2011, p.48).
97. The *adaptation paths* were created by putting the first measures and the long-term possible options in a logical chronological order (DP 2012, p.61).
98. Each *Preferential Strategy* includes possible options for the long-term, some may not be required after 2050 but are included anyway as an ‘option’. E.g. until 2050, the DP intends to gradually adapt the existing FRM system to changing conditions (SLR, increasing river discharges). This implies gradual changes in the system, e.g. in the management, maintenance and replacement of defences, in spatial organization, and in specific sectors (shipping, agriculture). After 2050, larger-scale interventions in the system will be required, which will be carried out according to the then-existing climatic and socioeconomic conditions. A final decision on the post-2050 measures was not taken in 2014, but preparations were made for the case such measures are necessary. This required anticipating long-term conditions (DP2013, p.102).
99. The DP sought to ensure that the chosen strategies and measures provided flexibility to respond to new insights and changes, for example, in terms of ‘*stepping up measures*’ or ‘changing strategy’ if necessary (DP 2014, p.6).
100. For instance, in the Preferential Strategy for the RE-D, by switching between ‘*more dyke reinforcements’* and ‘*river widening’*, it will be possible to cope with the projected CC (indeed both measures are part of the current strategy) (Jeuken et al. 2014, p.18).
101. As the lifetime of many defences comes closer (many are expected to reach the end of their technical life in the coming years), it will be necessary to implement new / additional measures, and the map of *adaptation pathways* shows the measures available (Alphen 2015, p.313).
102. However, for Restemeyer et al., the *Preferential Strategy of the RE-D* looks quite ‘*determined and not so flexible’*: it offers little room for adjustments over time, and it focusses mainly on ‘prevention’ through a *gradual adjustment* of the existing defences. Although the Team recognized the need for spatial adaptation measures in addition to the traditional preventive measures, in this Strategy, ‘*adaptability mainly gets down to gradual adjustments of certain measures*’, e.g. dyke strengthening (Restemeyer et al.2017, p.932, 934-935).
103. ADM seeks to avoid over- and under-investments, and insight into pathways was useful for this. One way of reducing the risk of over-investment is to delay a measure until more knowledge is available (a hurried implementation reduces the possibility of including new knowledge). However, it also important to avoid taking measures too late (under-investment). The lead-time required to plan and implement measures must be considered, to avoid under- and over-investments (Marchand and Ludwig 2014).
104. In this sense, they can be deemed sub-elements or ingredients of this 3^rd^ key-element (the development of *a robust and flexible set of actions*, through the ‘APs method’).
105. ADM seeks to avoid *over- and under-investment* (DP 2010, p.70; 2011, p.8; Haegen and Wieriks 2015; Brugge and Bruggeman), it aims at implementing measures ‘*neither too early or too late, too much or too little’* (DP 2014, p.49; 2011, p.16, 45; 2012, p.88; Klijn et al. 2015; Vink et al. 2013), ensuring that the right measures are taken at the right time (DP 2013, p.102; 2012, p.88; Gersonius et al. 2016; Zandvoort et al. 2018). ADM aims to ensure that the necessary measures are taken, whilst keeping open options that might be required in the future (Rhee 2012; Werners et al. 2016; DP 2014, p.49; 2011, p.16, 45; 2012, p.88).
106. It can be argued that this was not only one of the ADM principles, but also an element essential to develop a *robust adaptive strategy* (in each *Preferential Strategy*) and to make the DP an *adaptable policy-programme*.
107. Each pathway is a sequence of actions over time to achieve the defined objectives (Marchand and Ludwig 2014, p.2, 13, based on Haasnoot 2013). ADM builds on the idea of developing diverse trajectories that the dynamic system might follow as time unfolds, i.e. various pathways (Zandvoort et al. 2018, p.190). According to this principle, adaptation pathways should be created with successive actions over time, rather than a final solution defined for a certain point in the future (Rhee 2012, *in* Klijn et al. 2016, 2015). Adaptation pathways offer ‘*coherent sequences of measures and potential options, which may be triggered before an ATP occurs*’ (Zevenbergen et al. 2018, based on Haasnoot et al. 2012, 2013). Once a tipping-point is in sight, a switch to a new measure is needed, and, in this way, a pathway arises; the various pathways provide possible trajectories to achieve the objectives (Haasnoot et al. 2012, *in* Zandvoort et al. 2018, p.191). In ADM, pathways are like ‘*successions of measures over time*’, which are explored under many plausible transient scenarios of CC and socioeconomic development (Haegen and Wieriks 2015, p.50).

     With the APs method, the Subprogrammes could assemble sequences of measures and plan the activation of such measures (Zandvoort et al. 2018, p.189). A pathway is a sequence of measures that may be activated before an ATP occurs (Gersonius et al. 2016, p.204).
108. Each of the *Promising Strategies* (of each Regional Subprogrammes) offers a cohesive, logical set of promising strategies (DP 2012, p.81).
109. Each *Preferential Strategy* (per region) delivers a set (or sets) of measures, which includes measures planned for the short-term linked to other measures for the mid- and long-term (DP 2013, p.56).
110. The *Preferential Strategies* contain the measures for each area; each *Preferential Strategy* defines aims / goals, and it specifies measures to meet the objectives in the short-, mid- and long-term, and concrete specifications for each region) (DP 2012, p.35; 2013, p.16, 32, 36, 94; 2014, p.6, 47, 49, 46; 2013, p.94; 2016, p.6). Each Regional Subprogramme developed a *Preferential Strategy* for FRM for its region (DP 2014, p.8, 46, 132).

     A *Preferential Strategy* contains measures to address FRM tasks in the short-, mid- and long-term (DP 2014, p.49, 6). Within the measures for the long-term, there may be: (1) measures *already certain to be required in the long-term* (e.g. dyke improvements in places where no alternatives are available); and (2) measures not yet certain to be needed and that will depend on climatic and socioeconomic developments (DP 2014, p.49). The final decision on the measures for the long-term will be taken later and will depend on the developments in the next decades. This is in line with one of the mottos of ADM: making what is needed now and identifying which measures can be implemented if the conditions change. Thus, a *Strategy* usually contains ‘options’ left open for future decision (envisioning long-term options in plans is a key feature of ADM) (DP 2014, p.49, 62). This also helped to identify which locations might be needed for implementing such measures in the future, and to prevent developments that could render such measures unviable. Dutch government can use several tools for this purpose, e.g. by defining ‘area reservations’ in a *General Rule on Spatial Planning* called ‘Barro’ (DP 2014, p.49, 62).
111. In each *Preferential Strategy* and in most *Delta Decisions*, there is a map of ‘*adaptation path(s)*’. In the DP, each *Preferential Strategy* contains a set of measures sequenced over time, i.e. a pathway. The adaptation pathways are called ‘*adaptation paths*’. Each *Preferential Strategy* has a map with one or more *adaptation paths*. Each *adaptation path* (pathway) is a set of several sequenced measures over time, i.e. a sequence of actions for the short- (2030), mid- (2050) and long-term (2100).
112. The map of *adaptation paths* shows the measures available for the short-term (2030) and mid-term (2050), and possible options to keep open for the long-term (up to 2100); it may include (anticipatory or preparatory) measures required to keep open options for the long-term (e.g. measures necessary to shift from the current measure to another in the future, like research studies on a particular option) (Gersonius et al. 2016 p. 211). In each *Preferential Strategy*, the map of *adaptation path(s)* identifies measures that must taken in the short-term and possible measures (options) for the long-term on which a decision will be taken in due time.
113. The APs’ approach also served to identify *decision-moments* (Jeuken and Reeder 2011, p.6; Jeuken et al. 2014, p.10). In the DP, the moment for implementing a new measure and the associated *decision-moment* not only depend on thresholds and ATPs of prior measures, but also on opportunities to combine such measure with other investments thematically and spatially (e.g. dyke reinforcements combined with urban projects), on the lead-time required to plan and design the measure, and its complexity (the *decision-moment* occurs prior to the ATP) (Jeuken and Reeder 2011, p.6-7).
114. In synthesis, in the DP, strategy-making was based on the ADM approach. ADM builds on an important element to develop strategies: the *construction of pathways*. ADM, and the design of pathways, also require the assessment of the flexibility of measures and pathways, the scanning of options to keep open, and the combination of measures with investments and agendas of diverse actors (Zandvoort et al. 2018, p.190). These aspects are patent in the DP’s Strategies for FRM.
115. The Subprogrammes assessed the *flexibility* of the strategies in a simplified way, for instance, by examining if it is easy to realize measures step-by-step or adjust them to accommodate to changes as these occur (DP 2012, p.88,90). Each Subprogramme should assess if its *Strategy* was *robust* and *flexible*, among other aspects (DP 2013, p.95). The *Preferential Strategies* were subjected to a *robustness assessment* under worst-case events beyond the scope of the *Delta Scenarios* (e.g. a SLR greater than 85cm in 2100), which showed that the *Delta Decision on the Rhine-Meuse Delta*, the *Decision on Sand*, and all *Preferential Strategies*, were satisfactory even in worst-case events (DP 2014, p.135, 8).
116. for example: P1 was met through *paths* that connect short-term measures to long-term tasks; P2 is ensured e.g. / mainly by taking measures *step-by-step* and *leaving options open*; P3 reflects in the design of *adaptation paths* as part of each *Preferential Strategy* (a map of *adaptation paths* was produced in each *Preferential Strategy*, indicating *what* and *when* measures are necessary; and, depending on actual developments, measures may be changed, switched, taken sooner or later) (DP 2014, p.47).
117. As noted by Restemeyer et al. one of the main conditions for making a policy plan ‘*adaptive*’ is the elaboration of *flexible* strategies, and recent literature suggests three interrelated tools to make long-term plans more adaptable - scenarios, tipping-points, and adaptation pathways (Restemeyer et al. 2017, p.922, 923, 935).
118. Jeuken and Reeder note that a key element of the Adaptive Planning approach applied in the DP, i.e. of the ADM approach, was *finding flexible strategies (that do not block further adaptation measures when necessary)*, and *avoiding irreversible investments as much as possible* (Jeuken and Reeder 2011, p.2-3), by using the ‘APs approach’.
119. As the seriousness and timing of CC are uncertain, the DP decided (in 2011) that it would create a monitoring system to provide early signals of CC and detect thresholds / tipping-points (Werners et al. 2016).
120. Importantly, although the DP operates in *a dynamic environment*, with ongoing developments, a *continuous adaptation of all the strategies to those developments is neither feasible nor necessary*, and this implies finding an appropriate *rhythmicity for the adaptation of strategies and plans* (DP 2016, p.61).
121. The results of the 1^st^ review were expected to be published in DP 2020 (for 2021), and explain to which extent the goals set out in DP2014 are being achieved in time (DP 2018, p.22).
122. Importantly, prior to the MAA system, there was already a monitoring system for evaluating national water policies (Jeuken et al. 2014, p.22). Moreover, the law already defined that the condition of dykes and dunes must be checked every 6 years against projected water levels, storm surge conditions and predefined criteria (e.g. on the strength and height required). If dykes and dunes fail to meet these criteria, they must be improved considering the necessary robustness for the next 50 years. In the coast, more or less sand must be nourished depending on the results of monitoring of the coastline position. In this case, thresholds are ‘a moving target’: they can vary over time depending on several aspects (claims on space available, public acceptance of measures, costs, non-quantifiable aspects) (Jeuken and Reeder 2011, p.8).
123. ADM differs from the traditional Adaptive Management. The scientific basis of ADM lies in *Adaptive Planning* (Kwakkel et al. 2012; Ranger et al. 2013), namely in Adaptation Pathways and Adaptation Tipping-Points (Werners et al. 2016; DP 2011, p.48,70).

     The new ‘adaptive delta planning’ mentioned by the DP Commissioner in 2010 (later renamed as *Adaptive Delta Management*) corresponds well with the *Dynamic Adaptive Policy Pathways approach* (DAPP) presented by Haasnoot et al. (2013); and the DP offered a study-case to test the applicability of the DAPP (Haasnoot et al. 2013, p.490). ADM is based on, and similar to, DAPP (which builds on *Adaptive Policymaking* (APM) and *Adaptation Pathways* (APs), however, it was developed in the policy context of the DP (Gersonius et al. 2016, Buuren et al. 2016); the scientific basis for ADM also lies in the concepts of *Adaptive Planning* (Kwakkel et al. 2012; Ranger et al. 2013) and *Adaptation Tipping-Points* (ATPs) (Kwadijk et al. 2010) (*in* Werners et al. 2016). Moreover, the DP’s ADM is based on ‘Planned Adaptation’ (Petersen and Bloemen 2015). Overall, ADM stemmed from a combination of existent insights and work about ‘several potential strategies’, ‘adaptation paths / solution paths’, and into the appraisal of the flexibility of such paths (DP 2011, p.48,70). Besides that, other two examples served as source of inspiration: the TE2100 Project, and the programme of restructuring of Hamburg port area (DP 2011, p.45, 55). Inspired by TE2100 Project’s approach, the DP developed its own adaptive planning approach (ADM) (Zevenbergen et al. 2018).

     ADM is different from the Adaptive Management (AM) usually referred in literature (AM of Holling 1978), because: 1) it is anticipatory rather than responsive (it focusses on planning in anticipation rather than reactive responses); 2) it explicitly takes into account, and deal with, uncertainties about the future in the planning and management; 3) AM focusses on trial-and-error experiments and monitoring of developments, while ADM recognizes that, for some variables, the signals of CC cannot be derived from monitoring / measurement soon enough (due to large natural climate variability), hence, ex-ante assessments and anticipatory planning are needed; (4) the concepts of ‘robustness’ and ‘flexibility’ are essential in ADM (Klijn et al. 2016). A new aspect of ADM in relation to AM is the use of ATPs and APs (Zevenbergen et al. 2018).
124. Flexibility (capacity to be flexible) can be *flexibility* to switch to other strategies when needed, in the face of uncertain future changes, it has elements of experimentation and learning. Adaptive capacity refers to capacities, attributes, and resources to undertake adaptation; it concerns the ability to anticipate, innovate and generate experience in dealing with change and learn from mistakes (Haegen and Wieriks 2015).
125. To ensure a *sustainable and robust FRM* and cope with climatic extremes, the country should be *prepared for various scenarios* (DP 2014, p.6).
126. According to the *ADM Implementation Guide*, to develop *robust* *flexible strategies*, it is necessary to identify tipping-points and design adaptation pathways (by ordering possible measures in time); in specific, to make strategies *flexible*, it was important to know ‘*when to take action or change course*’, i.e. when an ATP might be reached (Rhee 2012, *in* Restemeyer et al. 2017, p. 931).
127. In ADM, ATPs are understood as ‘*points where the magnitude of change due to socioeconomic developments, climate change or sea-level rise is such that the current strategy* (measure) *will no longer be able to meet the objectives*’ (*in* Zevenbergen et al. 2018, p.4, based on Kwadijk et al. 2010; Haegen and Wieriks 2015, p.51). An ATP indicates conditions under which a given measure fails and other measure is needed (e.g. a level of SLR at which a storm surge barrier ceases to function); thus, a switch to a new measure is needed once an ATP is in sight (Haegen and Wieriks 2015, p.51). In ADM, ATPs correspond to points / conditions under which the existing (current) management actions / measures or policies become ‘*too expensive, technically impossible or societally unacceptable*’; the date of an ATP indicates an ‘expiration date’ at which a given measure or policy ceases to be feasible (Rhee 2012, p.18, *in* Zandvoort et al. 2018, p.190). More precisely, in ADM, ATPs indicate conditions under which a given measure ceases to be effective or acceptable (or no longer suffices), and a new measure is required (source).
128. According to Jeuken and Reeder, adaptation planning requires questioning how vulnerable is a certain context (e.g. a FRM system) to CC, how much CC can the system handle, and whether it is necessary to take new / additional measures because of CC or socioeconomic developments (Jeuken and Reeder 2011, p.2).
129. To identify ATPs, the DP Team and Subprogrammes addressed the question of ‘*how long the current policy will suffice*’ and ‘*when the tipping-point (at which a policy is no longer tenable) will be reached*’ (DP 2010, p.68). The DP used scenarios to *map out how much longer current policy and management are expected to suffice and when adjustments will be required; in other words, when will we reach the tipping points for our water system* (DP 2010, p.3-4, 32, 36, 68-69).

     In 2011/2012, the DP stimulated the Subprogrammes to assess *how much longer current policies and management practices were expected to suffice and when adjustments would be required* (Werners et al. 2016) – which is related with the identification of the first ATPs: a failure of existing policy or management measures.
130. In the DP, the *decision-moments* and *implementation-moments* depend on the ATPs of measures, but also on the lead-time required for designing and implementing a measure, and on early opportunities to combine measures with other investments (Jeuken and Reeder 2011, p.5). To determine moment to take a decision (so-called ‘decision-point’), the Teams used various scenarios, and took into account the lead-time required for planning and implementing a measure and its complexity*.* The ‘decision-point’ is reached earlier than the ATP (Jeuken and Reeder 2011, p.6). The decision-moments and implementation-moments depend on the ATPs of measures, but also on earlier opportunities to combine measures with other objectives, investments, and agendas (Jeuken and Reeder 2011, p.5).
131. The DP’s ADM approach utilizes scenarios, tipping-points and adaptation pathways *to make strategies more robust and flexible* (Restemeyer et al. 2017, p.935). One of the *building blocks* for ADM is *the development of adaptive pathways* (Marchand and Ludwig 2014, p.17).
132. To design the adaptation pathways, the method of Haasnoot et al. (2012, 2013) could be used; pathways could be designed as **series** of sequenced measures that provide a solution to problems (*in* Brugge and Bruggeman).

     The APs approach is one of the Adaptive Planning approaches (Haasnoot et al. 2012, 2013; Walker et al. 2013).

     To develop *flexible* *strategies*, the DP used the methods of ATPs and APs (the APs served to develop *flexible strategies*) (Jeuken and Reeder 2011, p.5). The method of adaptation pathways has been applied in all Subprogrammes (working with adaptation pathways) (Marchand and Ludwig 2014, p.27).

     The APs approach, as employed in ADM, is an adaptive approach to design ‘strategies’ as pathways – i.e. *to develop a set of measures along adaptive pathways* – which incorporates flexibility to switch to other measures and strategies when needed, in view uncertain future CC and socioeconomic developments (*an adaptive approach that allows for switching between strategies along adaptation pathways when needed in view of socio-economic developments or climate change*) (Haegen and Wieriks 2015, p.55, 54, 56).
133. Importantly, the development of the *Preferential Strategies* was based on the ADM approach and its four basic principles (DP 2014, p.47, 12; 2017, p.7; 2011, p.18). Such principles are patent in the *Preferential Strategies* in several ways, for example: P1 was met through *paths* that connect short-term measures to long-term tasks; P2 is ensured e.g. / mainly by taking measures *step-by-step* and *leaving options open*; P3 is reflected in the design of *adaptation paths* as part of each *Preferential Strategy* (a map of *adaptation paths* was produced in each *Preferential Strategy*, indicating *what* and *when* measures are necessary; and, depending on actual developments, measures may be changed, switched, taken sooner or later); **P4** was delivered by linking the FRM measures envisioned to other objectives and ambitions in the area (DP 2014, p.47).
134. A *Preferential Strategy* contains measures to address FRM tasks in the short- (2030), mid- (2050) and long-term (2100) (DP 2014, p.49, 6). A *Strategy* usually contains ‘options’ left open for future decision (envisioning long-term options in plans is a key feature of ADM); the final decision on the measures for the long-term will be taken later and will depend on the developments in the next decades. (DP 2014, p.49, 62).
135. Each pathway is a sequence of actions over time to achieve the defined objectives (Marchand and Ludwig 2014, p.2, 13, based on Haasnoot 2013). ADM builds on the idea of developing diverse trajectories that the dynamic system might follow as time unfolds, i.e. various pathways (Zandvoort et al. 2018, p.190). According to this principle, adaptation pathways should be created with successive actions over time, rather than a final solution defined for a certain point in the future (Rhee 2012, *in* Klijn et al. 2016, 2015). Adaptation pathways offer ‘*coherent sequences of measures and potential options, which may be triggered before an ATP occurs*’ (Zevenbergen et al. 2018, based on Haasnoot et al. 2012, 2013). Once a tipping-point is in sight, a switch to a new measure is needed, and, in this way, a pathway arises; the various pathways provide possible trajectories to achieve the objectives (Haasnoot et al. 2012, *in* Zandvoort et al. 2018, p.191). In ADM, pathways are like ‘*successions of measures over time*’, which are explored under many plausible transient scenarios of CC and socioeconomic development (Haegen and Wieriks 2015, p.50). A pathway is a sequence of measures that may be activated before an ATP occurs (Gersonius et al. 2016, p.204).
136. To develop *adaptation paths*, the Subprogrammes used the ATPs and APs methods (Zandvoort et al.2018). The design of the *adaptation paths* implied the identification of tipping-points (Buuren et al. 2016), i.e. it implied defining the conditions under which changing of measure is necessary (DP 2013, p.95). The design of *paths* implied the analysis of *Adaptation Tipping-Points* (ATPs, see Kwadijk et al. 2010); ATPs were essential for phasing the possible measures within each *Strategy* (*in* Jeuken et al. 2014, p.17). To design the pathways, the method of ATPs was applied: once an ATP was in sight, a new measure was needed (Haegen and Wieriks 2015, p.51).
137. For instance, the Subprogramme for the Rhine Estuary-Drechtsteden, in its *Preferential Strategy for FRM*, developed an *adaptation path* that indicates diverse measures and at what times these will be required (DP 2012, p.90; Gersonius et al. 2016 p. 211).
138. In the ADM process, **Step 4** requires specifying indicators and triggers (critical values at which it is necessary to activate actions, or make adjustments to the strategy or shift of measure) (Deltares 2018, p.2-3; Jeuken et al. 2014, p.4-5; Haasnoot and Jeuken; Marchand and Ludwig 2014; Brugge and Bruggeman, p.4-5); thus, in a broader sense, Step 4 implies the definition of a monitoring system that collects information on indicators (Haasnoot and Jeuken) and mechanisms to adjust the plan / strategy if necessary (Deltares 2018, p.2-3; Gersonius et al. 2016, p.204-206). Moreover, **Step 6** consists of monitoring and evaluating climatic, physical, and socioeconomic changes, implemented measures, and progresses in knowledge, and also regularly reassessing the Plan/Strategy, and, if necessary, reviewing or adjusting it (Haasnoot and Jeuken); the latter implies assessing if and when it is necessary to adapt the plan / strategy (e.g. shift from a measure to another, change strategies or measures, or anticipate / delay measures) (Deltares 2018, p.2-3, 5; Jeuken et al. 2014, p. 4; Brugge and Bruggeman, p.4-5). **Step 6** is essential to ensure an adaptive planning and management. The monitoring system indicates the rate and direction of risks and their implications for adjusting the plan, it allows the adjustment of the plan / strategy - its measures or their timing (Brugge and Bruggeman, p.4-5).
139. Importantly, the ADM process differs from the traditional adaptation planning approaches because the starting point of the analysis of ‘*how long will the current strategies be effective under different scenarios*’, which leads to the exploration of alternative measures and the design of multiple solution-pathways (rather than a single solution that is designed for a ‘business-as-usual’ scenario or worst scenario) (Deltares 2018, p.4, 3). Moreover, ADM is different from the traditional Dutch practice of flood risk management because it requires an *adaptive flood risk management* (Klijn et al. 2015, p.848); indeed, with ADM, the DP aims to manage risks and respond to potential problems *in a timely and adaptive manner* (DP 2018, p.14). ADM is also different from the traditional *Adaptive Management* approach; the scientific basis of ADM lies in *Adaptive Planning* (Kwakkel et al. 2012; Ranger et al. 2013), namely in Adaptation Pathways and Adaptation Tipping-Points (Werners et al. 2016; DP 2011, p.48,70). ADM also differs from the ‘classical’ approach where a robust solution is often chosen based on business-as-usual and worst-case scenarios (Brugge and Bruggeman, p.2). The new ‘adaptive delta planning’ mentioned by the DP Commissioner in 2010 (later renamed as *Adaptive Delta Management*) corresponds well with the *Dynamic Adaptive Policy Pathways approach* (DAPP) presented by Haasnoot et al. (2013); and the DP offered a study-case to test the applicability of the DAPP (Haasnoot et al. 2013, p.490). ADM is based on, and similar to, DAPP (which builds on *Adaptive Policymaking* (APM) and *Adaptation Pathways* (APs) (Gersonius et al. 2016, Buuren et al. 2016); the scientific basis for ADM also lies in the concepts of *Adaptive Planning* (Kwakkel et al. 2012; Ranger et al. 2013) and *Adaptation Tipping-Points* (ATPs) (Kwadijk et al. 2010) (*in* Werners et al. 2016). Moreover, the DP’s ADM is based on ‘Planned Adaptation’ (Petersen and Bloemen 2015). Overall, ADM stemmed from a combination of existent insights and work about ‘several potential strategies’, ‘adaptation paths / solution paths’ (DP 2011, p.48,70). Inspired by TE2100 Project’s approach, the DP developed its own adaptive planning approach (ADM) (Zevenbergen et al. 2018; DP 2011, p.45, 55). ADM is different from the Adaptive Management (AM) usually referred in literature (AM of Holling 1978), because: 1) it is anticipatory rather than responsive (it focusses on planning in anticipation rather than reactive responses); 2) it explicitly takes into account, and deal with, uncertainties about the future in the planning and management; 3) AM focusses on trial-and-error experiments and monitoring of developments, while ADM recognizes that, for some variables, the signals of CC cannot be derived from monitoring / measurement soon enough (due to large natural climate variability), hence, ex-ante assessments and anticipatory planning are needed; (4) the concepts of ‘robustness’ and ‘flexibility’ are essential in ADM (Klijn et al. 2016). A new aspect of ADM in relation to AM is the use of ATPs and APs (Zevenbergen et al. 2018).
140. Flexibility (capacity to be flexible) can be *flexibility* to switch to other strategies when needed, in the face of uncertain future changes, it has elements of experimentation and learning. Adaptive capacity refers to capacities, attributes, and resources to undertake adaptation; it concerns the ability to anticipate, innovate and generate experience in dealing with change and learn from mistakes (Haegen and Wieriks 2015).
141. Moreover, the DP highlights that a continual investment in FRM and ‘*sufficient financial resources* *are* *a condition for a dynamic implementation of the DP* (DP 2013, p.120; 2014, p.120).
142. Difficulty was found in determining tipping-points in situations where: a) there is large natural variability, b) intrinsically flexible strategies are used, c) there are no clear policy goals defined (Bloemen et al. 2018).
144. Restemeyer et al. examined to what extent the Strategy for FRM elaborated in the RE-D Subprogramme was flexible, and found that, in practice, the Subprogramme faced several difficulties in working with scenarios, ATPs and APs (Restemeyer et al. 2017).
145. This Strategy reflects the old belief that authorities are able to control flooding, but in case of technical failure of a dyke, the hinterland continues to be vulnerable. In this Strategy, ‘adaptability’ is ‘*limited to the idea of gradual adjustments*’ to existing defence system (i.e. ‘*gradual adjustments of certain measures’* like ‘dyke improvement’) in a business-as-usual approach, less focused on improving the capacity to deal with unexcepted events and reducing the vulnerability (Restemeyer et al. 2017, p.935).
146. Dordrecht is an exception; it follows a more integrated approach. Most of the Island is protected by a dyke ring, but many parts of it were difficult to improve due to the proximity of historic buildings. Before the DP, Dordrecht had searched for alternatives, taking forward the concept of “multi-layered FRM’ and its 3 layers (Restemeyer et al. 2017, p 934). Dordrecht is interested in ML FRM, because in case of failure of the primary defence system, flood impacts can be reduced via *adjusted spatial planning* and *evacuation plans* (Gersonius et al. 2015; Bloemen et al. 2018). This Strategy considers risk communication and preparedness measures, but it does not mention how flood risk and evacuation possibilities should be communicated to citizens; this is pertinent, as 57% of Dutch citizens feel badly informed about flood risk but trust the Government to prevent flooding (Restemeyer et al. 2017).
147. *The cases of TE2100 and Delta Program highlight how short-term decisions are explicitly coupled with long-term options using adaptation pathways or route maps with clearly marked thresholds defining when to decide to switch from one action to another, this ensures maximum flexibility* (Jeuken et al. 2014, p.24).
148. It should be noted that the flexibility offered by dyke improvement measures is limited because this type of measures is often more cost-effective than implementing multiple smaller phased / stepwise projects or investments. Actually, measures like “river widening” or “coastal sand nourishments” are better suited to be implemented in small steps (Jeuken et al. 2014, p.18). Nevertheless, the flexibility of FRM plans has often been undermined / jeopardized by the type and lifespan of some FRM measures implemented in the past and present (e.g. hard flood protection structures like dykes and storm surge barriers that remain in place for many decades). For instance, in both the TE2100 and DP2014 cases, the adaptation pathways maps offer a sense of flexibility into the future, but, in part, because new major hard defence measures were postponed. Besides, in both cases, soft protection measures (e.g. coastal sand nourishments), as well as ‘making room for the river’ measures, and flood-proofing building measures, which are more adequate to be implemented in small steps, have only contributed partially to the plans, which focused mainly on improving the existing flood defense systems (i.e. barriers, dikes) (Jeuken et al. 2014, p.18). While the flexibility of measures for improving hard defences (dykes, barriers) is often deemed low, in densely populated low-lying coastal cities and deltas with a very robust flood defence system already in place, a single large investment in dyke improvement is usually more cost-effective than multiple small investments (Jeuken et al. 2014, p.18, 24). Besides, dyke improvement measures can be deemed robust if they include safety margins and increase the resistance of the system (Jeuken et al. 2014, p.24).
149. According to Sayers et al. (2012), central elements / characteristics of Adaptive Planning are: a) measures (to respond to changes) should be effective under the widest set of possible future scenarios; b) measures should not unnecessarily hinder – foreclose or constrain – future options; c) targeted monitoring of relevant changes, including the continual reassessment of future scenarios, and redefine plans / policies (and their measures / actions) adequately (Sayers et al. 2012, *in* Jeuken et al. 2014, p.3, 16, 21). Jeuken et al. add that ‘no / low regret’ measures (i.e. that have properties like robustness, flexibility and reversibility) should be favoured (Jeuken et al. 2014, p.3). It is thus necessary a monitoring system that includes signposts (indicators) and triggers (informed by monitoring results, new scientific knowledge, and updated projections), which keeps track of drivers of changes, scientific progresses, and effects and effectiveness of implemented adaptation measures and policies / plans (Jeuken et al. 2014, p.22).

     Jeuken et al. (2014) proposed / assumed / argued that there are three main elements (main supposed characteristics) of Adaptive Planning: i) to prepare for a wide range of plausible future scenarios (climate, SLR, socioeconomic scenarios); ii) to respond to uncertain change with robust and flexible actions (a robust and flexible set of actions); and iii) to monitor relevant changes and reassess the plan accordingly (Jeuken et al. 2014, p.1, 23).
150. The adaptive approach is reflected in the way of managing flood risk - the *managed adaptive approach* – where several measures are introduced over time (at different moments) to keep risk below the target level, and alternatives are kept open to manage future risk (Ranger et al. 2013, 247-249; HM Treasury 2009, p.22; EA 2012, p.34; Ramsbottom and Sheppard 2017, p.3). The TE2100 is an example of a well-founded decision to postpone a major ‘irreversible’ action; the APs approach showed that there is still time before making a major intervention (Jeuken et al. 2014, p.18). Instead of rushing into the construction of major engineering defences, the TE2100 applied an adaptive approach (Penning-Rowsell et al. 2013). In APs, “*large-scale decisions can be made robust in the face of deep uncertainty* *over future climate* (Jeuken and Reeder 2011, p.4).
151. Low-regrets measures are measures that *work through the whole range of change* (Reeder and Ranger 2011, p.6). In the TE2100, ‘low-regret measures’ were those that reduce risk immediately and cost-efficiently under a wide range of SLR scenarios. They are ‘low-regret’ rather than ‘no-regret’ because they may involve some opportunity cost (in Ranger et al. 2013).
152. The measure ‘upgrade existing flood protection infrastructure’ was detailed in the 40-year investment plan (Reeder and Ranger 2011, p.9).
153. Geels (2004) refers to this as the “hardness” of a measure, which is related with the physical structure but also with its economic costs, e.g. sunk costs. Gerrits and Marks (2008) also demonstrated how the initial selection of hard defences (particularly dykes) tends to initiate a process of ‘increasing returns’ that makes it almost impossible to leave that measure: indeed dyke-based protection tends to be much cheaper than alternative options, because of its ‘sunk costs’, its deep interrelatedness with the landscape geometry and functions (*in* Buuren et al. 2016).
154. This also happens in US where levees are usually built by the US-Army Corps of Engineers, while their maintenance falls within the responsibility of the regional authorities. The National Committee on Levee Safety of the American Society of Civil Engineers (ASCE) estimated the cost of repairing or rehabilitating the US levees to the required level around $100 billion dollars (*in* Klijn et al. 2016).
